# Supplementary material for: Multi-schema computational prediction of the comprehensive SARS-CoV-2 vs. human interactome
Source: PeerJ. 2021 Apr 5;9:e11117. doi: 10.7717/peerj.11117 (PMC8029698; doi:10.7717/peerj.11117)
Supplement: Supplemental Information 1 [file peerj-09-11117-s001.pdf]

Supplementary Materials

Multi-Schema Computational Prediction of the Comprehensive SARS-CoV-2 vs. Human Interactome

Kevin Dick[1,2], Anand Chopra[3,4], Kyle K. Biggar[3,4], and James R. Green[1,2]

[1]**Department of Systems & Computer Engineering, Carleton University, Ottawa, ON, Canada K1S 5B6**
[2]**Institute of Data Science, Carleton University, Ottawa, ON, Canada K1S 5B6**
[3]**Institute of Biochemistry, Carleton University, Ottawa, ON, Canada K1S 5B6**
[4]**Department of Biology, Carleton University, Ottawa, ON, Canada K1S 5B6**

Corresponding author:
James R. Green

Email address: jrgreen@sce.carleton.ca

ABSTRACT

These supplementary materials provide extended and complimentary materials in support of the main research findings. First is an extended introduction covering coronavirus history and biology, followed by additional methodological details and results. We emphasize the novelty of the first usage of the Reciprocal Perspective method as Combination of Multiple Experts within this work. We additionally provide guidance on the interpretation and use of the landscape matricies for subsequent use in the identification of putative sites that may mediate a physical interaction. Finally, these appendix materials contain many of the intermediate and auxiliary findings of this work.

INTRODUCTION

The novel coronavirus (CoV) pandemic has galvanized the research community into the investigation of the SARS-CoV-2 virus and the COVID-19 disease it manifests in humans (Guarner, 2020). Research has progressed with unprecedented speed due, in large part, to the rapid determination of the SARS-CoV-2 genome and proteome. These data enable the research community to collectively contribute to the study and understanding of SARS-CoV-2 and its disease pathogenesis.

This family of viruses has previously emerged as lethal human pathogens during the 2002-2004 severe acute respiratory syndrome (SARS) and the 2012 Middle East respiratory syndrome (MERS) outbreaks. These human-afflicting coronaviruses (HCoVs) are concerning not only as causative agents of mild respiratory illness but additionally for the severity of disease. Notably, since the 1960s two HCoVs have been known to regularly circulate among the human population and cause 15-20% of the common cold cases (Monto, 1974). Besides human infections, CoVs are causative agents of disease in domesticated animals with a symptomology comprising respiratory disease and enteritis in the infected hosts. To demonstrate the range of species infected by this virus subfamily, well-studied CoVs include the canine respiratory CoV (CRCoV), the mouse hepatitis virus (MHV), the bovine CoV (BCV), the canine CoV (CCoV), and the feline CoV (FCoV) (Erles et al., 2003; Weiner, 1973; Bridger et al., 1978; Binn et al., 1974; Pedersen et al., 1984). As discussed later, animal species (including humans) serve as "factories" for CoV reproduction and evolution. Given the emergence of three HCoVs causative of severe disease of epidemic or pandemic proportions within the last two decades, it is critical to expand our fundamental understanding of these viruses to rapidly identify putative therapeutic targets, facilitate complimentary research, and inform public discussions.

Coronavirus Classification, Structure, Hosts, and Genome

The *Coronavirinae* and *Torovirinae* subfamilies of the *Coronaviridae* family belonging to the *Nidovirales* order of viruses (Payne, 2017). The CoV subfamily is further divided into the four genera Alphacoro-

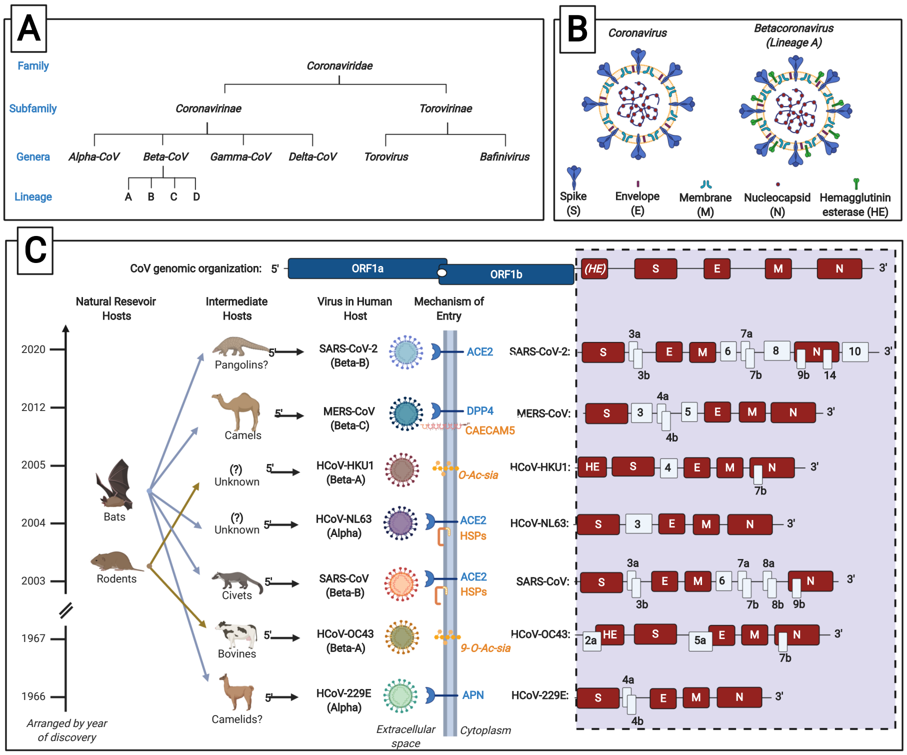

Supplementary Figure S1. Historical Overview of Coronavirus Biology. Panel (A) depicts a taxonometric tree of the family *Coronaviridae*. Panel (B) presents the structure and composition of coronaviruses; the Coronavirus structure depicted is representative of CoVs from all four genera and lineages, whereas the structure depicted by Betacoronavirus (Lineage A) is specific to CoV species within this taxon. Panel (C) depicts the discovery of HCoVs throughout history in combination with the animal origin and the known, suspected, or unknown intermediate host prior to infection and propagation within the human population. For each virus we depict the initial attachment (orange) and main fusion (blue) receptors utilized by HCoVs for cell entry. Finally, we illustrate the genomic organization of each of the HCoVs. ORF1a and ORF1b (blue) encode for polyproteins. A ribosomal frameshift occurs at a slippery sequence and pseudoknot structure located between ORF1a and ORF1b (open circle). Downstream open reading frames encode the Spike (S), Envelope (E), Membrane (M), Nucleocapsid (N), and Hemagglutinin Esterase (HE) structural proteins (red), as well as accessory proteins (light blue box, black text). Gene mappings not shown to scale. This figure was created with biorender.com.

navirus (αCoV), Betacoronavirus (βCoV), Gammacoronavirus (γCoV), and Deltacoronavirus (δCoV) (Payne, 2017). Species within the βCoV genera are further divided into four A, B, C, and D lineages (Figure S1A).

Coronaviruses share many similarities to the influenza viruses in that they are both enveloped, single-stranded, and helical RNA-viruses among the Group IV viral families (Baltimore, 1971). The four coronaviruses known to commonly infect humans are believed to have evolved to maximize proliferation within a population. This evolved strategy involves sickening, but not ultimately killing, their hosts. By contrast, the two prior novel coronavirus outbreaks (SARS and MERS) arose in humans after cross-species jumps from animals, as has H5N1 (the avian influenza). These latter diseases were highly fatal to humans, with a few mild or asymptomatic cases. A greater proportion of mild or asymptomatic cases would have resulted in wide-spread disease, however, and SARS and MERS each ultimately killed fewer than 1,000 people (World Health Organization, 2020; Regional Office for the Eastern Mediterranean, 2011).

To date, seven HCoV species have been identified (Figure S1). Beginning in the mid-1960s, HCoV-229E and HCoV-OC43 were the first HCoVs isolated from patients with mild respiratory illness (Hamre and Procknow, 1966; McIntosh et al., 1967). It was not until the start of the 21st century that the remaining five HCoVs were discovered (Figure S1). All known HCoVs arise from zoonotic origins (*i.e.* from other

animal species). The wide diversity of CoVs within the animal kingdom stems from the high genomic mutation rates high frequency of recombination between different CoV genomes (Makino et al., 1986; Van Der Most et al., 1992). In fact, different genotypes of HCoV-OC43 and HCoV-HKU1 have evolved due to natural recombination events occurring during human infection (Woo et al., 2006; Lau et al., 2011). Such genetic modifications occurring in animal CoVs facilitate a "host jump" and are the primary reason for inter-species and animal-to-human transmission (Cui et al., 2019). The HCoVs that are endemic to the human population are causative agents of more mild disease (*e.g.* common cold) and there is less urgency to identify the animal reservoirs of these viruses. Currently, the ecological route of transmission of these HCoVs into individual humans is likely entirely due to human-to-human transmission. However, similarity between these HCoVs and other animals' CoVs provides insight into their animal origins, modes of transmission, and cellular entry.

CoVs are enveloped viruses with a mostly spherical membrane approximately 120 nm in diameter and comprised of 4-5 structural proteins (Figure S1B). The single-stranded RNA genome is encapsulated by the Nucleocapsid (N) protein, which functions to package the viral genome into CoV particles during assembly (Chang et al., 2006). The Membrane (M) protein plays a central role in assembly of the viral particles, largely by promoting membrane curvature (Neuman et al., 2011). The Envelope (E) protein is multi-functional, playing key roles in viral assembly and maintenance, such as mediating ion-channel activity (Schoeman and Fielding, 2019). The specific naming of these viruses was due to the protrusions of approximately 20 nm above the virion surface which give the virus a crown-like appearance ("corona" in Latin) in electron micrographs (Lai and Cavanagh, 1997). These large projections are trimers of the Spike (S) glycoprotein, responsible for attachment and entry into target cells. Additional smaller 8 nm projections, composed of hemagglutinin esterase (HE) dimers are inherent to lineage A βCoVs (Figure S1B). The HE projections have implications in viral attachment and spread via attachment and modification of sugars, such as sialic acids, on target hosts cells (Klausegger et al., 1999).

The major viral determinant of cell entry is that of the 20 nm Spike protein projections. This projection is a trimer of the Spike glycoprotein, and different regions of this protein play a role in facilitating viral entry. The Spike protein has two main functionally distinct regions denoted as S1 and S2, which play roles in host cell attachment and membrane fusion, respectively (Heald-Sargent and Gallagher, 2012). Although these regions belong to the same polypeptide, a critical step in viral entry is the covalent separation of S1 and S2 by proteolytic cleavage at the S1/S2 boundary by host cell proteases. Proteolytic cleavage at the S1/S2 boundary is critical for activating the function of S2 to trigger viral-host membrane fusion and release the CoV genome into the cell.

The S1 region, responsible for cell attachment, is subdivided into two regions; the S1 N-terminal domain (NTD) and the S1 C-terminal domain (CTD, also known as the receptor-binding domain (RBD)). Overall, the S1 region specifies the range of hosts capable of interacting with CoVs through specific interactions with host cell surface biomolecules. The S1-NTD plays a role in initial adhesion to the cell surface via binding to sugars and adhesion molecules. This role of the S1-NTD has primarily been studied in the context of other animal CoVs (Krempl et al., 1997; Kubo et al., 1994), however it is also believed that at least three HCoVs (*i.e.* HCoV-NL63, SARS-CoV, MERS-CoV) utilize surface sugars or proteins as initial attachment receptors for adhesion to the cell surface Lang et al. (2011); Milewska et al. (2014); Chan et al. (2016).

The CoV RNA genome resembles that of a canonical eukaryotic mRNA, due to the presence of a 5'-terminal cap structure (methylated N7 position of the guanine cap and methylated ribose at 2'-O position of the first nucleotide) and 3'-terminal poly-adenine(A) tail (Chen et al., 2013). Within these large RNA genomes of approximately 27-32 kb, multiple open reading frames encode for the previously mentioned structural proteins, as well as polyproteins and accessory proteins (Figure S1C). The first two-thirds of the genome encode for pp1a and pp1ab polyproteins, translated from ORF1a and the -1 frameshifted ORF1b, respectively. Specifically, translation may continue through the -1 frameshifted ORF1b due to a slippery sequence and pseudoknot structure, enabling translation of pp1ab (Baranov et al., 2005; Brierley et al., 1989). These polyproteins are further processed into 16 non-structural proteins (NSP1-16) by autoproteolytic activity inherent to NSP3 and NSP5 within the polyproteins (Ziebuhr et al., 2000). This is essential for formation of a viral replicase-transcriptase complex (RTC). NSP12 possesses RNA-dependent RNA polymerase (RdRp) activity within the RTC, whereas NSP7 and NSP8 function as processivity clamps. The last-third of the genome contains ORFs encoding for the structural proteins (*e.g.* S, E, M, N, and HE), as well as accessory proteins that are not essential for CoV life cycle. It is of

critical importance that the cellular entry mechanism and viral replication pathways of SARS-CoV-2 and the role of accessory proteins be rapidly elucidated to develop anti-viral therapies to mitigate the spread and infectivity of the virus in the present pandemic.

Computational Prediction of SARS-CoV-2 Targets

Promisingly, many computational approaches have been rapidly deployed to increase our understanding of SARS-CoV-2, including protein function, three-dimensional (3D) protein structures, and possible target regions for small inhibitory molecules (Senior et al., 2020; Smith and Smith, 2020). Two notable examples include the use of DeepMind's recently published AlphaFold protein structure predictor by Senior et al. (2020) to predict the 3D protein structure of each of the SARS-CoV-2 proteins, and the use of the SUMMIT high-performance computing (HPC) infrastructure to perform large-scale virtual docking simulations as a form of high-throughput screening to identify small inhibitory molecules (Smith and Smith, 2020). Given that the Spike protein from the original SARS coronavirus, SARS-CoV, is known to interact with the human Angiotensin-Converting Enzyme 2 (ACE2), current efforts are focused to better characterize the SARS-CoV-2 Spike protein and its putative interaction with the ACE2 protein.

The computational prediction of PPIs is a diverse field which encompasses multiple paradigms (e.g. sequence-, structure-, evolution-, and network-based methods) (Kotlyar et al., 2017). The shortcomings of one approach are often the strength of another and certain paradigms can be useful in generating insightful interaction interface information (Dick and Green, 2016). Here, we will discuss the two paradigms with specific relevance to the SARS-CoV-2 pandemic given the current focus of the research community in an effort to develop therapeutics that might slow the progression and impact of COVID-19. Structure-based methods require knowledge the 3D structure of each of the proteins from the set of known PPIs and also for each of the proteins for which one wishes to make inferences (Kotlyar et al., 2017). Consequently, these methods suffer from low coverage throughout a complete proteome and are generally unsuitable for comprehensive interactome predictions. Furthermore, many structure-based methods rely on *de novo* or template-based modelling, which tend to be computationally taxing. Promisingly, the DeepMind team that developed the AlphaFold computation protein structure predictor have publicly released their predictions of the 14 proteins in the SARS-CoV-2 proteome for use by the scientific community, enabling the use of structure-based prediction methods (Jumper et al., 2020). However, high quality structures are not available for all human proteins and, even with complete 3D structural information of each protein in both organisms' proteomes, the computational time complexity to elucidate all possible inter-species pairings make these methods prohibitive beyond modestly sized networks. Promisingly, these methods are highly complimentary to other prediction paradigms and can be applied following the initial screening using other, more computationally efficient and high-throughput PPI prediction methods.

At the other computational extreme, sequence-based predictors rely solely upon primary sequence data making them amenable to the investigation of proteome-wide networks. Furthermore, these methods tend to be highly efficient, where individual PPIs can be predicted in a fraction of a second.

The rapidity of our response is thanks in part to having produced an analogous study during the Zika Virus outbreak of 2015, where our sequence-based PPI prediction method (PIPE) was used to identify putative human-Zika inter-species PPIs and inform possible synthetic biology approaches for novel interventions and therapeutics Kazmirchuk et al. (2017). In the present study, of the $\sim$285,000 predicted pairs, we leverage three prediction schemas and two independent PPI predictors to select a highly conservative set of predicted interactions for each of the 14 SARS-CoV-2 proteins considered in this study resulting in the identification of several putative human protein targets. We publicly released these predictions and related meta-data for use by the broader scientific community in the following DataVerse repository: 10.5683/SP2/JZ77XA, Dick et al. (2020).

METHODS

Determining an Appropriate Per-Protein Decision Threshold

For each of the 14 SARS-CoV-2 proteins, we predicted their interaction with each of the 20,366 human proteins resulting in 285,124 unique predictions from each of the two predictors considered. While each method, through a form of cross-validation, might determinate a highly-conservative *global* decision threshold, we know from our work in (Dick and Green, 2018) that such thresholds are sub-optimal. Consequently, we for the first time, adapt the method for the determination of a global decision threshold

for the PIPE4 and SPRINT algorithms to a RP-inspired method to determine *local* decision thresholds on a per-protein basis.

From the predicted interactomes (leveraging the CPM), we can plot the rank-ordered distribution of the putative interaction scores involving each of the *single* SARS-CoV-2 proteins separately. This presents an opportunity to develop protein-specific local decision thresholds, where only those interactions scoring significantly above baseline are reported. These one-to-all score curves are based on the underlying assumption that we expect true SARS-CoV-2 vs. human PPIs to be rare, such that the vast majority of prediction scores should fall below the decision threshold. Furthermore, by also plotting the one-to-all curves for each human protein, we can apply the same local decision logic to the reciprocal perspective (while not performed within the *all* and *proximal* schema, this analysis is leveraged within the *RP-PPI* schema) (Dick and Green, 2018).

Thus, for each one-to-all score curve, a score threshold delineating the "high-scoring" pairs from the baseline was identified and used to determine the high-confidence predicted interactions. In the absence of known PPIs between SARS-CoV-2 and human, it is difficult to determine a suitable global decision threshold. By instead examining the morphology of the one-to-all score curves for both perspectives, we can qualitatively identify high-scoring pairs. This process can be further automated through the identification of the baseline/knee for each view under the assumption that true PPIs are rare and high-scoring, while non-interacting pairs tend to generate scores residing below the knee in the baseline. In Figure S2, we overlay the one-to-all score curves for each SARS-CoV-2 protein and "zoom" into the high-score/low-rank region to emphasize that the selection of a single global top-k or score threshold would inappropriately exclude relatively high-scoring pairs within specific SARS-CoV-2 proteins, while admitting too many low-scoring putative PPI for other proteins.

Furthermore, our use of the one-to-oll score curves assumes that the vast majority of pairs are not likely to interact and consequently the distribution of scores about its baseline represent a proxy for a statistical *null model*. That is, by considering the $\sim$20,000 naturally-occurring and biologically plausible sequences within the human proteome as our "null model", our identification of "significantly" high-scoring pairs would be a more robust comparison than to consider an alternatice statistical approach which would compare a predicted pair with an equivalent number (i.e. 20,000) randomly generated sequences that wouldn't be biologically plausible. It is important to note that our combined use of the one-to-all score curve and Kneedle algorithm is not a statistics-based method but rather a machine learning-based approach.

We automated the selection of this operational decision threshold for the 14 SARS-CoV-2 proteins using the Kneedle algorithm, applied to its top-1000 predictions, using a sensitivity parameter of 2.0. The cut-offs for each protein are tabulated in Table S1.

Predicted PPI Site of Interaction using PIPE-Sites & the New Similarity Weighted Landscape

The list of PPIs generated from both methods can be used to inform the design of anti-SARS-CoV-2 therapeutics by using peptide sequences from the predicted PPI site, which we refer to as the PPI-Site. We define the PPI-Site as the peptide sequence that is responsible for mediating a given PPI, which is here estimated using the PIPE-Sites method. A conceptual overview of the PIPE4 landscape matrix and PIPE-Site prediction is illustrated in Figure S3.

The Reciprocal Perspective Cascaded Classifier: Combination of Multiple Experts

In previous work, we demonstrated that the use of a the Reciprocal Perspective PPI cascaded classifier (RP-PPI) produced statistically significant improvement in performance (Dick and Green, 2018). Moreover, the RP-PPI method, as a cascaded machine learning algorithm, can we leveraged to combine features from multiple expert models. Here, for the first time, we jointly combine the features derived from the PIPE and SPRINT models and demonstrate the resulting improvement in performance as part of the *RP-PPI* schema. Furthermore, following from the work of Kyrollos et al. (2020), we implement the cascaded model as an eXtreme Gradient Boosting (XGBoost) regression model as opposed to the Random Forest classifier originally proposed.

To evaluate the performance increase of the combined classifier, we perform Leave-One-Family-Out cross-validation (LOFOCV), and plot the average Reciever Operating Characteristic (ROC) curve with confidence intervals of one standard deviation. Given certain families had relatively few PPIs, we omitted those with fewer than 50 PPIs from this analysis (a negligible number of pairs were left out). The

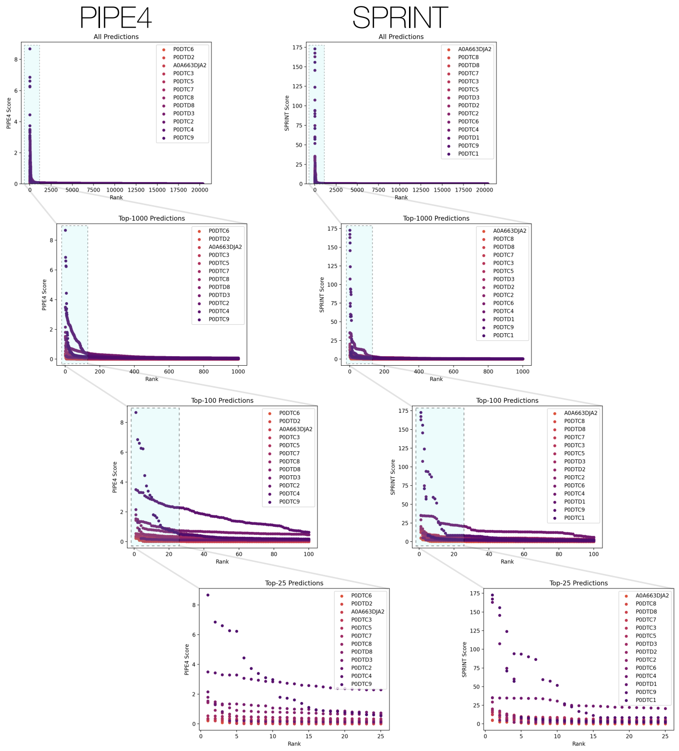

Supplementary Figure S2. **One-to-All Score Curves by Top-k.** The top panels depict the combination of one-to-all score curves for each protein, by each predictor and each subplot is a top-k subset of the previous; highlighted in blue. Selected example from the *all* schema.

determination that the combined use of PIPE4 and SPRINT features from their respectively predicted CPMs does, in fact, result in improved performance, we then performed extensive hyper-parameter tuning, evaluated via 10-fold cross-validation, to obtain the most performant model to then generate our SARS-CoV-2 vs. human predictions. Varying maximum tree depth ($[3,4,5,...,18]$), number of estimators ($[50,75,100,...,600]$), and the learning rate (9 values considered), we trained and evaluated 29,700 models to arrive to the final model that was used to generate the comprehensive set of prediction as part of the *RP-PPI* schema.

High-Performance Computing Infrastructure

In order to generate the $\sim 280,000$ PPI predictions for three independent schemas, high-performance computing infrastructure was required. Two https://www.computecanada.ca/ heterogeneous clusters were leveraged to generate these predictions: Graham and Cedar. The former has more than 41,000 cores and 520 GPU devices across 1,185 nodes and the latter boasts over 94,000 cores and 1,352 GPU devices across 2,470 nodes. In combination, these HPC clusters enabled the rapid computation and compilation of these predictions. Computational research related to the COVID-19 pandemic has been assigned increased priority which expedited the generation of these predictions.

RESULTS & DISCUSSION

It is of critical importance that the global research community focus its efforts on the rapid understanding the SARS-CoV-2 virus and the pathogenesis of COVID-19 in order to develop anti-viral therapeutics and vaccine targets. Fortunately, the prior decades of research into related viral families provide a wealth of data with which to guide current and future studies, such as with the elucidation of the SARS-CoV vs. human inter-species interactome in 2011 using the high-throughput (though false positive-prone) yeast-two hybrid method to highlight cyclophilins as a target for pan-coronavirus inhibitors (Pfefferle et al., 2011). Previous knowledge of related coronaviruses within the *Coronaviridae* family provide training samples with which we can identify a number of new high-confidence PPIs that contribute to our

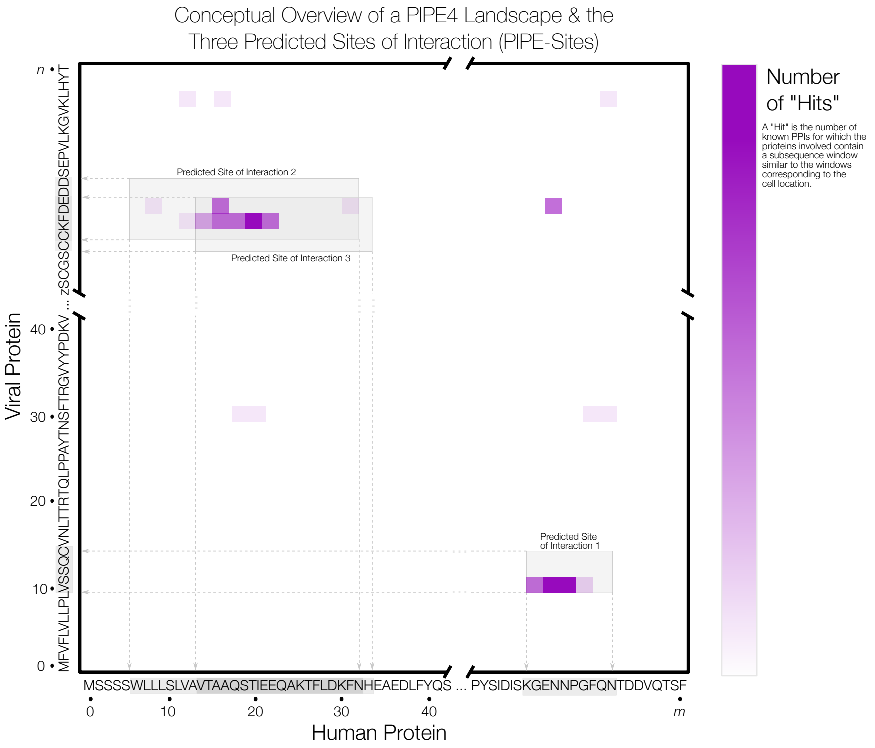

Supplementary Figure S3. **Conceptual Overview of the PIPE4 Landscape and the Three Predicted Sites of Interaction (PIPE-Sites)**

understanding of COVID-19 disease pathogenesis and which may represent targets for novel inhibitory therapeutics.

Predictions from the *All* and *Proximal* Schemas

As part of the first two schemas (*all* and *proximal*), for each of the 14 viral proteins, we sort the 20,366 scores (for each human protein) into a monotonically decreasing rank-order which enables the identification of the subset of high scoring putative interactors with that one viral protein.

Rather than apply a globally defined decision threshold (*i.e.* top-k or minimum threshold), we automatically detected a highly conservative "knee" for each curve (the point of greatest rate of change) to delineate those rare high-scoring pairs from the remaining baseline. For example, within the *all* schema, the union of the $n = 1,209$ predicted PIPE4 and SPRINT high-confidence putative PPIs comprises only ~0.42% of all possible pairs, and their intersection of $n = 279$ putative pairs comprises a highly conservative $< 0.098\%$. These data are tabulated in TableS1.

Predictions from the *RP-PPI* Schema

Following from the experimental design of the *all* and *proximal* schemas, the independent predictions from the RP-PIPE4 model and the RP-SPRINT models would have been combined into a single intersection set. However, we for the first time, jointly combined the RP features derived from the PIPE4 O2As with those derived from the SPRINT O2As to train and evaluate a "combination of muliple experts" (CME) RP-PPI model. The joint model (using default hyperparameter settings) demonstrated an improvement over the RP-predictor model alone. Interestingly, as illustrated in Figure S4 the improvement does not appear to be symmetric: the improvement of performance when SPRINT features are joined with the PIPE4 features (A, blue & grey) is greater than when the PIPE4 features are joined with SPRINT features (B, blue & grey).

Having established that the combination of multiple experts RP-PPI approach produces improved models, we performed extensive hyperparameter tuning to determine model parameters. Each experiment was evaluated via 10-fold cross-validation with performance measure using the F1 score. Following the

Supplementary Table S1. Summary of the Number of Predicted Interactions for the *All* and *Proximal* Schemas.

Schema	Predictor	SARS-CoV-2 Protein	Cut-Off Rank (*i.e.* Num. Predicted)	Cut-Off Score
All	PIPE4	P0DTC8	39	0.17616893
		P0DTC9	31	0.45052419
		A0A663DJA2	23	0.00868677
		P0DTD8	80	0.19851291
		P0DTD3	86	0.51450285
		P0DTD2	43	0.03406663
		P0DTC2	21	0.12438851
		P0DTC3	72	0.10978781
		P0DTC4	111	0.42306311
		P0DTC5	64	0.1712625
		P0DTC7	124	0.05241327
		P0DTC6	7	0.08283571
All	SPRINT	P0DTC8	117	1.43286
		P0DTC9	16	8.54969
		A0A663DJA2	22	0.164667
		P0DTD8	78	0.614801
		P0DTD3	37	5.10505
		P0DTD2	27	1.61548
		P0DTD1	17	2.47021
		P0DTC2	23	3.86361
		P0DTC3	28	3.64141
		P0DTC1	16	3.41798
		P0DTC4	48	13.5018
		P0DTC5	24	1.94603
		P0DTC7	44	2.61779
		P0DTC6	12	1.91867
Proximal	PIPE4	P0DTC8	80	0.05152177
		P0DTC9	12	2.08759585
		P0DTD1	2	4.52125180
		P0DTC1	3	3.72194125
		P0DTC2	20	0.14253816
		P0DTC3	53	0.02177234
		P0DTC4	13	0.12761254
		P0DTC5	81	0.27192328
Proximal	SPRINT	P0DTC8	78	0.84881
		P0DTC9	14	8.33724
		A0A663DJA2	68	0.0332739
		P0DTD8	54	0.15322
		P0DTD3	87	0.115683
		P0DTD2	71	0.361557
		P0DTD1	13	2.10023
		P0DTC2	31	1.08007
		P0DTC3	38	0.276883
		P0DTC1	17	2.42659
		P0DTC4	32	0.0546491
		P0DTC5	53	1.32324
		P0DTC7	104	0.183356
		P0DTC6	86	0.218939

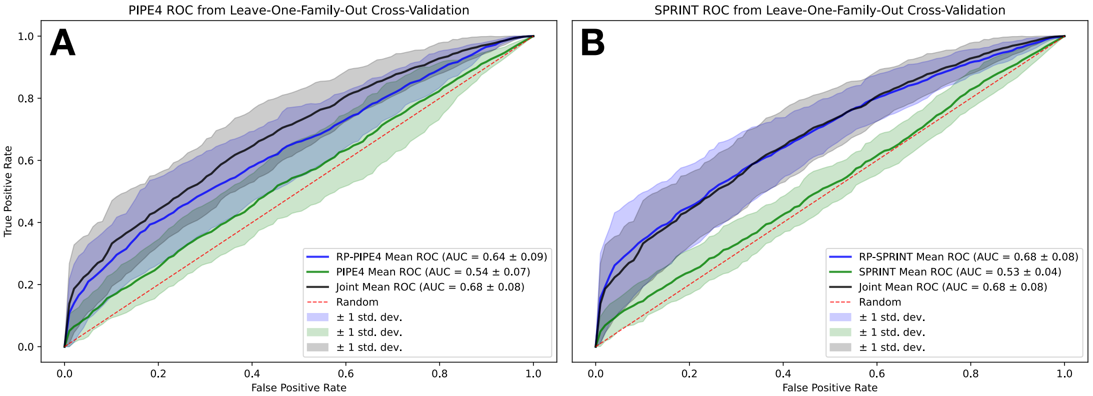

Supplementary Figure S4. RP-PPI Combination of Multiple Experts (Joint) Improvement in Predictive Performance using Leave-One-Family-Out Cross-Validation. The combined use of PIPE4 and SPRINT features within the RP-PPI Joint model depicts an overall average improvement in performance. Interestingly, the improvement does not appear to be symmetric: the improvement of performance when SPRINT features are joined with the PIPE4 features (A, blue & grey) is greater than when the PIPE4 features are joined with SPRINT features (B, blue & grey).

training and evaluation of 29,700 models, we identified the best performing model parameters as having a learning rate of 0.1, a maximum tree-depth of 17 and 550 estimators (Figure S5).

To better understand the features focused upon by the RP-PPI model, we plot the relative feature importance, measured by average information gain in Figure S6. Many of the original features from the work of (Dick and Green, 2018) are leveraged in addition to new "statistics-type" features where a given pairs' score is measured in standard deviations away from the identified baseline of a given one-to-all score curve. Notably, baseline scores and ranks for Element A (the SARS-CoV-2 protein) of both methods are among the most distinguishing features (top-4).

On the Interpretation of PIPE-Sites Predictions

When interpreting the landscapes, it is important to note that the PIPE-Sites algorithm used here is simplistic in its implementation. Briefly, a maximum of three potential peaks in the landscape are identified and a walk algorithm expands the predicted site of interaction until the score falls below a given threshold (Amos-Binks et al., 2011).

The highlighted sites may appear "shifted" relative to the highlighted cells (typically in the bottom-left); this is due to the algorithm's use of a window of 20 amino acids in length that extends both to the left (along the x-axis) and upwards (along the y-axis). Consequently, the *minimum PIPE-Site size* is 20×20 with the peak in the bottom-left corner. Additionally, this implementation may result in the predicted site extending past the coloured matrix, either to the right or above. This defined window size additionally prevents predictions within the terminal 20 amino acids of both sequences given that the widow sizes in these regions would necessarily be less than 20 amino acids in length. Finally, the PIPE-Sites may overlap when numerous hits appear within close proximity, as is the case when a "band" of hits appears in the matrix. Finally, when the peak of the landscape comprises only a few hits (generally < 3) the entire landscape is predicted as a site of interaction; evidently, these should be disregarded (Amos-Binks et al., 2011).

Therefore, when interpreting the landscapes, it is important not to solely rely on these proposed regions; they function as an initial guide, yet other high-scoring, or "hot-spot", regions of interest may exist in the landscape. We additionally provide the SW landscapes to compliment the determination of putative regions mediating a given interaction. By providing the matrices of raw scores (in the form of a space-separated .mat file), visual interpretation of the results promise to reveal notable subsequences as well as enable the application of related interaction site predictors to identify putative sites of interaction.

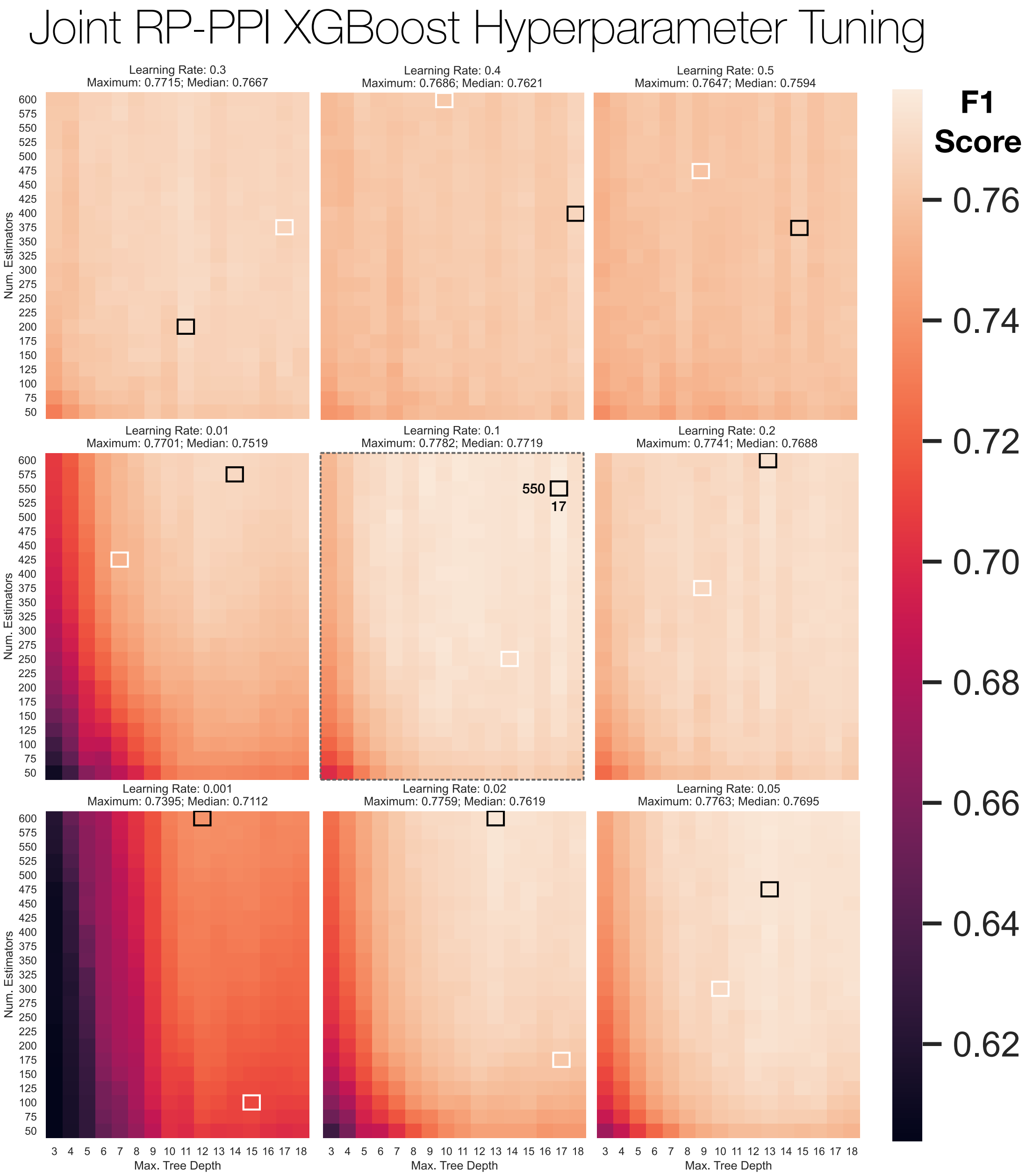

Supplementary Figure S5. Hyperparameter Tuning of the RP-PPI XGBoost Model. Each of the nine subplots depicts the results keeping the learning rate fixed as we vary the maximum tree depth (x-axis) between $[3, 18]$ by increments of 1 and the number of estimators (y-axis) between $[50, 600]$ by increments of 25. Within each subplot, we highlight the maximum value with a black bounding box and the median value with a white bounding box. All results are normalized to the same colour range where lighter values represent better performing models. The best performance is achieved with a learning rate of 0.1, a maximum tree-depth of 17 and 550 estimators.

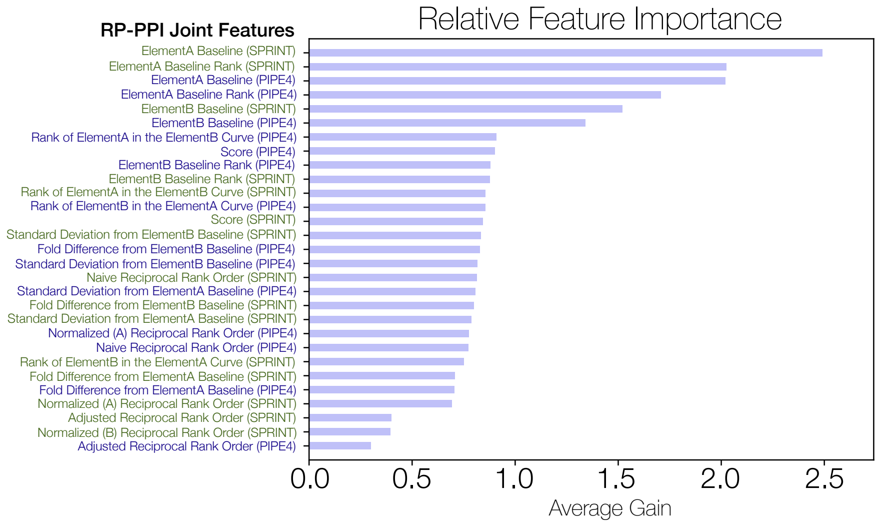

Supplementary Figure S6. Feature Importance of the RP-PPI CME Model. The combined RP features from each model are sorted by relative importance, measured as the average information gain.

CONCLUSIONS

The purpose of this work is to help guide the broader research community in the collective pursuit to understand the SARS-CoV-2 viral pathogenesis. To that end, we assessed 285,124 protein pairs using two state-of-the-art sequence-based PPI predictors within three prediction schemas, thereby creating the comprehensive SARS-CoV-2 vs. human interactome. For each of the 14 SARS-CoV-2 proteins considered in this study, a highly conservative locally defined decision threshold was determined to obtain a predicted interactome comprising putative PPIs within the predicted intersection of the PIPE4 and SPRINT methods. Furthermore, the PIPE-Sites algorithm was used to predict the putative interaction interfaces to identify the subsequence regions of interest that might mediate these interactions.

Beyond a highly applied study focused on countering the COVID19 pandemic, this work introduces for the first time a number of methodological contributions:

1. **RP-Inspired Local Decision Threshold of Model Predictions:** to delineate the rare and high-scoring predicted pairs (most likely to be positives) from the common and low-scoring predicted pairs (most likely to be negative), a single perspective one-to-all score curve is generated and baseline detection applied to identify a local decision threshold.

2. **RP as an Ensembling Method (Combination of Multiple Experts):** the joint use of RP features derived from independent predictors (RP-PIPE4 and RP-SPRINT) demonstrated improved performance suggesting that the RP framework may be an effective ensembling method of independent models.

3. **The Similarity Weighted Landscape:** published in this work for the first time are the SW landscapes that differ from the original PIPE hit landscape in that subsequence frequency is normalized according to the SW score.

These predictions have been deposited in this public DataVerse repository for use by the broader scientific community in this collective effort to combat the COVID-19 pandemic (Dick et al., 2020). All data and metadata are released under a CC-BY 4.0 licence and we re-emphasize that the information provided is theoretical modelling only and caution should be exercised in its use. It is intended only as a resource for the scientific community at large in furthering our understanding of SARS-CoV-2.

REFERENCES

Amos-Binks, A., Patulea, C., Pitre, S., Schoenrock, A., Gui, Y., Green, J. R., Golshani, A., and Dehne, F. (2011). Binding site prediction for protein-protein interactions and novel motif discovery using re-occurring polypeptide sequences. *BMC bioinformatics*, 12(1):225.

Baltimore, D. (1971). Expression of animal virus genomes. *Bacteriological reviews*, 35(3):235.

Baranov, P. V., Henderson, C. M., Anderson, C. B., Gesteland, R. F., Atkins, J. F., and Howard, M. T. (2005). Programmed ribosomal frameshifting in decoding the sars-cov genome. *Virology*, 332(2):498–510.

Binn, L., Lazar, E., Keenan, K., Huxsoll, D., Marchwicki, R., and Strano, A. (1974). Recovery and characterization of a coronavirus from military dogs with diarrhea. In *Proceedings,... annual meeting of the United States Animal Health Association*.

Bridger, J. C., Caul, E., and Egglestone, S. (1978). Replication of an enteric bovine coronavirus in intestinal organ cultures. *Archives of virology*, 57(1):43–51.

Brierley, I., Digard, P., and Inglis, S. C. (1989). Characterization of an efficient coronavirus ribosomal frameshifting signal: requirement for an rna pseudoknot. *Cell*, 57(4):537–547.

Chan, C.-M., Chu, H., Wang, Y., Wong, B. H.-Y., Zhao, X., Zhou, J., Yang, D., Leung, S. P., Chan, J. F.-W., Yeung, M.-L., Yan, J., Lu, G., Gao, G. F., and Yuen, K.-Y. (2016). Carcinoembryonic antigen-related cell adhesion molecule 5 is an important surface attachment factor that facilitates entry of middle east respiratory syndrome coronavirus. *Journal of Virology*, 90(20):9114–9127.

Chang, C.-k., Sue, S.-C., Yu, T.-h., Hsieh, C.-M., Tsai, C.-K., Chiang, Y.-C., Lee, S.-j., Hsiao, H.-h., Wu, W.-J., Chang, W.-L., Lin, C.-H., and Huang, T.-h. (2006). Modular organization of sars coronavirus nucleocapsid protein. *Journal of biomedical science*, 13(1):59–72.

Chen, Y., Tao, J., Sun, Y., Wu, A., Su, C., Gao, G., Cai, H., Qiu, S., Wu, Y., Ahola, T., and Guo, D. (2013). Structure-function analysis of severe acute respiratory syndrome coronavirus rna cap guanine-n7-methyltransferase. *Journal of Virology*, 87(11):6296–6305.

Cui, J., Li, F., and Shi, Z.-L. (2019). Origin and evolution of pathogenic coronaviruses. *Nature Reviews Microbiology*, 17(3):181–192.

Dick, K., Biggar, K. K., and Green, J. R. (2020). Comprehensive Prediction of the SARS-CoV-2 vs. Human Interactome using PIPE4, SPRINT, and PIPE-Sites.

Dick, K. and Green, J. (2016). Comparison of sequence-and structure-based protein-protein interaction sites. In *2016 IEEE EMBS International Student Conference (ISC)*, pages 1–4. IEEE.

Dick, K. and Green, J. R. (2018). Reciprocal perspective for improved protein-protein interaction prediction. *Scientific reports*, 8(1):1–12.

Erles, K., Toomey, C., Brooks, H. W., and Brownlie, J. (2003). Detection of a group 2 coronavirus in dogs with canine infectious respiratory disease. *Virology*, 310(2):216–223.

Guarner, J. (2020). Three Emerging Coronaviruses in Two Decades: The Story of SARS, MERS, and Now COVID-19. *American Journal of Clinical Pathology*, 153(4):420–421.

Hamre, D. and Procknow, J. J. (1966). A new virus isolated from the human respiratory tract. *Proceedings of the Society for Experimental Biology and Medicine*, 121(1):190–193.

Heald-Sargent, T. and Gallagher, T. (2012). Ready, set, fuse! the coronavirus spike protein and acquisition of fusion competence. *Viruses*, 4(4):557–580.

Jumper, J., Tunyasuvunakool, K., Kohli, P., and Hassabis, D. (2020). Computational predictions of protein structures associated with covid-19.

Kazmirchuk, T., Dick, K., Burnside, D. J., Barnes, B., Moteshareie, H., Hajikarimlou, M., Omidi, K., Ahmed, D., Low, A., Lettl, C., Hooshyar, M., Schoenrock, A., Pitre, S., Babu, M., Cassol, E., Samanfar, B., Wong, A., Dehne, F., Green, J. R., and Golshani, A. (2017). Designing anti-zika virus peptides derived from predicted human-zika virus protein-protein interactions. *Computational Biology and Chemistry*, 71:180–187.

Klausegger, A., Strobl, B., Regl, G., Kaser, A., Luytjes, W., and Vlasak, R. (1999). Identification of a coronavirus hemagglutinin-esterase with a substrate specificity different from those of influenza c virus and bovine coronavirus. *Journal of virology*, 73(5):3737–3743.

Kotlyar, M., Rossos, A. E., and Jurisica, I. (2017). Prediction of protein-protein interactions. *Current Protocols in Bioinformatics*, 60(1):8–2.

Krempl, C., Schultze, B., Laude, H., and Herrler, G. (1997). Point mutations in the s protein connect the sialic acid binding activity with the enteropathogenicity of transmissible gastroenteritis coronavirus. *Journal of virology*, 71(4):3285–3287.

Kubo, H., Yamada, Y. K., and Taguchi, F. (1994). Localization of neutralizing epitopes and the receptor-binding site within the amino-terminal 330 amino acids of the murine coronavirus spike protein. *Journal of Virology*, 68(9):5403–5410.

Kyrollos, D. G., Reid, B., Dick, K., and Green, J. R. (2020). Rpmirdip: Reciprocal perspective improves mirna targeting prediction. *Scientific reports*, 10(1):1–13.

Lai, M. M. and Cavanagh, D. (1997). The molecular biology of coronaviruses. In *Advances in virus research*, volume 48, pages 1–100. Elsevier.

Lang, J., Yang, N., Deng, J., Liu, K., Yang, P., Zhang, G., and Jiang, C. (2011). Inhibition of sars pseudovirus cell entry by lactoferrin binding to heparan sulfate proteoglycans. *PloS one*, 6(8):e23710.

Lau, S. K. P., Lee, P., Tsang, A. K. L., Yip, C. C. Y., Tse, H., Lee, R. A., So, L.-Y., Lau, Y.-L., Chan, K.-H., Woo, P. C. Y., and Yuen, K.-Y. (2011). Molecular epidemiology of human coronavirus oc43 reveals evolution of different genotypes over time and recent emergence of a novel genotype due to natural recombination. *Journal of Virology*, 85(21):11325–11337.

Makino, S., Keck, J. G., Stohlman, S. A., and Lai, M. (1986). High-frequency rna recombination of murine coronaviruses. *Journal of Virology*, 57(3):729–737.

McIntosh, K., Dees, J. H., Becker, W. B., Kapikian, A. Z., and Chanock, R. M. (1967). Recovery in tracheal organ cultures of novel viruses from patients with respiratory disease. *Proceedings of the National Academy of Sciences of the United States of America*, 57(4):933.

Milewska, A., Zarebski, M., Nowak, P., Stozek, K., Potempa, J., and Pyrc, K. (2014). Human coronavirus nl63 utilizes heparan sulfate proteoglycans for attachment to target cells. *Journal of virology*, 88(22):13221–13230.

Monto, A. S. (1974). Medical reviews. coronaviruses. *The Yale journal of biology and medicine*, 47(4):234.

Neuman, B. W., Kiss, G., Kunding, A. H., Bhella, D., Baksh, M. F., Connelly, S., Droese, B., Klaus, J. P., Makino, S., Sawicki, S. G., Siddell, S. G., Stamou, D. G., Wilson, I. A., Kuhn, P., and Buchmeier, M. J. (2011). A structural analysis of m protein in coronavirus assembly and morphology. *Journal of Structural Biology*, 174(1):11–22.

Payne, S. (2017). Family coronaviridae. *Viruses*, page 149.

Pedersen, N., Evermann, J., McKeirnan, A., and Ott, R. (1984). Pathogenicity studies of feline coronavirus isolates 79-1146 and 79-1683. *American journal of veterinary research*, 45(12):2580–2585.

Pfefferle, S., Schöpf, J., Kögl, M., Friedel, C. C., Müller, M. A., Carbajo-Lozoya, J., Stellberger, T., von Dall'Armi, E., Herzog, P., Kallies, S., Niemeyer, D., Ditt, V., Kuri, T., Züst, R., Pumpor, K., Hilgenfeld, R., Schwarz, F., Zimmer, R., Steffen, I., Weber, F., Thiel, V., Herrler, G., Thiel, H.-J., Schwegmann-Weßels, C., Pöhlmann, S., Haas, J., Drosten, C., and von Brunn, A. (2011). The sars-coronavirus-host interactome: Identification of cyclophilins as target for pan-coronavirus inhibitors. *PLOS Pathogens*, 7(10):1–15.

Regional Office for the Eastern Mediterranean (2011). Mers situation update.

Schoeman, D. and Fielding, B. C. (2019). Coronavirus envelope protein: current knowledge. *Virology journal*, 16(1):1–22.

Senior, A. W., Evans, R., Jumper, J., Kirkpatrick, J., Sifre, L., Green, T., Qin, C., Žídek, A., Nelson, A. W., Bridgland, A., Penedones, H., Petersen, S., Simonyan, K., Crossan, S., Kohli, P., Jones, D. T., Silver, D., Kavukcuoglu, K., and Hassabis, D. (2020). Improved protein structure prediction using potentials from deep learning. *Nature*, pages 1–5.

Smith, M. and Smith, J. C. (2020). Repurposing therapeutics for covid-19: Supercomputer-based docking to the sars-cov-2 viral spike protein and viral spike protein-human ace2 interface. *ChemRxiv*.

Van Der Most, R. G., Heijnen, L., Spaan, W. J., and De Groot, R. J. (1992). Homologous rna recombination allows efficient introduction of site-specific mutations into the genome of coronavirus mhv-a59 via synthetic co-replicating rnas. *Nucleic acids research*, 20(13):3375–3381.

Weiner, L. P. (1973). Pathogenesis of demyelination induced by a mouse hepatitis. *Archives of Neurology*, 28(5):298–303.

Woo, P. C., Lau, S. K., Yip, C. C., Huang, Y., Tsoi, H.-W., Chan, K.-H., and Yuen, K.-Y. (2006). Comparative analysis of 22 coronavirus hku1 genomes reveals a novel genotype and evidence of natural recombination in coronavirus hku1. *Journal of virology*, 80(14):7136–7145.

World Health Organization (2020). *Laboratory Biosafety Manual, 3rd edition.*

Ziebuhr, J., Snijder, E. J., and Gorbalenya, A. E. (2000). Virus-encoded proteinases and proteolytic processing in the nidovirales. *Journal of General Virology*, 81(4):853–879.

APPENDIX

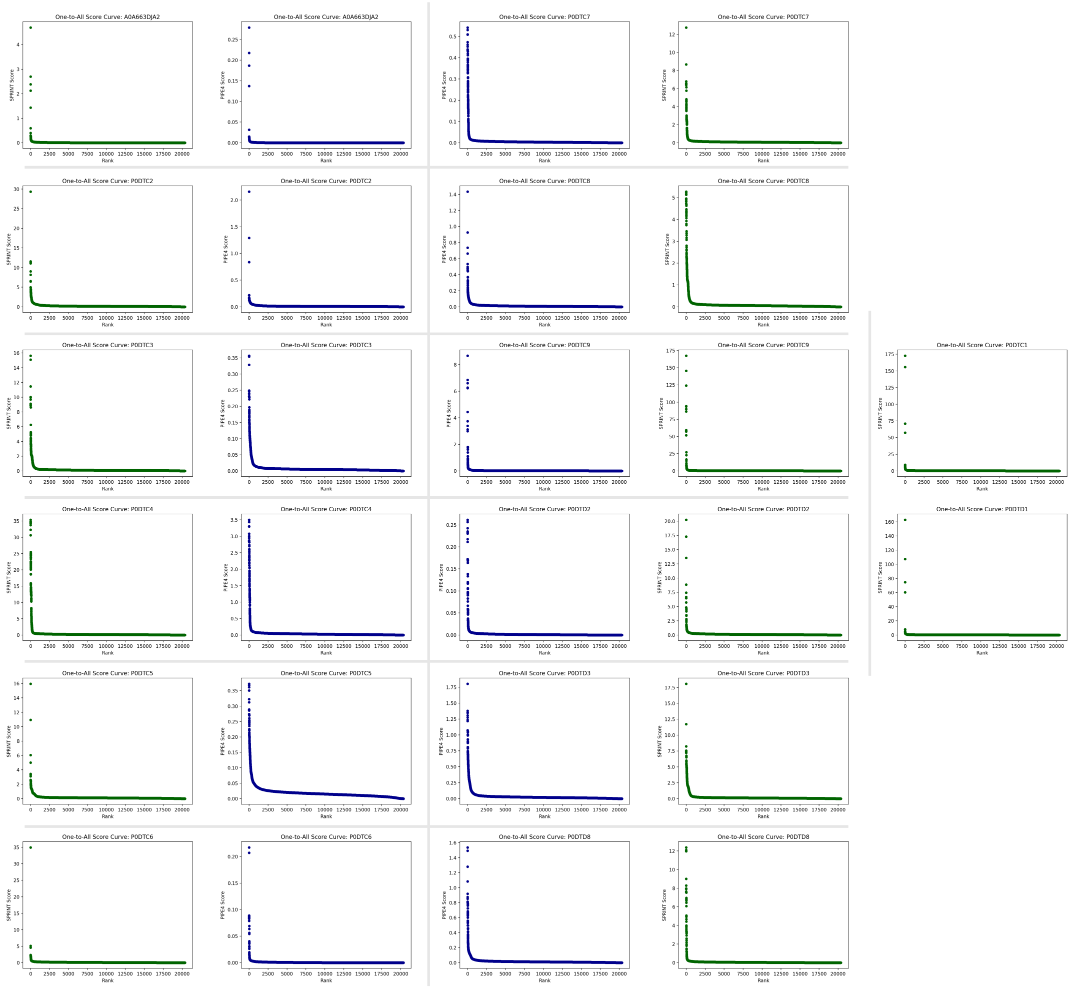

Supplementary Figure S7. Compilation of the One-to-All Score Curves for each SARS-CoV-2 protein by PIPE4 (blue) and SPRINT (green) in the *All* Schema. Each of the subplots depicts a characteristic "L"-shape, where there are a relatively small number of high-scoring pairs as compared to a large number of low-scoring pairs within the baseline. Note that the y-axes are not shared among subplots.

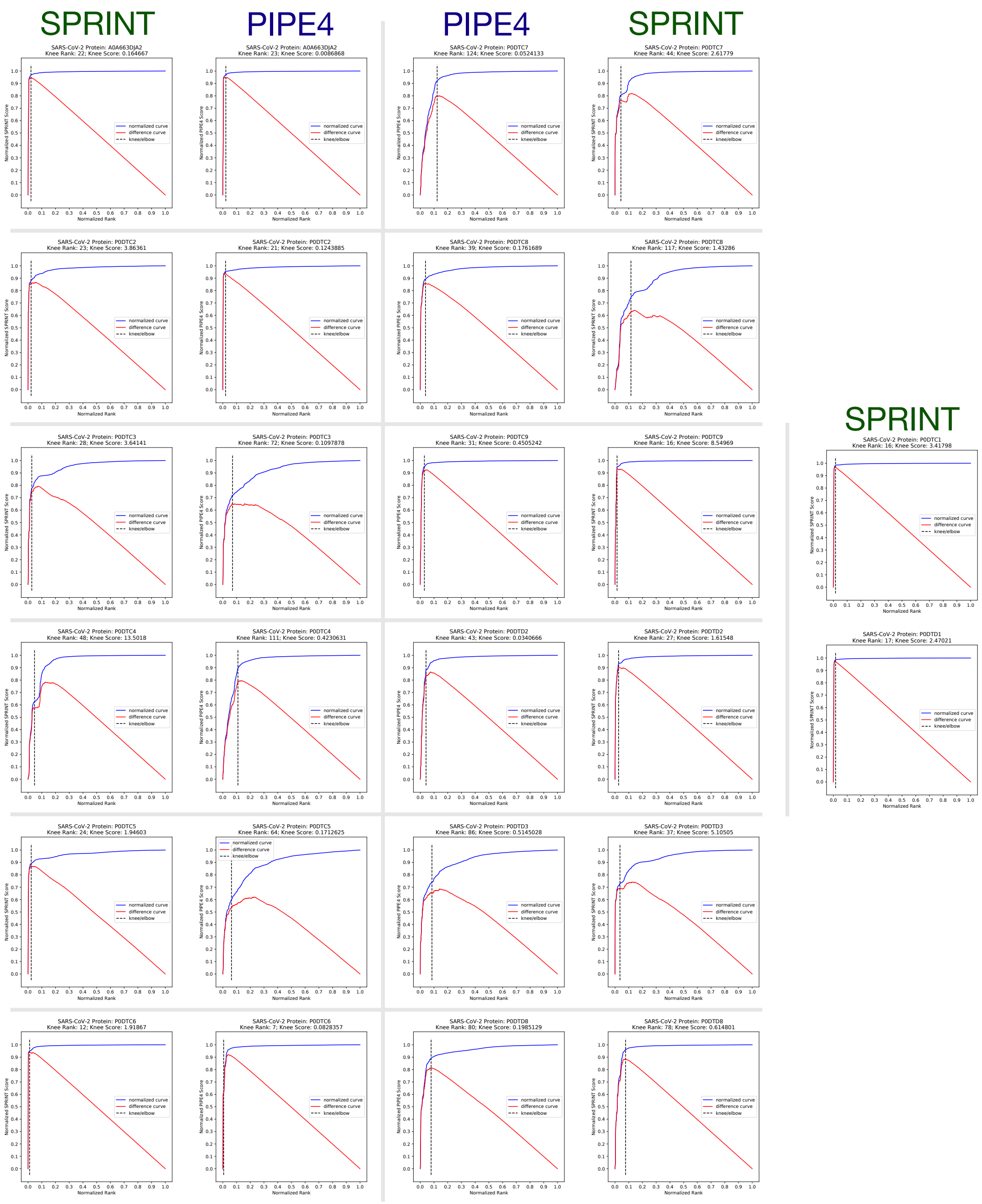

Supplementary Figure S8. Compilation of the Detected Knee of each One-to-All Score Curves for each SARS-CoV-2 protein by PIPE4 and SPRINT in the *All* Schema. Each of the subplots highlights the detected knee of the normalized top-1000 predictions obtained using the Kneedle algorithm. The *differences* curve plots the value obtained from subtracting the perpendicular distance of each point to $y = x$ from the distance of each point vertically to $y = x$ of the normalized plot. The peak of this curve, parameterized by S, estimates the location of the knee.

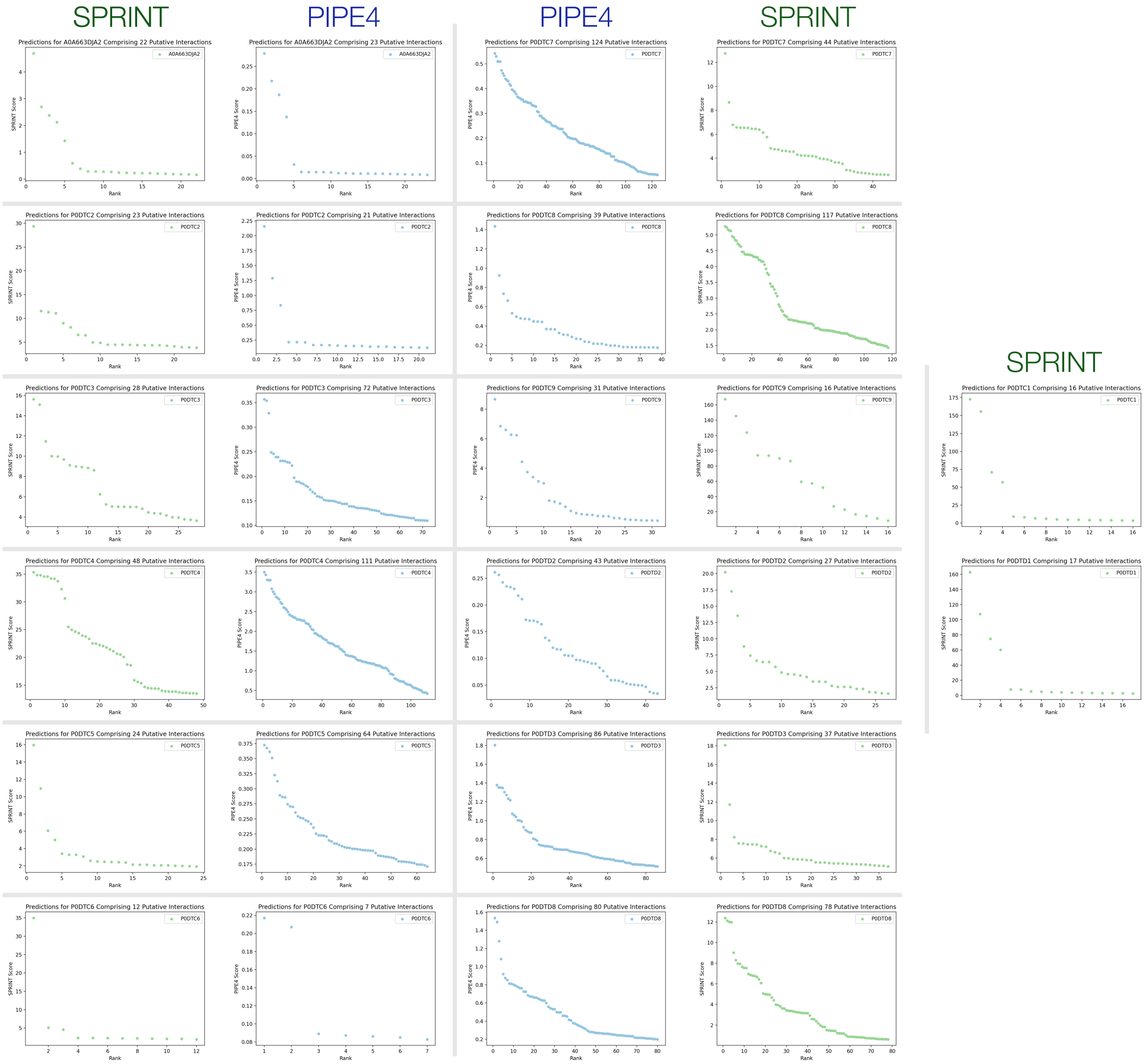

Supplementary Figure S9. **Compiled plot of all the Predicted Interactions for each Protein and each Method in the *All* Schema.**

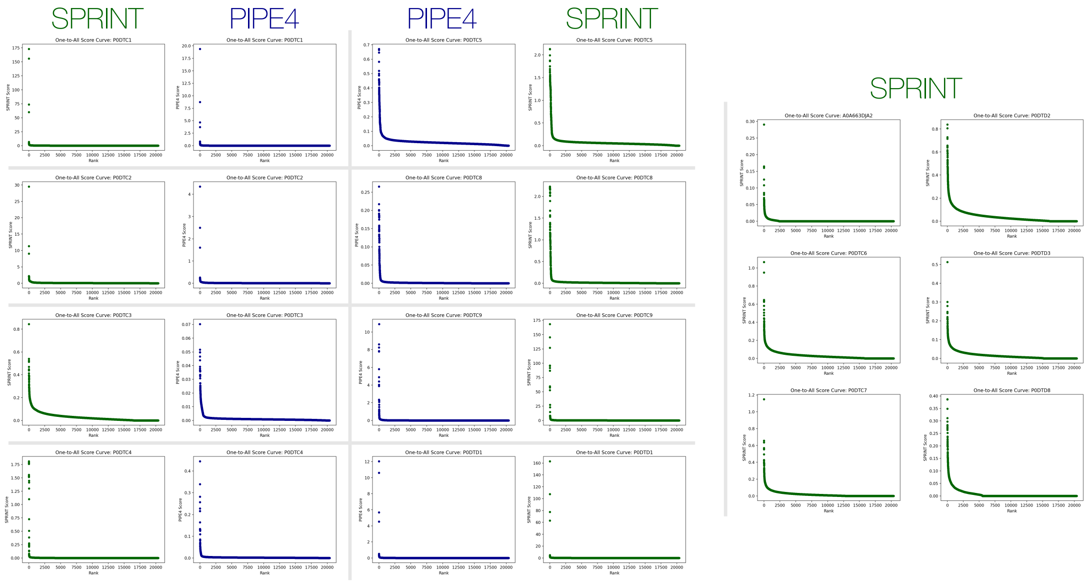

Supplementary Figure S10. Compilation of the One-to-All Score Curves for each SARS-CoV-2 protein by PIPE4 (blue) and SPRINT (green) in the *Proximal* Schema. Each of the subplots depicts a characteristic "L"-shape, where there are a relatively small number of high-scoring pairs as compared to a large number of low-scoring pairs within the baseline. Note that the y-axes are not shared among subplots.

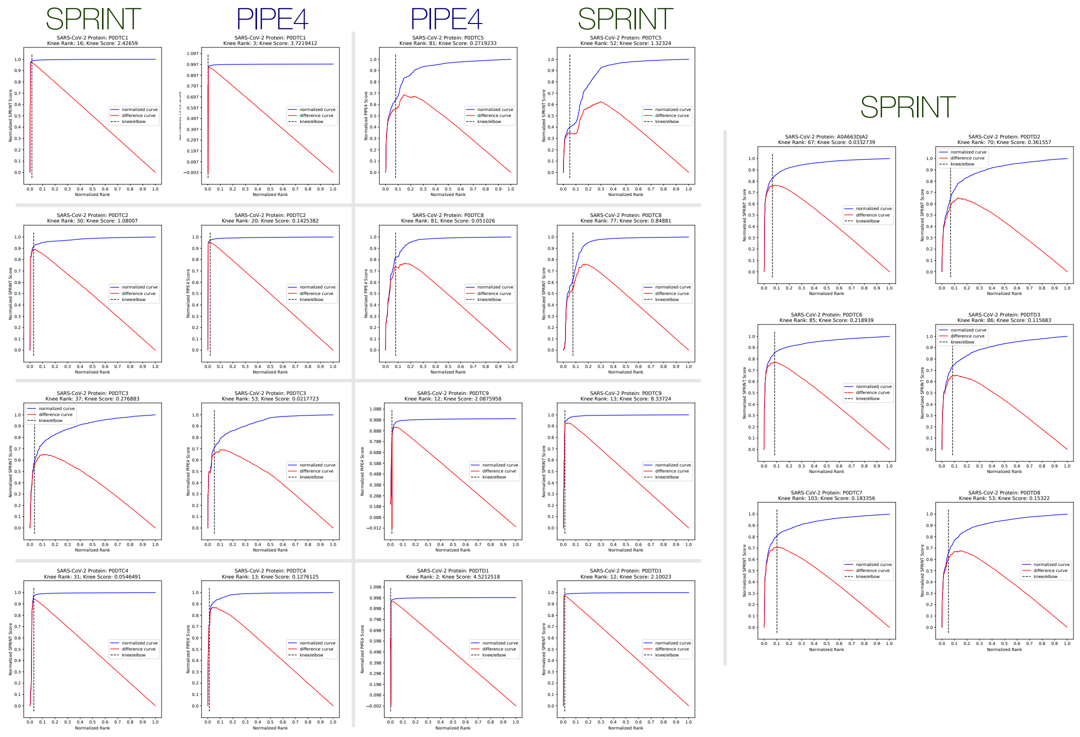

Supplementary Figure S11. Compilation of the Detected Knee of each One-to-All Score Curves for each SARS-CoV-2 protein by PIPE4 and SPRINT in the *Proximal* Schema. Each of the subplots highlights the detected knee of the normalized top-1000 predictions obtained using the Kneedle algorithm. The *differences* curve plots the value obtained from subtracting the perpendicular distance of each point to $y = x$ from the distance of each point vertically to $y = x$ of the normalized plot. The peak of this curve, parameterized by S, estimates the location of the knee.

Supplementary Table S2. Proteomes of the Majority of Organisms Considered in the *All* Schema.

Organism	Taxonomy Id	Proteome Acc.
Rotavirus A	9913	UP000106064
Sindbis virus (SINV)	11034	UP000006710
Rubella virus (strain M33) (RUBV)	11043	UP000007143
Dengue virus 1	11053	UP000101782
Dengue virus 2	11060	UP000096836
Dengue virus type 2 (strain Thailand/NGS-C/1944) (DENV-2)	11065	UP000007196
Japanese encephalitis virus	11072	UP000121923
Kunjin virus	11077	UP000100779
Kunjin virus (strain MRM61C)	11078	UP000099558
West Nile virus (WNV)	11082	UP000102709
Tick-borne encephalitis virus	11084	UP000140821
Classical swine fever virus	11096	UP000106488
Bovine viral diarrhea virus (BVDV) (Mucosal disease virus)	11099	UP000155116
Hepatitis C virus genotype 1a (isolate H) (HCV)	11103	UP000000518
Hepatitis C virus genotype 1a (isolate 1) (HCV)	11104	UP000008855
Hepatitis C virus genotype 1b (isolate BK) (HCV)	11105	UP000007413
Hepatitis C virus genotype 1a (isolate H) (HCV)	11108	UP000000518
Hepatitis C virus genotype 2a (isolate HC-J6) (HCV)	11113	UP000002682
Hepatitis C virus genotype 1b (isolate Japanese) (HCV)	11116	UP000008095
Human coronavirus 229E (HCoV-229E)	11137	UP000006716
Hepatitis E virus (HEV)	12461	UP000106507
Porcine reproductive and respiratory syndrome virus (PRRSV)	28344	UP000146080
Dengue virus type 2 (strain Thailand/16681/1984) (DENV-2)	31634	UP000180751
Dengue virus type 2 (strain 16681-PDK53) (DENV-2)	31635	UP000008390
Hepatitis C virus genotype 1b (isolate Taiwan) (HCV)	31645	UP000002679
Hepatitis E virus genotype 1 (isolate Human/Burma) (HEV-1)	31767	UP000007243
Hepatitis E virus genotype 2 (isolate Human/Mexico) (HEV-2)	31768	UP000007245
Bovine viral diarrhea virus 2	54315	UP000129869
Alkhumra hemorrhagic fever virus (ALKV)	172148	UP000097483
Human SARS coronavirus (SARS-CoV)	227859	UP000000354
Porcine epidemic diarrhea virus (strain CV777) (PEDV)	229032	UP000008159
SARS coronavirus Frankfurt 1	229992	UP000113286
Porcine torovirus	237020	UP000269215
Human coronavirus NL63 (HCoV-NL63)	277944	UP000103541
Hepatitis C virus genotype 1b (isolate Con1) (HCV)	333284	UP000007414
Hepatitis C virus genotype 2a (isolate JFH-1) (HCV)	356411	UP000008096
Breda virus 1 (BRV-1)	360393	UP000000355
Dengue virus type 4 (strain Dominica/814669/1981) (DENV-4)	408871	UP000108177
Hepatitis C virus genotype 1b (strain HC-J4) (HCV)	420174	UP000008094
Hepatitis C virus genotype 1b (isolate HC-J1) (HCV)	421877	UP000008093
Hepatitis C virus genotype 1b (isolate HCR6) (HCV)	421879	UP000008100
Hepatitis E virus genotype 4 (isolate Human/China/T1) (HEV-4)	509627	UP000007242
Hepatitis E virus genotype 1 (isolate Human/India/Hyderabad)	512346	UP000007244

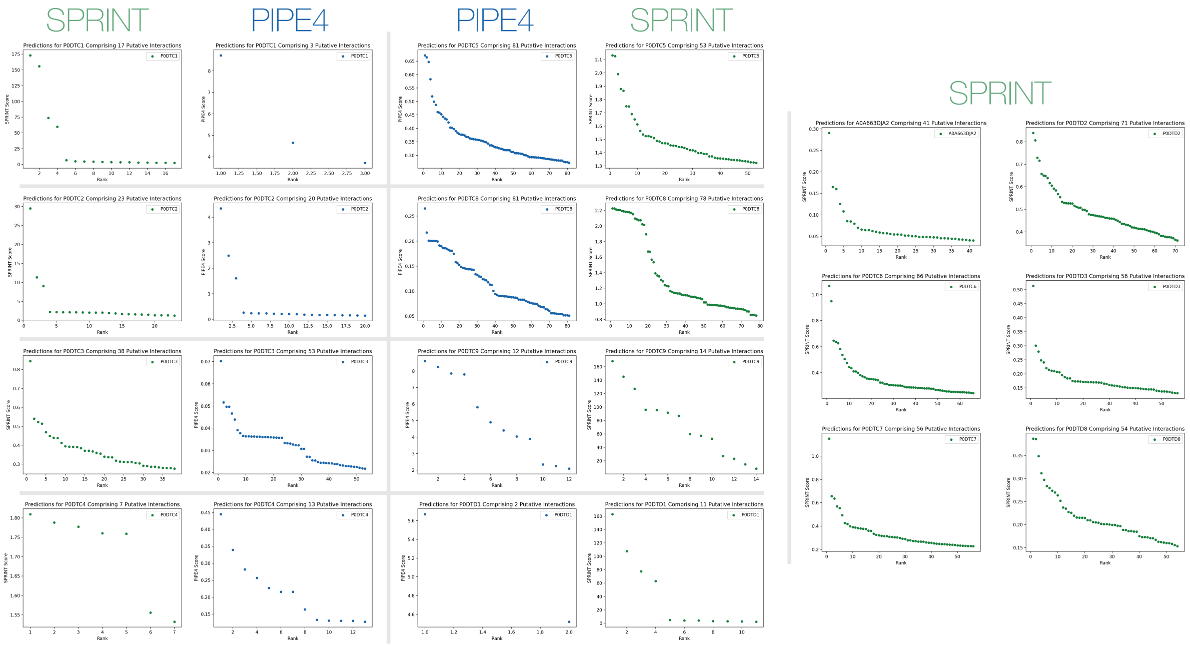

Supplementary Figure S12. Compiled plot of all the Predicted Interactions for each Protein and each Method in the *Prox* Schema.

Supplementary Table S3. PANTHER GO-Term Analysis of Molecular Function Over/Under-Representation for the 225 Predicted Human Interactors in the *All* Schema.

PANTHER GO Molecular Function	Homo sapiens Reference (N=20,851)	Num. Predicted	Predicted Num. Expected	Over/Under Represented	Fold Enrichment	*p*-value	FDR
peptidase activator activity (GO:0016504)	6	4	0.06	+	64.65	2.11E-06	4.88E-05
tumor necrosis factor receptor superfamily binding (GO:0032813)	9	5	0.09	+	53.88	1.96E-07	6.53E-06
TBP-class protein binding (GO:0017025)	9	3	0.09	+	32.33	2.15E-04	3.28E-03
ubiquitin-like protein ligase binding (GO:0044389)	46	14	0.47	+	29.52	9.96E-16	1.77E-13
protein tyrosine kinase activity (GO:0004713)	61	17	0.63	+	27.03	2.71E-18	7.20E-16
ubiquitin protein ligase binding (GO:0031625)	41	9	0.42	+	21.29	1.77E-09	1.05E-07
signal sequence binding (GO:0005048)	33	7	0.34	+	20.57	1.47E-07	6.00E-06
heat shock protein binding (GO:0031072)	30	5	0.31	+	16.16	2.67E-05	4.91E-04
ATP binding (GO:0005524)	40	5	0.41	+	12.12	9.26E-05	1.54E-03
unfolded protein binding (GO:0051082)	58	6	0.6	+	10.03	4.83E-05	8.28E-04
endopeptidase activity (GO:0004175)	307	24	3.17	+	7.58	6.01E-14	5.33E-12
ATPase activity, coupled (GO:0042623)	117	8	1.21	+	6.63	4.37E-05	7.75E-04
peptidase activity (GO:0008233)	415	28	4.28	+	6.54	1.35E-14	1.43E-12
peptidase activity, acting on L-amino acid peptides (GO:0070011)	407	27	4.2	+	6.43	6.06E-14	4.61E-12
ubiquitin-protein transferase activity (GO:0004842)	239	14	2.46	+	5.68	3.57E-07	1.00E-05
peptide binding (GO:0042277)	194	11	2	+	5.5	8.82E-06	1.80E-04
ubiquitin-like protein transferase activity (GO:0019787)	249	14	2.57	+	5.45	5.70E-07	1.52E-05
ubiquitin protein ligase activity (GO:0061630)	145	8	1.5	+	5.35	1.80E-04	2.91E-03
cytokine receptor binding (GO:0005126)	93	5	0.96	+	5.21	3.33E-03	4.43E-02
ubiquitin-like protein ligase activity (GO:0061659)	149	8	1.54	+	5.21	2.15E-04	3.36E-03
amide binding (GO:0033218)	211	11	2.18	+	5.06	1.86E-05	3.67E-04
catalytic activity, acting on a protein (GO:0140096)	1400	65	14.44	+	4.5	5.29E-25	2.81E-22
ATPase activity (GO:0016887)	262	12	2.7	+	4.44	2.65E-05	5.04E-04
phosphotransferase activity, alcohol group as acceptor (GO:0016773)	519	21	5.35	+	3.92	1.77E-07	6.28E-06
protein kinase activity (GO:0004672)	435	17	4.49	+	3.79	4.31E-06	9.56E-05
enzyme binding (GO:0019899)	610	23	6.29	+	3.66	1.50E-07	5.71E-06
kinase activity (GO:0016301)	559	21	5.76	+	3.64	5.71E-07	1.45E-05
signaling receptor binding (GO:0005102)	629	23	6.49	+	3.55	2.53E-07	7.49E-06
transferase activity, transferring phosphorus-containing groups (GO:0016772)	665	21	6.86	+	3.06	7.94E-06	1.69E-04

Supplementary Table S4. PANTHER GO-Term Analysis of Biological Process Over/Under-Representation for the 225 Predicted Human Interactors in the *All* Schema.

PANTHER GO Biological Process	Homo sapiens Reference (N=20,851)	Num. Predicted	Predicted Num. Expected	Over/Under Represented	Fold Enrichment	p-value	FDR
antigen processing and presentation of exogenous peptide antigen via MHC class Ib (GO:0002477)	2	2	0.02	+	96.98	6.14E-04	1.53E-02
nerve growth factor production (GO:0032902)	2	2	0.02	+	96.98	6.14E-04	1.53E-02
neurotrophin production (GO:0032898)	2	2	0.02	+	96.98	6.14E-04	1.52E-02
positive regulation of endoplasmic reticulum calcium ion concentration (GO:0032470)	2	2	0.02	+	96.98	6.14E-04	1.52E-02
entry of viral genome into host nucleus through nuclear pore complex via importin (GO:0075506)	2	2	0.02	+	96.98	6.14E-04	1.52E-02
positive regulation of telomerase RNA reverse transcriptase activity (GO:1905663)	2	2	0.02	+	96.98	6.14E-04	1.52E-02
positive regulation of fast-twitch skeletal muscle fiber contraction (GO:0031448)	2	2	0.02	+	96.98	6.14E-04	1.51E-02
regulation of fast-twitch skeletal muscle fiber contraction (GO:0031446)	2	2	0.02	+	96.98	6.14E-04	1.51E-02
calcium ion transport from cytosol to endoplasmic reticulum (GO:1903515)	2	2	0.02	+	96.98	6.14E-04	1.51E-02
multi-organism nuclear import (GO:1902594)	4	3	0.04	+	72.74	3.56E-05	1.18E-03
viral penetration into host nucleus (GO:0075732)	4	3	0.04	+	72.74	3.56E-05	1.18E-03
nerve growth factor processing (GO:0032455)	4	3	0.04	+	72.74	3.56E-05	1.18E-03
adenine transport (GO:0015853)	4	3	0.04	+	72.74	3.56E-05	1.18E-03
proteasomal ubiquitin-independent protein catabolic process (GO:0010499)	23	16	0.24	+	67.47	2.42E-22	3.01E-20
histamine secretion by mast cell (GO:0002553)	3	2	0.03	+	64.65	1.02E-03	2.33E-02
histamine secretion involved in inflammatory response (GO:0002441)	3	2	0.03	+	64.65	1.02E-03	2.33E-02
positive regulation of caveolin-mediated endocytosis (GO:2001288)	3	2	0.03	+	64.65	1.02E-03	2.33E-02
histamine production involved in inflammatory response (GO:0002349)	3	2	0.03	+	64.65	1.02E-03	2.32E-02
regulation of telomerase RNA reverse transcriptase activity (GO:1905661)	3	2	0.03	+	64.65	1.02E-03	2.32E-02
positive regulation of translation in response to endoplasmic reticulum stress (GO:0036493)	3	2	0.03	+	64.65	1.02E-03	2.32E-02
calcium ion import into sarcoplasmic reticulum (GO:1990036)	3	2	0.03	+	64.65	1.02E-03	2.31E-02
positive regulation of ATPase-coupled calcium transmembrane transporter activity (GO:1901896)	5	3	0.05	+	58.19	5.65E-05	1.80E-03

Supplementary Table S5. PANTHER GO-Term Analysis of Cellular Component Over/Under-Representation for the 225 Predicted Human Interactors in the *All* Schema..

PANTHER GO Cellular Component	Homo sapiens Reference (N=20,851)	Num. Predicted	Predicted Num. Expected	Over/Under Represented	Fold Enrichment	*p*-value	FDR
MHC class Ib protein complex (GO:0032398)	2	2	0.02	+	96.98	6.14E-04	1.13E-02
proteasome core complex, alpha-subunit complex (GO:0019773)	8	8	0.08	+	96.98	1.24E-12	8.33E-11
proteasome activator complex (GO:0008537)	3	3	0.03	+	96.98	2.05E-05	5.04E-04
spermatoproteasome complex (GO:1990111)	5	4	0.05	+	77.59	1.28E-06	4.02E-05
phosphopyruvate hydratase complex (GO:0000015)	4	3	0.04	+	72.74	3.56E-05	8.64E-04
proteasome core complex (GO:0005839)	21	15	0.22	+	69.27	3.82E-21	5.50E-19
proteasome core complex, beta-subunit complex (GO:0019774)	11	7	0.11	+	61.72	3.03E-10	1.60E-08
proteasome regulatory particle, base subcomplex (GO:0008540)	12	7	0.12	+	56.57	4.75E-10	2.33E-08
MHC class I protein complex (GO:0042612)	9	5	0.09	+	53.88	1.96E-07	6.82E-06
eukaryotic translation elongation factor 1 complex (GO:0005853)	4	2	0.04	+	48.49	1.51E-03	2.44E-02
cytosolic proteasome complex (GO:0031597)	9	4	0.09	+	43.1	7.02E-06	1.94E-04
protein phosphatase type 1 complex (GO:0000164)	9	4	0.09	+	43.1	7.02E-06	1.91E-04
PTW/PP1 phosphatase complex (GO:0072357)	7	3	0.07	+	41.56	1.19E-04	2.67E-03
proteasome complex (GO:0000502)	65	26	0.67	+	38.79	8.25E-31	3.32E-28
proteasome accessory complex (GO:0022624)	25	10	0.26	+	38.79	1.46E-12	9.49E-11
endopeptidase complex (GO:1905369)	66	26	0.68	+	38.2	1.14E-30	3.83E-28
CD40 receptor complex (GO:0035631)	11	4	0.11	+	35.27	1.32E-05	3.36E-04
platelet dense tubular network membrane (GO:0031095)	9	3	0.09	+	32.33	2.15E-04	4.62E-03
cytoplasmic side of lysosomal membrane (GO:0098574)	6	2	0.06	+	32.33	2.79E-03	4.01E-02
proteasome regulatory particle (GO:0005838)	22	7	0.23	+	30.86	1.35E-08	5.91E-07
peptidase complex (GO:1905368)	91	26	0.94	+	27.71	1.18E-27	2.64E-25
glycogen granule (GO:0042587)	7	2	0.07	+	27.71	3.56E-03	4.98E-02
platelet dense tubular network (GO:0031094)	11	3	0.11	+	26.45	3.51E-04	7.01E-03
pseudopodium (GO:0031143)	17	4	0.18	+	22.82	5.51E-05	1.31E-03
postsynaptic specialization, intracellular component (GO:0099091)	22	5	0.23	+	22.04	7.11E-06	1.91E-04
integral component of lumenal side of endoplasmic reticulum membrane (GO:0071556)	29	6	0.3	+	20.07	1.34E-06	4.14E-05
lumenal side of endoplasmic reticulum membrane (GO:0098553)	29	6	0.3	+	20.07	1.34E-06	4.08E-05
MHC protein complex (GO:0042611)	28	5	0.29	+	17.32	1.99E-05	4.94E-04
COP9 signalosome (GO:0008180)	36	6	0.37	+	16.16	4.07E-06	1.21E-04
lumenal side of membrane (GO:0098576)	36	6	0.37	+	16.16	4.07E-06	1.19E-04
extrinsic component of cytoplasmic side of plasma membrane (GO:0031234)	93	15	0.96	+	15.64	3.15E-13	2.19E-11

Supplementary Table S6. PANTHER GO-Term Analysis of Molecular Function Over/Under-Representation for the 123 Predicted Human Interactors in the *Proximal* Schema.

PANTHER GO Molecular Function	Homo sapiens Reference (N=20,851)	Num. Predicted	Predicted Num. Expected	Over/Under Represented	Fold Enrichment	*p*-value	FDR
heat shock protein binding (GO:0031072)	30	6	.18	+	33.90	6.07E-08	2.02E-06
ubiquitin-like protein ligase binding (GO:0044389)	46	9	.27	+	33.17	3.09E-11	1.37E-09
ATP binding (GO:0005524)	40	6	.24	+	25.43	2.78E-07	8.22E-06
unfolded protein binding (GO:0051082)	58	8	.34	+	23.38	4.80E-09	1.83E-07
protein tyrosine kinase activity (GO:0004713)	61	8	.36	+	22.23	6.89E-09	2.44E-07
purine ribonucleoside triphosphate binding (GO:0035639)	154	20	.91	+	22.02	1.89E-20	1.00E-17
GTP binding (GO:0005525)	114	14	.67	+	20.82	2.64E-14	1.41E-12
purine ribonucleotide binding (GO:0032555)	180	20	1.06	+	18.84	3.15E-19	8.39E-17
ribonucleotide binding (GO:0032553)	186	20	1.10	+	18.23	5.70E-19	1.01E-16
purine nucleotide binding (GO:0017076)	192	20	1.13	+	17.66	1.01E-18	1.35E-16
ubiquitin protein ligase binding (GO:0031625)	41	4	.24	+	16.54	1.39E-04	2.96E-03
nucleoside phosphate binding (GO:1901265)	236	20	1.39	+	14.37	4.21E-17	4.48E-15
nucleotide binding (GO:0000166)	236	20	1.39	+	14.37	4.21E-17	3.73E-15
carbohydrate derivative binding (GO:0097367)	255	20	1.50	+	13.30	1.70E-16	1.29E-14
structural molecule activity (GO:0005198)	215	14	1.27	+	11.04	7.75E-11	3.17E-09
small molecule binding (GO:0036094)	377	21	2.22	+	9.44	1.73E-14	1.15E-12
ATPase activity, coupled (GO:0042623)	117	6	.69	+	8.69	8.74E-05	2.11E-03
drug binding (GO:0008144)	121	6	.71	+	8.41	1.04E-04	2.31E-03
anion binding (GO:0043168)	484	23	2.86	+	8.06	2.14E-14	1.27E-12
ion binding (GO:0043167)	717	25	4.23	+	5.91	1.10E-12	5.33E-11
ATPase activity (GO:0016887)	262	9	1.55	+	5.82	3.20E-05	8.12E-04
phosphotransferase activity, alcohol group as acceptor (GO:0016773)	519	10	3.06	+	3.27	1.13E-03	2.30E-02
kinase activity (GO:0016301)	559	10	3.30	+	3.03	1.93E-03	3.54E-02
organic cyclic compound binding (GO:0097159)	1677	27	9.89	+	2.73	1.57E-06	4.41E-05
heterocyclic compound binding (GO:1901363)	1646	26	9.71	+	2.68	3.62E-06	9.62E-05
protein binding (GO:0005515)	2440	30	14.39	+	2.08	1.02E-04	2.35E-03
binding (GO:0005488)	4589	53	27.07	+	1.96	2.73E-07	8.55E-06
molecular_function (GO:0003674)	8266	67	48.76	+	1.37	1.13E-03	2.23E-02
Unclassified (UNCLASSIFIED)	12585	56	74.24	-	.75	1.13E-03	2.15E-02

Supplementary Table S7. PANTHER GO-Term Analysis of Biological Process Over/Under-Representation for the 123 Predicted Human Interactors in the *Proximal* Schema.

PANTHER GO Biological Process	Homo sapiens Reference (N=20,851)	Num. Predicted	Predicted Num. Expected	Over/Under Represented	Fold Enrichment	*p*-value	FDR
neuron migration (GO:0001764)	6	3	.04	+	84.76	1.61E-05	1.19E-03
protein sumoylation (GO:0016925)	16	5	.09	+	52.98	1.21E-07	1.24E-05
chaperone-mediated protein folding (GO:0061077)	30	7	.18	+	39.55	1.79E-09	3.35E-07
response to unfolded protein (GO:0006986)	32	7	.19	+	37.08	2.64E-09	4.20E-07
cellular response to unfolded protein (GO:0034620)	32	7	.19	+	37.08	2.64E-09	3.90E-07
peptidyl-tyrosine phosphorylation (GO:0018108)	43	8	.25	+	31.54	5.73E-10	1.48E-07
peptidyl-tyrosine modification (GO:0018212)	45	8	.27	+	30.14	7.90E-10	1.81E-07
glycolytic process (GO:0006096)	18	3	.11	+	28.25	2.42E-04	1.52E-02
nucleotide phosphorylation (GO:0046939)	18	3	.11	+	28.25	2.42E-04	1.47E-02
cellular response to topologically incorrect protein (GO:0035967)	43	7	.25	+	27.60	1.63E-08	1.87E-06
response to topologically incorrect protein (GO:0035966)	43	7	.25	+	27.60	1.63E-08	1.77E-06
protein folding (GO:0006457)	96	9	.57	+	15.89	1.14E-08	1.39E-06
response to peptide hormone (GO:0043434)	57	4	.34	+	11.90	4.54E-04	2.53E-02
response to peptide (GO:1901652)	57	4	.34	+	11.90	4.54E-04	2.47E-02
transmembrane receptor protein tyrosine kinase signaling pathway (GO:0007169)	129	9	.76	+	11.83	1.24E-07	1.22E-05
regulation of cell population proliferation (GO:0042127)	136	9	.80	+	11.22	1.89E-07	1.78E-05
peptidyl-lysine modification (GO:0018205)	91	6	.54	+	11.18	2.30E-05	1.64E-03
microtubule cytoskeleton organization (GO:0000226)	315	19	1.86	+	10.23	8.97E-14	1.85E-10
cell population proliferation (GO:0008283)	153	9	.90	+	9.97	4.86E-07	4.36E-05
mitotic cell cycle (GO:0000278)	278	16	1.64	+	9.76	1.82E-11	1.25E-08
mitotic cell cycle process (GO:1903047)	278	16	1.64	+	9.76	1.82E-11	9.39E-09
mitotic nuclear division (GO:0140014)	278	16	1.64	+	9.76	1.82E-11	7.51E-09
nuclear division (GO:0000280)	321	16	1.89	+	8.45	1.38E-10	4.76E-08
organelle fission (GO:0048285)	340	16	2.01	+	7.98	3.10E-10	9.13E-08
microtubule-based process (GO:0007017)	408	19	2.41	+	7.89	6.87E-12	7.09E-09
peptidyl-amino acid modification (GO:0018193)	318	14	1.88	+	7.46	9.64E-09	1.24E-06
enzyme linked receptor protein signaling pathway (GO:0007167)	216	9	1.27	+	7.06	7.32E-06	6.04E-04
cell cycle process (GO:0022402)	430	17	2.54	+	6.70	1.07E-09	2.21E-07
cell cycle (GO:0007049)	477	17	2.81	+	6.04	4.81E-09	6.61E-07
cytoskeleton organization (GO:0007010)	587	19	3.46	+	5.49	2.54E-09	4.37E-07
cellular response to organic substance (GO:0071310)	444	12	2.62	+	4.58	1.57E-05	1.20E-03
cellular response to chemical stimulus (GO:0070887)	569	14	3.36	+	4.17	8.41E-06	6.67E-04
cellular response to stress (GO:0033554)	464	11	2.74	+	4.02	1.13E-04	7.29E-03
response to organic substance (GO:0010033)	514	12	3.03	+	3.96	6.30E-05	4.19E-03
phosphorylation (GO:0016310)	592	13	3.49	+	3.72	5.67E-05	3.90E-03

Supplementary Table S8. PANTHER GO-Term Analysis of Cellular Component Over/Under-Representation for the 123 Predicted Human Interactors in the *Proximal* Schema.

PANTHER GO Cellular Component	Homo sapiens Reference (N=20,851)	Num. Predicted	Predicted Num. Expected	Over/Under Represented	Fold Enrichment	*p*-value	FDR
COP9 signalosome (GO:0008180)	14	4	.08	+	48.43	3.23E-06	8.85E-05
PML body (GO:0016605)	8	2	.05	+	42.38	1.49E-03	2.35E-02
integral component of mitochondrial outer membrane (GO:0031307)	10	2	.06	+	33.90	2.17E-03	3.22E-02
extrinsic component of cytoplasmic side of plasma membrane (GO:0031234)	56	8	.33	+	24.22	3.74E-09	2.43E-07
cytoplasmic side of plasma membrane (GO:0009898)	62	8	.37	+	21.87	7.74E-09	4.47E-07
cytoplasmic side of membrane (GO:0098562)	67	8	.40	+	20.24	1.35E-08	7.02E-07
proton-transporting two-sector ATPase complex (GO:0016469)	26	3	.15	+	19.56	6.43E-04	1.19E-02
extrinsic component of plasma membrane (GO:0019897)	77	8	.45	+	17.61	3.67E-08	1.59E-06
integral component of mitochondrial membrane (GO:0032592)	35	3	.21	+	14.53	1.43E-03	2.32E-02
microtubule (GO:0005874)	168	14	.99	+	14.13	3.51E-12	1.83E-09
intrinsic component of mitochondrial membrane (GO:0098573)	37	3	.22	+	13.74	1.66E-03	2.54E-02
extrinsic component of membrane (GO:0019898)	122	8	.72	+	11.12	9.94E-07	3.04E-05
polymeric cytoskeletal fiber (GO:0099513)	249	15	1.47	+	10.21	4.42E-11	1.15E-08
supramolecular fiber (GO:0099512)	299	15	1.76	+	8.50	4.99E-10	6.49E-08
supramolecular polymer (GO:0099081)	302	15	1.78	+	8.42	5.69E-10	5.92E-08
supramolecular complex (GO:0099080)	302	15	1.78	+	8.42	5.69E-10	4.93E-08
microtubule cytoskeleton (GO:0015630)	382	17	2.25	+	7.54	1.89E-10	3.27E-08
cytoskeletal part (GO:0044430)	492	18	2.90	+	6.20	1.09E-09	8.09E-08
cytoskeleton (GO:0005856)	597	18	3.52	+	5.11	2.01E-08	9.49E-07
side of membrane (GO:0098552)	282	8	1.66	+	4.81	3.16E-04	6.33E-03
leaflet of membrane bilayer (GO:0097478)	282	8	1.66	+	4.81	3.16E-04	6.09E-03
cytosol (GO:0005829)	710	12	4.19	+	2.87	1.11E-03	1.86E-02
intracellular non-membrane-bounded organelle (GO:0043232)	1284	20	7.57	+	2.64	6.96E-05	1.81E-03
non-membrane-bounded organelle (GO:0043228)	1284	20	7.57	+	2.64	6.96E-05	1.72E-03
intracellular organelle part (GO:0044446)	2610	38	15.40	+	2.47	7.86E-08	3.14E-06
organelle part (GO:0044422)	2695	38	15.90	+	2.39	2.13E-07	7.91E-06
cytoplasm (GO:0005737)	4104	48	24.21	+	1.98	1.02E-06	2.93E-05
intracellular part (GO:0044424)	6630	67	39.11	+	1.71	2.72E-07	9.43E-06
intracellular (GO:0005622)	6661	67	39.29	+	1.71	2.97E-07	9.67E-06
intracellular organelle (GO:0043229)	5308	51	31.31	+	1.63	1.09E-04	2.57E-03
organelle (GO:0043226)	5421	51	31.98	+	1.59	1.89E-04	3.94E-03
cell part (GO:0044464)	8223	70	48.51	+	1.44	1.29E-04	2.91E-03
cell (GO:0005623)	8223	70	48.51	+	1.44	1.29E-04	2.79E-03
cellular_component (GO:0005575)	9090	72	53.62	+	1.34	9.82E-04	1.76E-02
Unclassified (UNCLASSIFIED)	11761	51	69.38	-	.74	9.82E-04	1.70E-02

Supplementary Table S9. PANTHER GO-Term Analysis of Molecular Function Over/Under-Representation for the 496 Predicted Human Interactors in the *RP-PPI* Schema (18 Unmapped).

PANTHER GO Molecular Function	Homo sapiens Reference (N=20,851)	Num. Predicted	Predicted Num. Expected	Over/Under Represented	Fold Enrichment	*p*-value	FDR
single-stranded RNA binding (GO:0003727)	31	13	.71	+	18.37	8.01E-12	5.33E-10
mRNA 3'-UTR binding (GO:0003730)	31	12	.71	+	16.96	1.09E-10	6.45E-09
snRNA binding (GO:0017069)	18	5	.41	+	12.17	1.31E-04	2.78E-03
unfolded protein binding (GO:0051082)	58	16	1.32	+	12.08	6.05E-12	4.60E-10
heat shock protein binding (GO:0031072)	30	7	.68	+	10.22	1.52E-05	4.05E-04
mRNA binding (GO:0003729)	139	32	3.17	+	10.08	1.45E-20	2.57E-18
ATP binding (GO:0005524)	40	9	.91	+	9.86	1.18E-06	3.71E-05
ubiquitin-like protein ligase binding (GO:0044389)	46	7	1.05	+	6.67	1.68E-04	3.30E-03
purine ribonucleoside triphosphate binding (GO:0035639)	154	20	3.52	+	5.69	2.28E-09	9.31E-08
purine ribonucleotide binding (GO:0032555)	180	23	4.11	+	5.60	1.89E-10	1.01E-08
ribonucleotide binding (GO:0032553)	186	23	4.25	+	5.42	3.40E-10	1.64E-08
purine nucleotide binding (GO:0017076)	192	23	4.38	+	5.25	5.98E-10	2.65E-08
drug binding (GO:0008144)	121	14	2.76	+	5.07	2.04E-06	5.72E-05
isomerase activity (GO:0016853)	97	10	2.21	+	4.52	1.42E-04	2.91E-03
RNA binding (GO:0003723)	516	52	11.78	+	4.41	5.09E-18	6.76E-16
nucleoside phosphate binding (GO:1901265)	236	23	5.39	+	4.27	2.17E-08	7.68E-07
nucleotide binding (GO:0000166)	236	23	5.39	+	4.27	2.17E-08	7.20E-07
GTP binding (GO:0005525)	114	11	2.60	+	4.23	1.15E-04	2.55E-03
carbohydrate derivative binding (GO:0097367)	255	24	5.82	+	4.12	1.98E-08	7.52E-07
structural molecule activity (GO:0005198)	215	17	4.91	+	3.46	1.97E-05	4.98E-04
ATPase activity, coupled (GO:0042623)	117	9	2.67	+	3.37	2.10E-03	3.49E-02
small molecule binding (GO:0036094)	377	26	8.61	+	3.02	1.49E-06	4.40E-05
heterocyclic compound binding (GO:1901363)	1646	110	37.58	+	2.93	8.03E-24	2.14E-21
organic cyclic compound binding (GO:0097159)	1677	112	38.28	+	2.93	2.93E-24	1.56E-21
nucleic acid binding (GO:0003676)	1325	86	30.25	+	2.84	9.45E-18	1.00E-15
anion binding (GO:0043168)	484	27	11.05	+	2.44	4.43E-05	1.07E-03
DNA binding (GO:0003677)	806	35	18.40	+	1.90	4.59E-04	7.87E-03
binding (GO:0005488)	4589	176	104.76	+	1.68	2.53E-13	2.24E-11
molecular_function (GO:0003674)	8266	229	188.70	+	1.21	2.17E-04	4.13E-03
Unclassified (UNCLASSIFIED)	12585	247	287.30	-	.86	2.17E-04	3.99E-03
transmembrane signaling receptor activity (GO:0004888)	640	4	14.61	-	.27	2.50E-03	4.03E-02
signaling receptor activity (GO:0038023)	749	4	17.10	-	.23	3.52E-04	6.25E-03
molecular transducer activity (GO:0060089)	820	4	18.72	-	.21	9.10E-05	2.11E-03

Supplementary Table S10. PANTHER GO-Term Analysis of Biological Process Over/Under-Representation for the 496 Predicted Human Interactors in the *RP-PPII* Schema (18 Unmapped).

PANTHER GO Biological Process	Homo sapiens Reference (N=20,851)	Num. Predicted	Predicted Num. Expected	Over/Under Represented	Fold Enrichment	*p*-value	FDR
mRNA stabilization (GO:0048255)	11	5	.25	+	19.91	1.93E-05	7.53E-04
positive regulation of translational initiation (GO:0045948)	9	4	.21	+	19.47	1.49E-04	4.22E-03
peptide hormone secretion (GO:0030072)	8	3	.18	+	16.43	1.60E-03	2.79E-02
protein sumoylation (GO:0016925)	16	6	.37	+	16.43	6.60E-06	3.40E-04
chaperone-mediated protein folding (GO:0061077)	30	11	.68	+	16.06	1.05E-09	1.45E-07
regulation of mRNA splicing, via spliceosome (GO:0048024)	56	20	1.28	+	15.64	2.18E-16	4.50E-14
regulation of hormone secretion (GO:0046883)	9	3	.21	+	14.60	2.09E-03	3.63E-02
regulation of mRNA processing (GO:0050684)	67	22	1.53	+	14.38	2.93E-17	1.21E-14
regulation of RNA splicing (GO:0043484)	65	21	1.48	+	14.15	2.04E-16	4.69E-14
mRNA splice site selection (GO:0006376)	13	4	.30	+	13.48	4.63E-04	1.04E-02
regulation of alternative mRNA splicing, via spliceosome (GO:0000381)	39	12	.89	+	13.48	9.60E-10	1.42E-07
hormone secretion (GO:0046879)	10	3	.23	+	13.14	2.67E-03	4.35E-02
hormone transport (GO:0009914)	10	3	.23	+	13.14	2.67E-03	4.31E-02
alternative mRNA splicing, via spliceosome (GO:0000380)	40	12	.91	+	13.14	1.22E-09	1.58E-07
regulation of mRNA metabolic process (GO:1903311)	87	26	1.99	+	13.09	2.58E-19	5.33E-16
regulation of mRNA stability (GO:0043488)	21	6	.48	+	12.52	2.38E-05	9.10E-04
regulation of RNA stability (GO:0043487)	21	6	.48	+	12.52	2.38E-05	8.93E-04
establishment of mitotic spindle orientation (GO:0000132)	15	4	.34	+	11.68	7.28E-04	1.52E-02
nucleus localization (GO:0051647)	16	4	.37	+	10.95	8.94E-04	1.81E-02
response to unfolded protein (GO:0006986)	32	8	.73	+	10.95	2.38E-06	1.49E-04
nuclear migration (GO:0007097)	16	4	.37	+	10.95	8.94E-04	1.79E-02
establishment of mitotic spindle localization (GO:0040001)	16	4	.37	+	10.95	8.94E-04	1.77E-02
cellular response to unfolded protein (GO:0034620)	32	8	.73	+	10.95	2.38E-06	1.45E-04
positive regulation of cellular amide metabolic process (GO:0034250)	25	6	.57	+	10.51	5.49E-05	1.92E-03
positive regulation of translation (GO:0045727)	25	6	.57	+	10.51	5.49E-05	1.89E-03
regulation of mRNA catabolic process (GO:0061013)	25	6	.57	+	10.51	5.49E-05	1.86E-03
establishment of spindle orientation (GO:0051294)	17	4	.39	+	10.31	1.08E-03	2.09E-02
regulation of translational initiation (GO:0006446)	18	4	.41	+	9.73	1.30E-03	2.44E-02

Supplementary Table S11. PANTHER GO-Term Analysis of Cellular Component Over/Under-Representation for the 496 Predicted Human Interactors in the *RP-PPI* Schema (18 Unmapped).

PANTHER GO Cellular Component	Homo sapiens Reference (N=20,851)	Num. Predicted	Predicted Num. Expected	Over/Under Represented	Fold Enrichment	*p*-value	FDR
Golgi cis cisterna (GO:0000137)	22	7	.50	+	13.94	2.69E-06	7.00E-05
cytoplasmic stress granule (GO:0010494)	16	5	.37	+	13.69	8.21E-05	1.42E-03
polysome (GO:0005844)	13	4	.30	+	13.48	4.63E-04	6.02E-03
COP9 signalosome (GO:0008180)	14	4	.32	+	12.52	5.85E-04	7.24E-03
Golgi cisterna (GO:0031985)	29	7	.66	+	10.57	1.26E-05	2.97E-04
nuclear speck (GO:0016607)	28	6	.64	+	9.39	9.47E-05	1.54E-03
U12-type spliceosomal complex (GO:0005689)	14	3	.32	+	9.39	5.95E-03	4.91E-02
Golgi stack (GO:0005795)	33	7	.75	+	9.29	2.60E-05	5.88E-04
cis-Golgi network (GO:0005801)	33	7	.75	+	9.29	2.60E-05	5.64E-04
cell cortex region (GO:0099738)	20	4	.46	+	8.76	1.83E-03	1.86E-02
nuclear body (GO:0016604)	46	7	1.05	+	6.67	1.68E-04	2.35E-03
cytoplasmic ribonucleoprotein granule (GO:0036464)	69	9	1.58	+	5.71	5.94E-05	1.10E-03
ribonucleoprotein granule (GO:0035770)	69	9	1.58	+	5.71	5.94E-05	1.06E-03
spliceosomal complex (GO:0005681)	109	13	2.49	+	5.22	3.49E-06	8.64E-05
extrinsic component of cytoplasmic side of plasma membrane (GO:0031234)	56	6	1.28	+	4.69	2.56E-03	2.47E-02
cytoplasmic side of plasma membrane (GO:0009898)	62	6	1.42	+	4.24	4.08E-03	3.53E-02
nuclear chromatin (GO:0000790)	130	12	2.97	+	4.04	8.42E-05	1.41E-03
cytoplasmic side of membrane (GO:0098562)	67	6	1.53	+	3.92	5.78E-03	4.93E-02
centriole (GO:0005814)	145	12	3.31	+	3.63	2.18E-04	2.98E-03
Golgi membrane (GO:0000139)	97	8	2.21	+	3.61	2.45E-03	2.40E-02
microtubule organizing center part (GO:0044450)	146	12	3.33	+	3.60	2.31E-04	3.09E-03
chromatin (GO:0000785)	159	13	3.63	+	3.58	1.35E-04	1.95E-03
ribonucleoprotein complex (GO:1990904)	457	37	10.43	+	3.55	1.47E-10	9.56E-09
Golgi subcompartment (GO:0098791)	99	8	2.26	+	3.54	2.75E-03	2.56E-02
organelle subcompartment (GO:0031984)	104	8	2.37	+	3.37	3.65E-03	3.22E-02
centrosome (GO:0005813)	132	10	3.01	+	3.32	1.35E-03	1.43E-02
polymeric cytoskeletal fiber (GO:0099513)	249	17	5.68	+	2.99	1.09E-04	1.71E-03
supramolecular fiber (GO:0099512)	299	20	6.83	+	2.93	3.62E-05	7.53E-04
supramolecular polymer (GO:0099081)	302	20	6.89	+	2.90	4.13E-05	8.26E-04
supramolecular complex (GO:0099080)	302	20	6.89	+	2.90	4.13E-05	7.95E-04
microtubule (GO:0005874)	168	11	3.84	+	2.87	2.35E-03	2.35E-02
microtubule organizing center (GO:0005815)	184	12	4.20	+	2.86	1.57E-03	1.63E-02
nuclear chromosome (GO:0000228)	228	14	5.20	+	2.69	1.14E-03	1.32E-02
nuclear chromosome part (GO:0044454)	224	13	5.11	+	2.54	2.70E-03	2.55E-02
microtubule cytoskeleton (GO:0015630)	382	22	8.72	+	2.52	1.22E-04	1.86E-03
cytosol (GO:0005829)	710	39	16.21	+	2.41	9.83E-07	2.84E-05
cytoskeletal part (GO:0044430)	492	26	11.23	+	2.31	1.22E-04	1.82E-03
chromosome (GO:0005694)	312	16	7.12	+	2.25	3.61E-03	3.24E-02
chromosomal part (GO:0044427)	300	15	6.85	+	2.19	5.81E-03	4.87E-02
cytoskeleton (GO:0005856)	597	28	13.63	+	2.05	4.87E-04	6.18E-03
intracellular non-membrane-bounded organelle (GO:0043232)	1284	60	29.31	+	2.05	2.78E-07	9.04E-06
non-membrane-bounded organelle (GO:0043228)	1284	60	29.31	+	2.05	2.78E-07	8.51E-06

Supplementary Table S12. GO-Terms Overlapping with Gordon *et al.*

GO-term	GO-name
GO:0000139	Golgi membrane
GO:0000209	protein polyubiquitination
GO:0000398	mRNA splicing via spliceosome
GO:0000776	kinetochore
GO:0001525	angiogenesis
GO:0001618	virus receptor activity
GO:0001701	in utero embryonic development
GO:0001889	liver development
GO:0001932	regulation of protein phosphorylation
GO:0002020	protease binding
GO:0002576	platelet degranulation
GO:0002931	response to ischemia
GO:0003682	chromatin binding
GO:0003697	single-stranded DNA binding
GO:0003714	transcription corepressor activity
GO:0003723	RNA binding
GO:0003729	mRNA binding
GO:0003730	mRNA 3'-UTR binding
GO:0004842	ubiquitin-protein transferase activity
GO:0005102	signaling receptor binding
GO:0005178	integrin binding
GO:0005201	extracellular matrix structural constituent
GO:0005518	collagen binding
GO:0005524	ATP binding
GO:0005543	phospholipid binding
GO:0005576	extracellular region
GO:0005604	basement membrane
GO:0005615	extracellular space
GO:0005634	nucleus
GO:0005654	nucleoplasm
GO:0005681	spliceosomal complex
GO:0005730	nucleolus
GO:0005737	cytoplasm
GO:0005739	mitochondrion
GO:0005769	early endosome
GO:0005783	endoplasmic reticulum
GO:0005788	endoplasmic reticulum lumen
GO:0005789	endoplasmic reticulum membrane
GO:0005790	smooth endoplasmic reticulum
GO:0005793	endoplasmic reticulum-Golgi intermediate compartment
GO:0005794	Golgi apparatus
GO:0005811	lipid droplet
GO:0005813	centrosome
GO:0005814	centriole
GO:0005829	cytosol
GO:0005886	plasma membrane
GO:0005925	focal adhesion
GO:0006281	DNA repair
GO:0006355	regulation of transcription DNA-templated
GO:0006396	RNA processing
GO:0006397	mRNA processing
GO:0006401	RNA catabolic process
GO:0006405	RNA export from nucleus
GO:0006406	mRNA export from nucleus
GO:0006511	ubiquitin-dependent protein catabolic process
GO:0006515	protein quality control for misfolded or incompletely synthesized proteins
GO:0006874	cellular calcium ion homeostasis
GO:0006888	endoplasmic reticulum to Golgi vesicle-mediated transport
GO:0006898	receptor-mediated endocytosis
GO:0006954	inflammatory response
GO:0006986	response to unfolded protein
GO:0007029	endoplasmic reticulum organization
GO:0007155	cell adhesion

GO:0007160	cell-matrix adhesion
GO:0007161	calcium-independent cell-matrix adhesion
GO:0007229	integrin-mediated signaling pathway
GO:0007283	spermatogenesis
GO:0007399	nervous system development
GO:0007507	heart development
GO:0008017	microtubule binding
GO:0008022	protein C-terminus binding
GO:0008134	transcription factor binding
GO:0008201	heparin binding
GO:0008203	cholesterol metabolic process
GO:0008284	positive regulation of cell population proliferation
GO:0008285	negative regulation of cell population proliferation
GO:0008360	regulation of cell shape
GO:0008543	fibroblast growth factor receptor signaling pathway
GO:0009615	response to virus
GO:0009791	post-embryonic development
GO:0010008	endosome membrane
GO:0010269	response to selenium ion
GO:0010628	positive regulation of gene expression
GO:0010886	positive regulation of cholesterol storage
GO:0016020	membrane
GO:0016021	integral component of membrane
GO:0016032	viral process
GO:0016070	RNA metabolic process
GO:0016192	vesicle-mediated transport
GO:0016234	inclusion body
GO:0016324	apical plasma membrane
GO:0016363	nuclear matrix
GO:0016567	protein ubiquitination
GO:0016605	PML body
GO:0016607	nuclear speck
GO:0016887	ATPase activity
GO:0016925	protein sumoylation
GO:0019221	cytokine-mediated signaling pathway
GO:0019899	enzyme binding
GO:0019900	kinase binding
GO:0019901	protein kinase binding
GO:0019904	protein domain specific binding
GO:0021549	cerebellum development
GO:0030054	cell junction
GO:0030198	extracellular matrix organization
GO:0030301	cholesterol transport
GO:0030308	negative regulation of cell growth
GO:0030433	ubiquitin-dependent ERAD pathway
GO:0030544	Hsp70 protein binding
GO:0030669	clathrin-coated endocytic vesicle membrane
GO:0030674	protein-macromolecule adaptor activity
GO:0030911	TPR domain binding
GO:0030968	endoplasmic reticulum unfolded protein response
GO:0031072	heat shock protein binding
GO:0031093	platelet alpha granule lumen
GO:0031397	negative regulation of protein ubiquitination
GO:0031398	positive regulation of protein ubiquitination
GO:0031625	ubiquitin protein ligase binding
GO:0031647	regulation of protein stability
GO:0031982	vesicle
GO:0032091	negative regulation of protein binding
GO:0032204	regulation of telomere maintenance
GO:0032436	positive regulation of proteasomal ubiquitin-dependent protein catabolic process
GO:0032496	response to lipopolysaccharide
GO:0032757	positive regulation of interleukin-8 production
GO:0032991	protein-containing complex
GO:0033344	cholesterol efflux
GO:0034361	very-low-density lipoprotein particle
GO:0034362	low-density lipoprotein particle

GO:0034379	very-low-density lipoprotein particle assembly
GO:0034383	low-density lipoprotein particle clearance
GO:0034446	substrate adhesion-dependent cell spreading
GO:0034450	ubiquitin-ubiquitin ligase activity
GO:0034599	cellular response to oxidative stress
GO:0034605	cellular response to heat
GO:0035198	miRNA binding
GO:0035722	interleukin-12-mediated signaling pathway
GO:0035925	mRNA 3'-UTR AU-rich region binding
GO:0035987	endodermal cell differentiation
GO:0036498	IRE1-mediated unfolded protein response
GO:0038128	ERBB2 signaling pathway
GO:0042060	wound healing
GO:0042162	telomeric DNA binding
GO:0042405	nuclear inclusion body
GO:0042632	cholesterol homeostasis
GO:0042789	mRNA transcription by RNA polymerase II
GO:0042802	identical protein binding
GO:0042803	protein homodimerization activity
GO:0042826	histone deacetylase binding
GO:0043025	neuronal cell body
GO:0043066	negative regulation of apoptotic process
GO:0043161	proteasome-mediated ubiquitin-dependent protein catabolic process
GO:0043202	lysosomal lumen
GO:0043231	intracellular membrane-bounded organelle
GO:0043312	neutrophil degranulation
GO:0043392	negative regulation of DNA binding
GO:0043488	regulation of mRNA stability
GO:0043565	sequence-specific DNA binding
GO:0043687	post-translational protein modification
GO:0044267	cellular protein metabolic process
GO:0044389	ubiquitin-like protein ligase binding
GO:0044829	positive regulation by host of viral genome replication
GO:0045070	positive regulation of viral genome replication
GO:0045296	cadherin binding
GO:0045727	positive regulation of translation
GO:0045862	positive regulation of proteolysis
GO:0045893	positive regulation of transcription DNA-templated
GO:0045944	positive regulation of transcription by RNA polymerase II
GO:0046034	ATP metabolic process
GO:0046332	SMAD binding
GO:0046872	metal ion binding
GO:0046982	protein heterodimerization activity
GO:0047485	protein N-terminus binding
GO:0048156	tau protein binding
GO:0048255	mRNA stabilization
GO:0048471	perinuclear region of cytoplasm
GO:0048487	beta-tubulin binding
GO:0050750	low-density lipoprotein particle receptor binding
GO:0050821	protein stabilization
GO:0050900	leukocyte migration
GO:0051028	mRNA transport
GO:0051082	unfolded protein binding
GO:0051085	chaperone cofactor-dependent protein refolding
GO:0051087	chaperone binding
GO:0051092	positive regulation of NF-kappaB transcription factor activity
GO:0051131	chaperone-mediated protein complex assembly
GO:0051170	import into nucleus
GO:0051592	response to calcium ion
GO:0051602	response to electrical stimulus
GO:0051702	biological process involved in interaction with symbiont
GO:0051787	misfolded protein binding
GO:0051865	protein autoubiquitination
GO:0051879	Hsp90 protein binding
GO:0060548	negative regulation of cell death
GO:0061024	membrane organization

GO:0061158	3'-UTR-mediated mRNA destabilization
GO:0061630	ubiquitin protein ligase activity
GO:0062023	collagen-containing extracellular matrix
GO:0070062	extracellular exosome
GO:0070370	cellular heat acclimation
GO:0070534	protein K63-linked ubiquitination
GO:0070971	endoplasmic reticulum exit site
GO:0071013	catalytic step 2 spliceosome
GO:0071230	cellular response to amino acid stimulus
GO:0071356	cellular response to tumor necrosis factor
GO:0071456	cellular response to hypoxia
GO:0071682	endocytic vesicle lumen
GO:0072562	blood microparticle
GO:0090063	positive regulation of microtubule nucleation
GO:0097157	pre-mRNA intronic binding
GO:0097718	disordered domain specific binding
GO:0120020	cholesterol transfer activity
GO:1900034	regulation of cellular response to heat
GO:1901673	regulation of mitotic spindle assembly
GO:1902236	negative regulation of endoplasmic reticulum stress-induced intrinsic apoptotic signaling pathway
GO:1903265	positive regulation of tumor necrosis factor-mediated signaling pathway
GO:1904813	ficolin-1-rich granule lumen
GO:1990837	sequence-specific double-stranded DNA binding
GO:1990904	ribonucleoprotein complex
GO:2001240	negative regulation of extrinsic apoptotic signaling pathway in absence of ligand
