# Supplementary material for: Multi-schema computational prediction of the comprehensive SARS-CoV-2 vs. human interactome
Source: PeerJ. 2021 Apr 5;9:e11117. doi: 10.7717/peerj.11117 (PMC8029698; doi:10.7717/peerj.11117)
Supplement: Supplemental Information 2 — Panel (A) depicts a taxonometric tree of the family Coronaviridae. Panel (B) presents the structure and composition of coronaviruses; the Coronavirus structure depicted is representative of CoVs from all four genera and lineages, whereas the structure depicted by Betacoronavirus (Lineage A) is specific to CoV species within this taxon. Panel (C) depicts the discovery of HCoVs throughout history in combination with the animal origin and the known, suspected, or unknown intermediate host prior to infection and propagation within the human population. For each virus we depict the initial attachment (orange) and main fusion (blue) receptors utilized by HCoVs for cell entry. Finally, we illustrate the genomic organization of each of the HCoVs. ORF1a and ORF1b (blue) encode for polyproteins. A ribosomal frameshift occurs at a slippery sequence and pseudoknot structure located between ORF1a and ORF1b (open circle). Downstream open reading frames encode the Spike (S), Envelope (E), Membrane (M), Nucleocapsid (N), and Hemagglutinin Esterase (HE) structural proteins (red), as well as accessory proteins (light blue box, black text). Gene mappings not shown to scale. This figure was created with biorender.com. [file peerj-09-11117-s002.pdf]

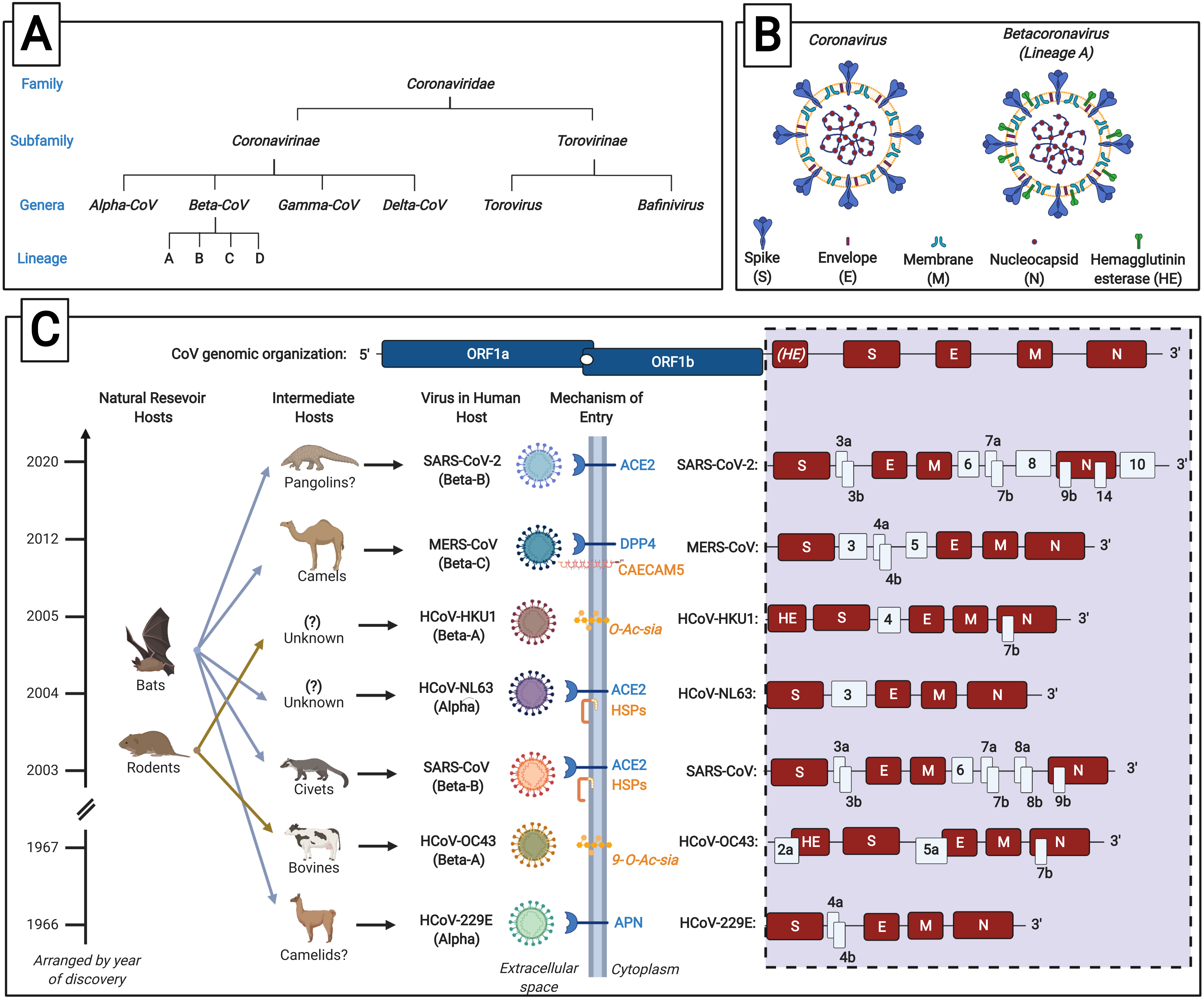

A
Family
Coronaviridae
Subfamily
Coronavirinae
Torovirinae
Genera
Alpha-CoV
Beta-CoV
Gamma-CoV
Delta-CoV
Torovirus
Bafinivirus
Lineage
A B C D
B
Coronavirus
Betacoronavirus (Lineage A)
Spike (S)
Envelope (E)
Membrane (M)
Nucleocapsid (N)
Hemagglutinin esterase (HE)
C
CoV genomic organization: 5'
ORF1a
ORF1b
(HE) S E M N 3'
Natural Resevoir Hosts
Intermediate Hosts
Virus in Human Host
Mechanism of Entry
2020
2012
2005
2004
2003
1967
1966
Arranged by year of discovery
Bats
Rodents
Pangolins?
Camels
(?) Unknown
(?) Unknown
Civets
Bovines
Camelids?
SARS-CoV-2 (Beta-B)
MERS-CoV (Beta-C)
HCoV-HKU1 (Beta-A)
HCoV-NL63 (Alpha)
SARS-CoV (Beta-B)
HCoV-OC43 (Beta-A)
HCoV-229E (Alpha)
ACE2
DPP4
CAECAM5
O-Ac-sia
ACE2
HSPs
ACE2
HSPs
9-O-Ac-sia
APN
Extracellular space
Cytoplasm
SARS-CoV-2: S 3a 3b E M 6 7a 7b 8 N 9b 14 10 3'
MERS-CoV: S 3 4a 4b 5 E M N 3'
HCoV-HKU1: HE S 4 E M N 7b 3'
HCoV-NL63: S 3 E M N 3'
SARS-CoV: S 3a 3b E M 6 7a 7b 8a 8b N 9b 3'
HCoV-OC43: 2a HE S 5a E M N 7b 3'
HCoV-229E: S 4a 4b E M N 3'
