# Supplementary material for: Multi-schema computational prediction of the comprehensive SARS-CoV-2 vs. human interactome
Source: PeerJ. 2021 Apr 5;9:e11117. doi: 10.7717/peerj.11117 (PMC8029698; doi:10.7717/peerj.11117)
Supplement: Supplemental Information 3 — The top panels depict the combination of one-to-all score curves for each protein, by each predictor and each subplot is a top-k subset of the previous; highlighted in blue. Selected example from the all schema. [file peerj-09-11117-s003.pdf]

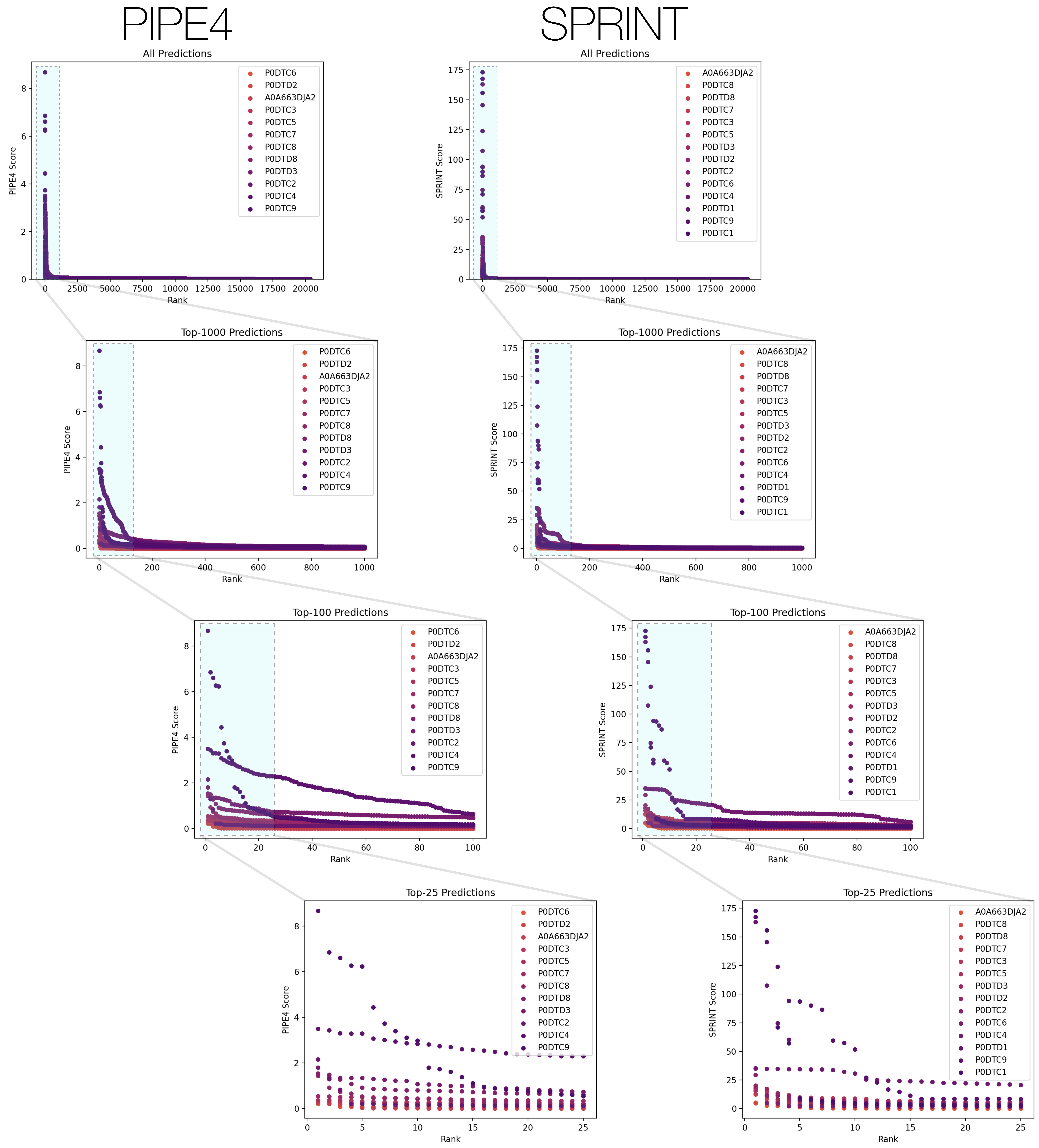
PIPE4
SPRINT
All Predictions
Top-1000 Predictions
Top-100 Predictions
Top-25 Predictions
PIPE4 Score
SPRINT Score
Rank
P0DTC6
P0DTD2
A0A663DJA2
P0DTC3
P0DTC5
P0DTC7
P0DTC8
P0DTD8
P0DTD3
P0DTC2
P0DTC4
P0DTC9
A0A663DJA2
P0DTC8
P0DTD8
P0DTC7
P0DTC3
P0DTC5
P0DTD3
P0DTD2
P0DTC2
P0DTC6
P0DTC4
P0DTD1
P0DTC9
P0DTC1
