# Supplementary figures and images for: Multi-schema computational prediction of the comprehensive SARS-CoV-2 vs. human interactome
Source: PeerJ. 2021 Apr 5;9:e11117. doi: 10.7717/peerj.11117 (PMC8029698; doi:10.7717/peerj.11117)

# Conceptual Overview of a PIPE4 Landscape & the Three Predicted Sites of Interaction (PIPE-Sites)

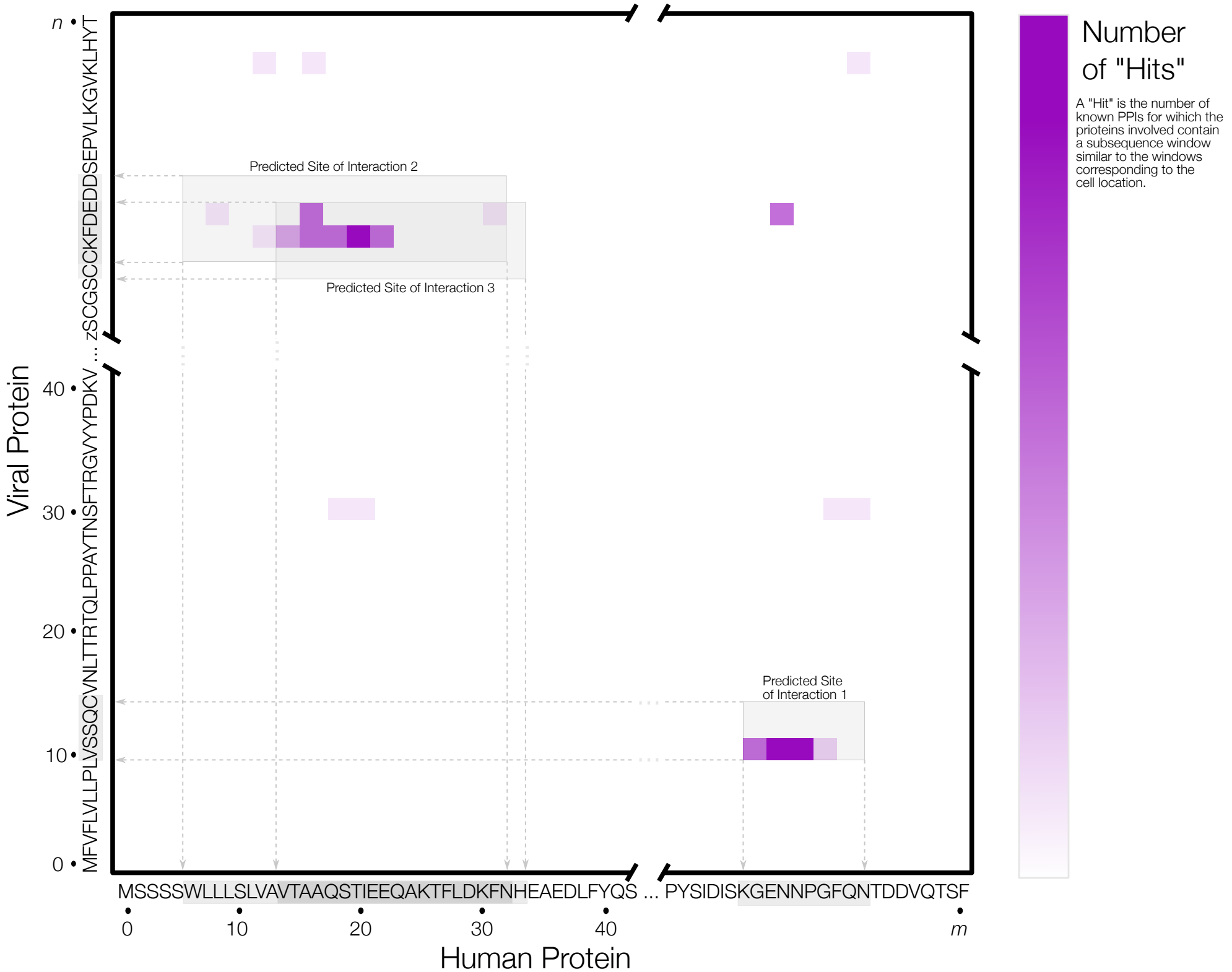

Supplement: Supplemental Information 4 [file peerj-09-11117-s004.pdf]

# Joint RP-PPI XGBoost Hyperparameter Tuning

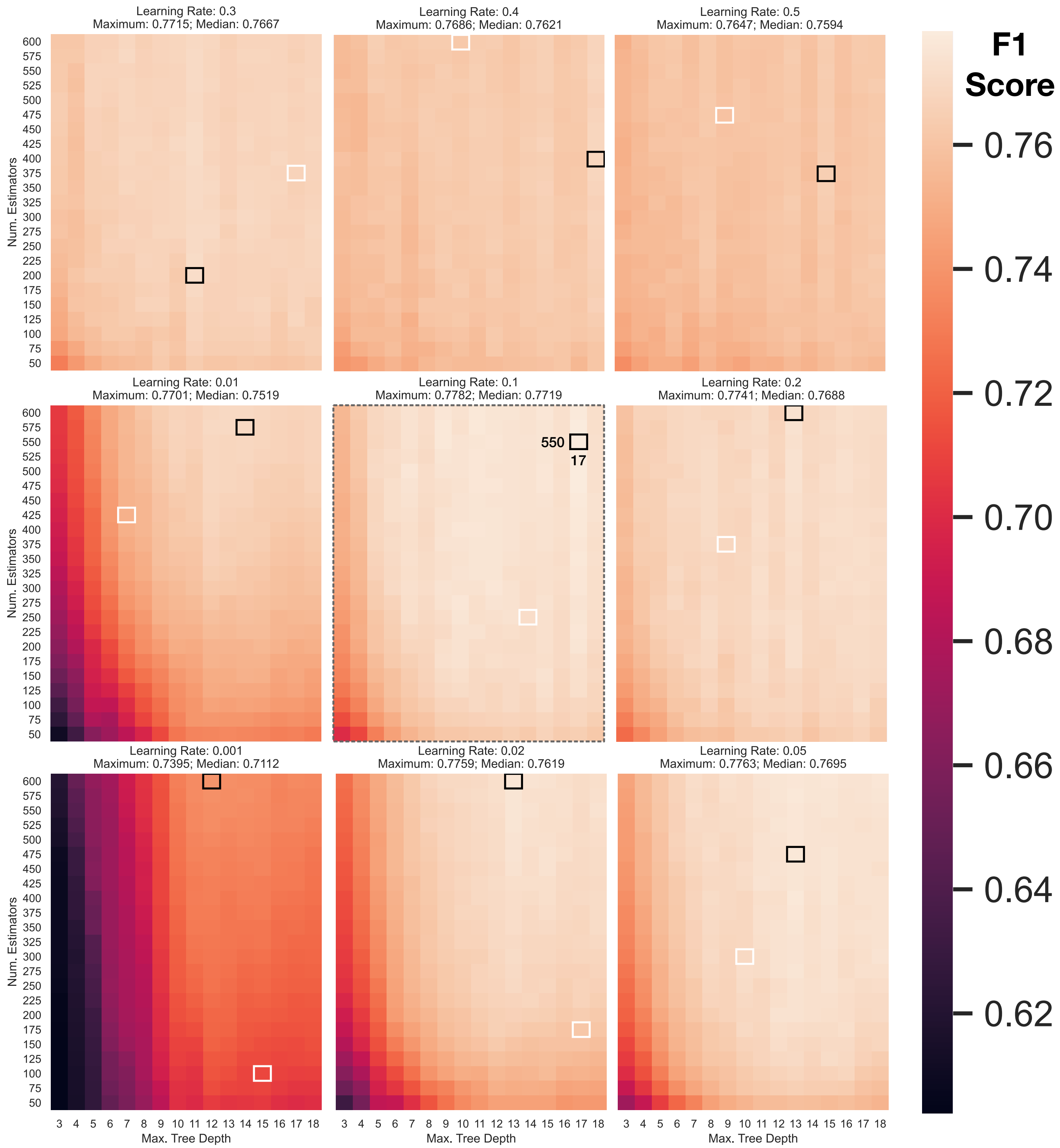

Supplement: Supplemental Information 6 — Each of the nine subplots depicts the results keeping the learning rate fixed as we vary the maximum tree depth (x-axis) between [3,18] by increments of 1 and the number of estimators (y-axis) between [50,600] by increments of 25. Within each subplot, we highlight the maximum value with a black bounding box and the median value with a white bounding box. All results are normalized to the same colour range where lighter values represent better performing models. The best performance is achieved with a learning rate of 0.1, a maximum tree-depth of 17 and 550 estimators. [file peerj-09-11117-s006.pdf]

## RP-PPI Joint Features

## Relative Feature Importance

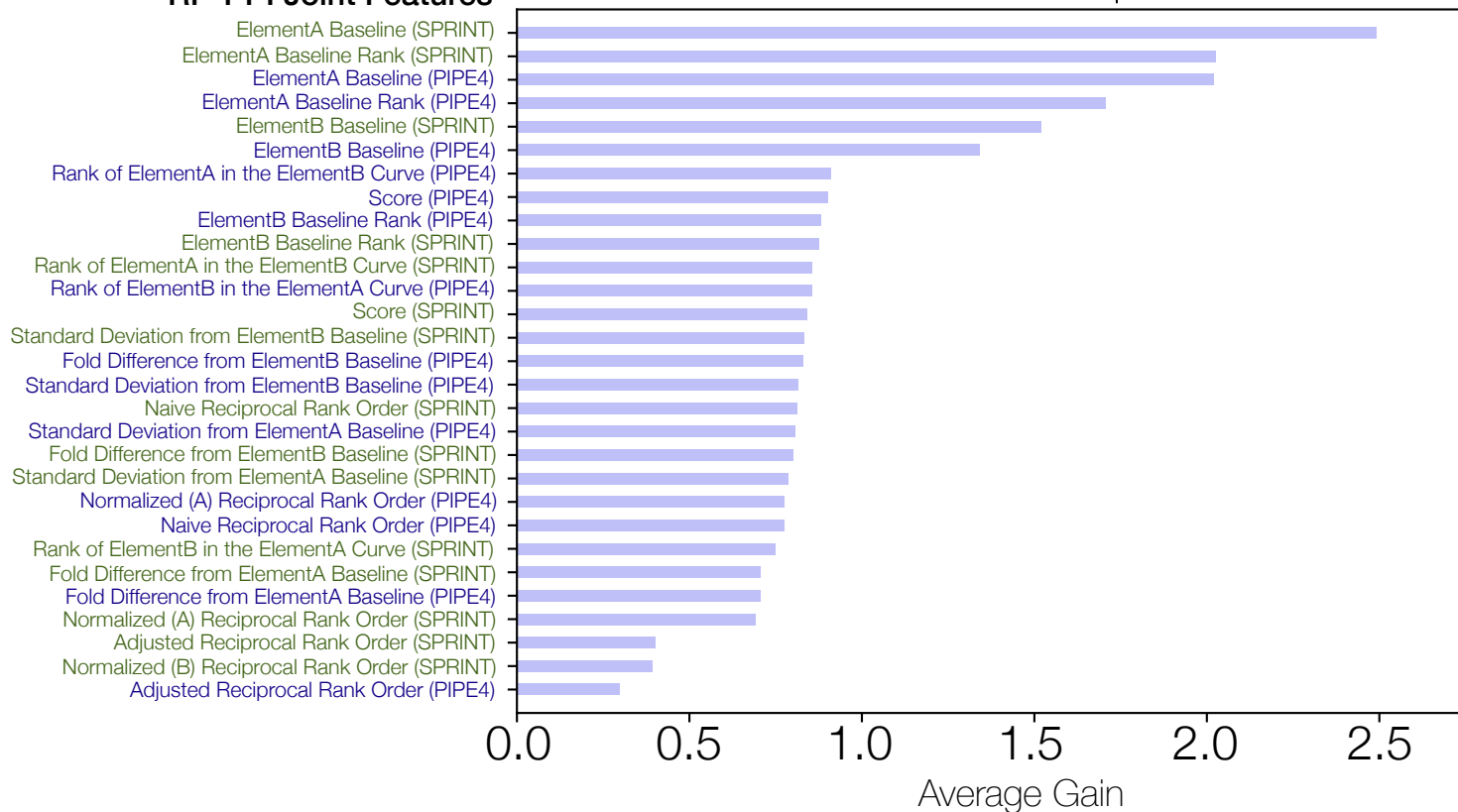

Supplement: Supplemental Information 7 — The combined RP features from each model are sorted by relative importance, measured as the average information gain. [file peerj-09-11117-s007.pdf]

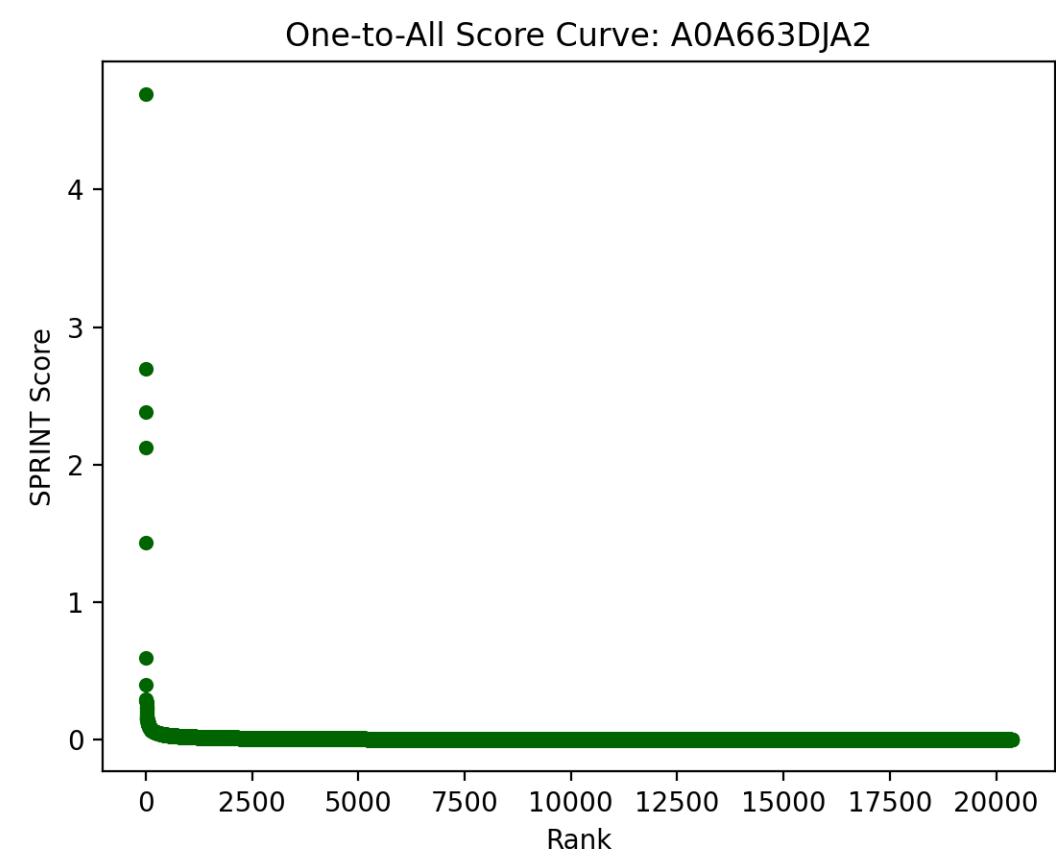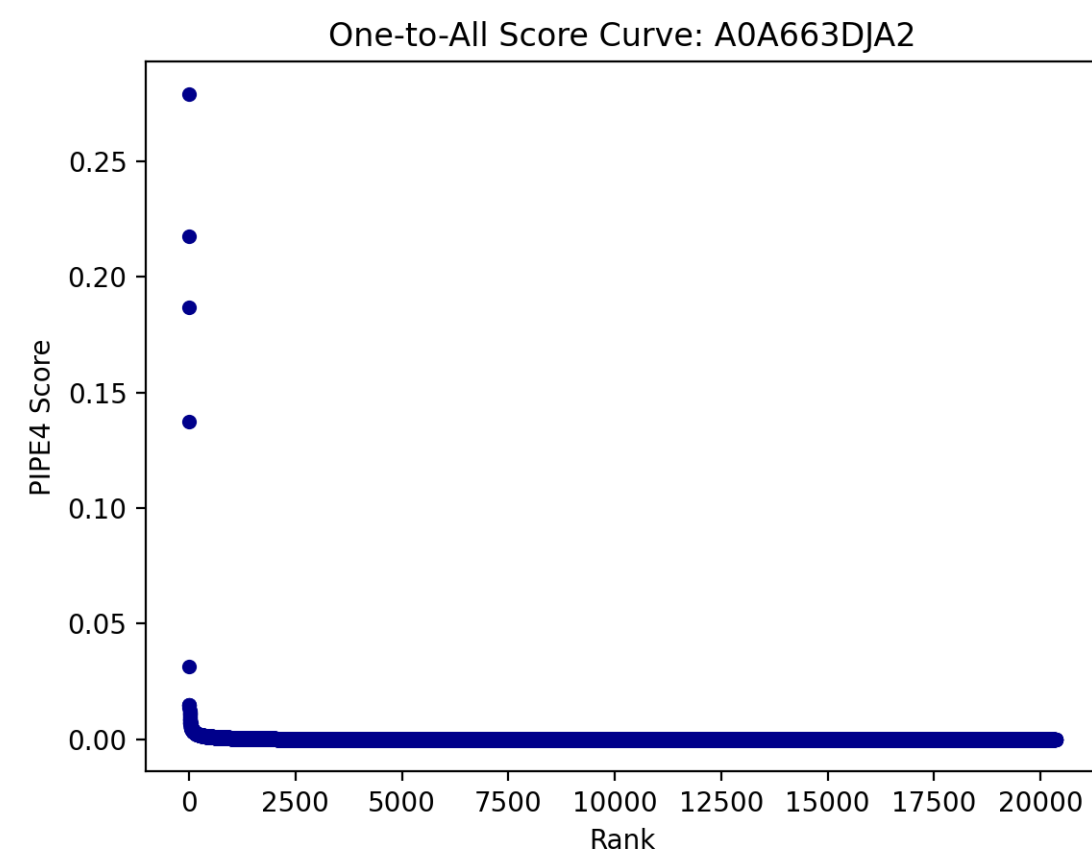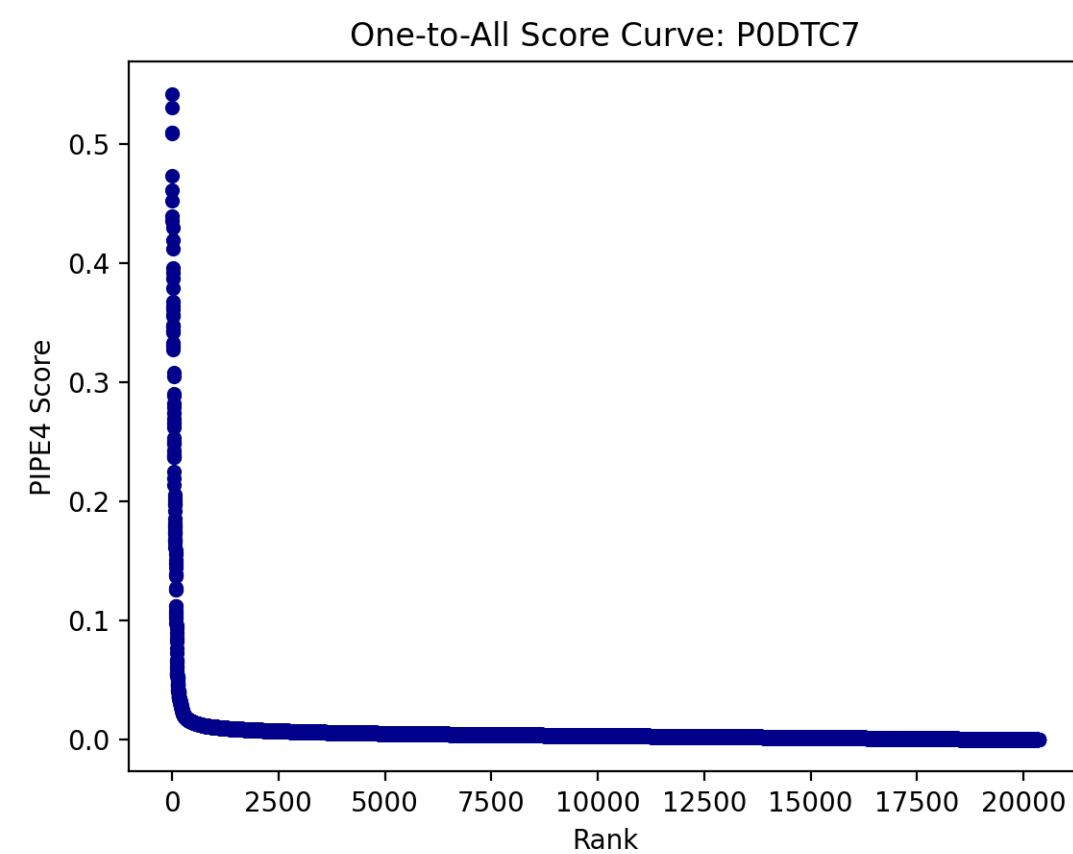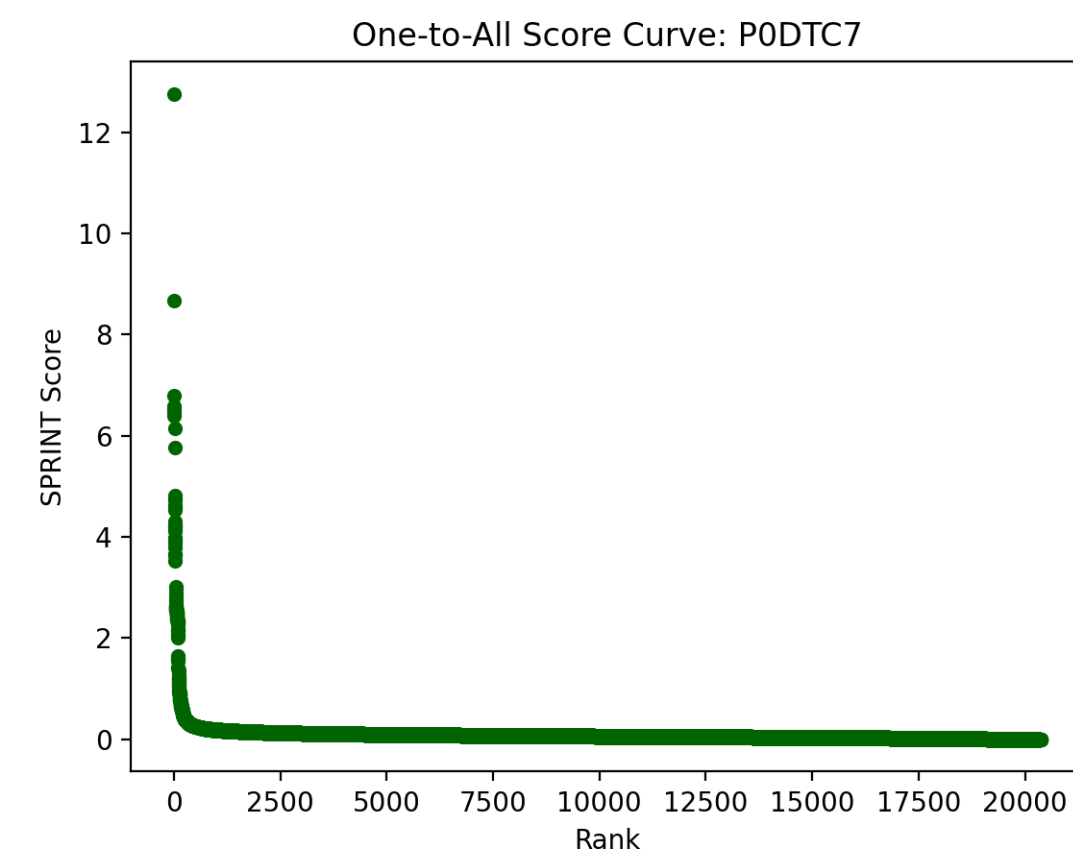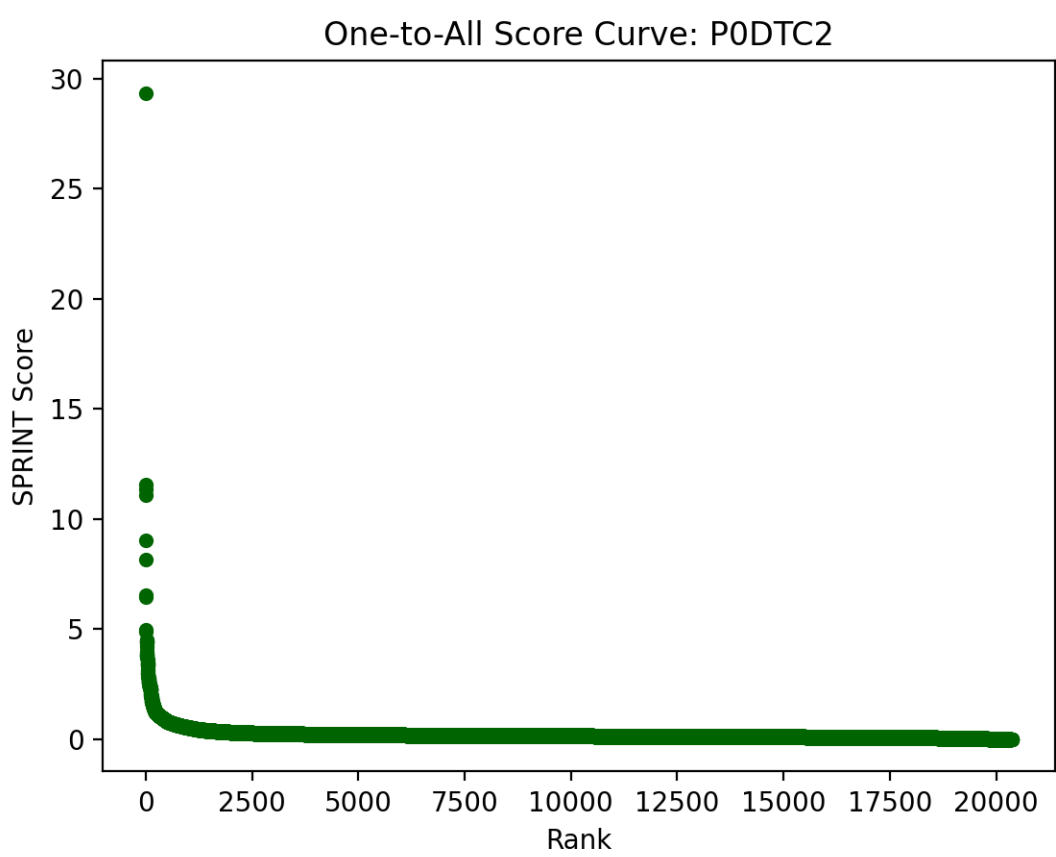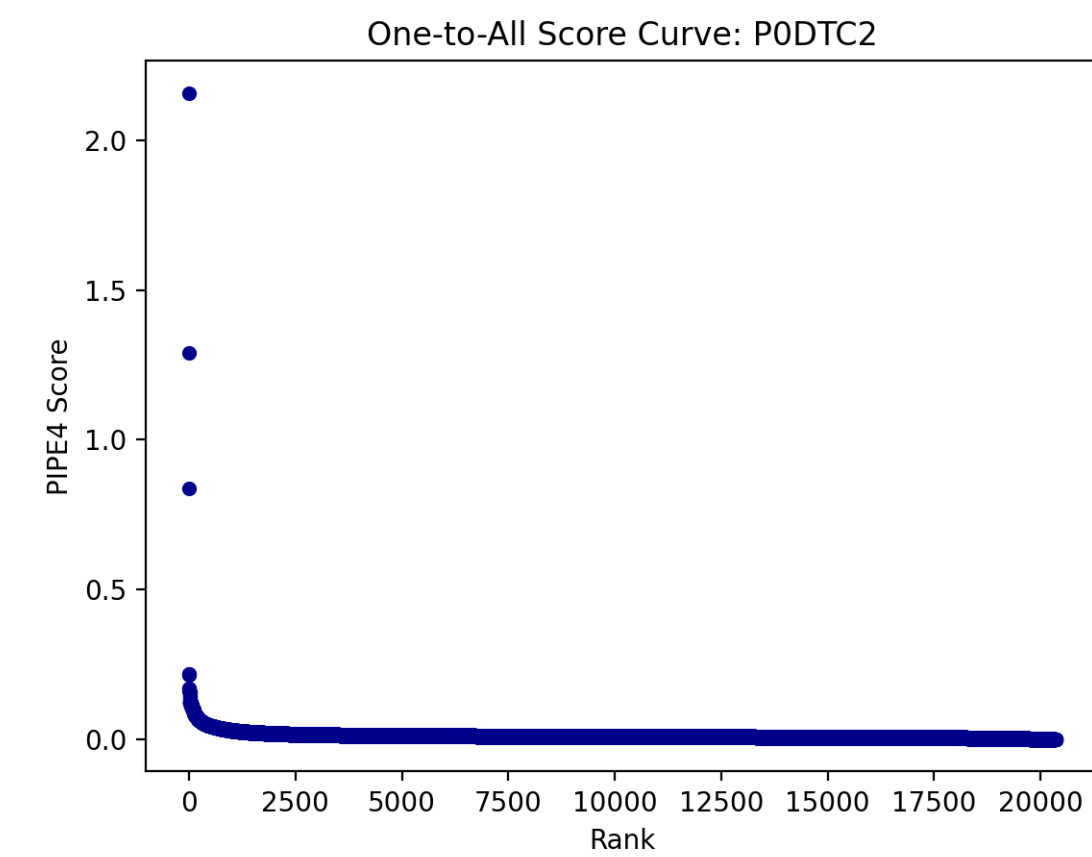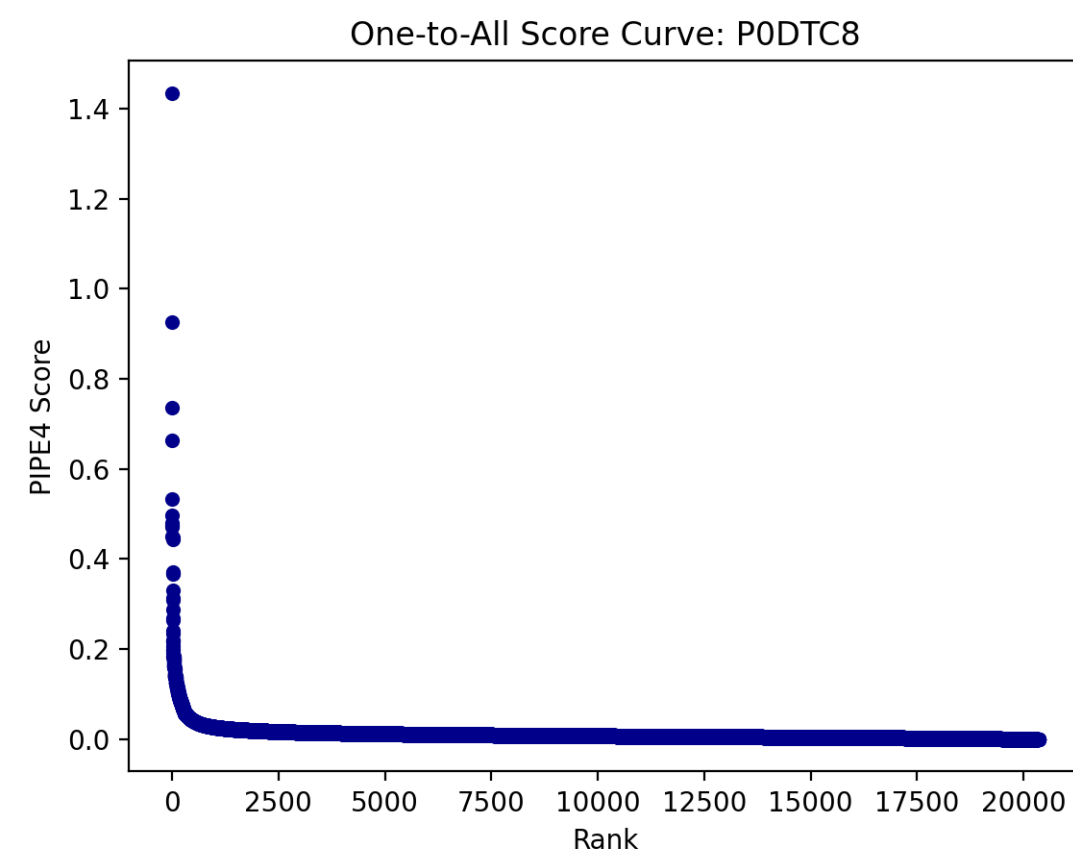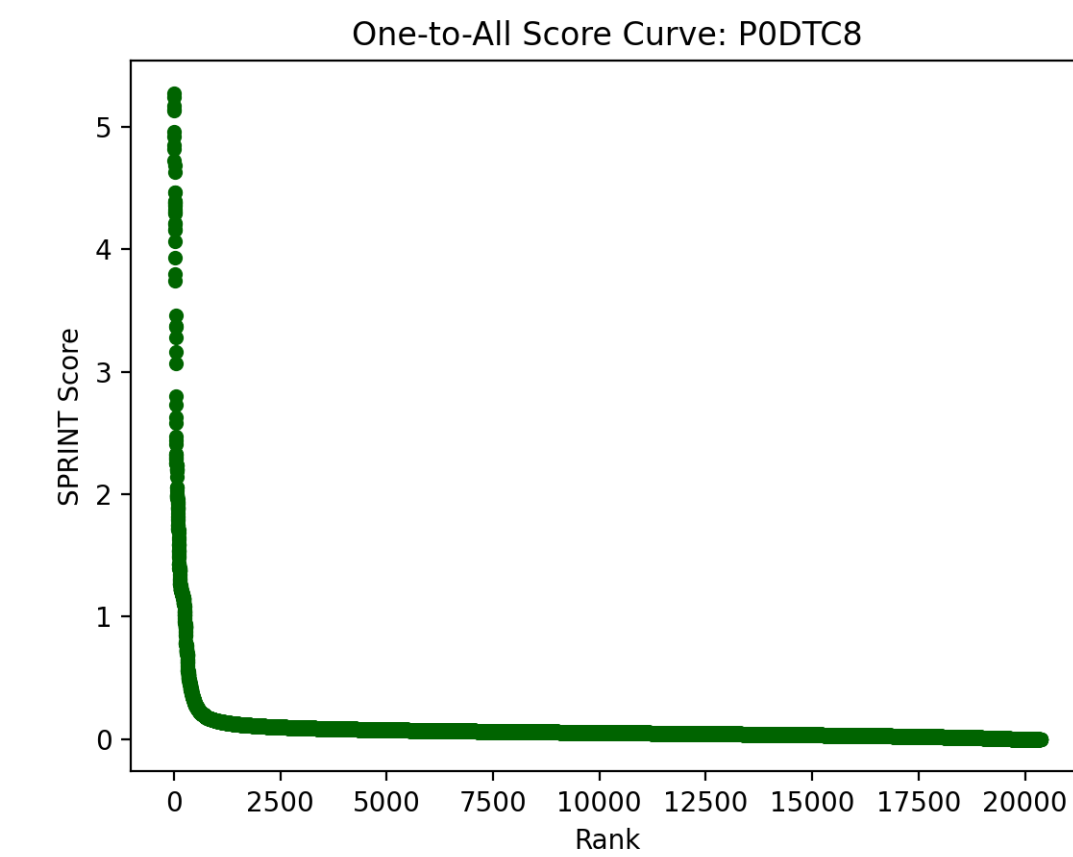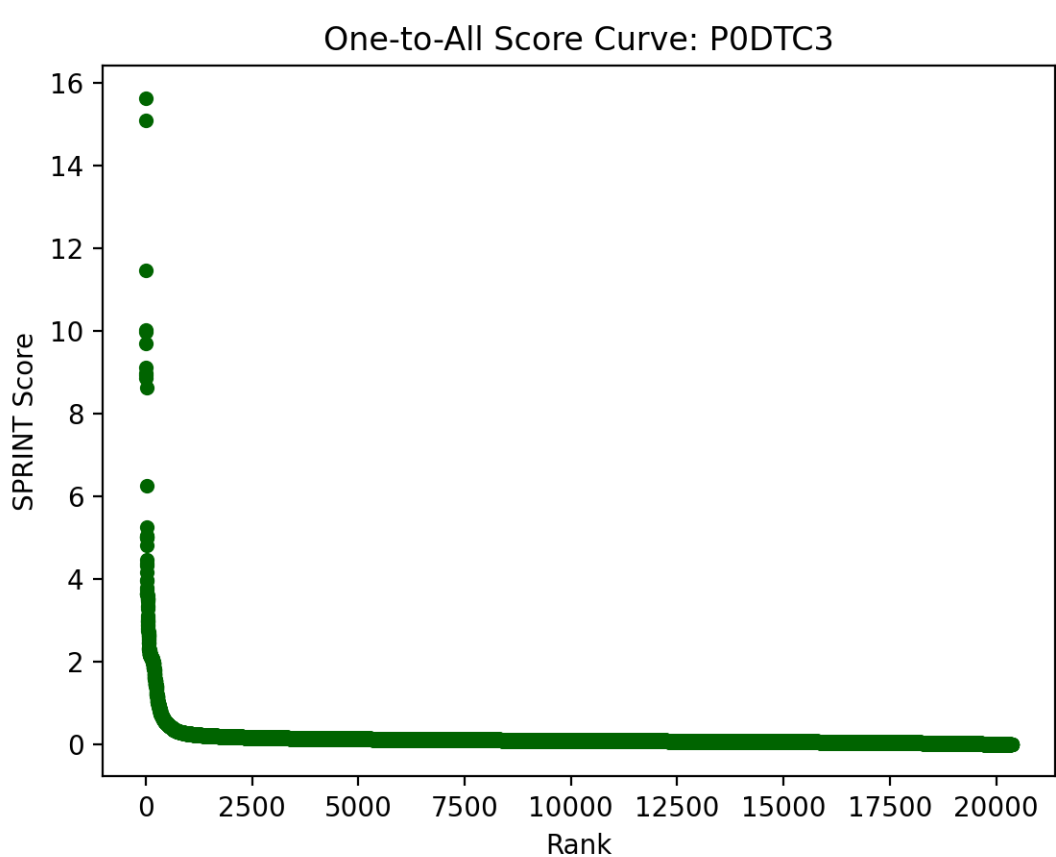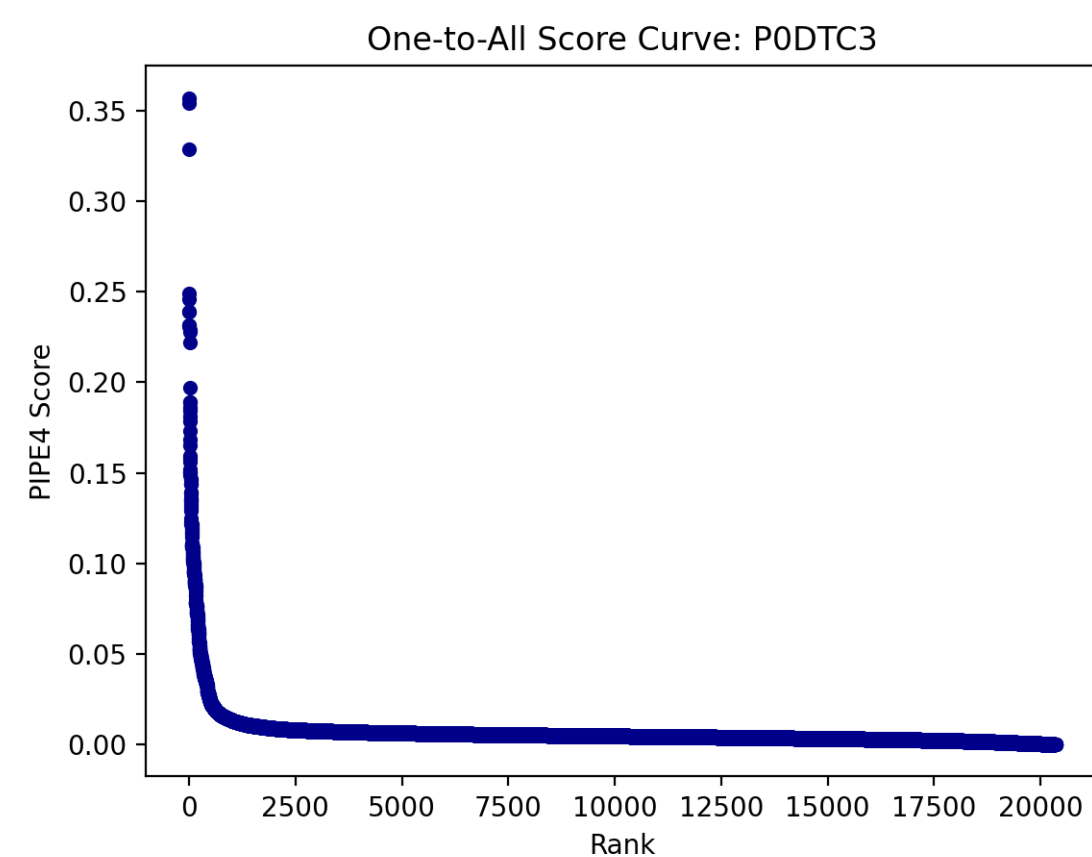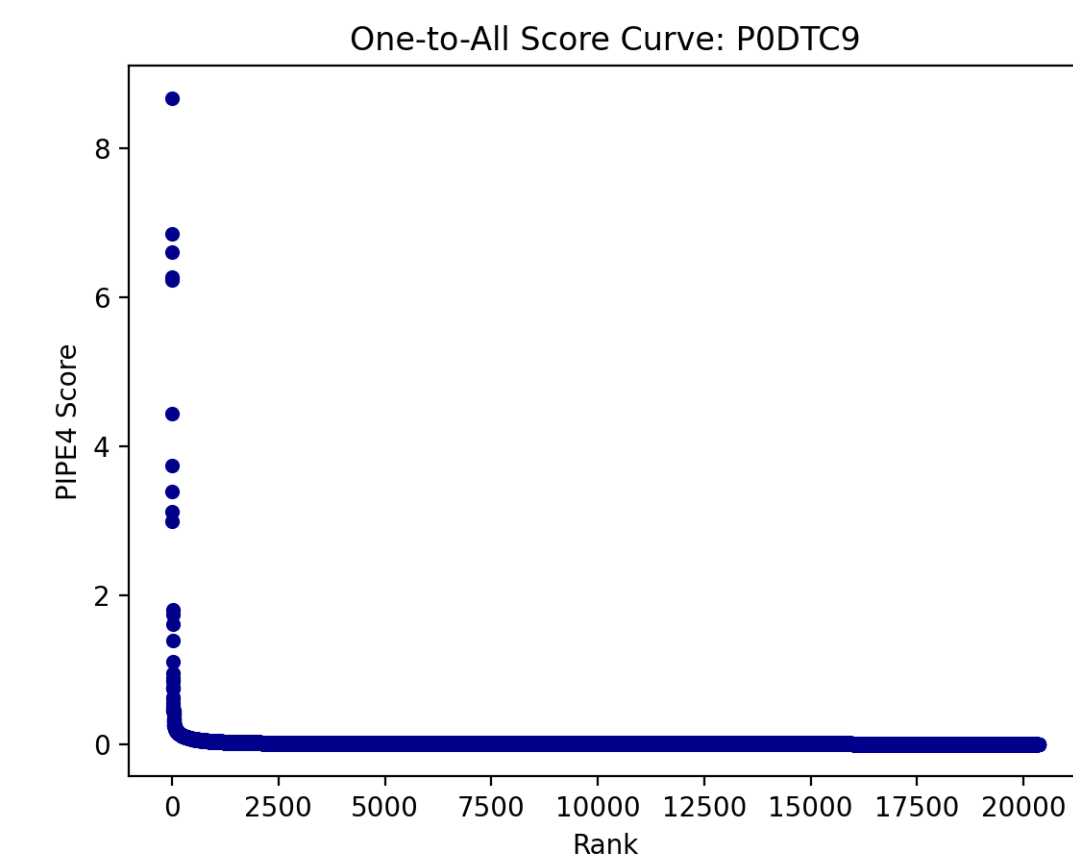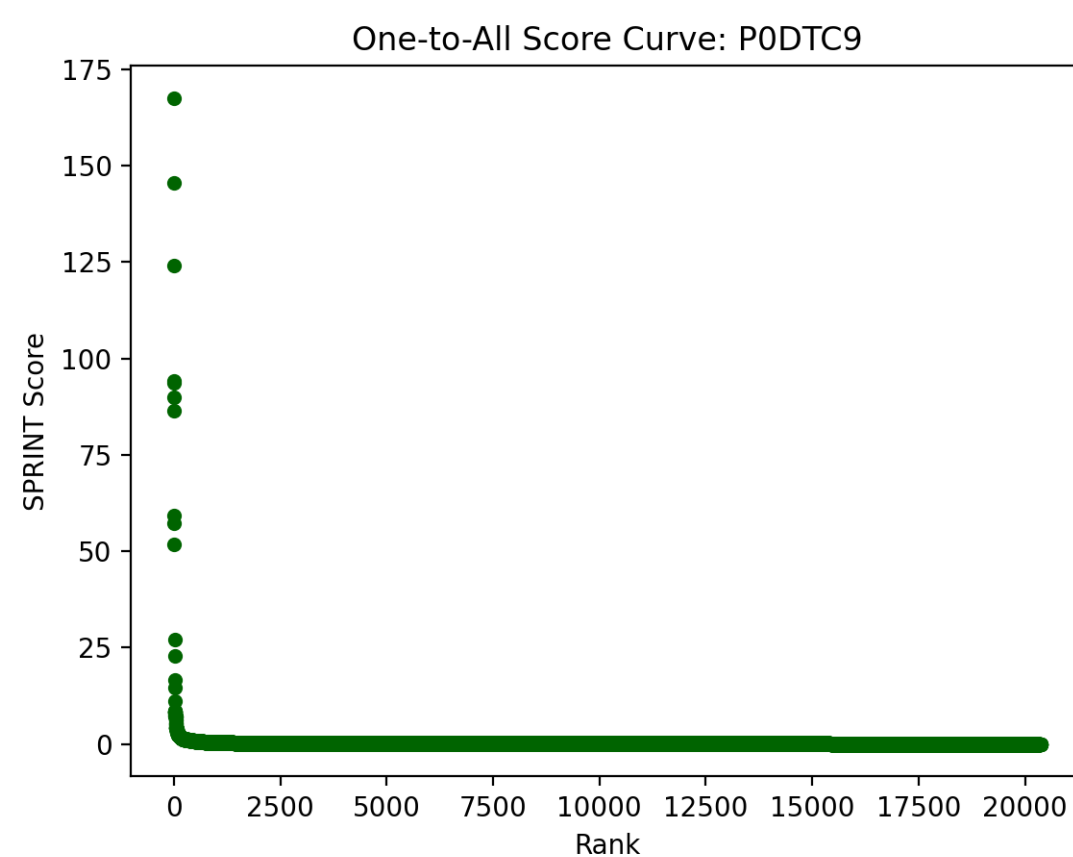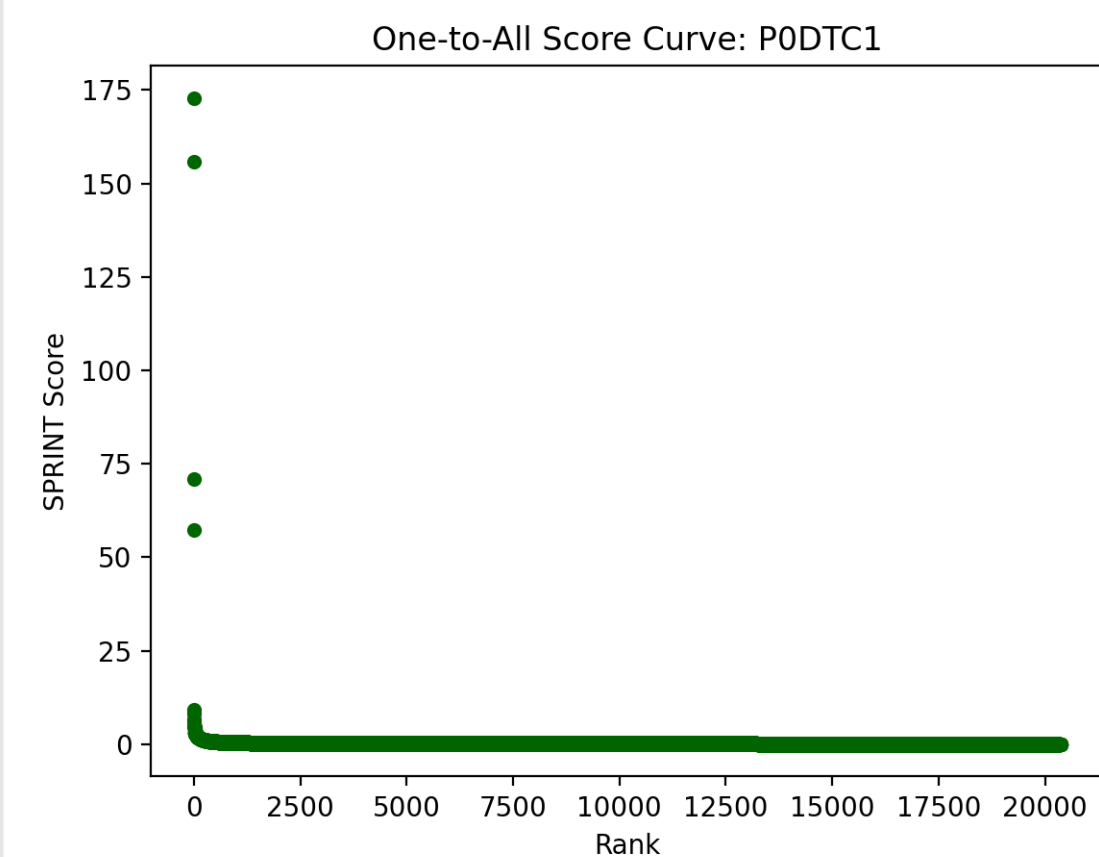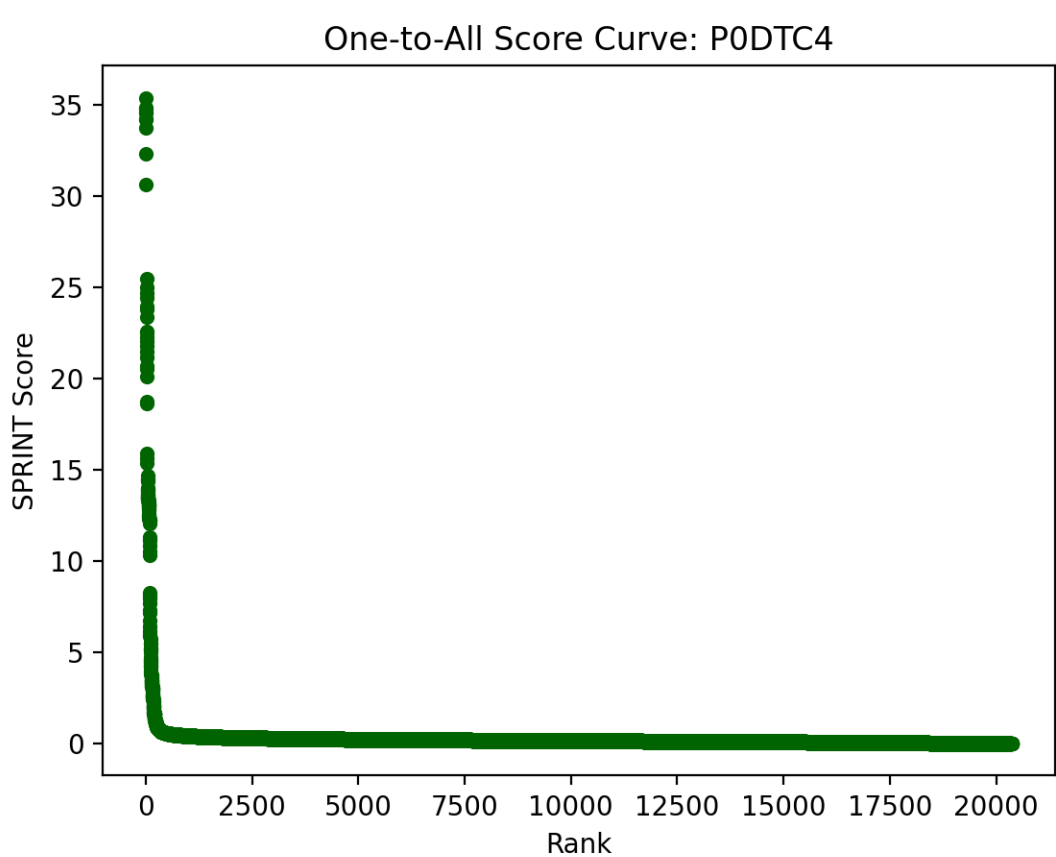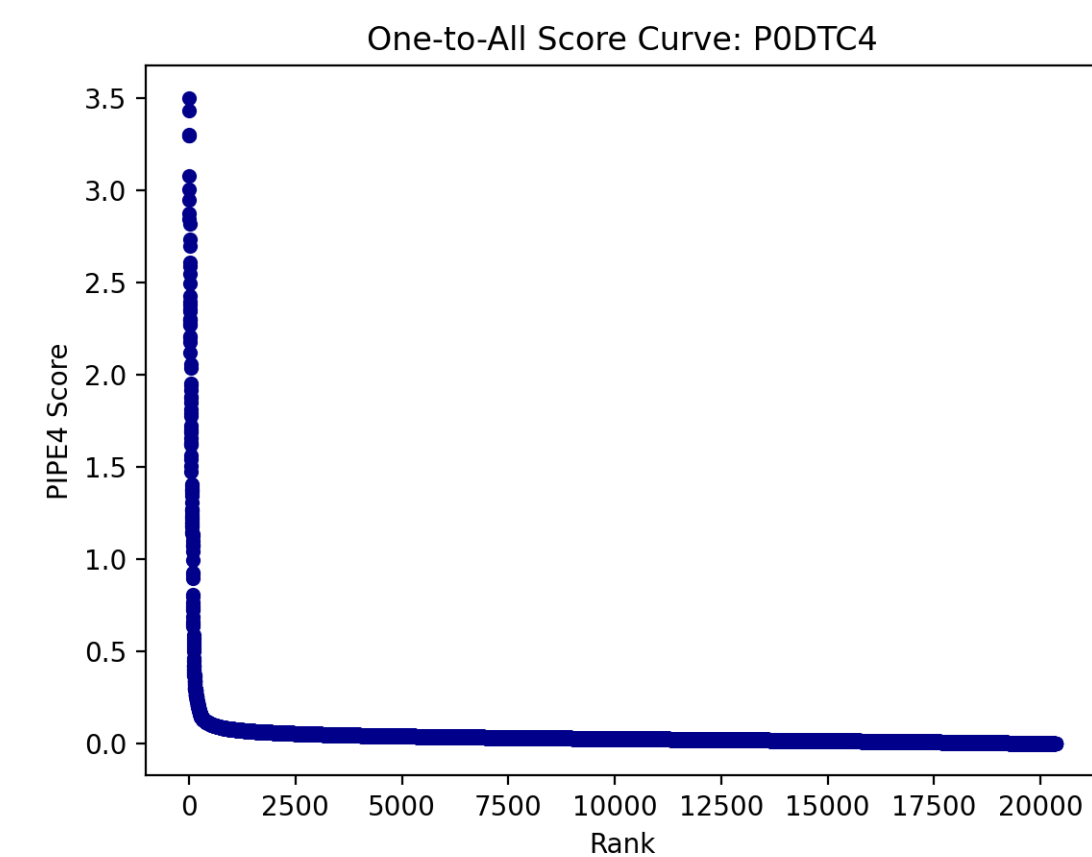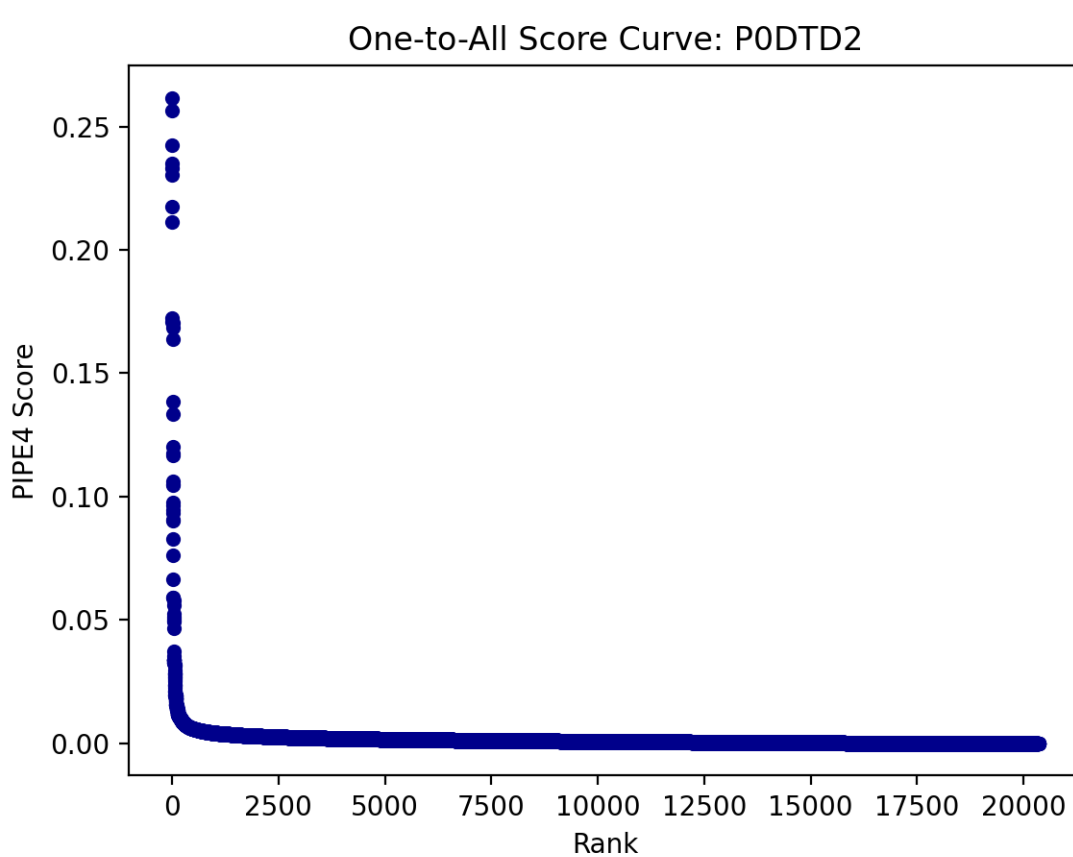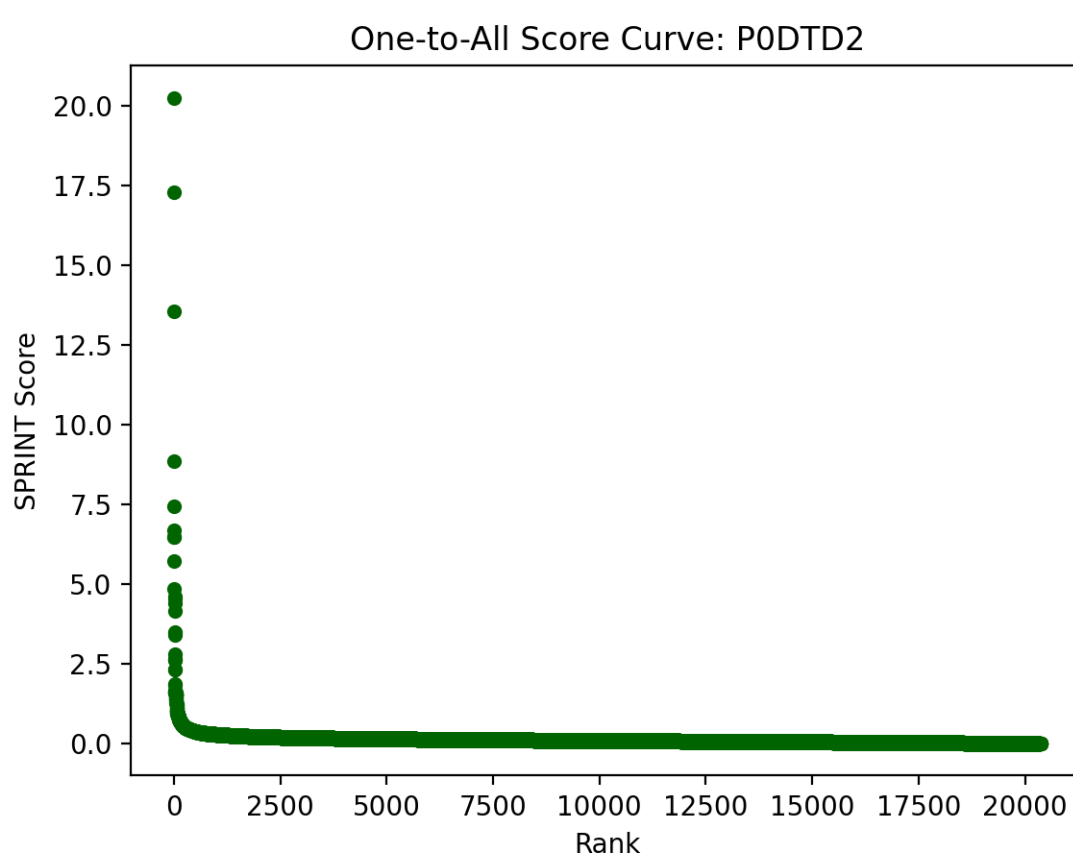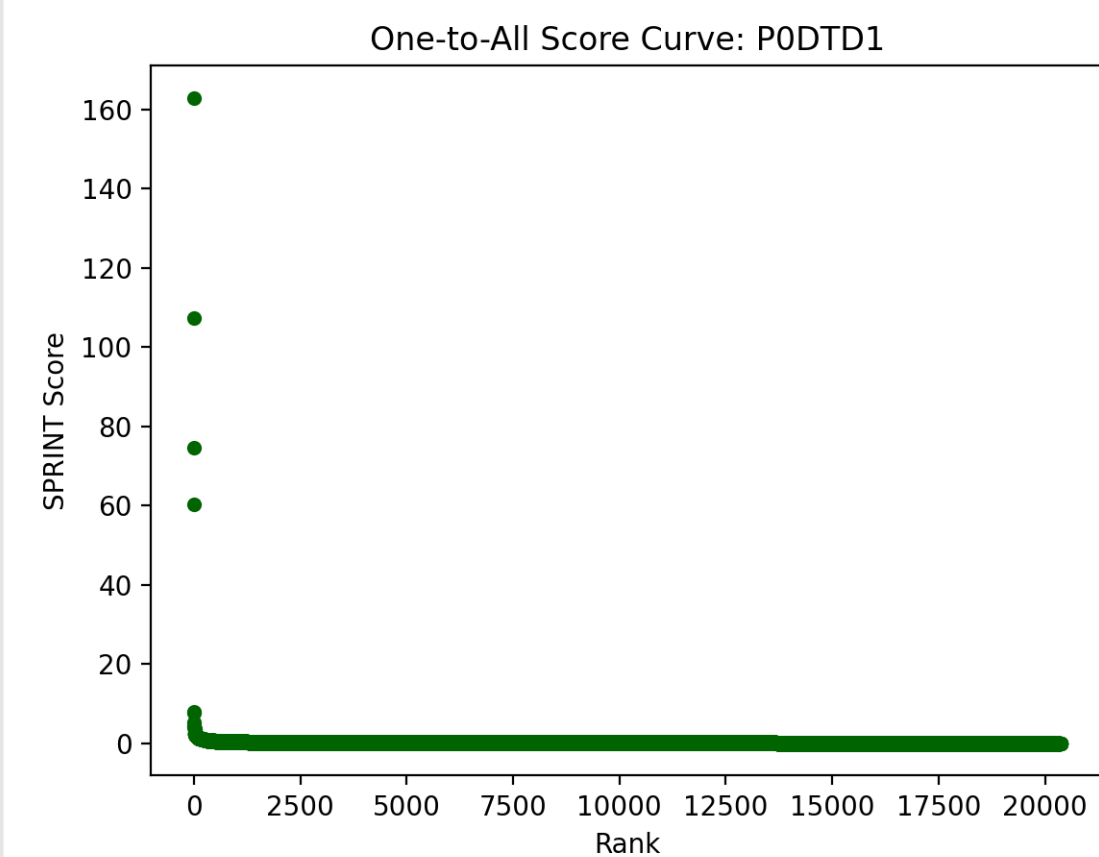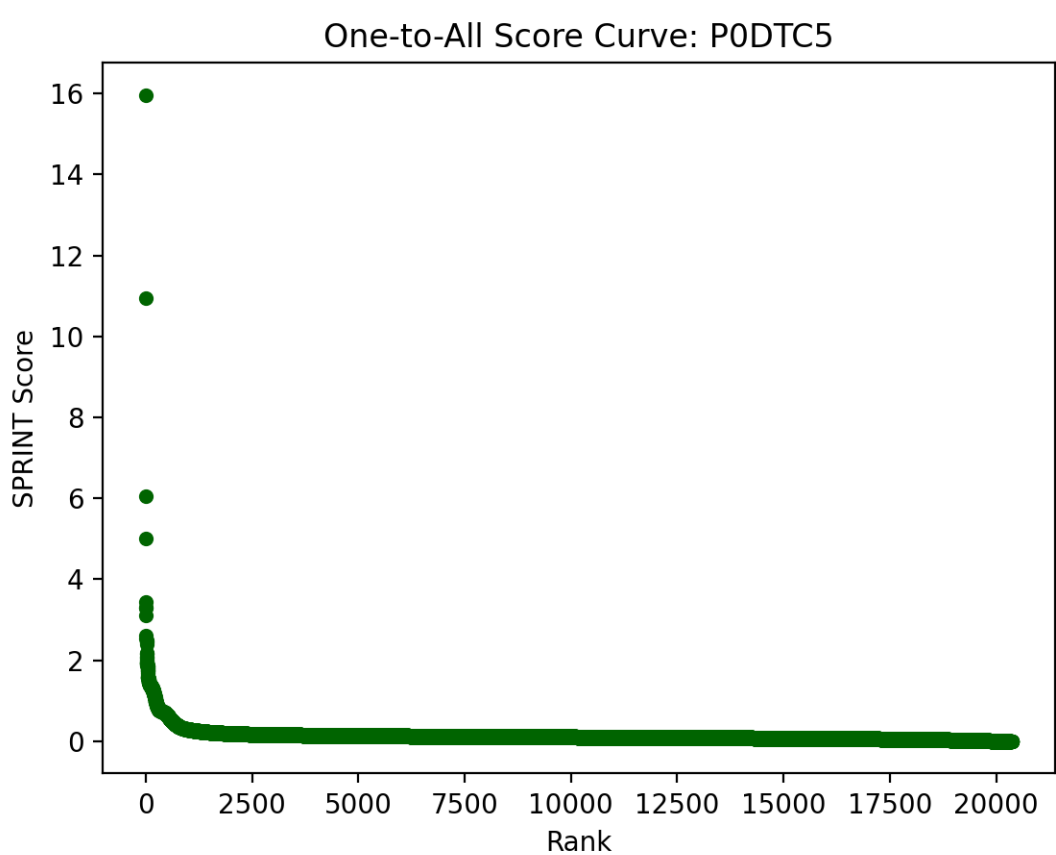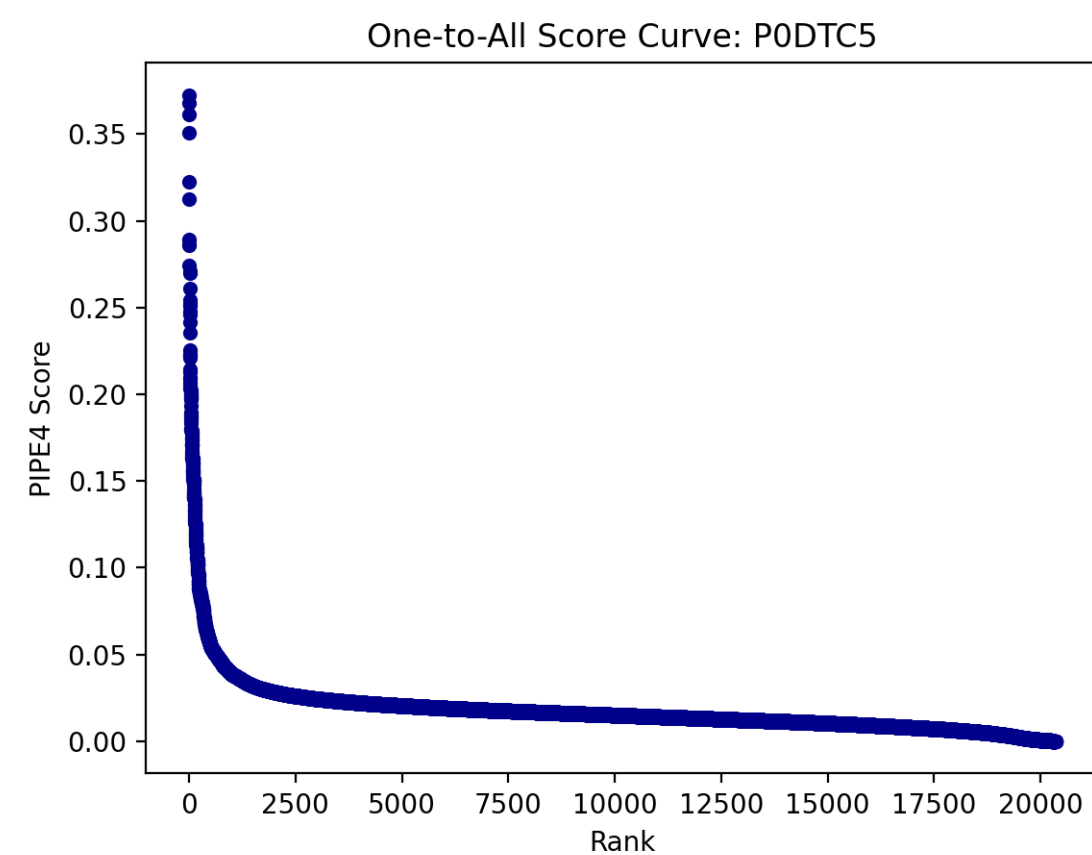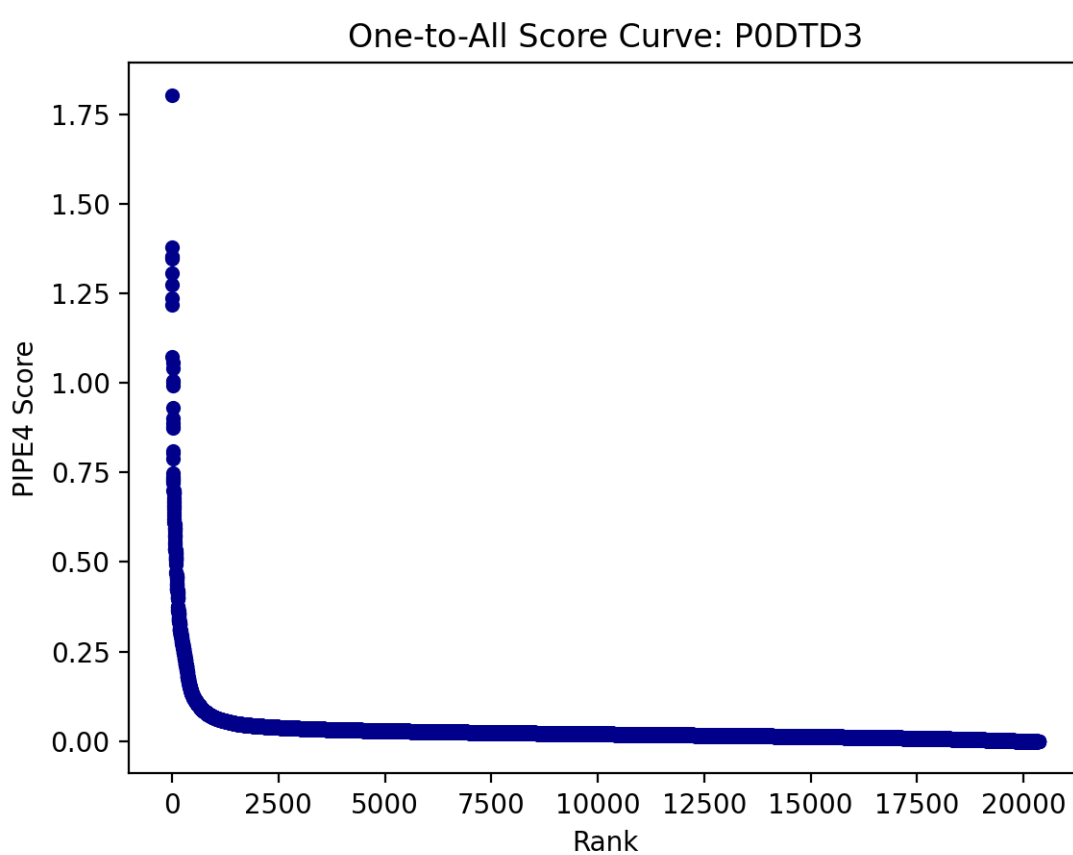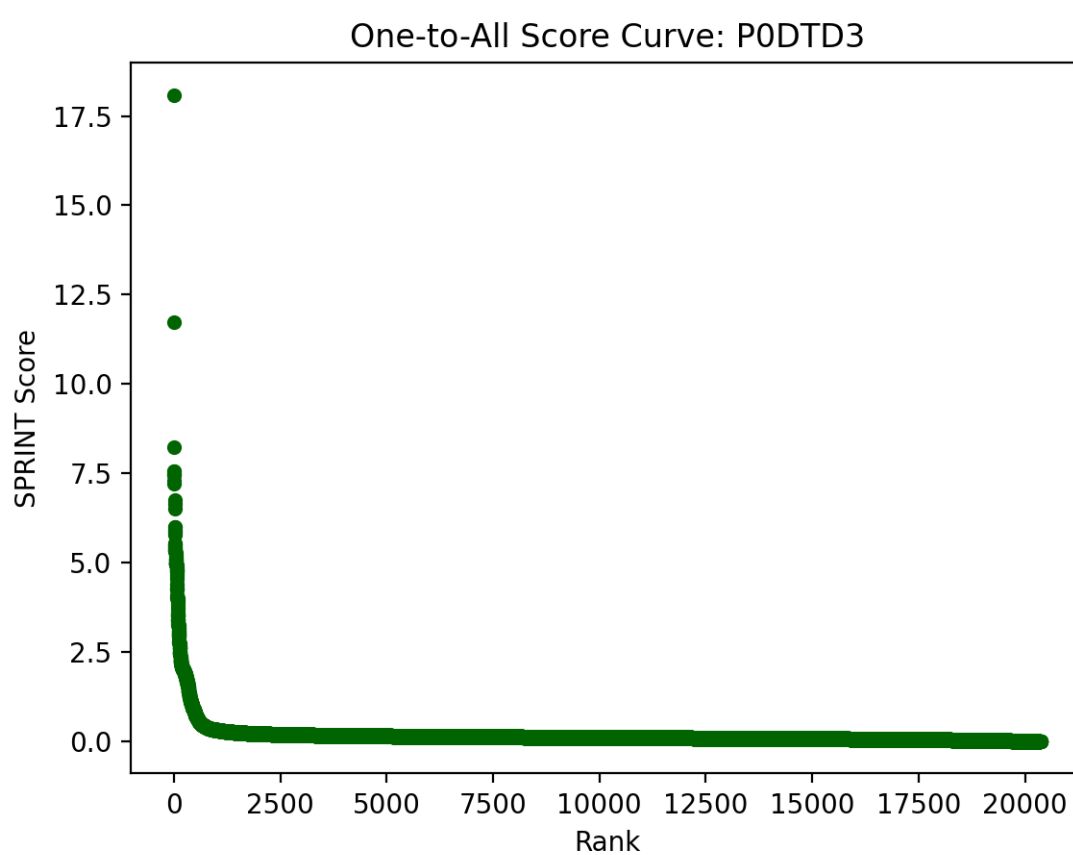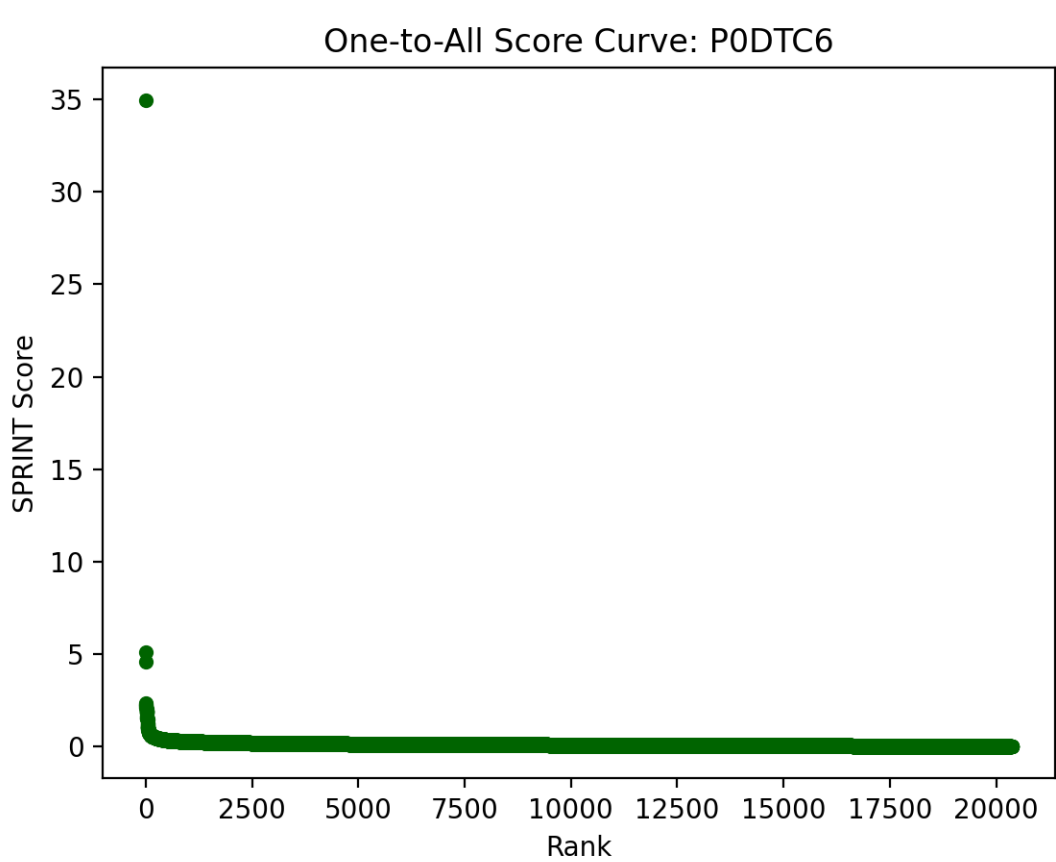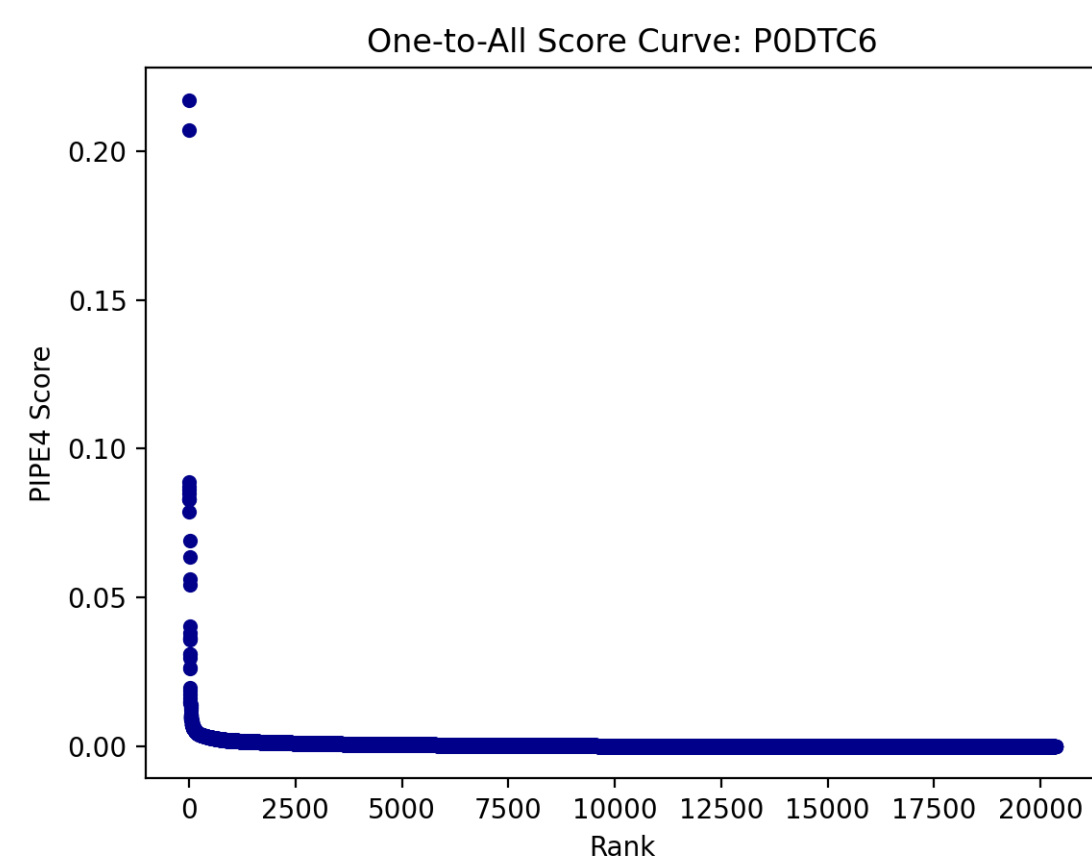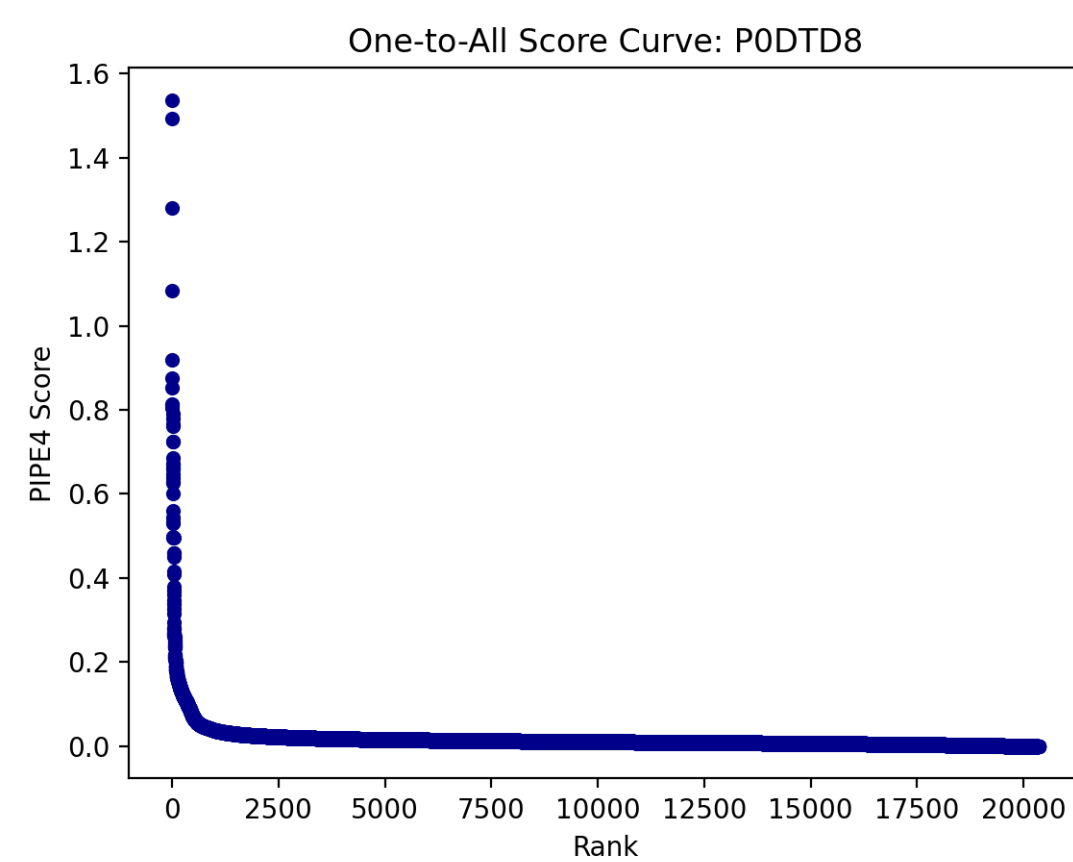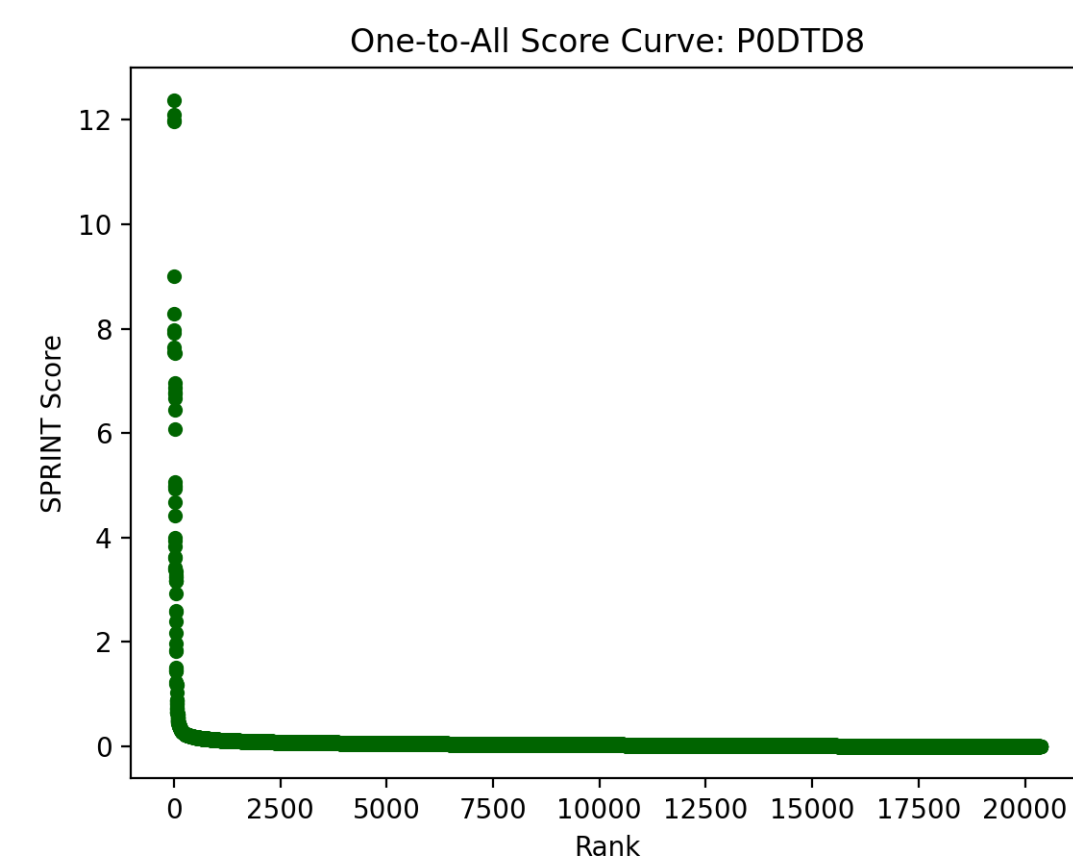

Supplement: Supplemental Information 8 — Each of the subplots depicts a characteristic “L”-shape, where there are a relatively small number of high-scoring pairs as compared to a large number of low-scoring pairs within the baseline. Note that the y-axes are not shared among subplots. [file peerj-09-11117-s008.pdf]

# SPRINT

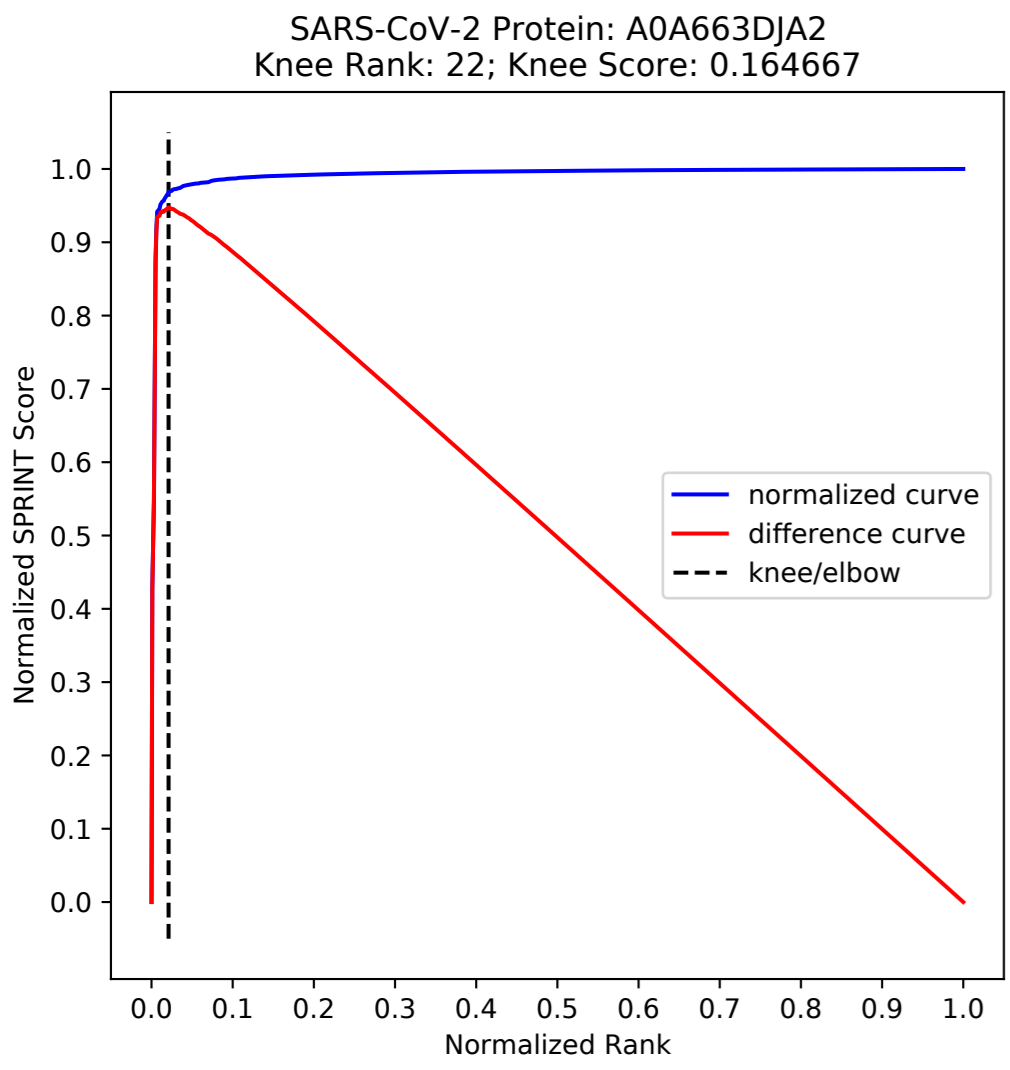

# PIPE4

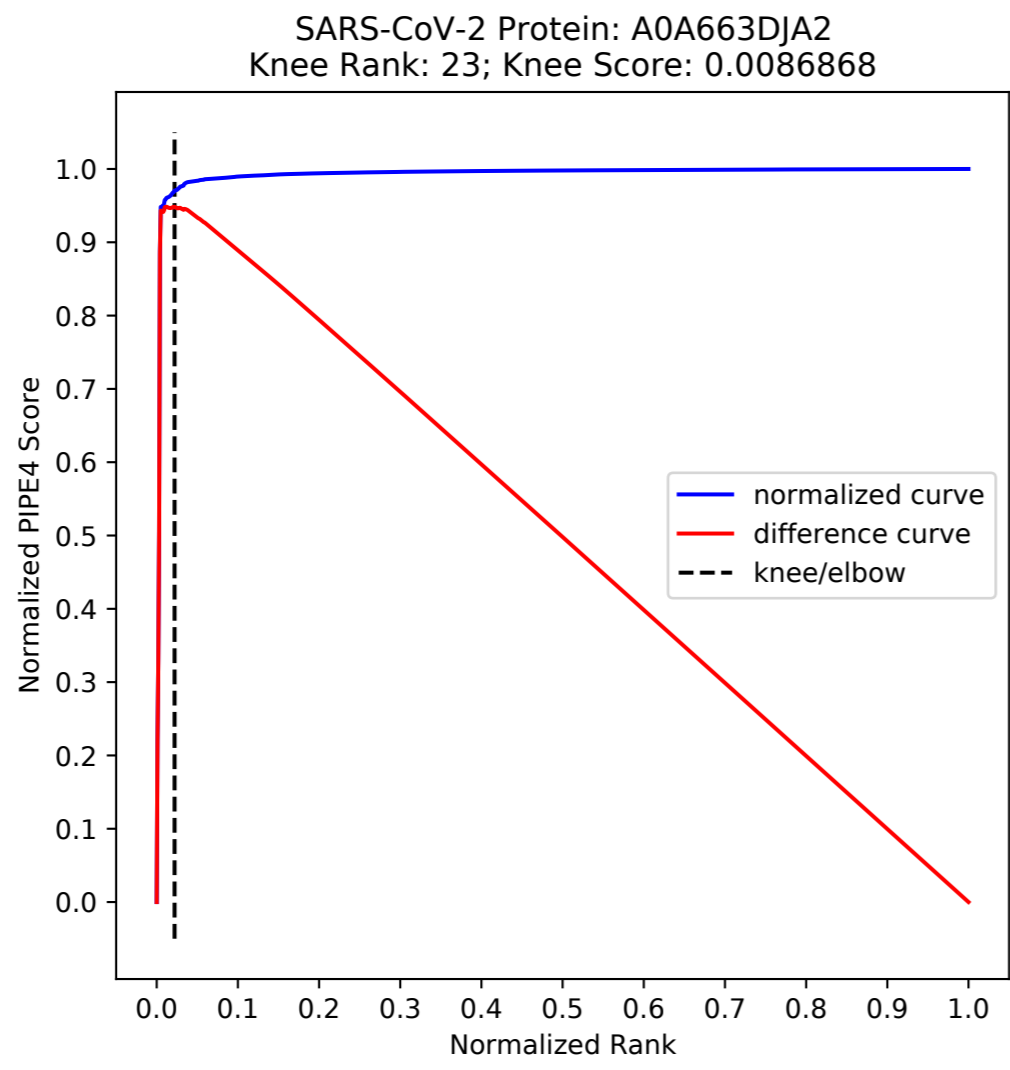

# PIPE4

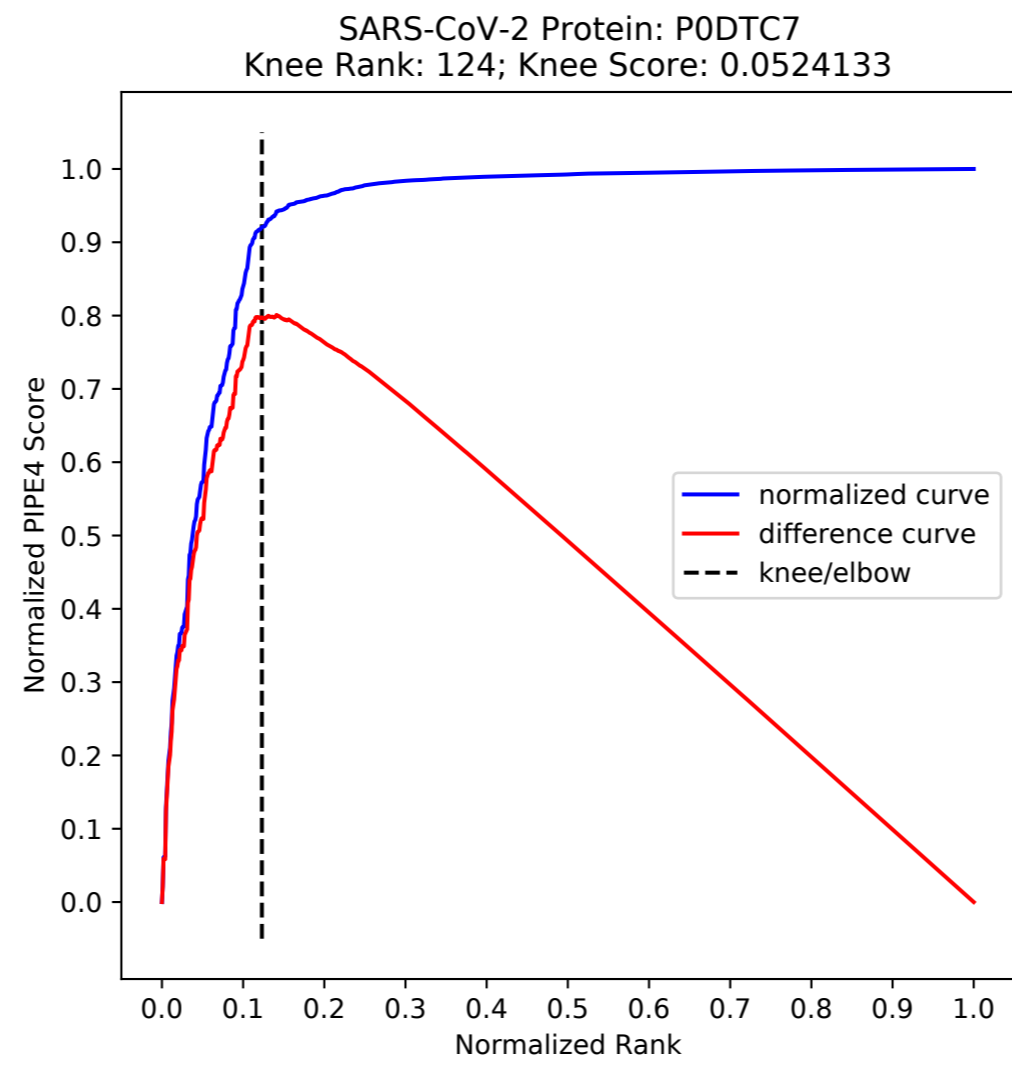

# SPRINT

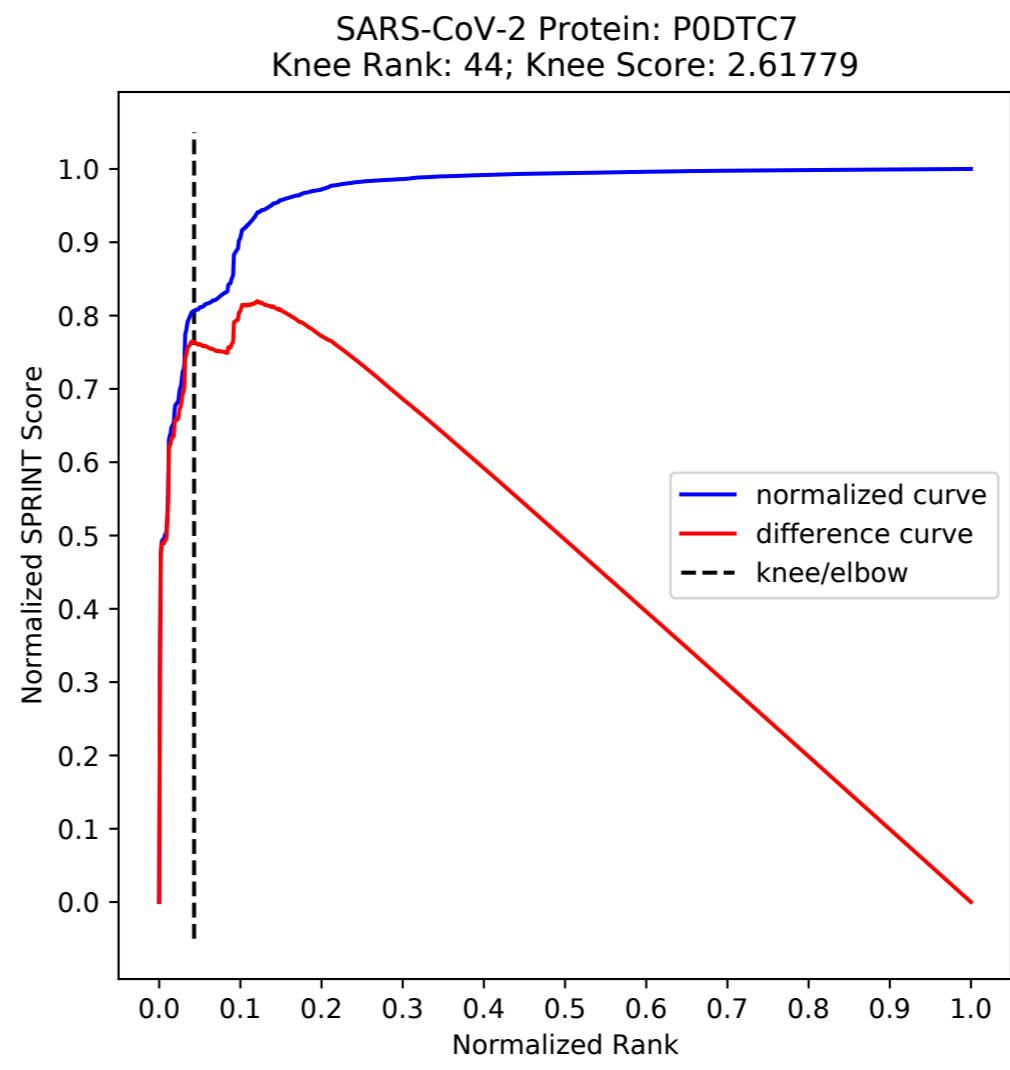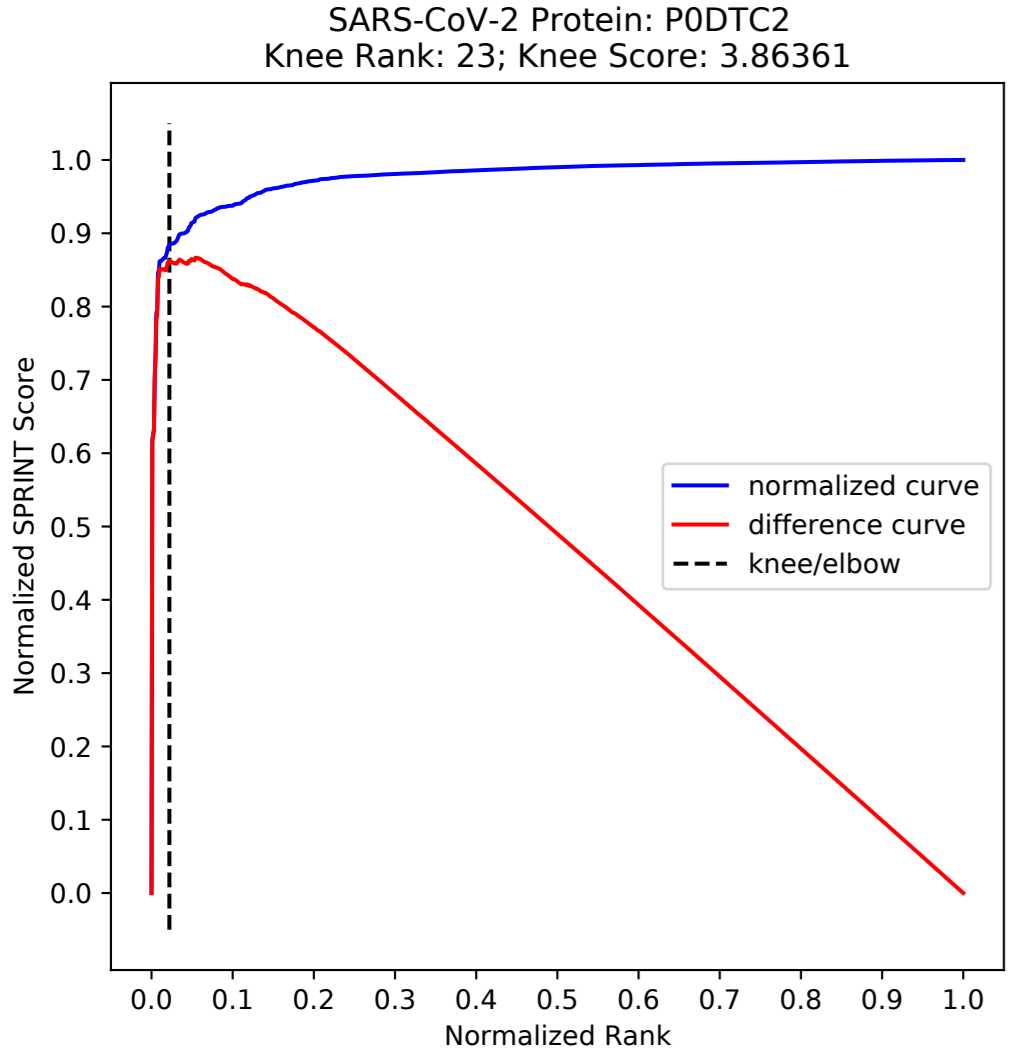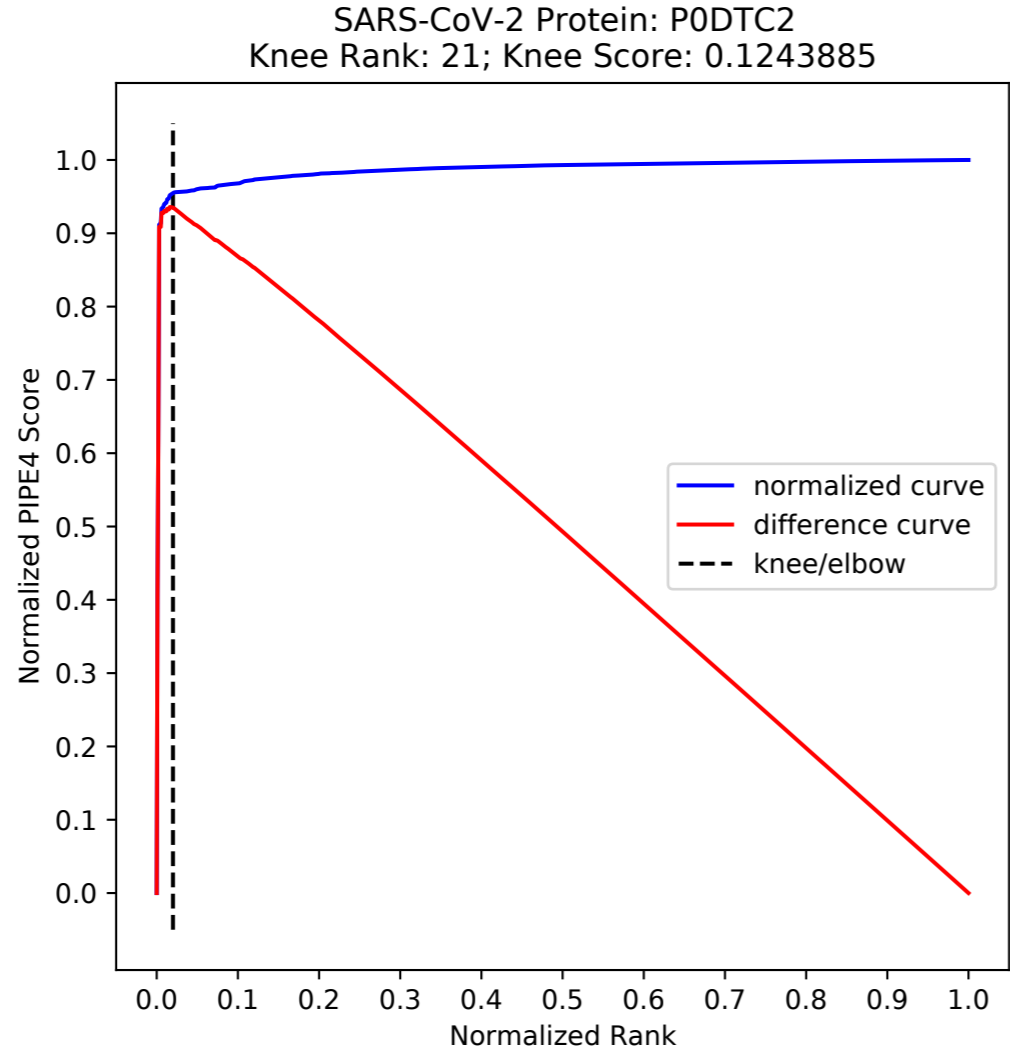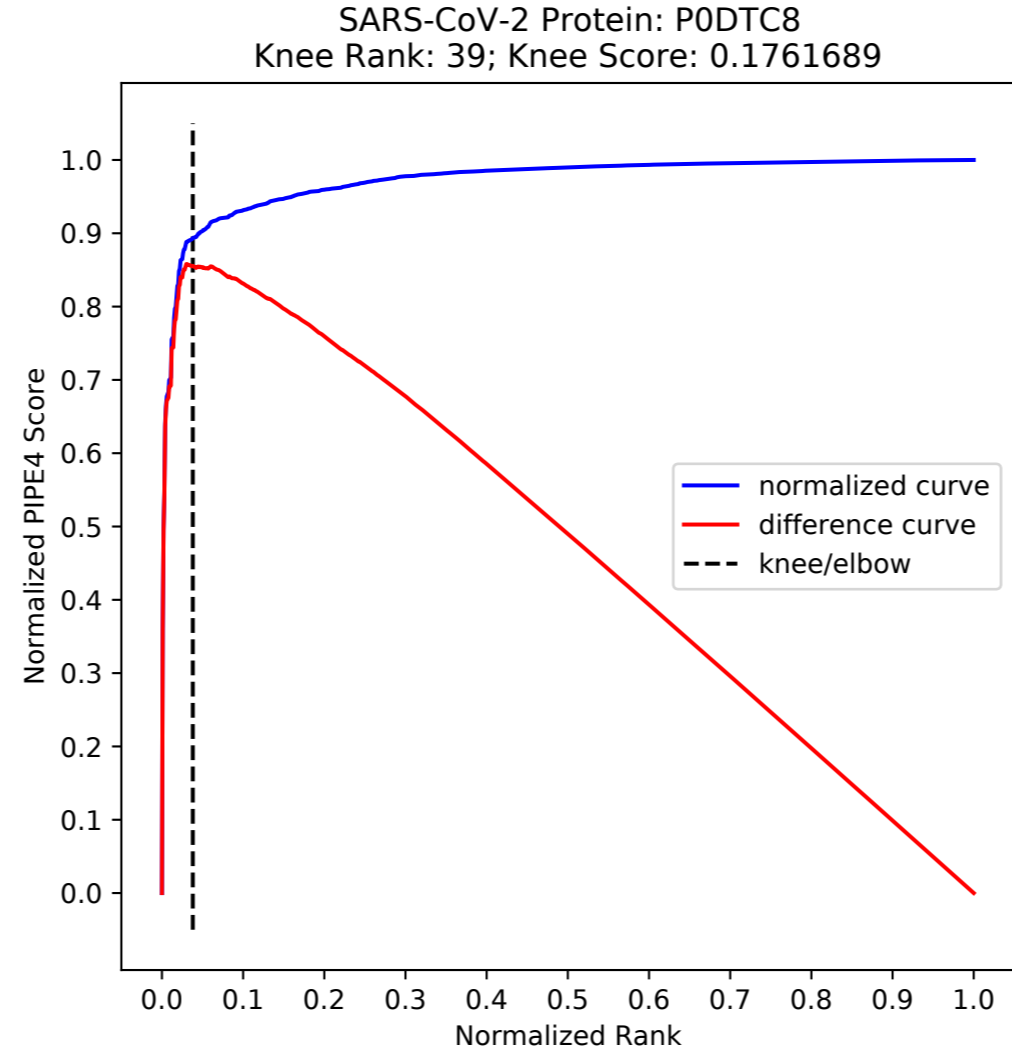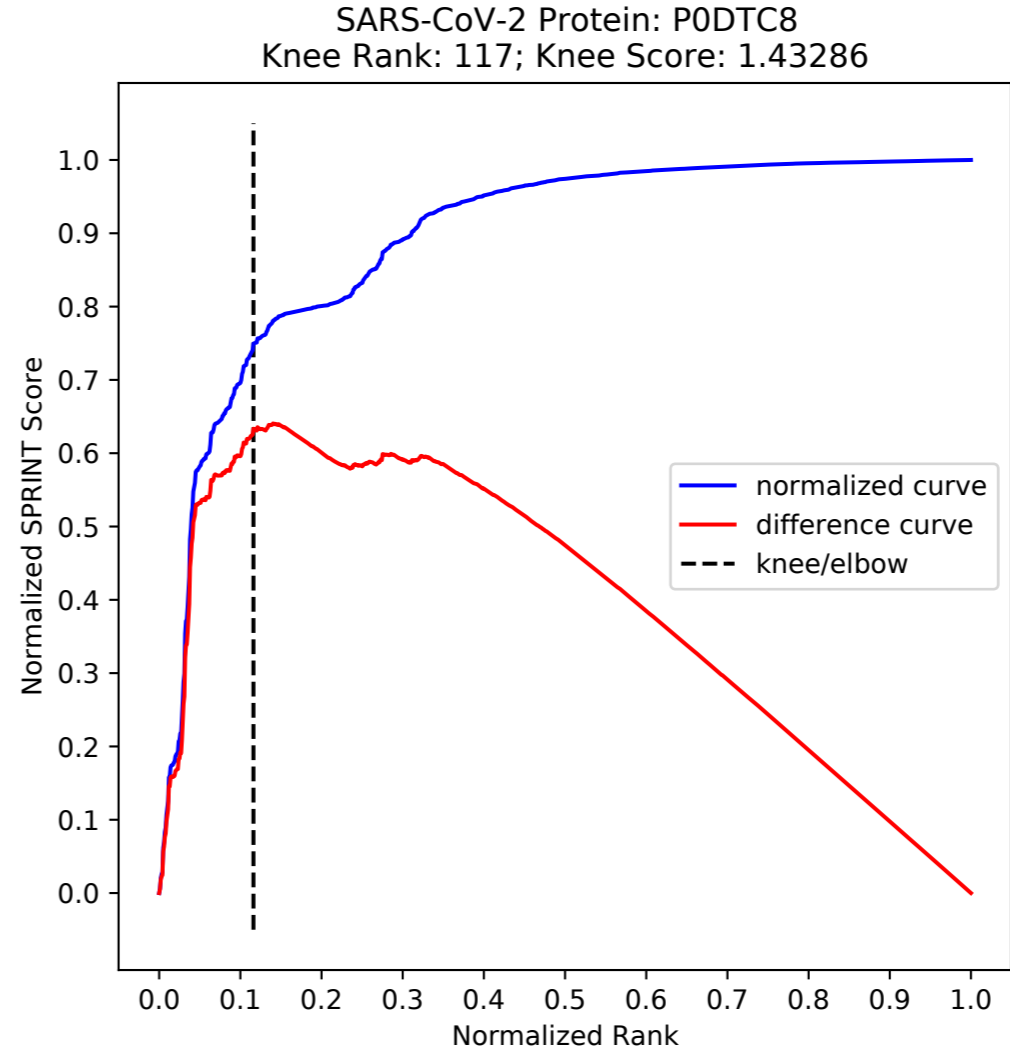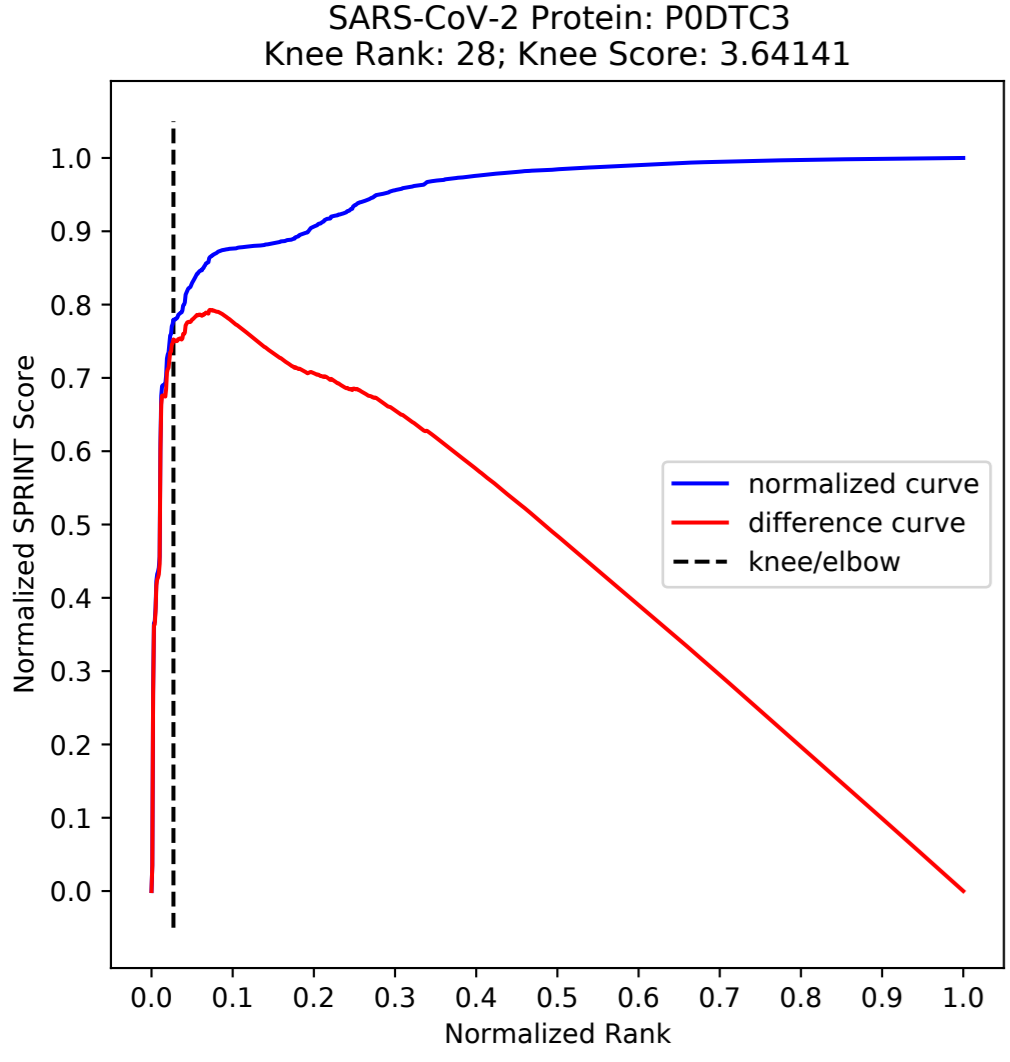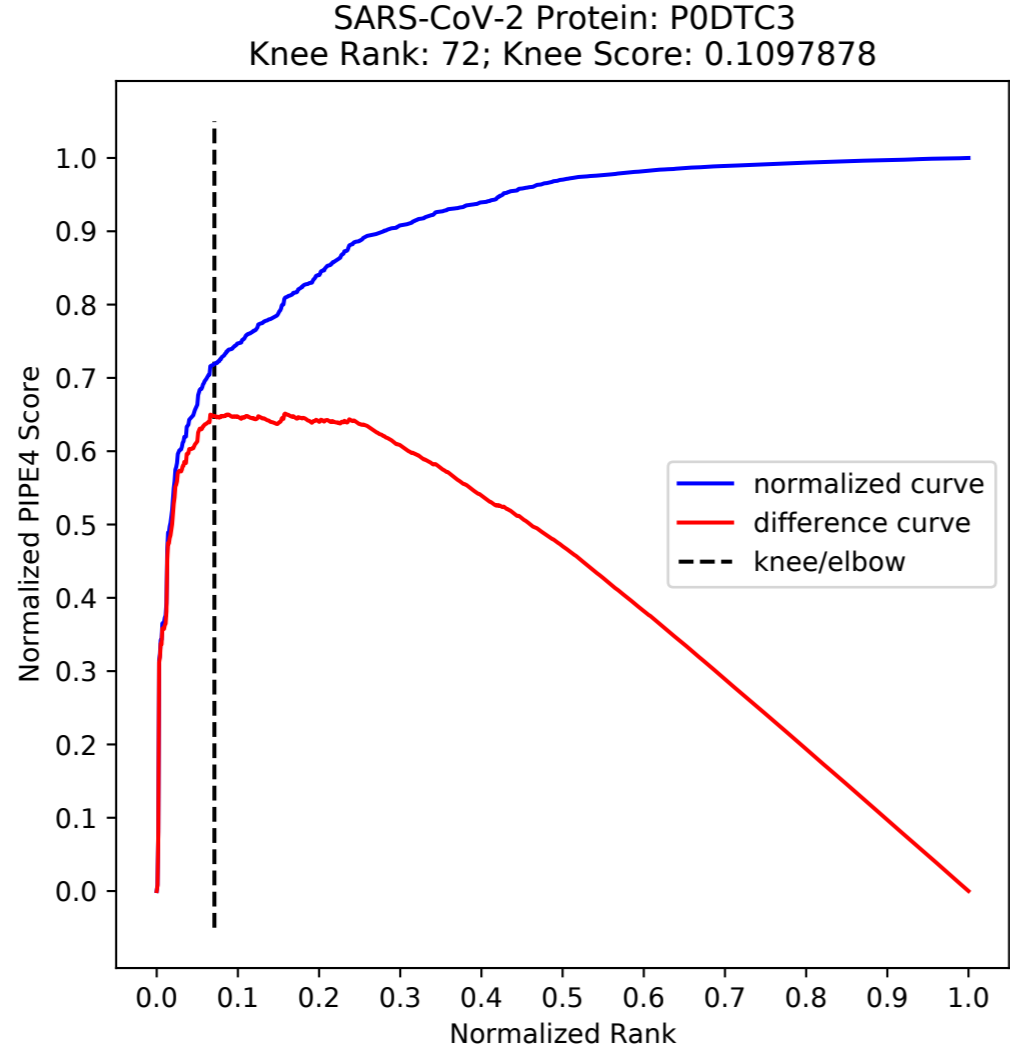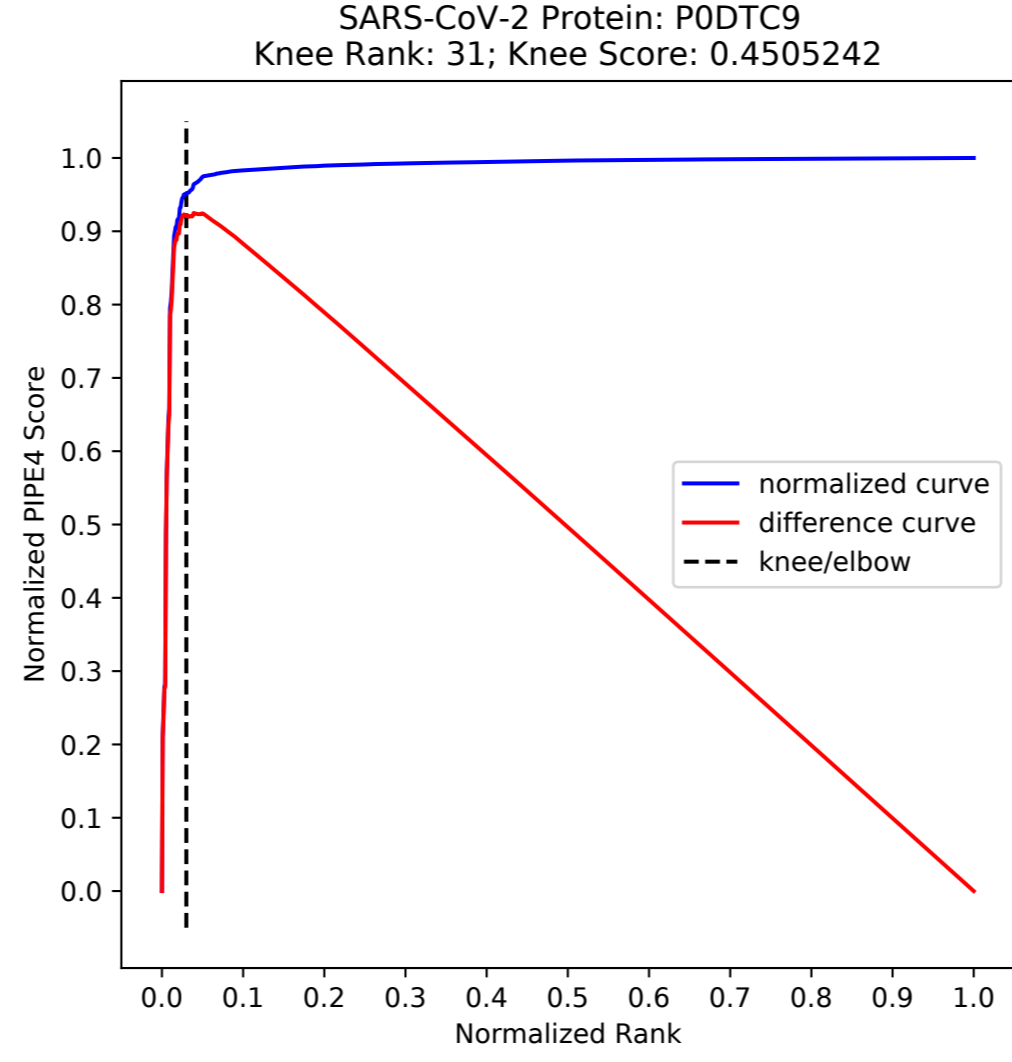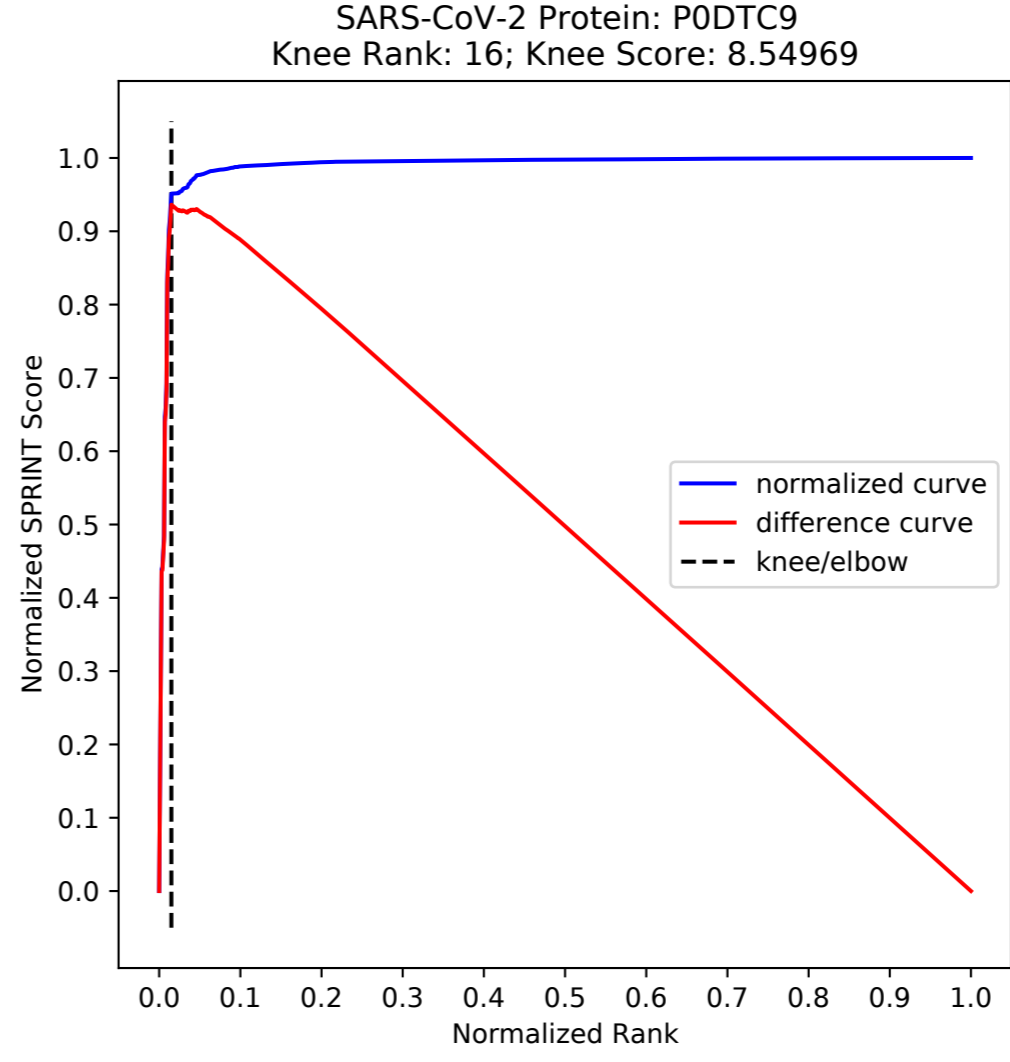

# SPRINT

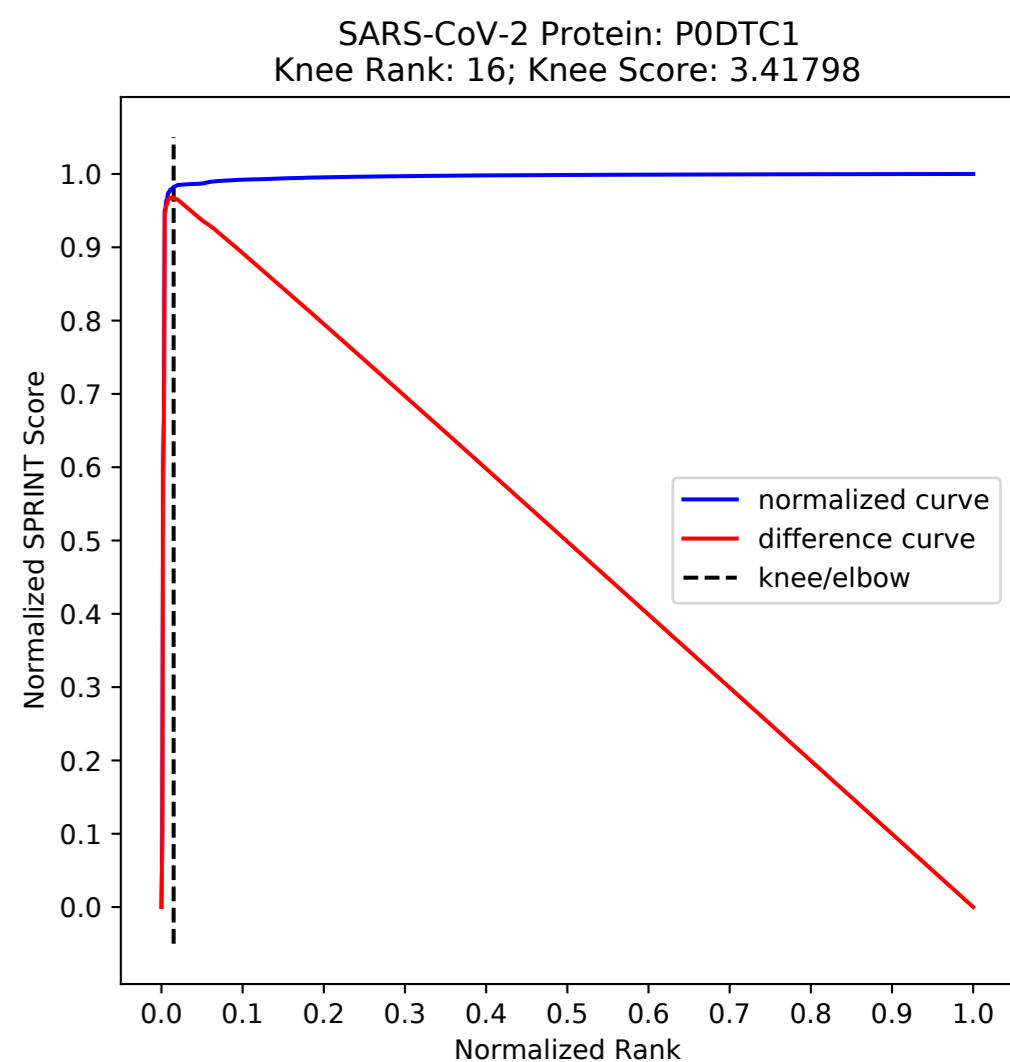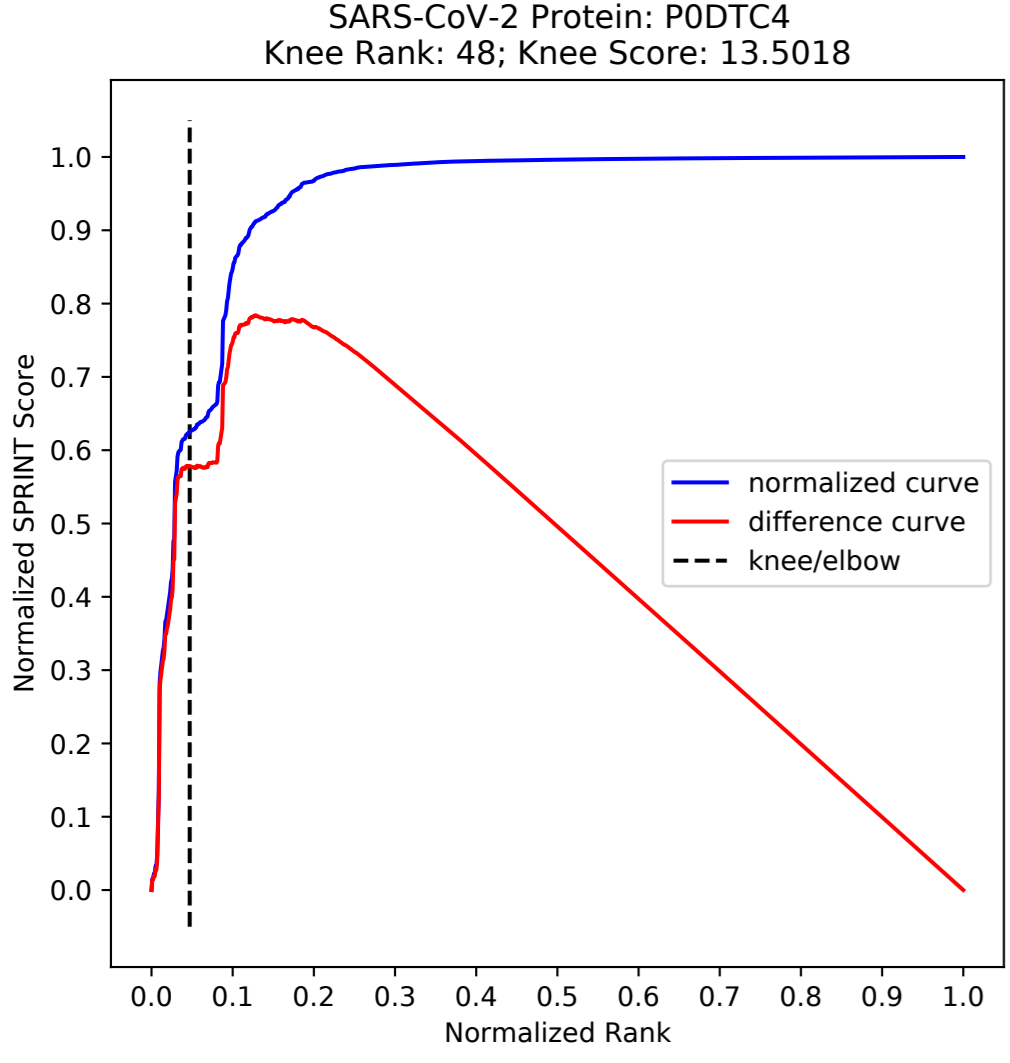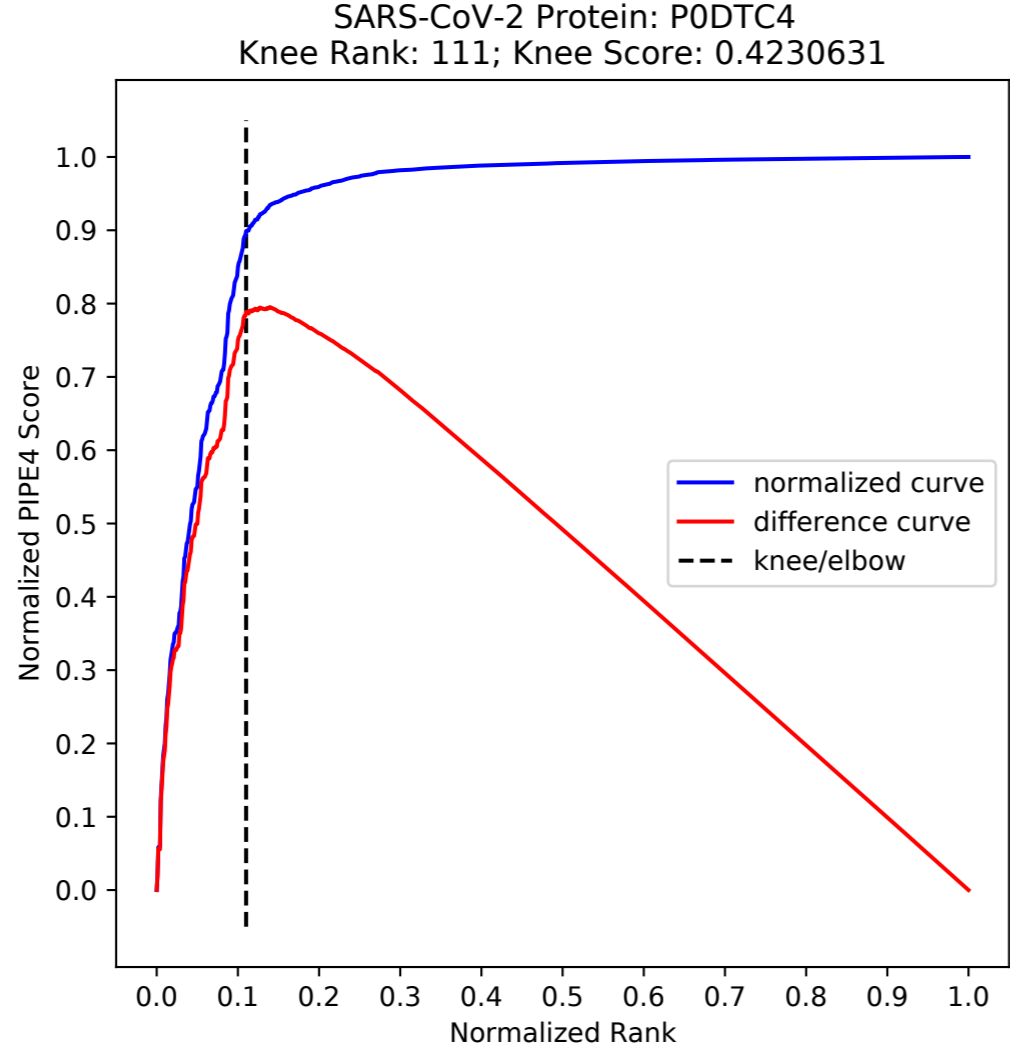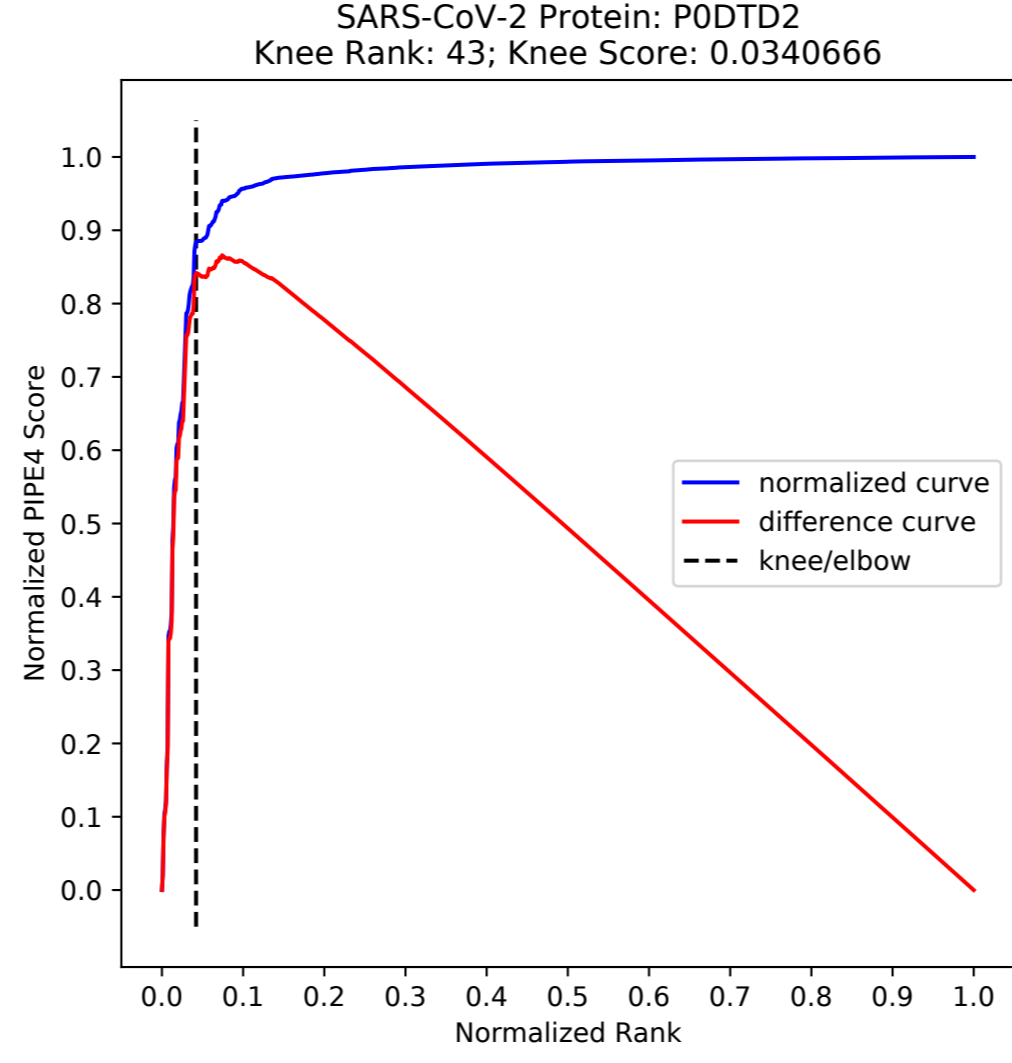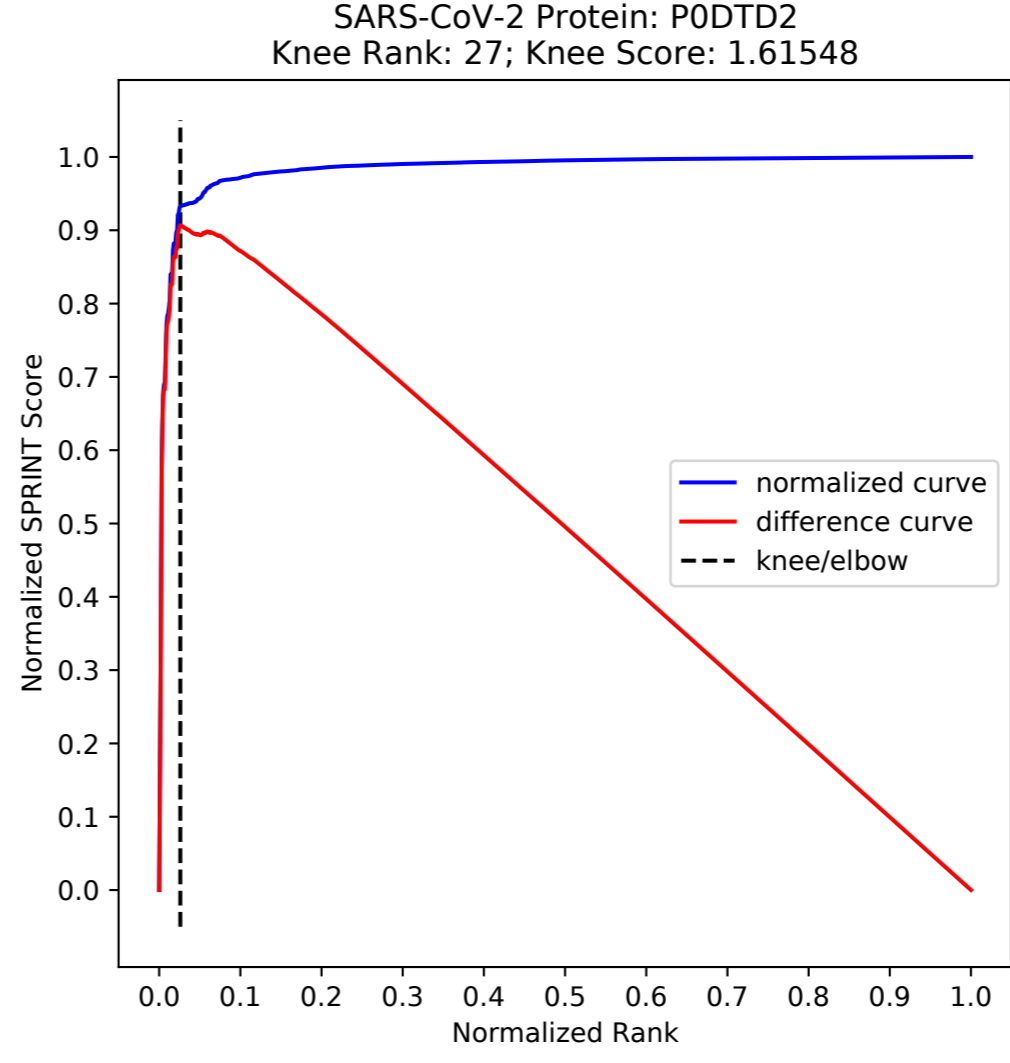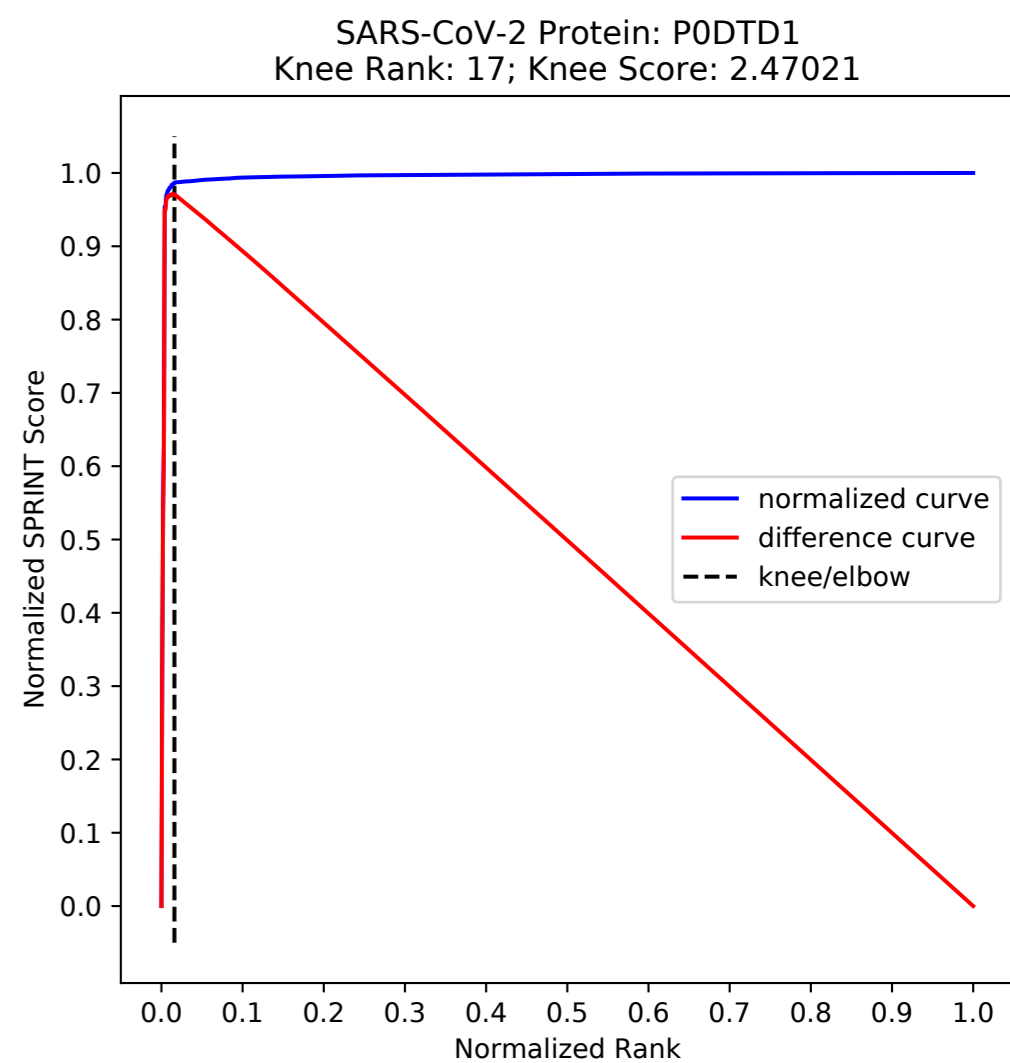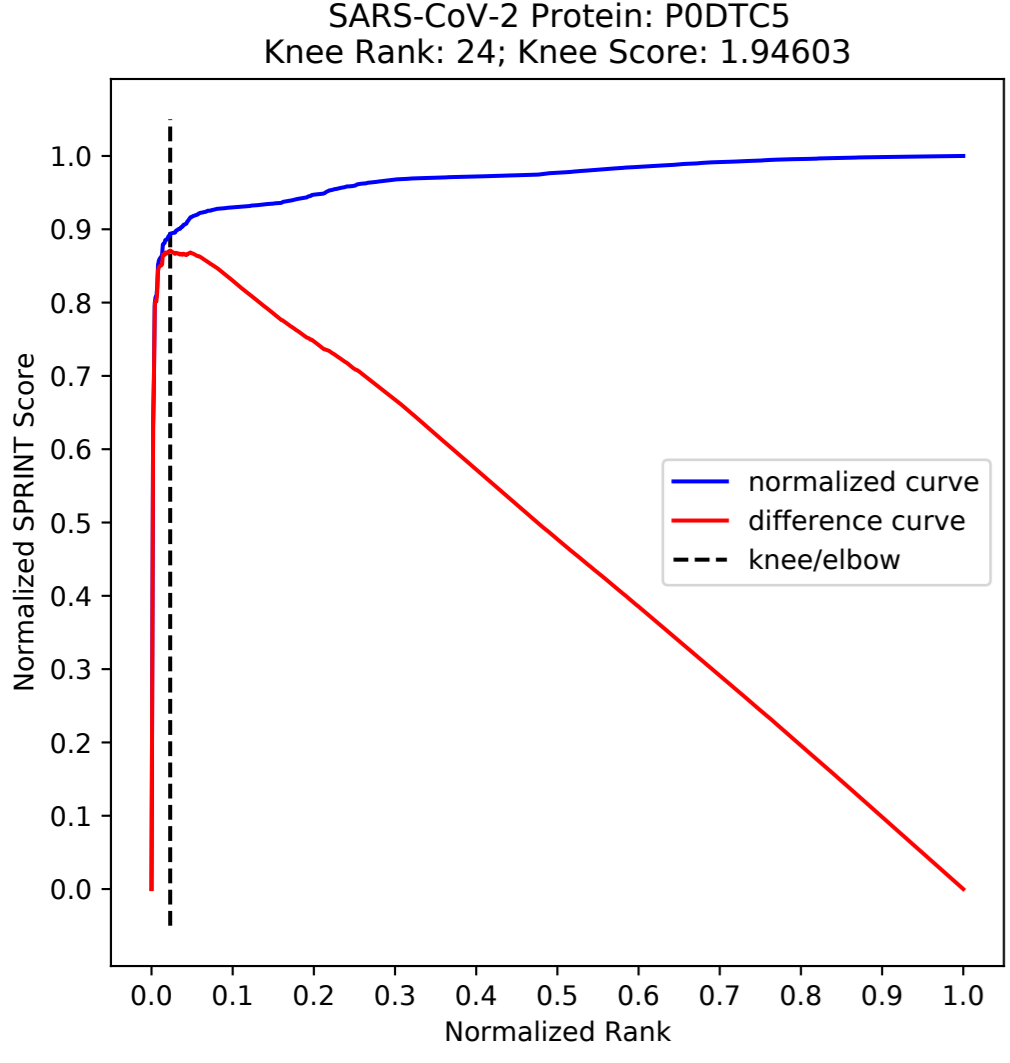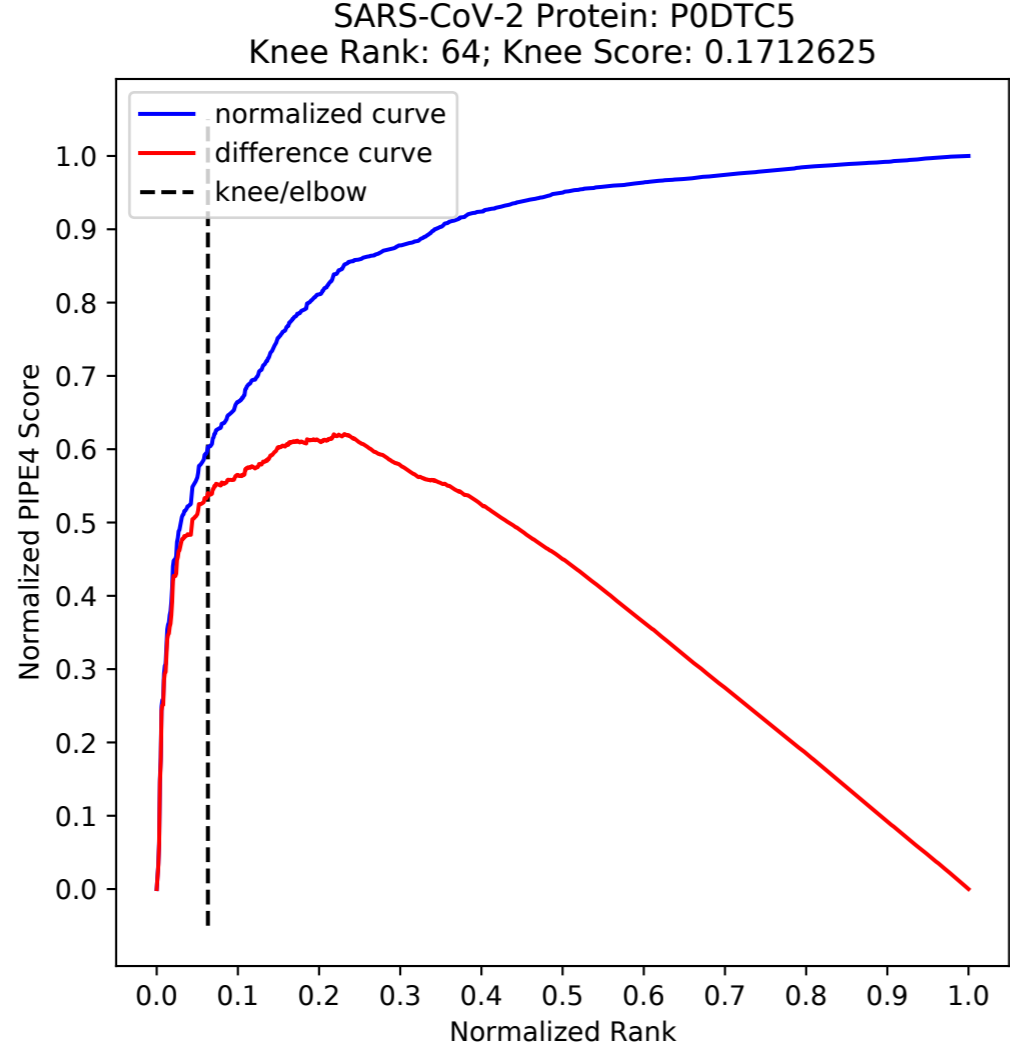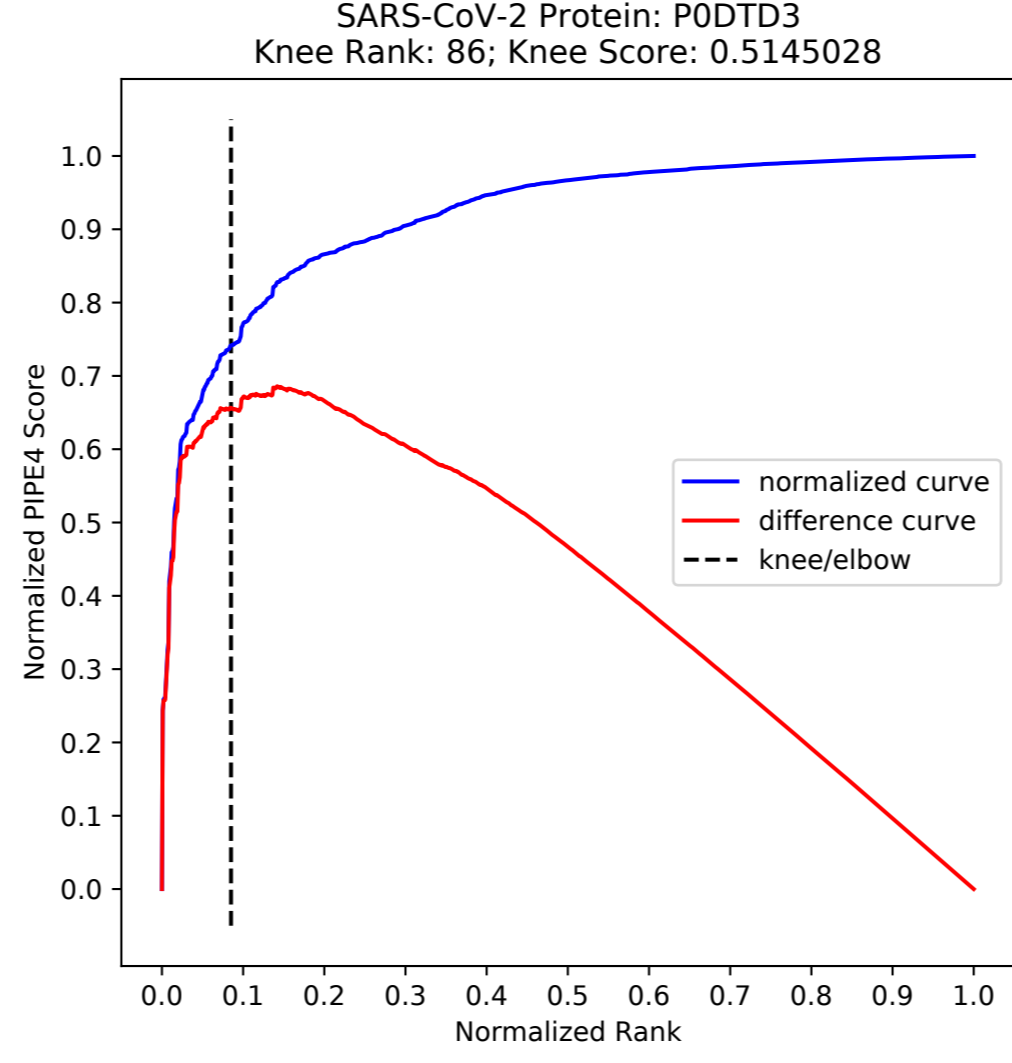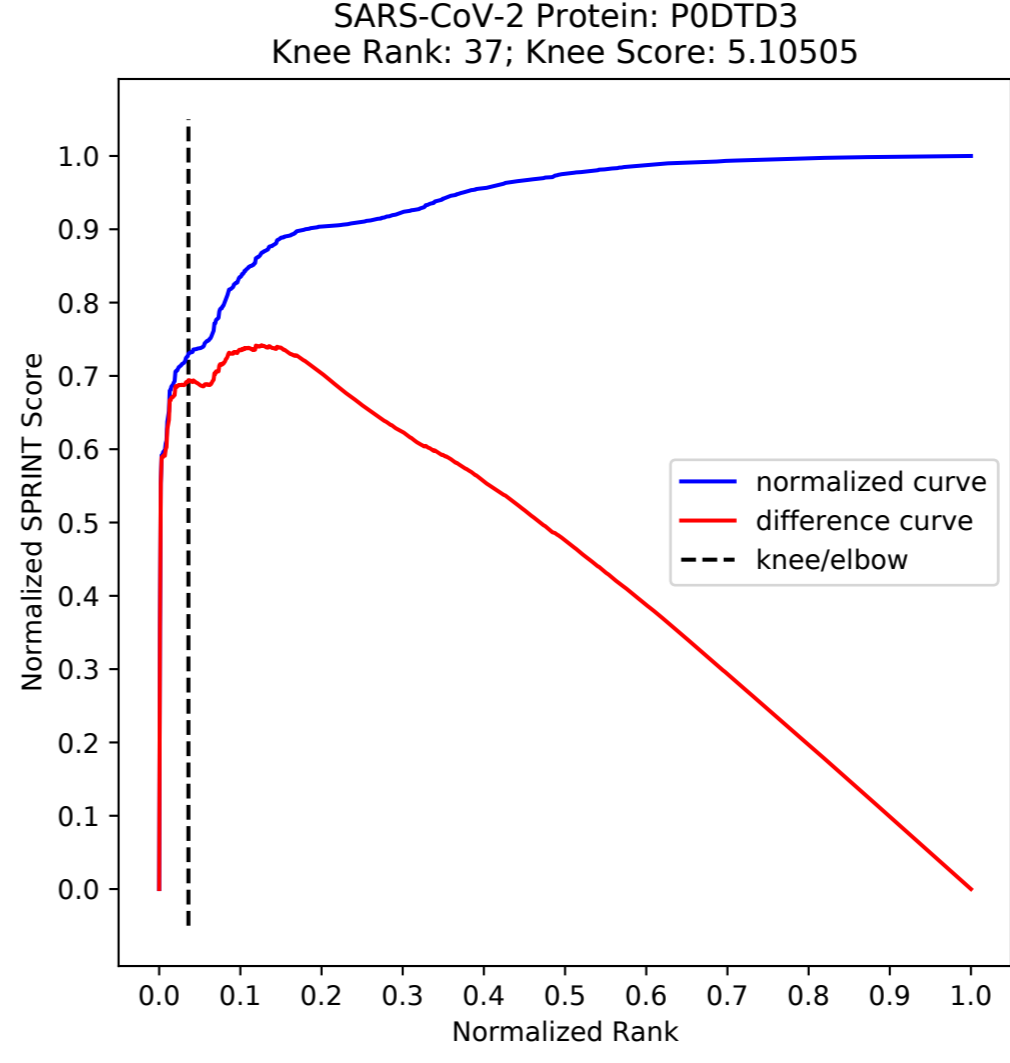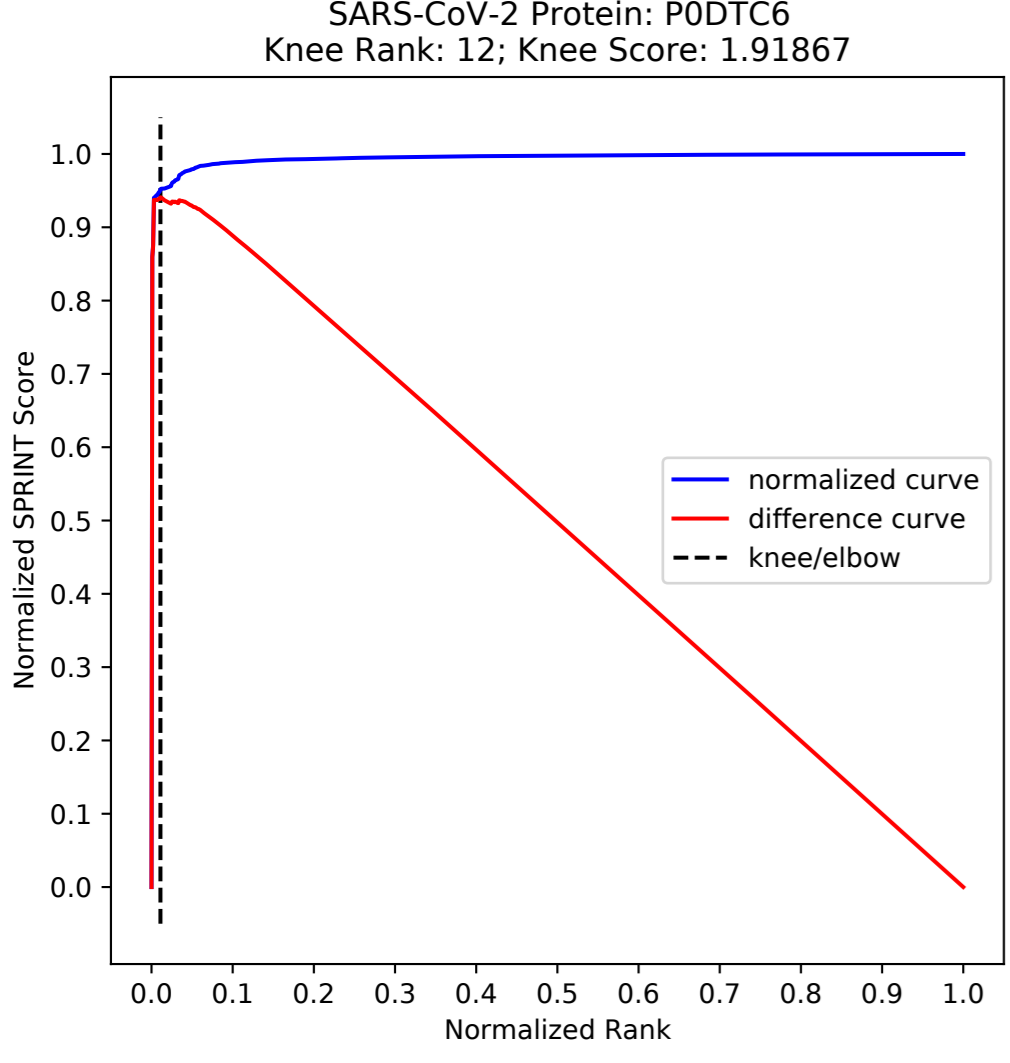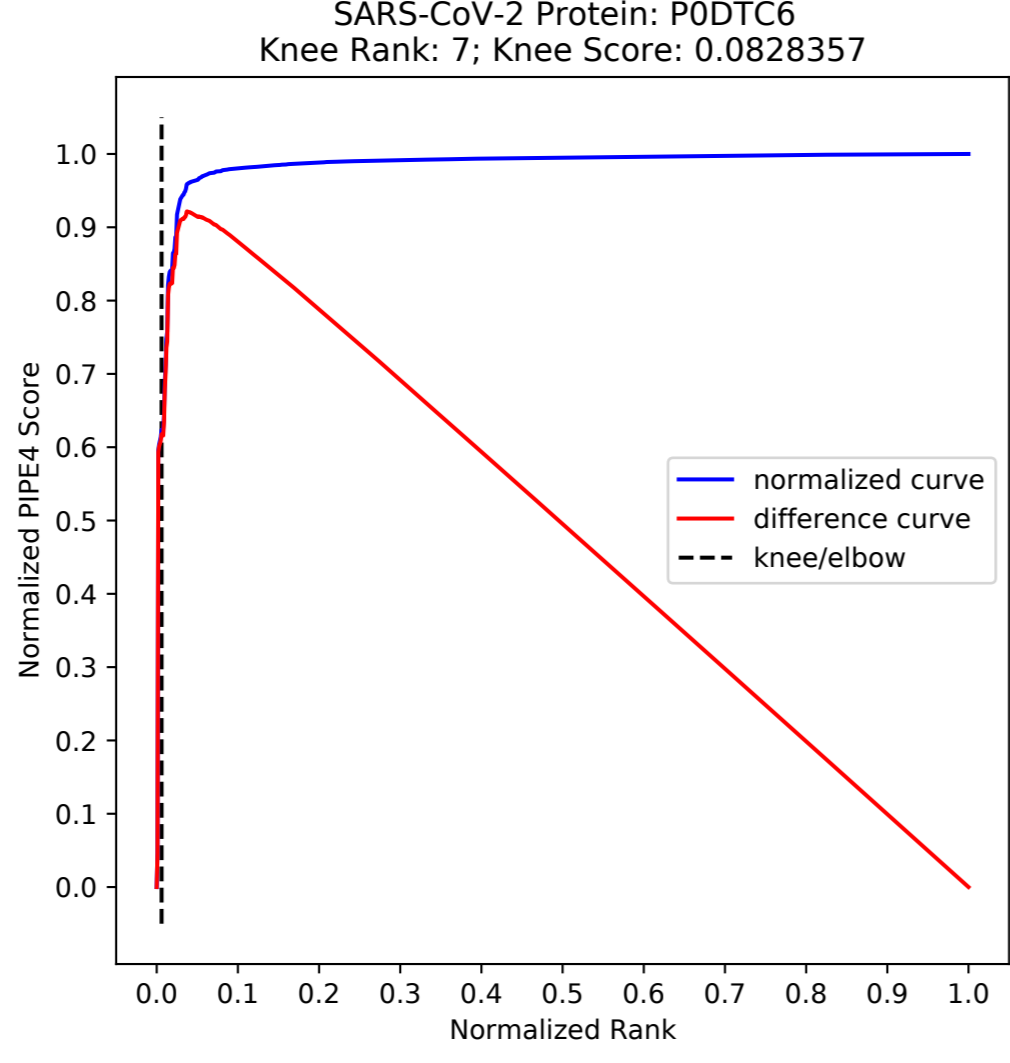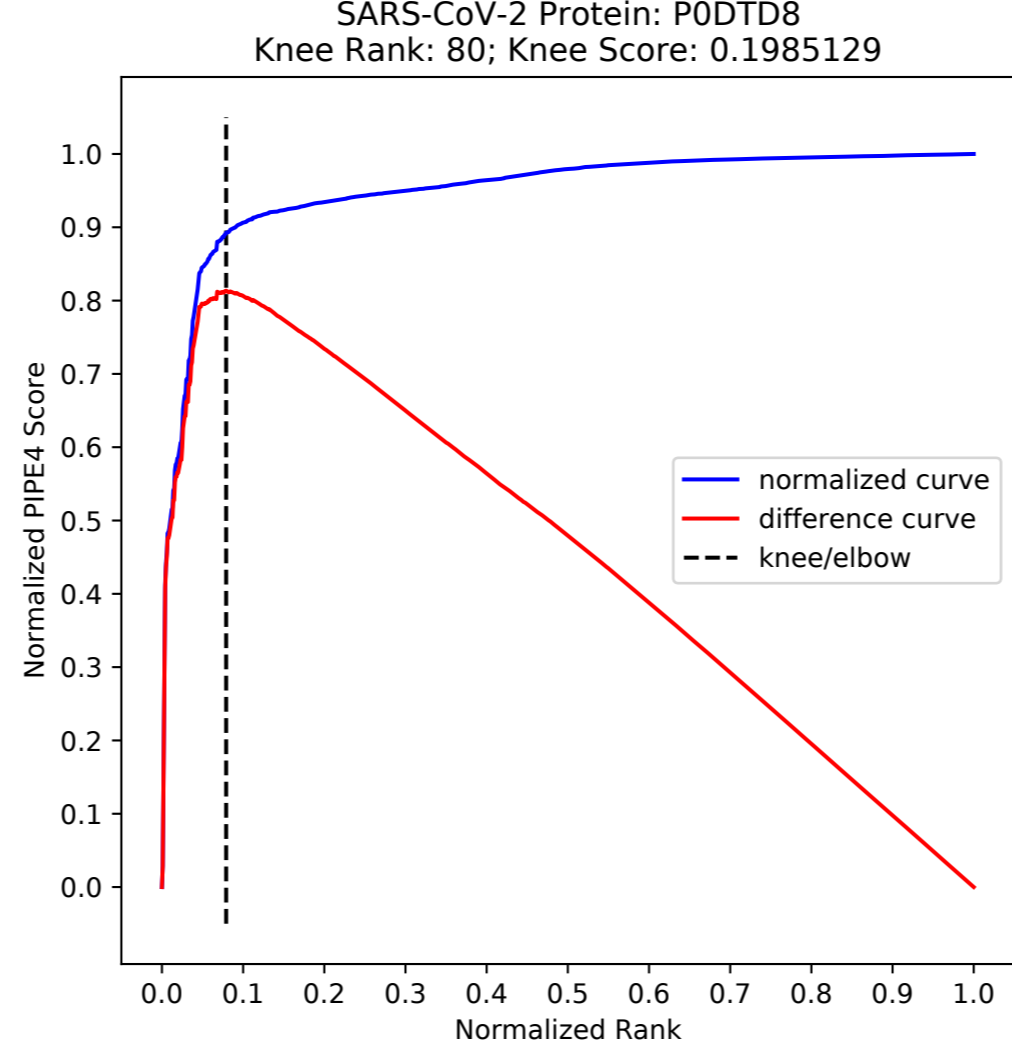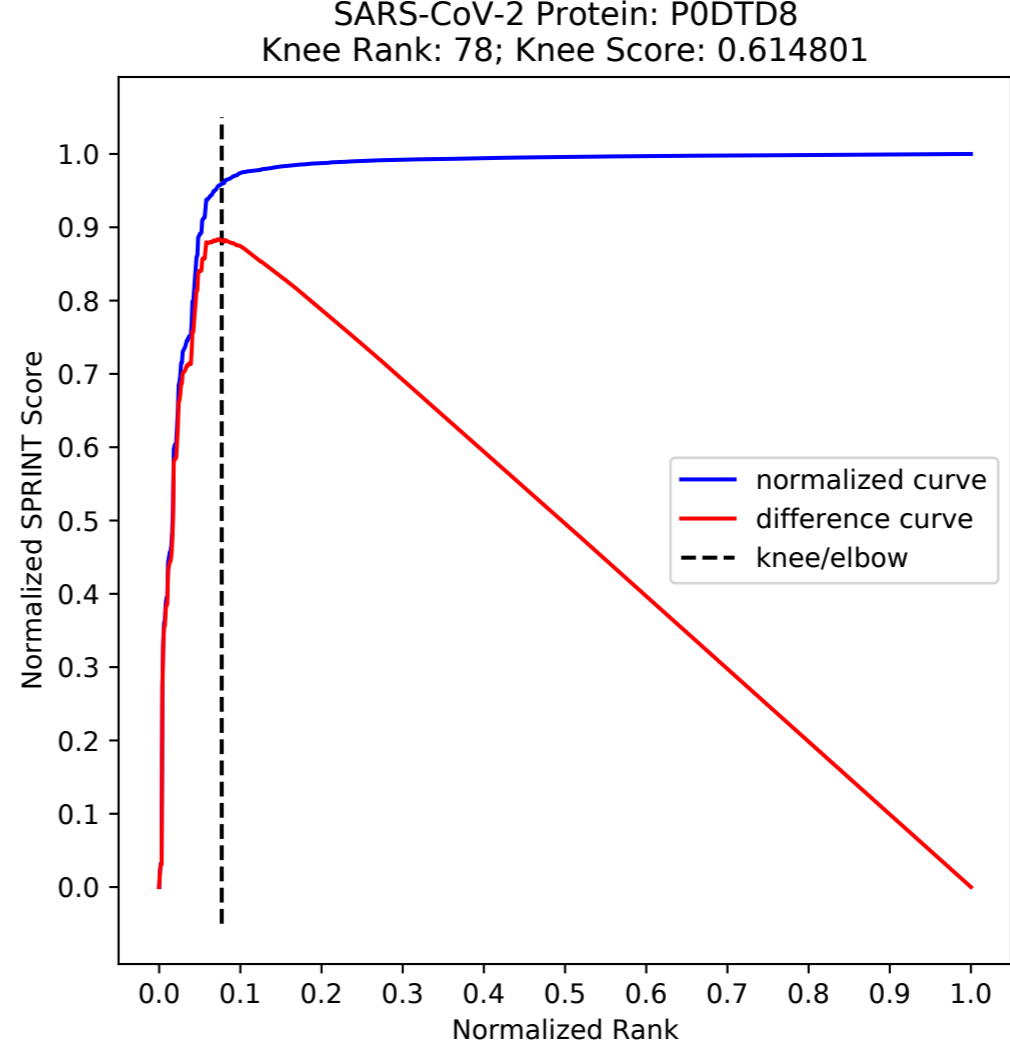

Supplement: Supplemental Information 9 — Each of the subplots highlights the detected knee of the normalized top-1000 predictions obtained using the Kneedle algorithm. The differences curve plots the value obtained from subtracting the perpendicular distance of each point to y=x from the distance of each point vertically to y=x of the normalized plot. The peak of this curve, parameterized by S, estimates the location of the knee. [file peerj-09-11117-s009.pdf]

# SPRINT

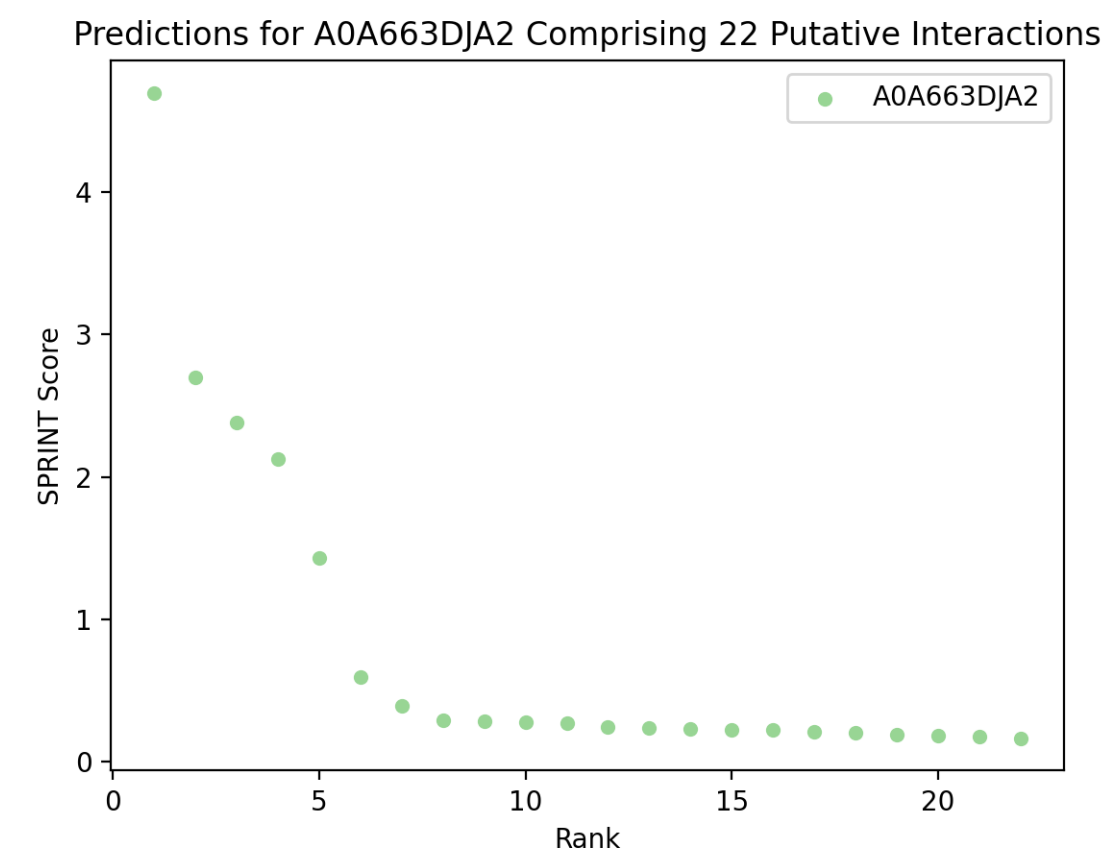

## PIPE4

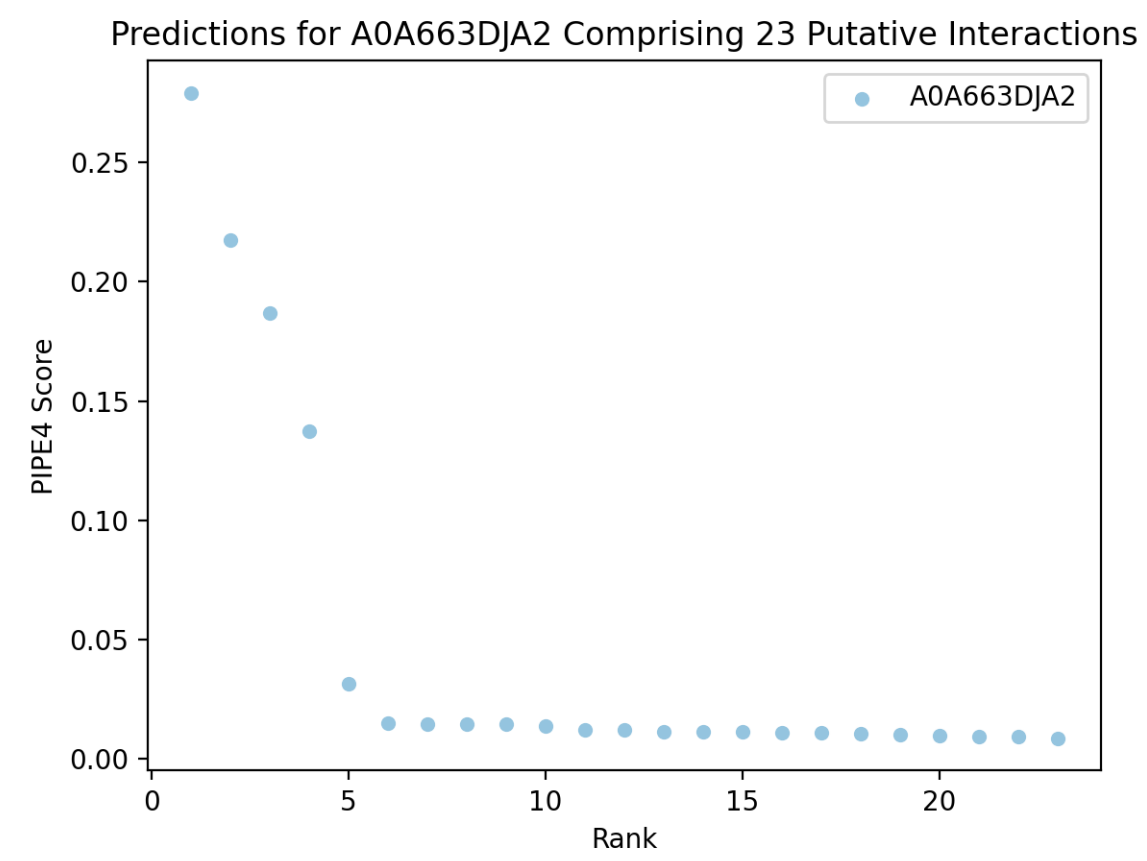

## PIPE4

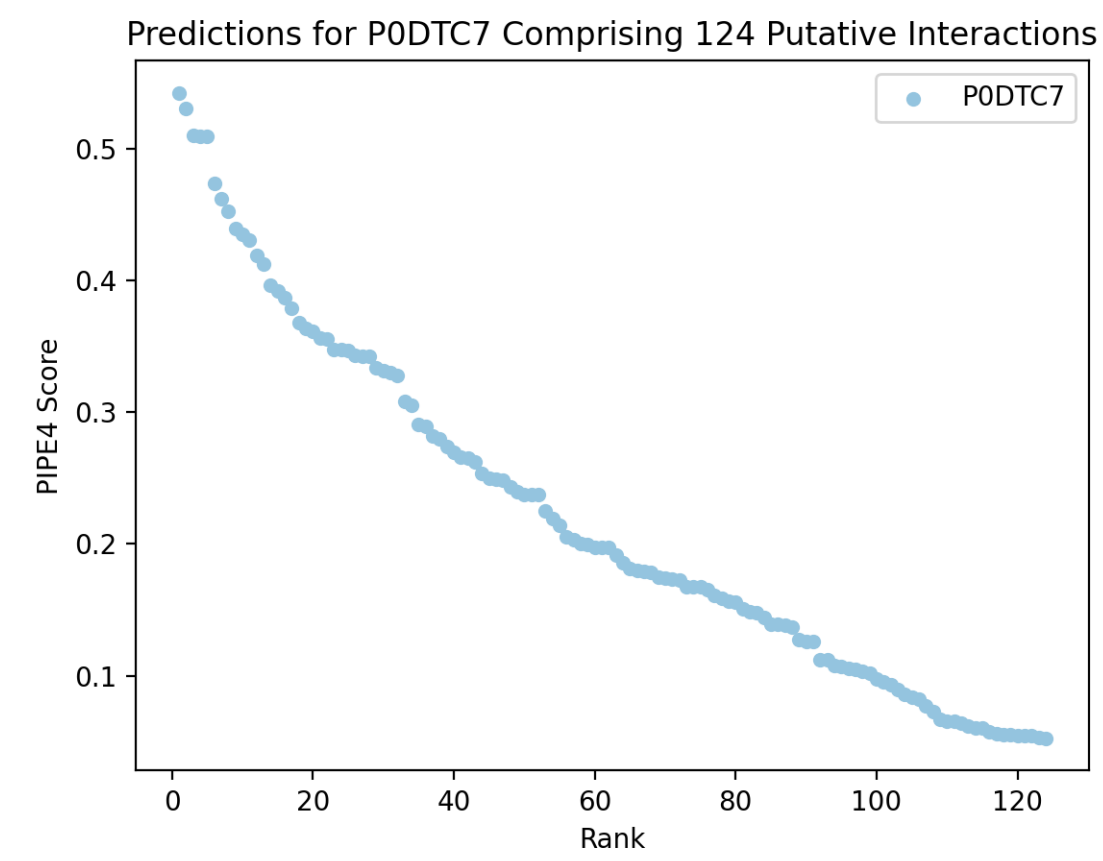

# SPRINT

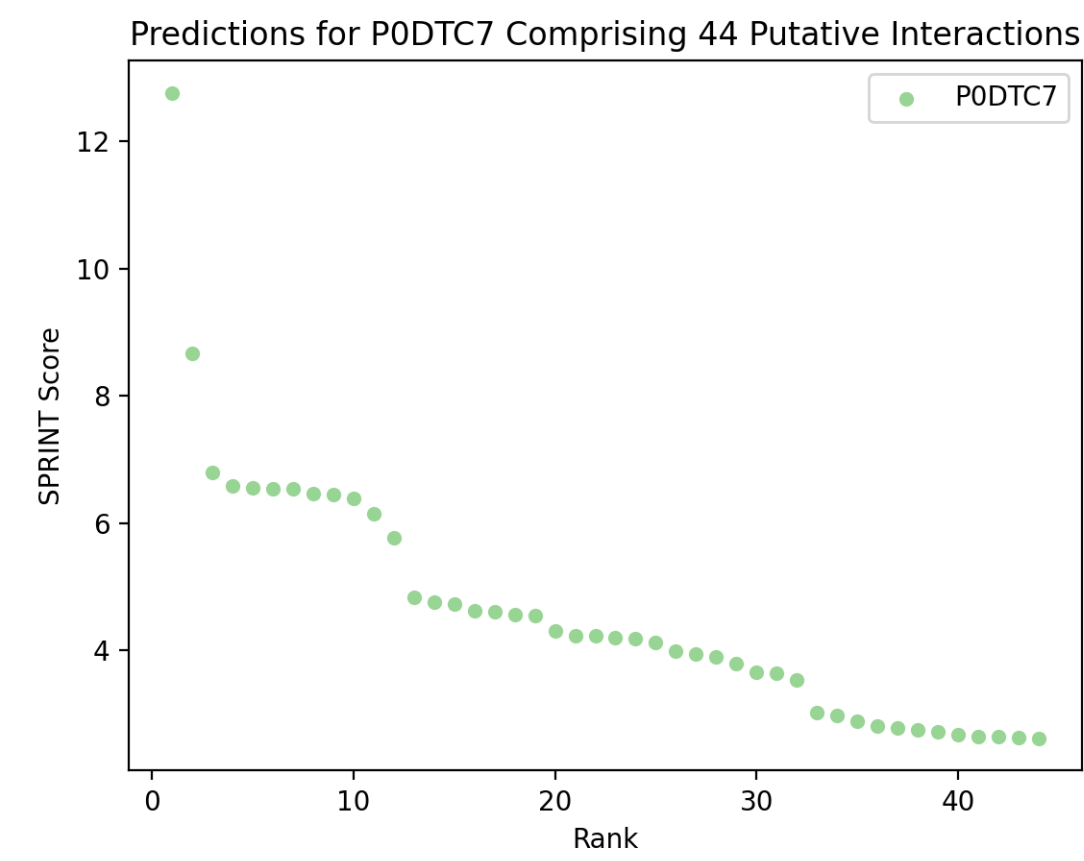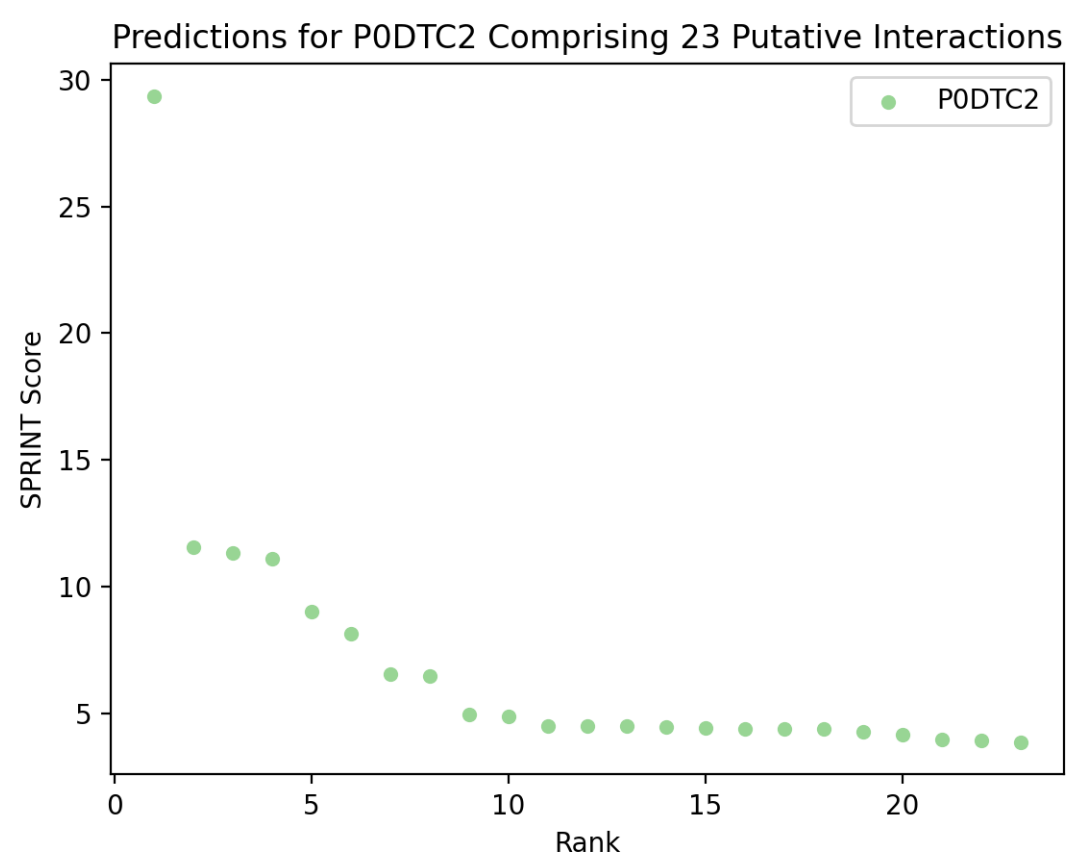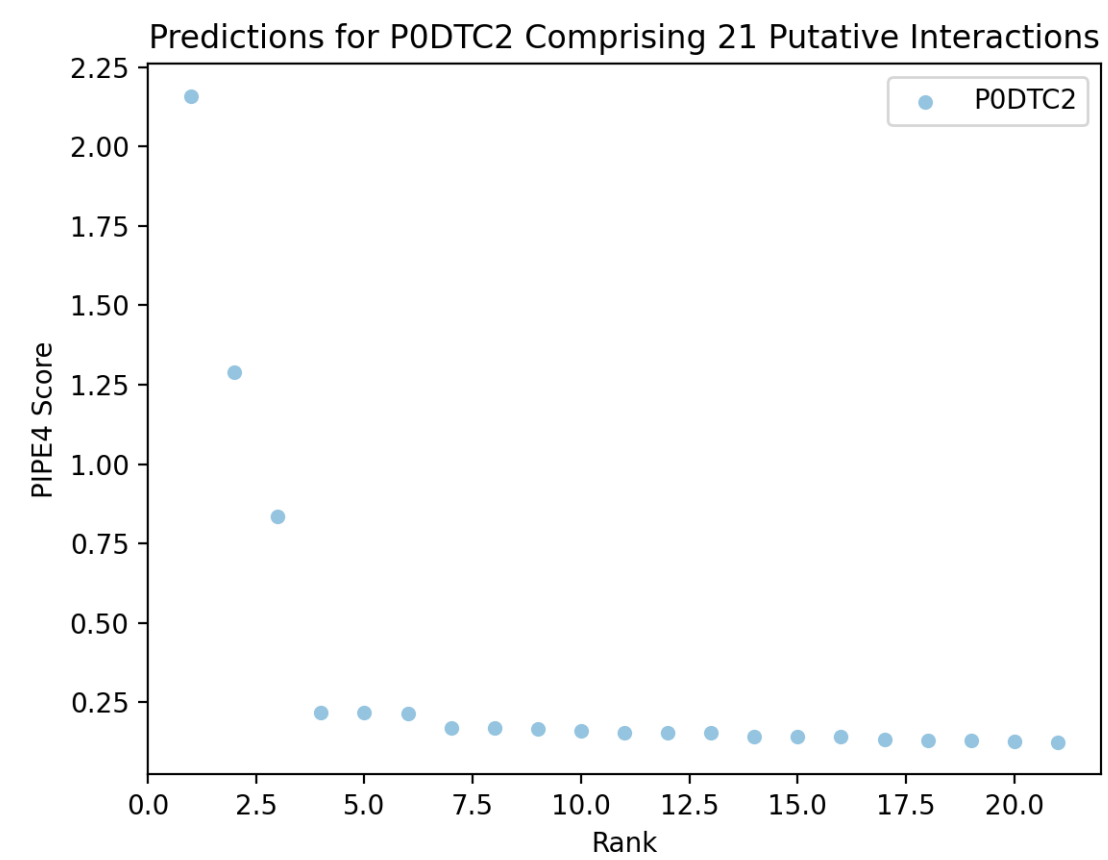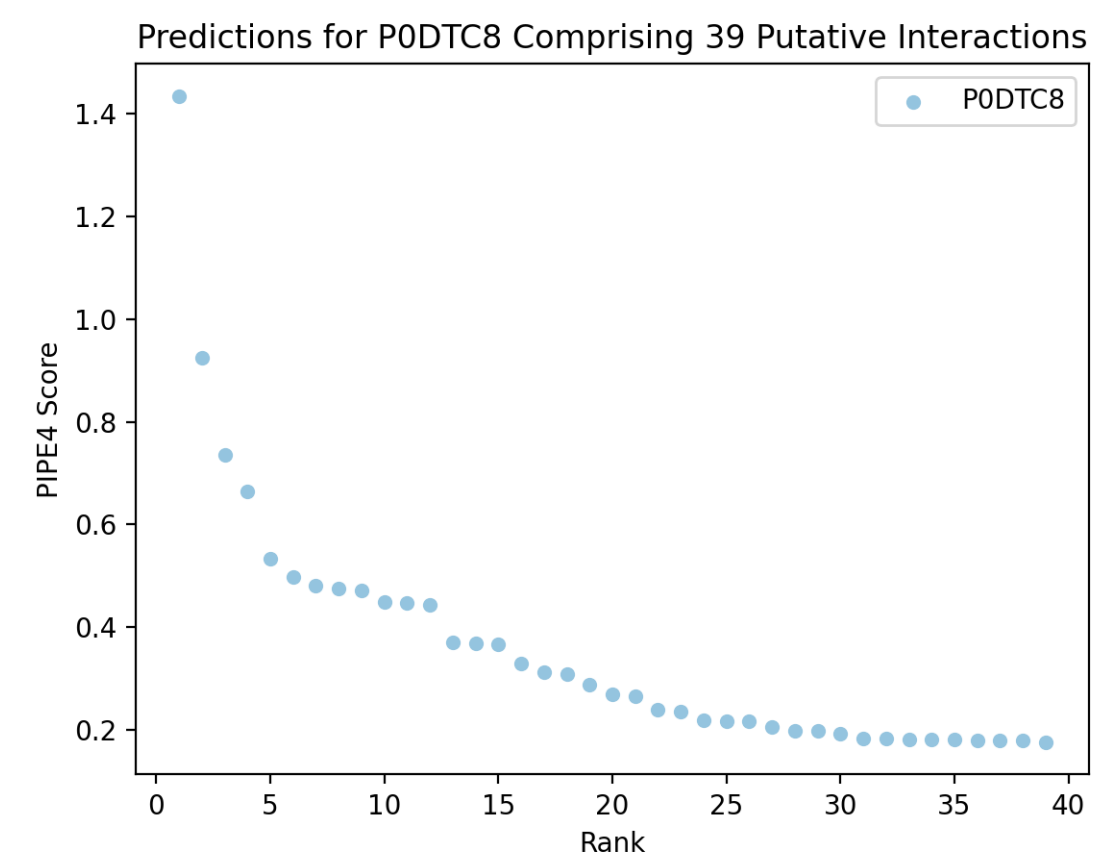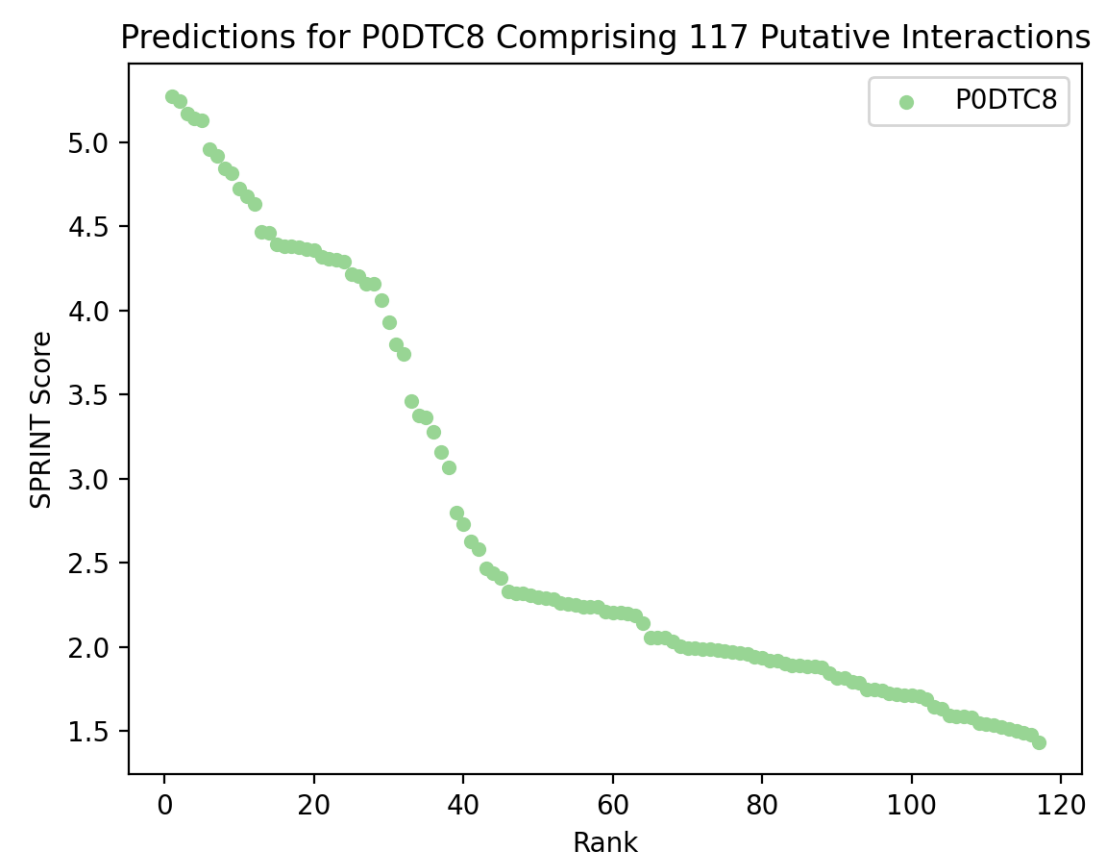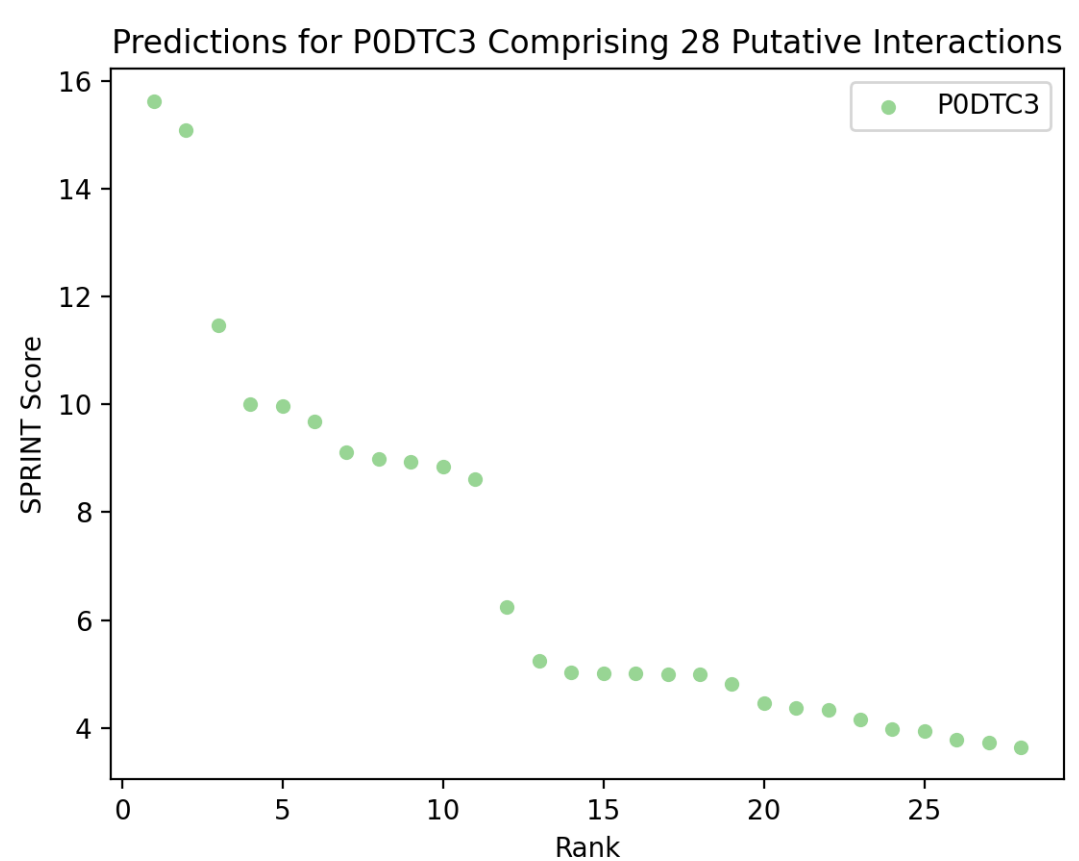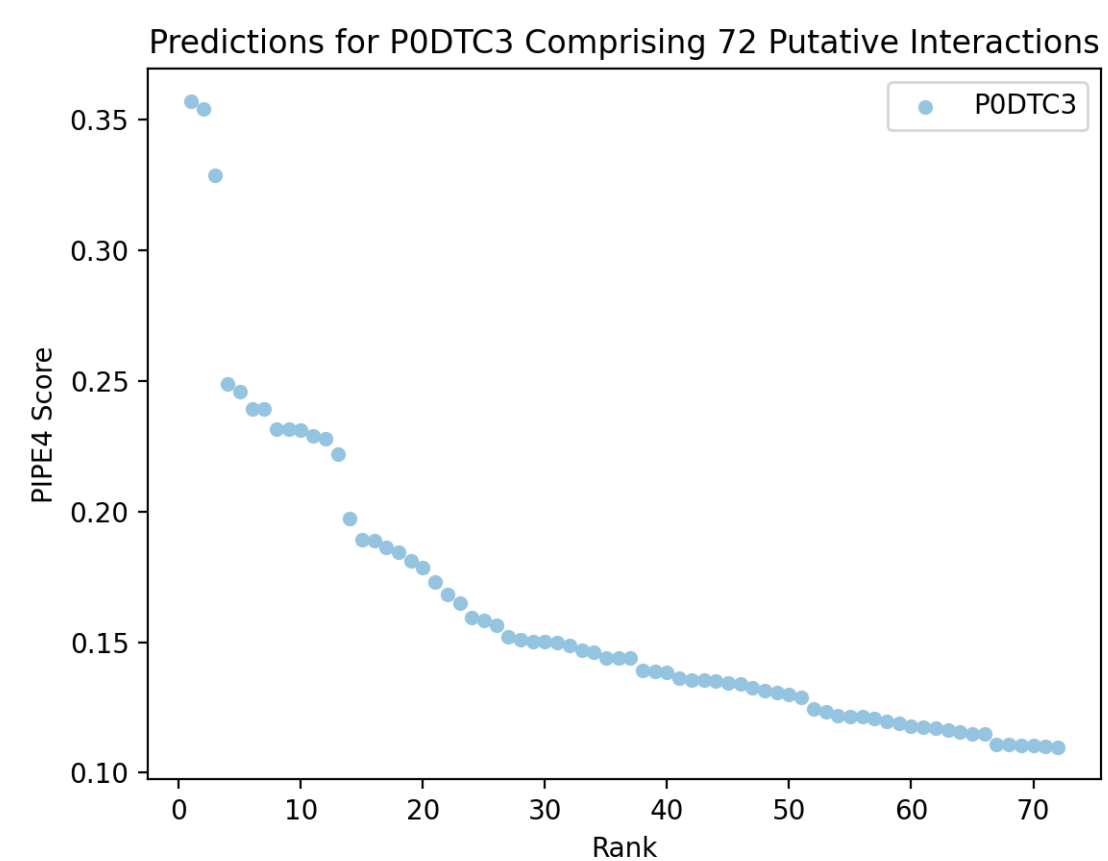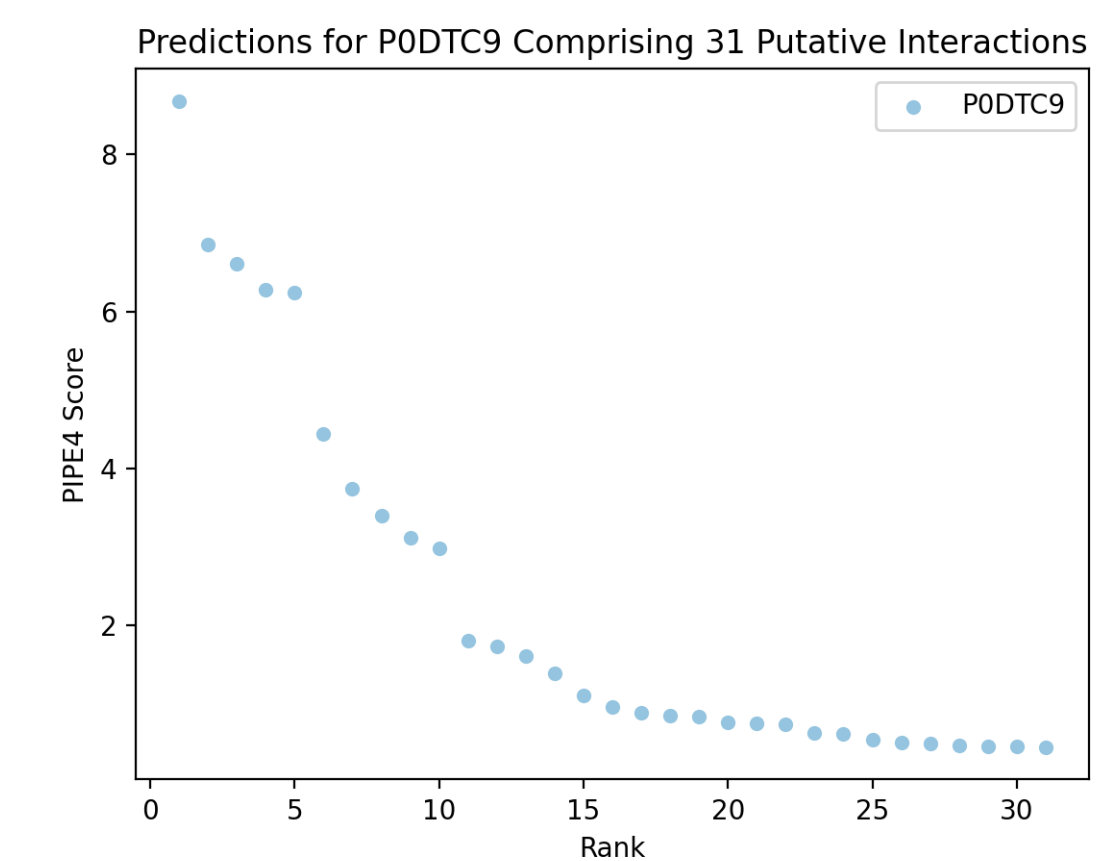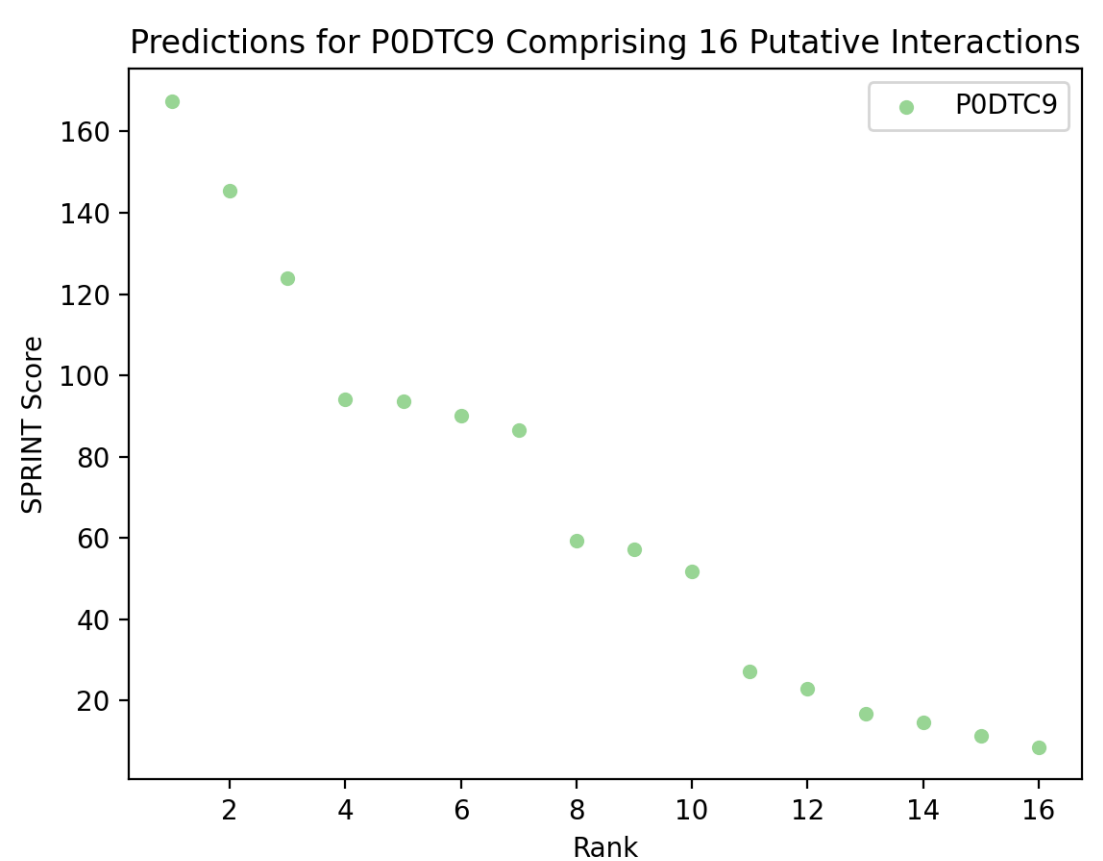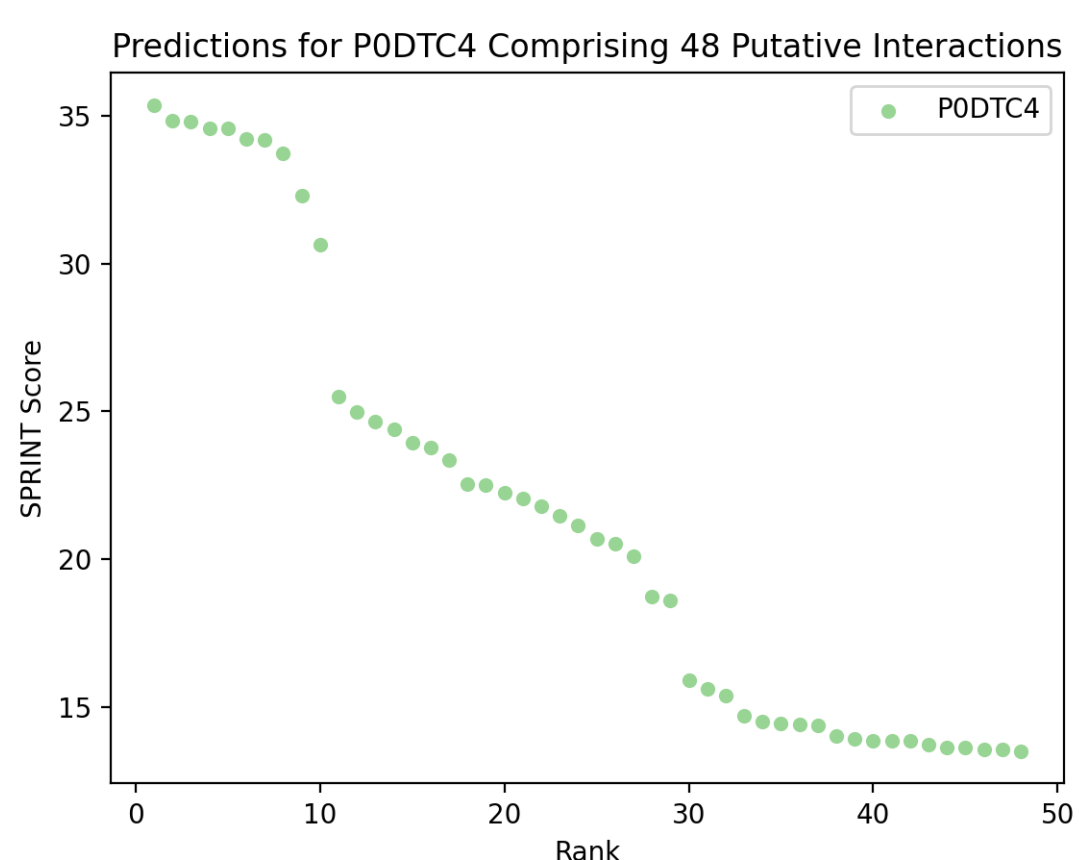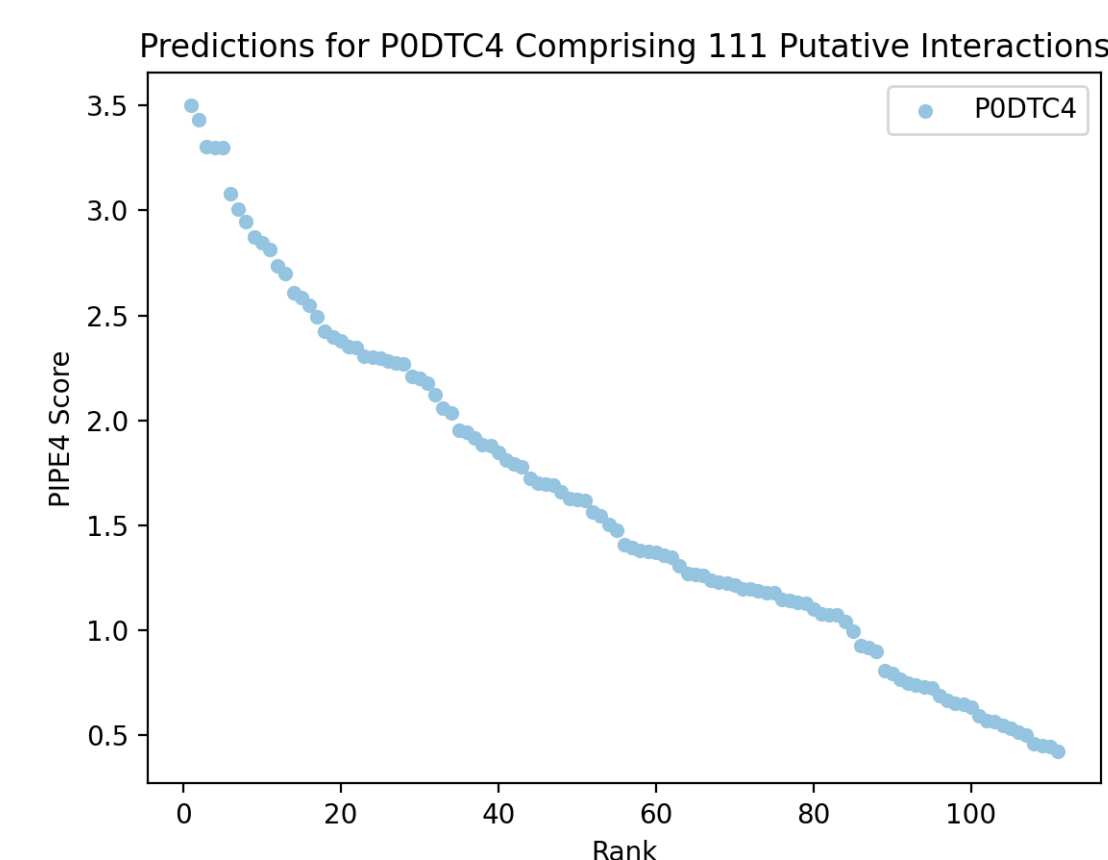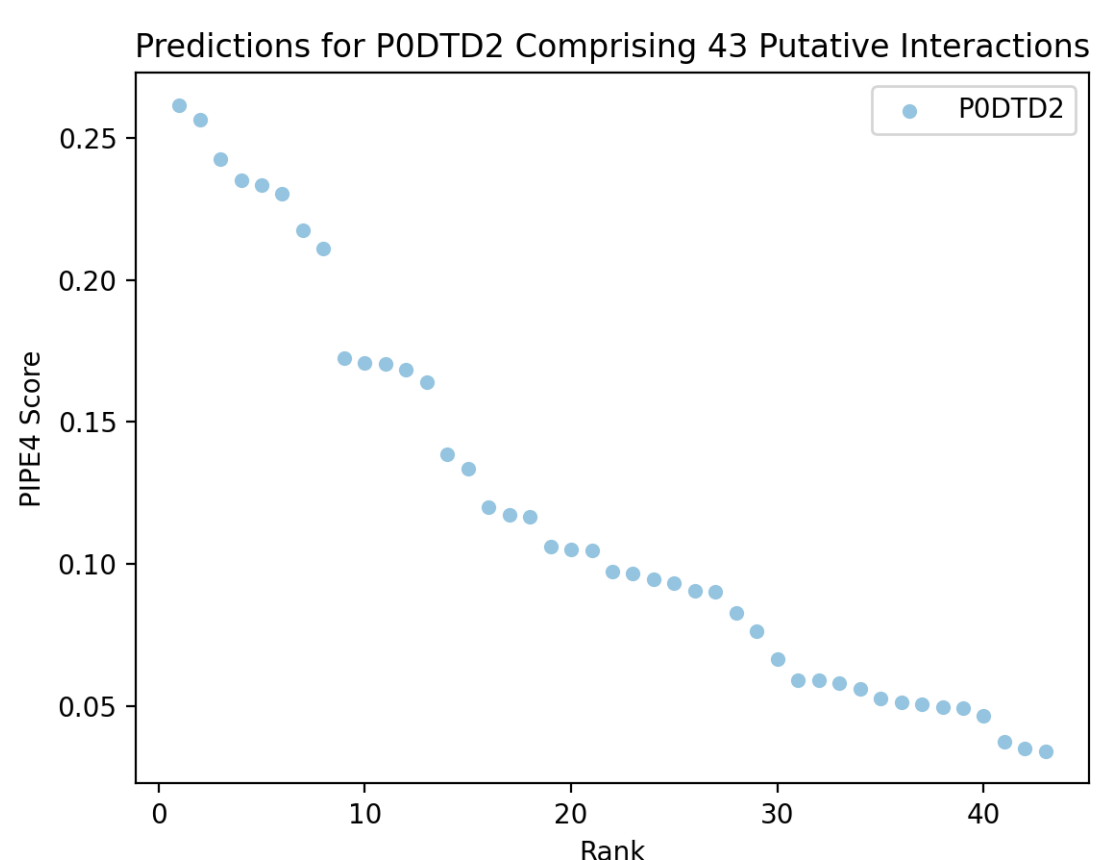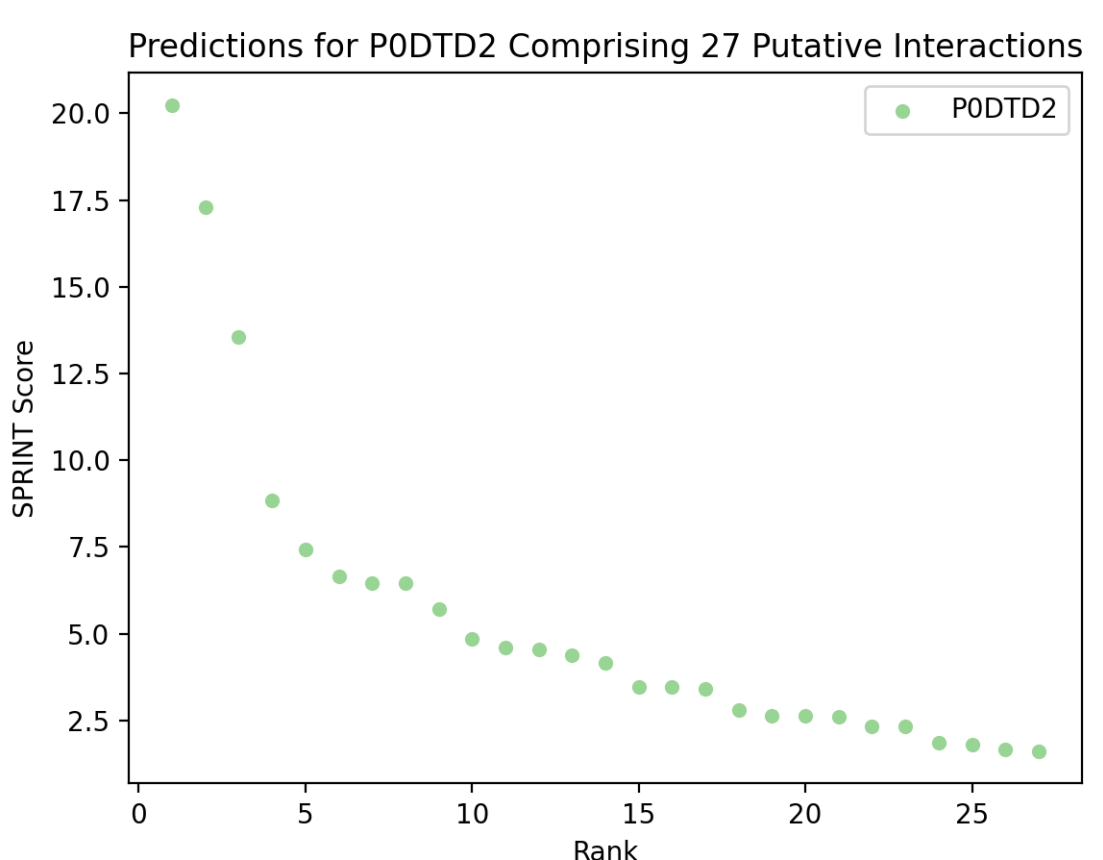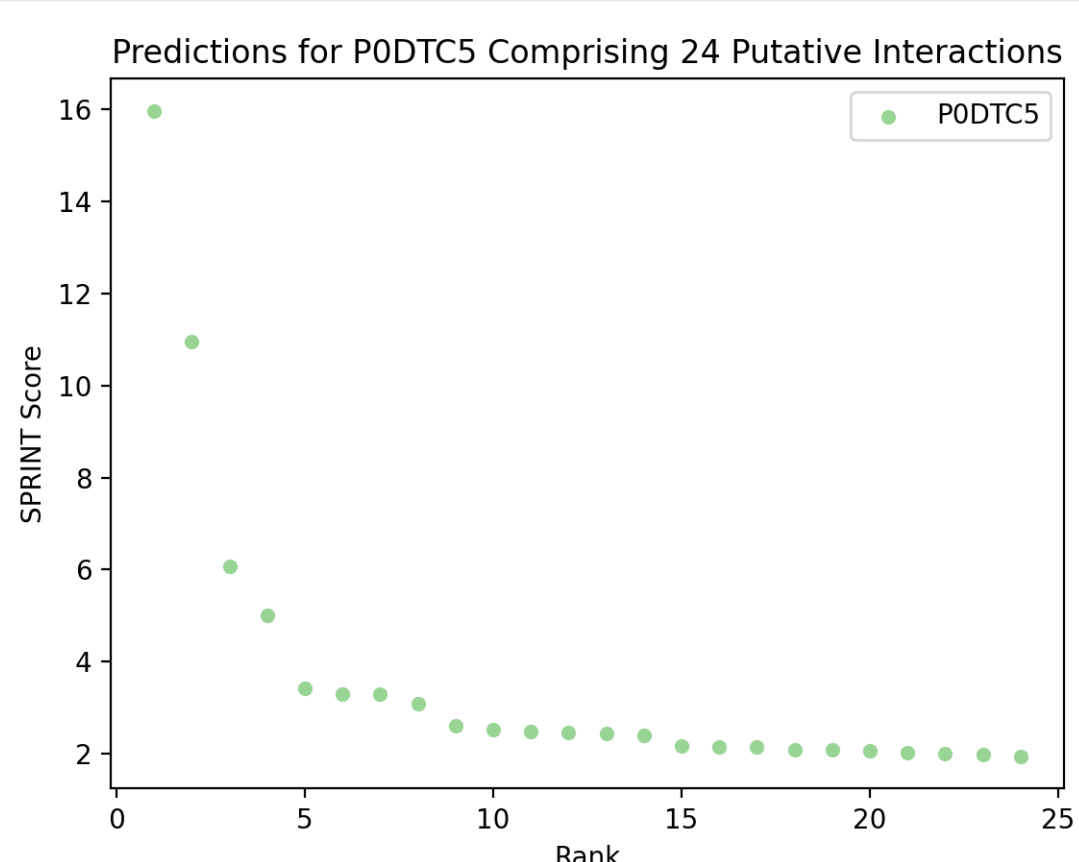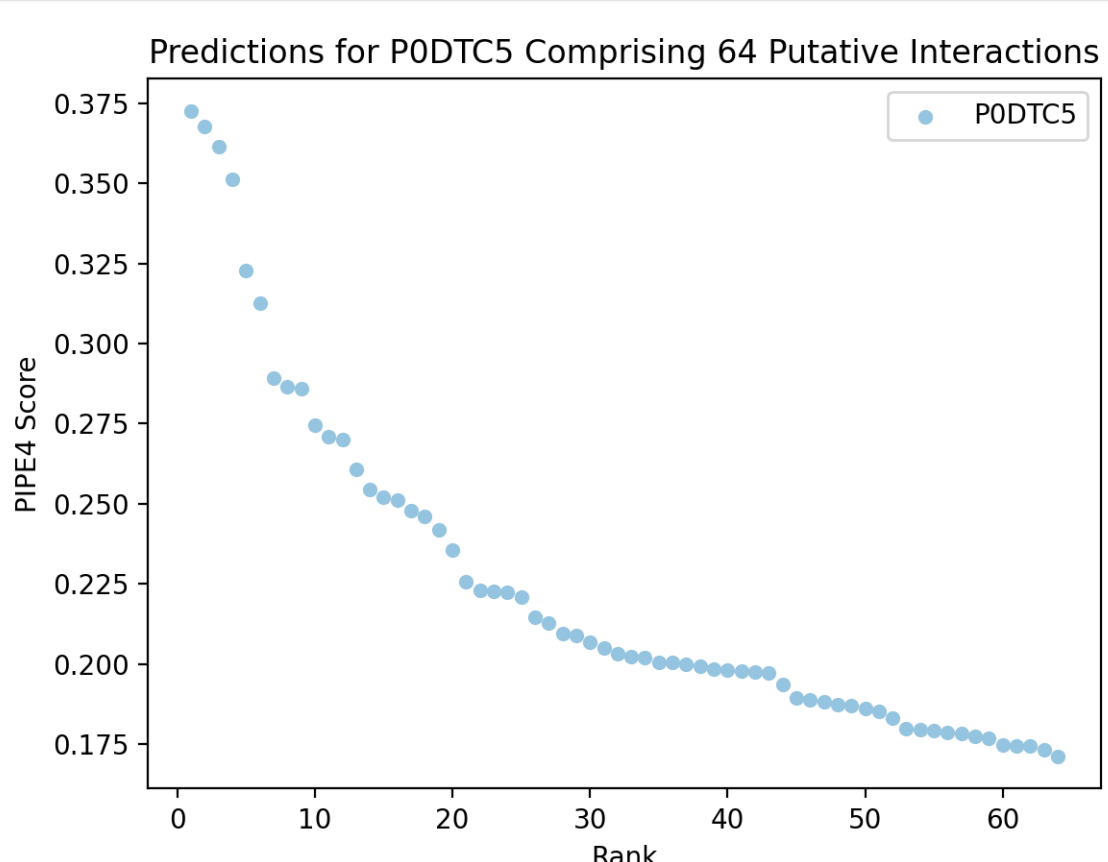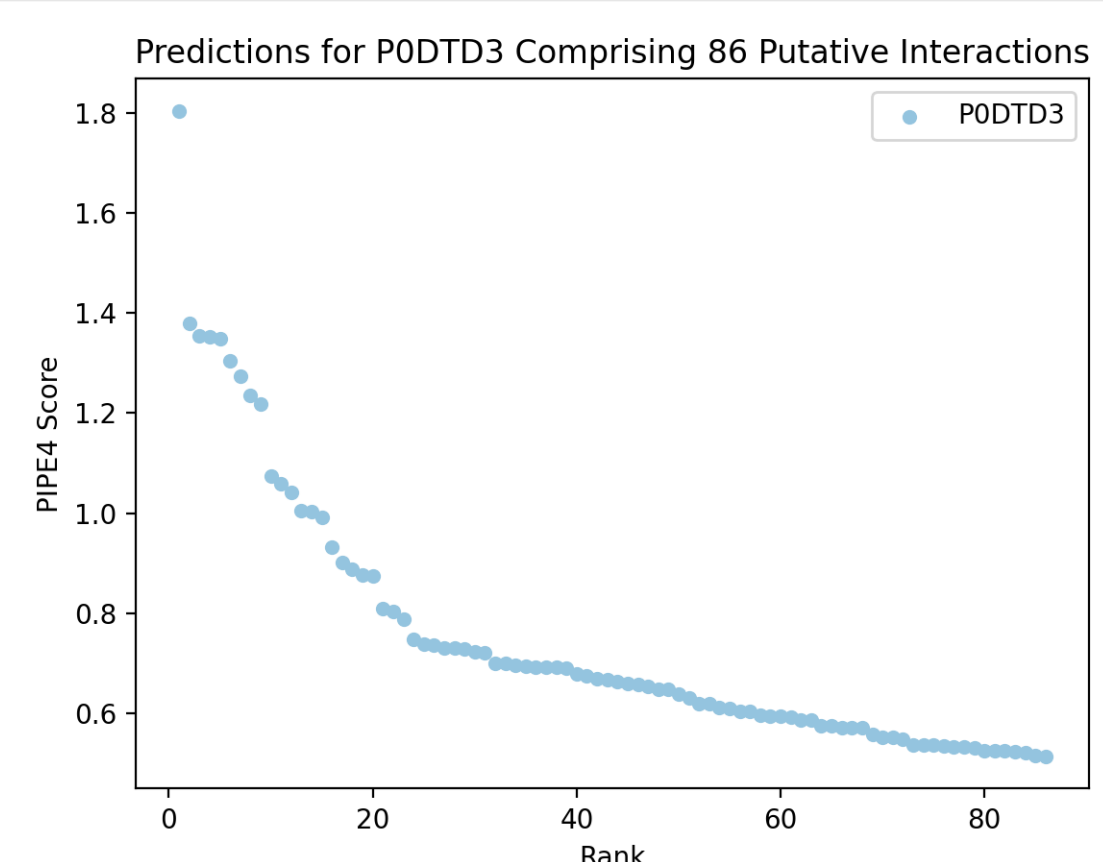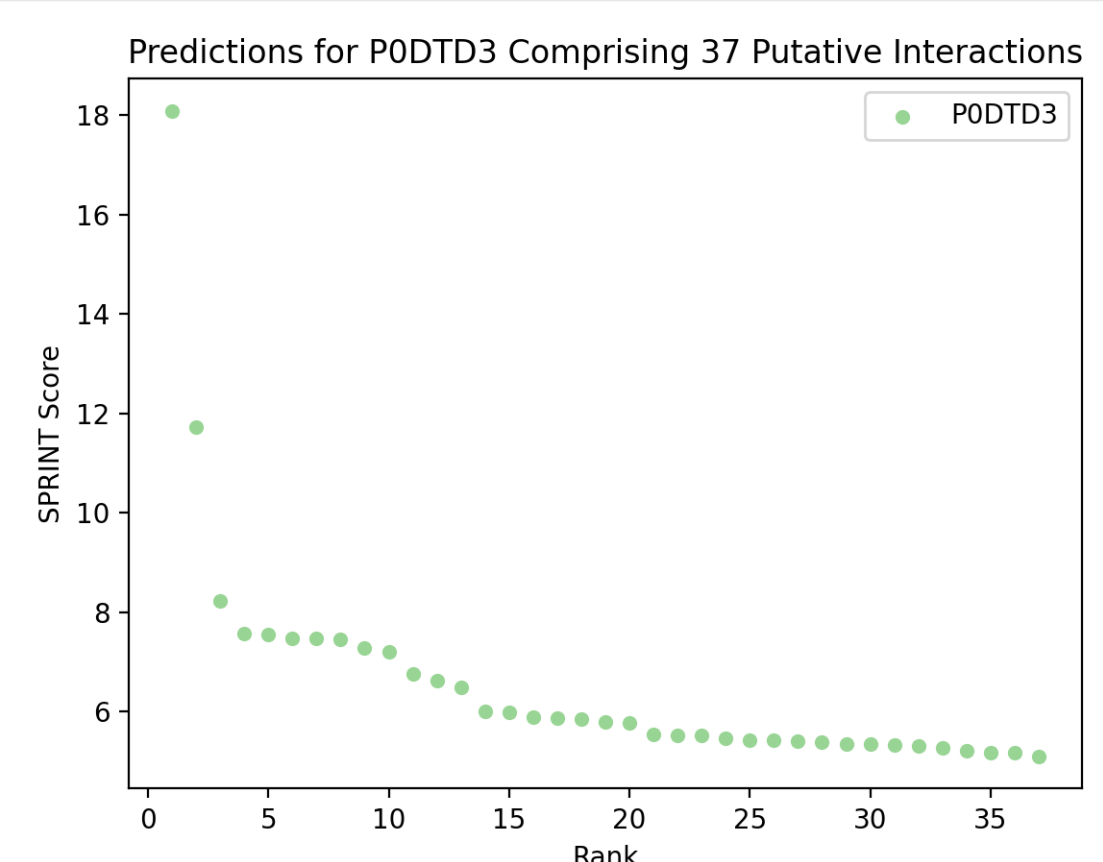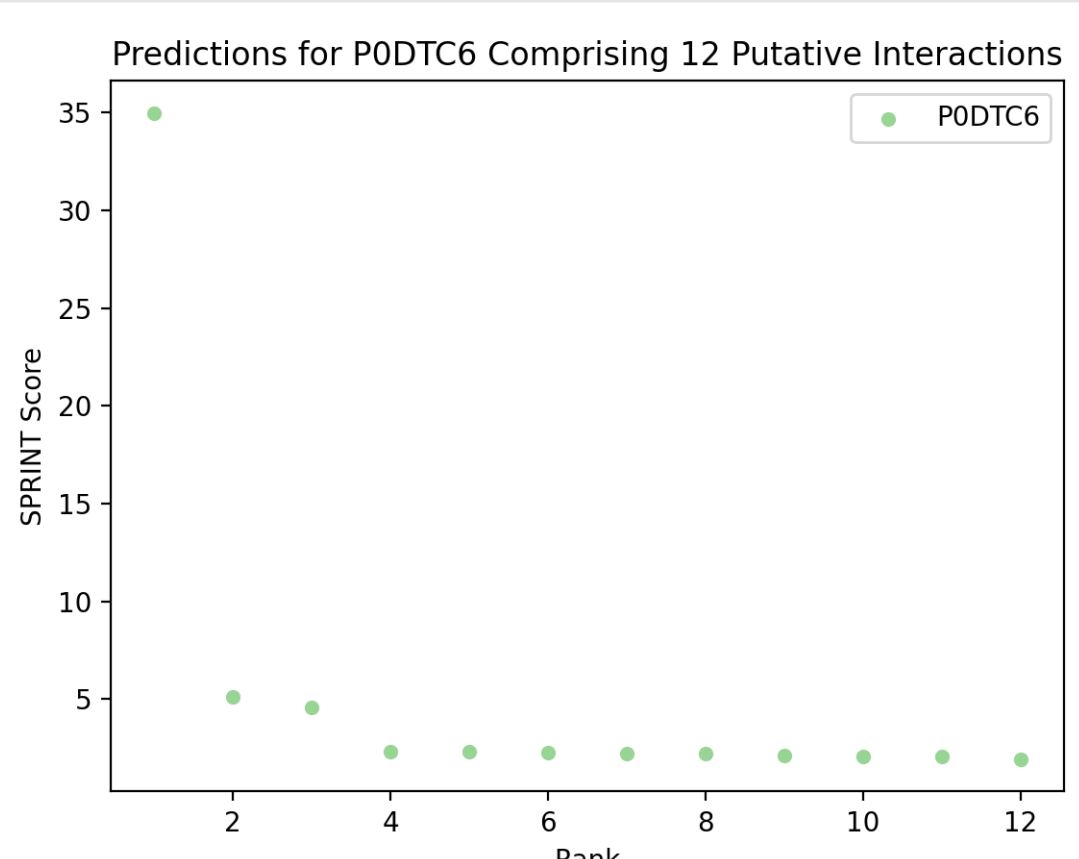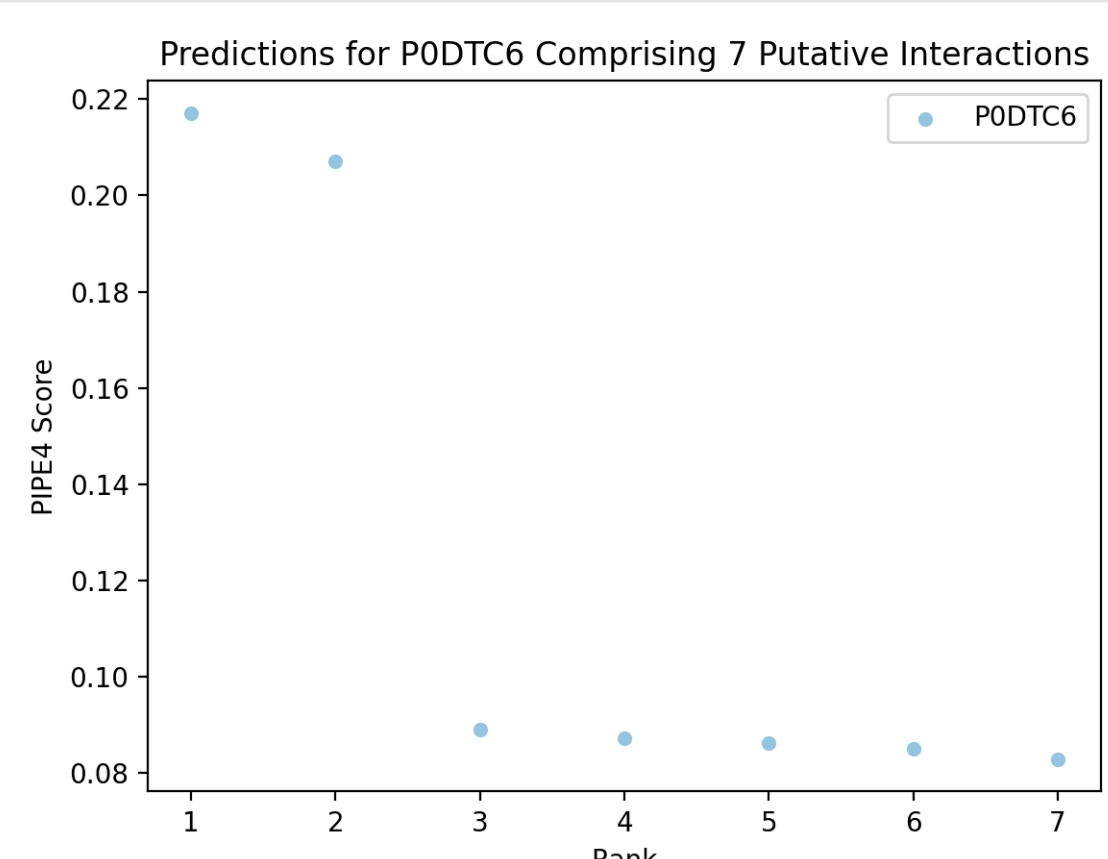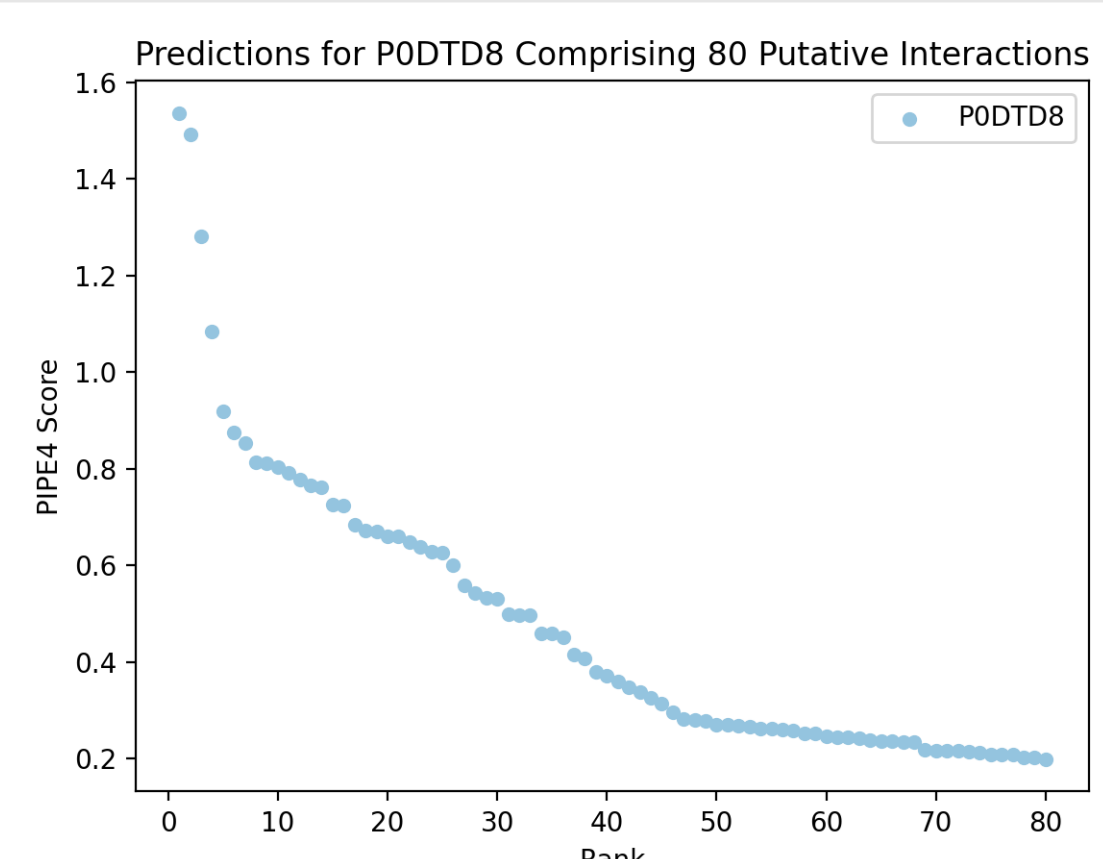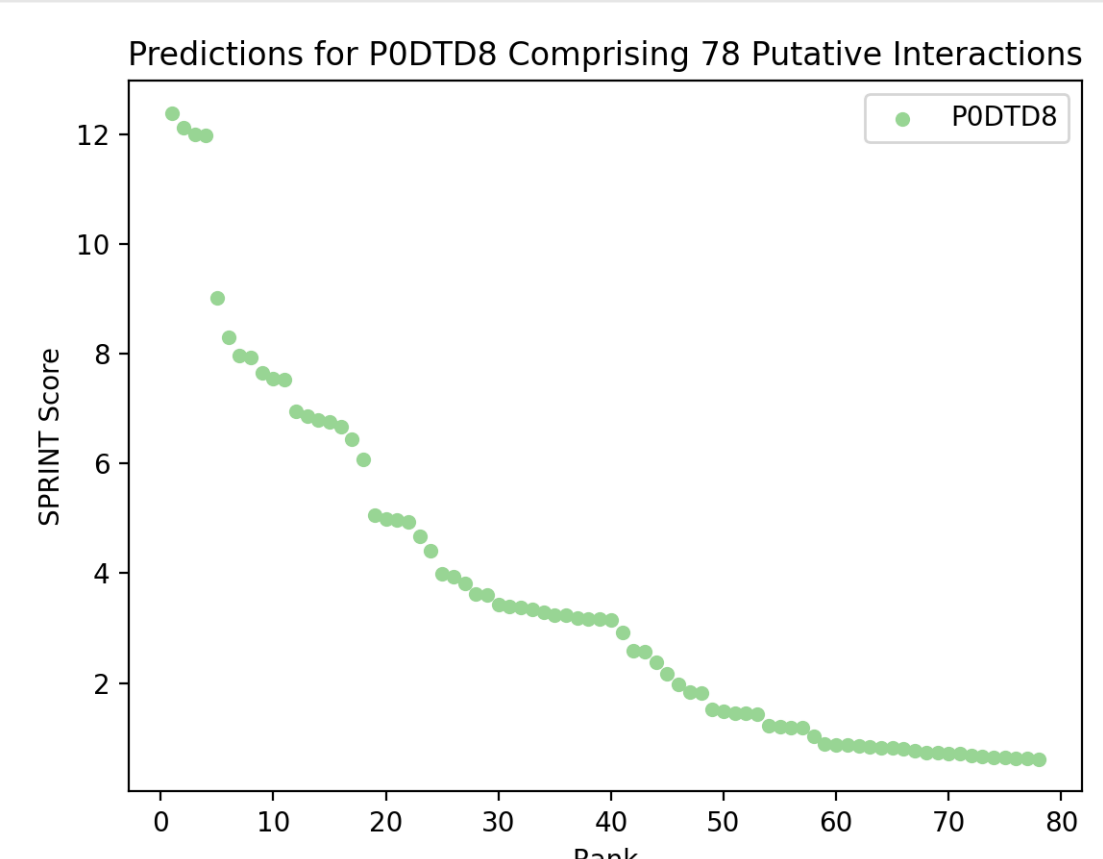

# SPRINT

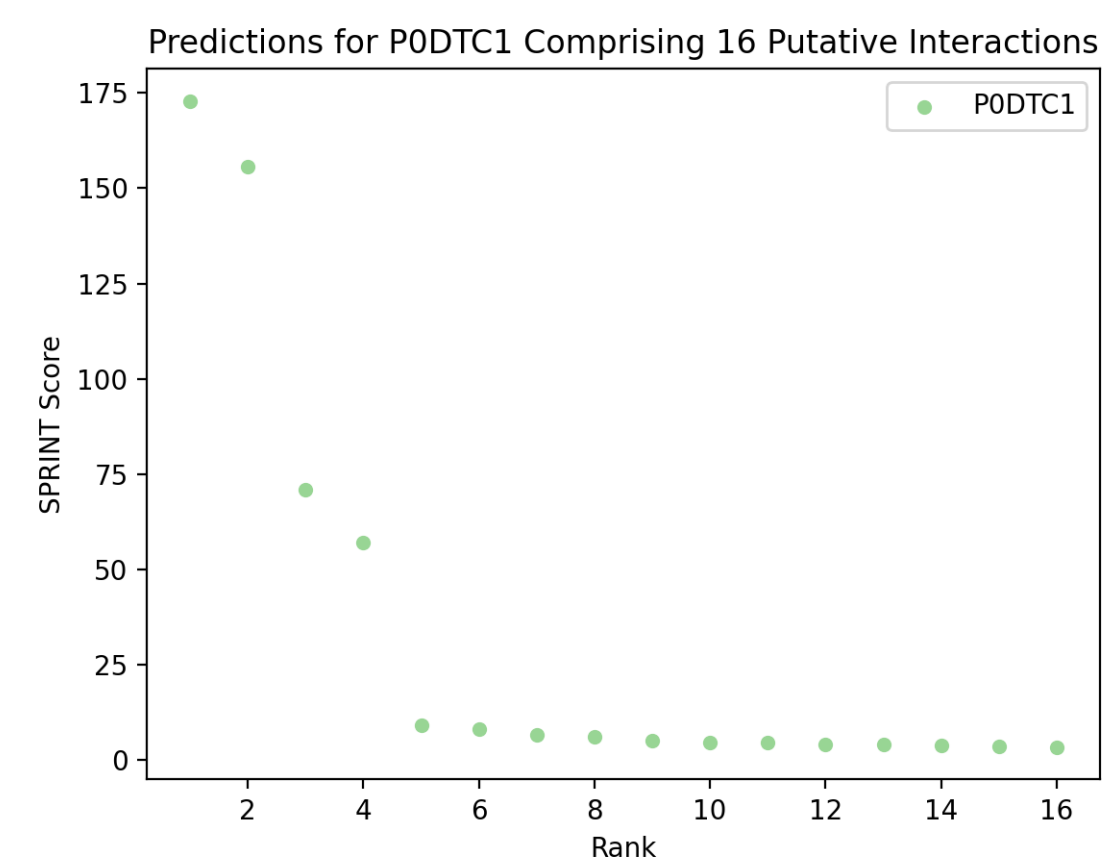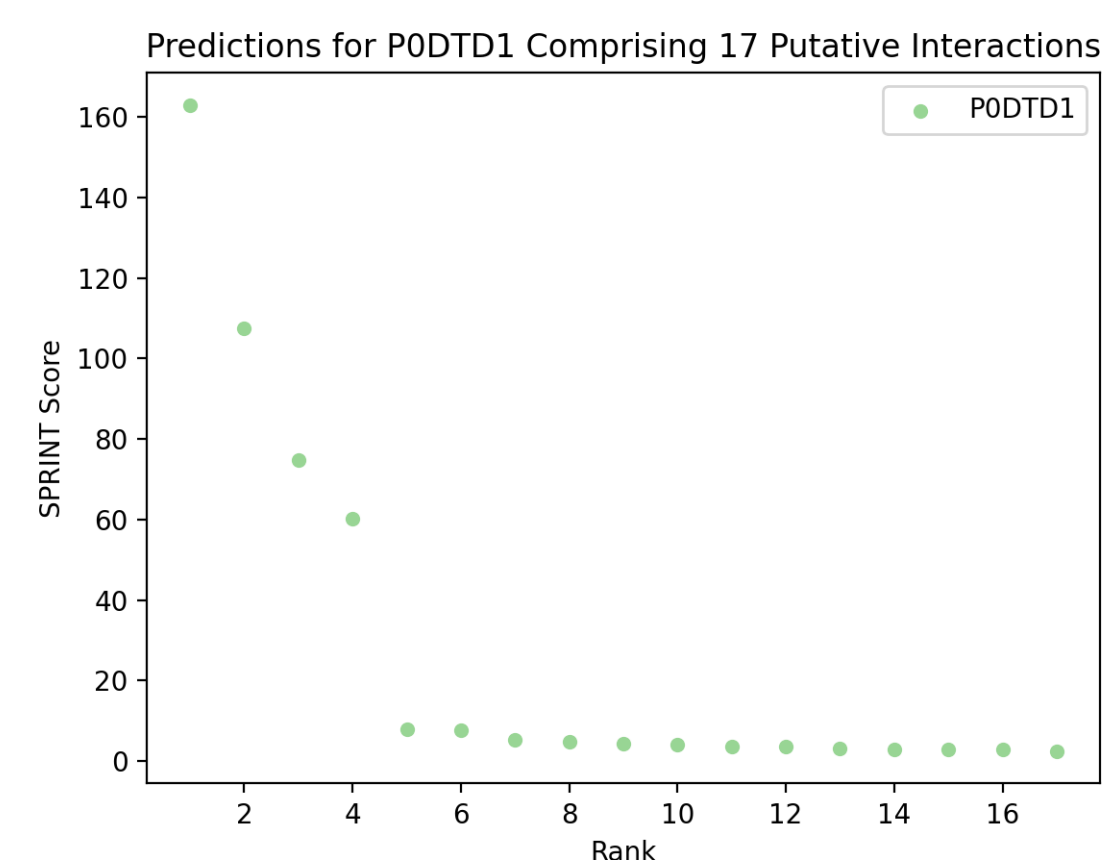

Supplement: Supplemental Information 10 [file peerj-09-11117-s010.pdf]

# PIPE4

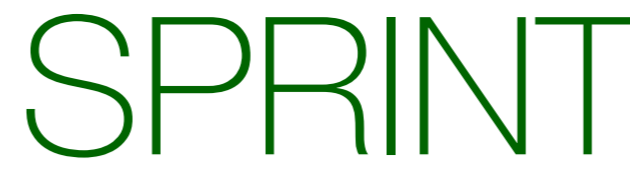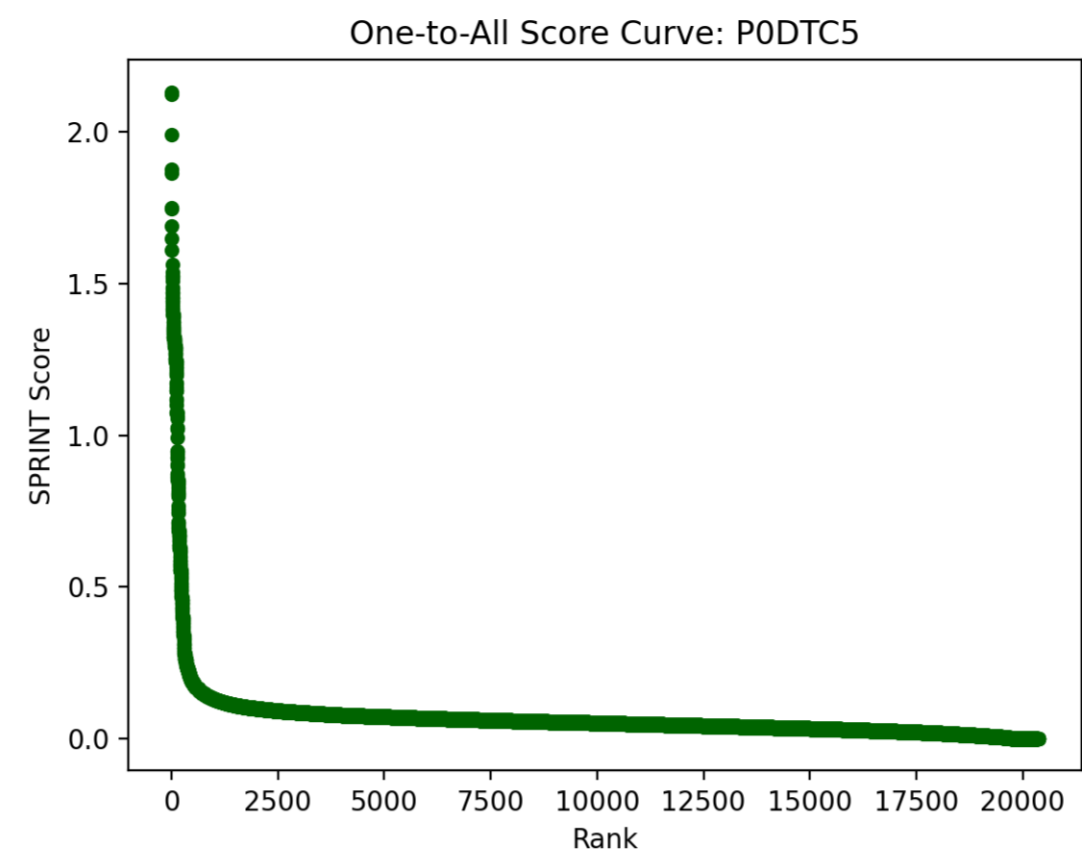

# SPRINT

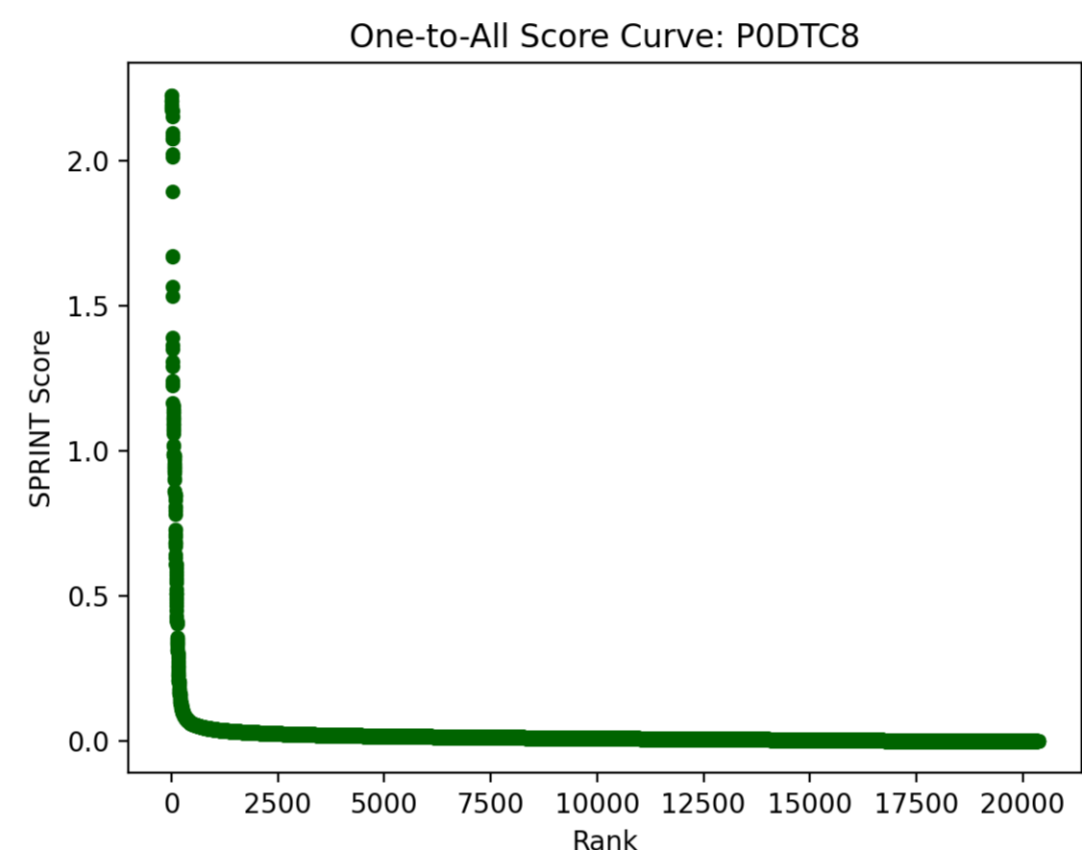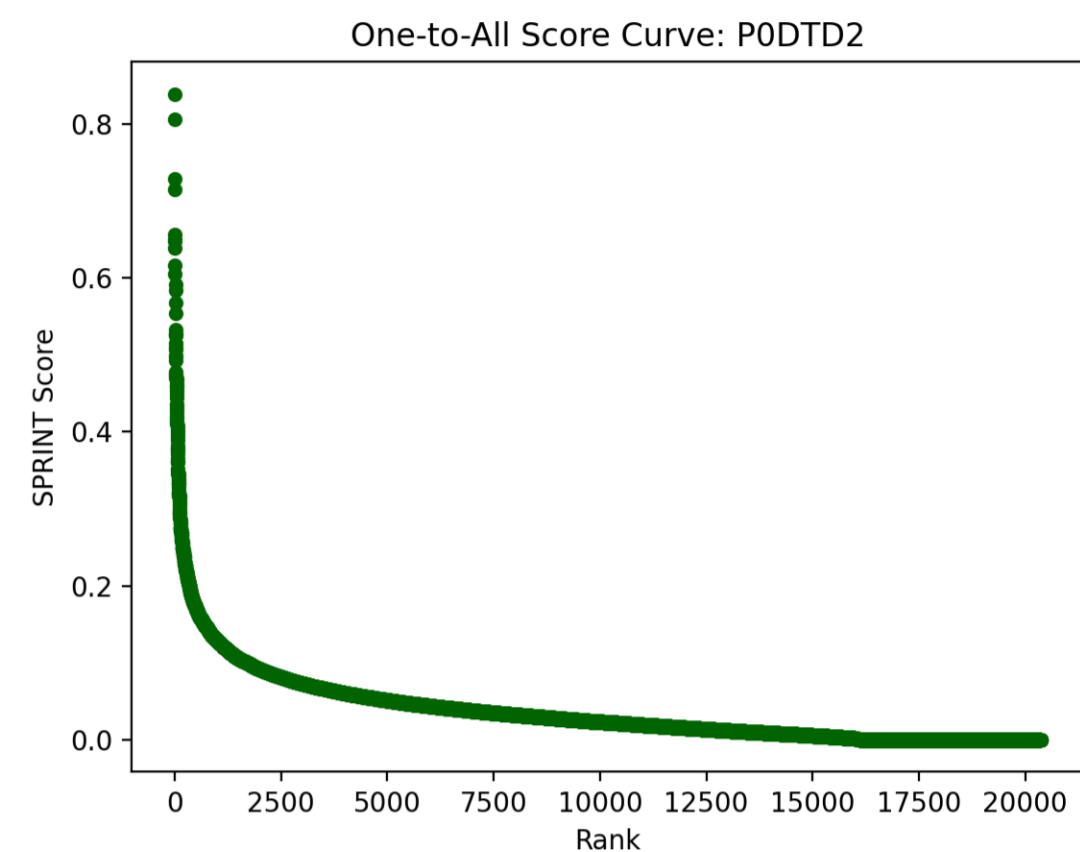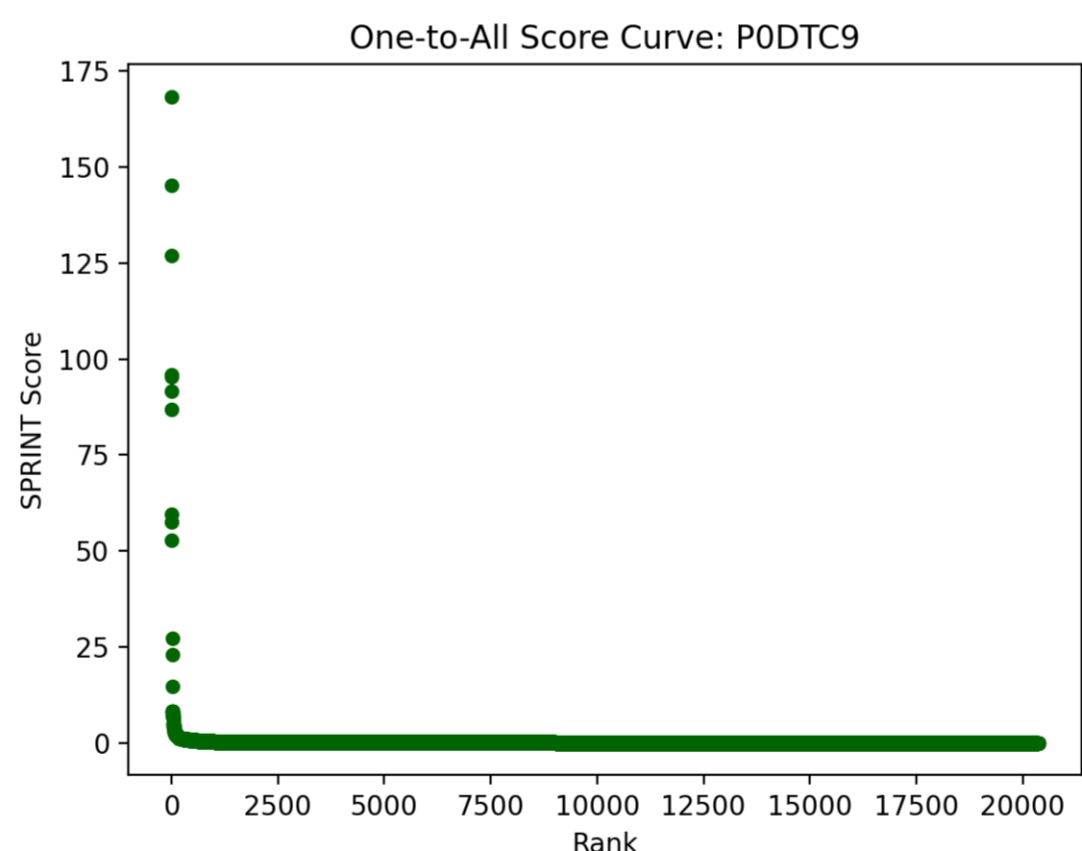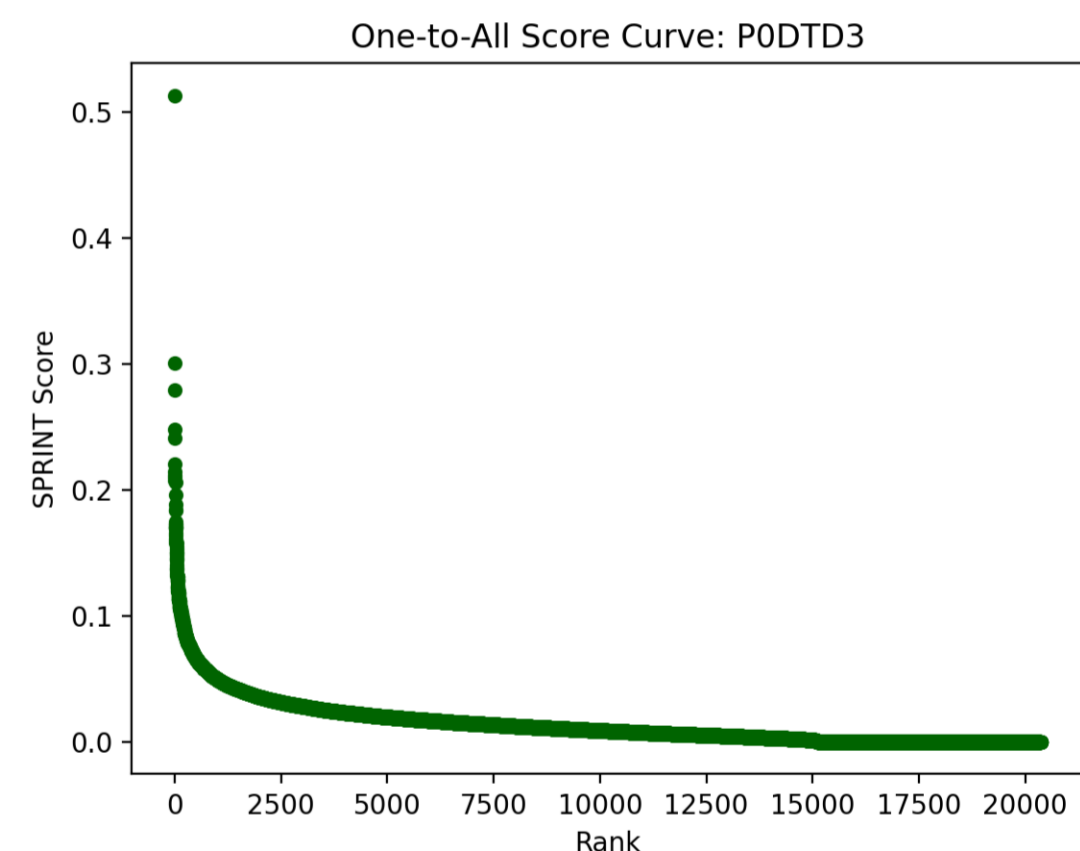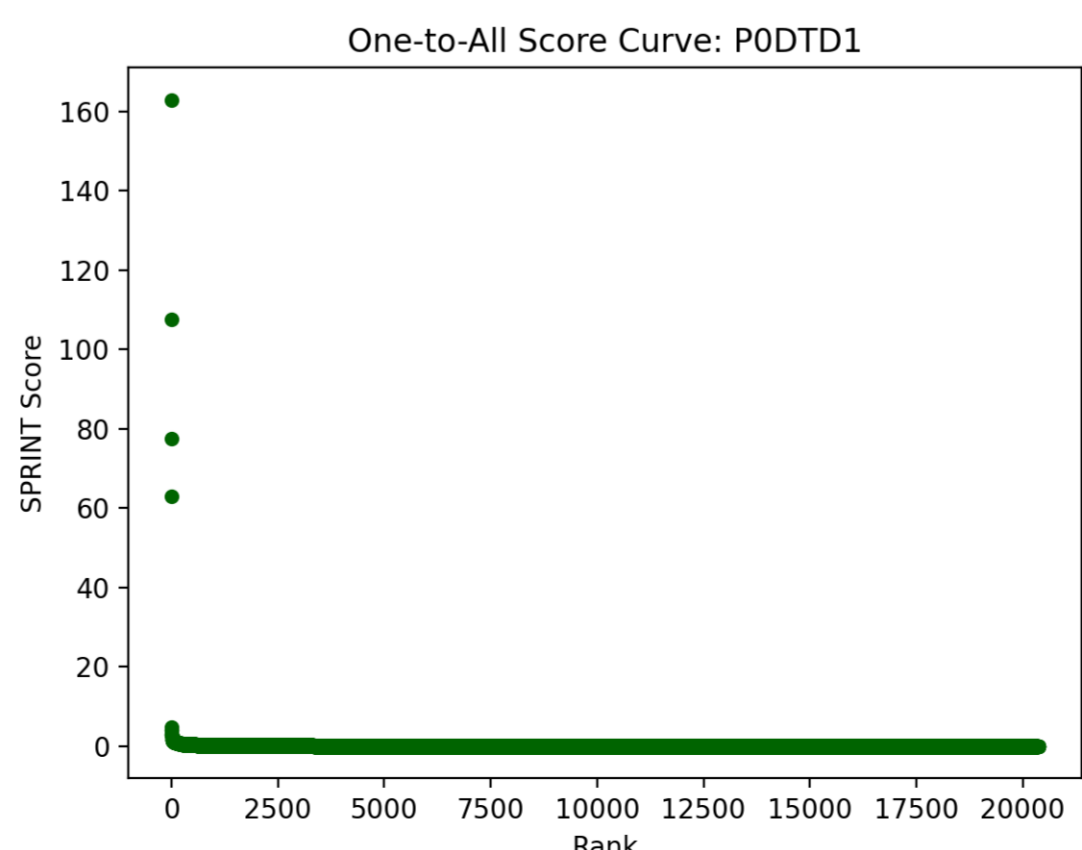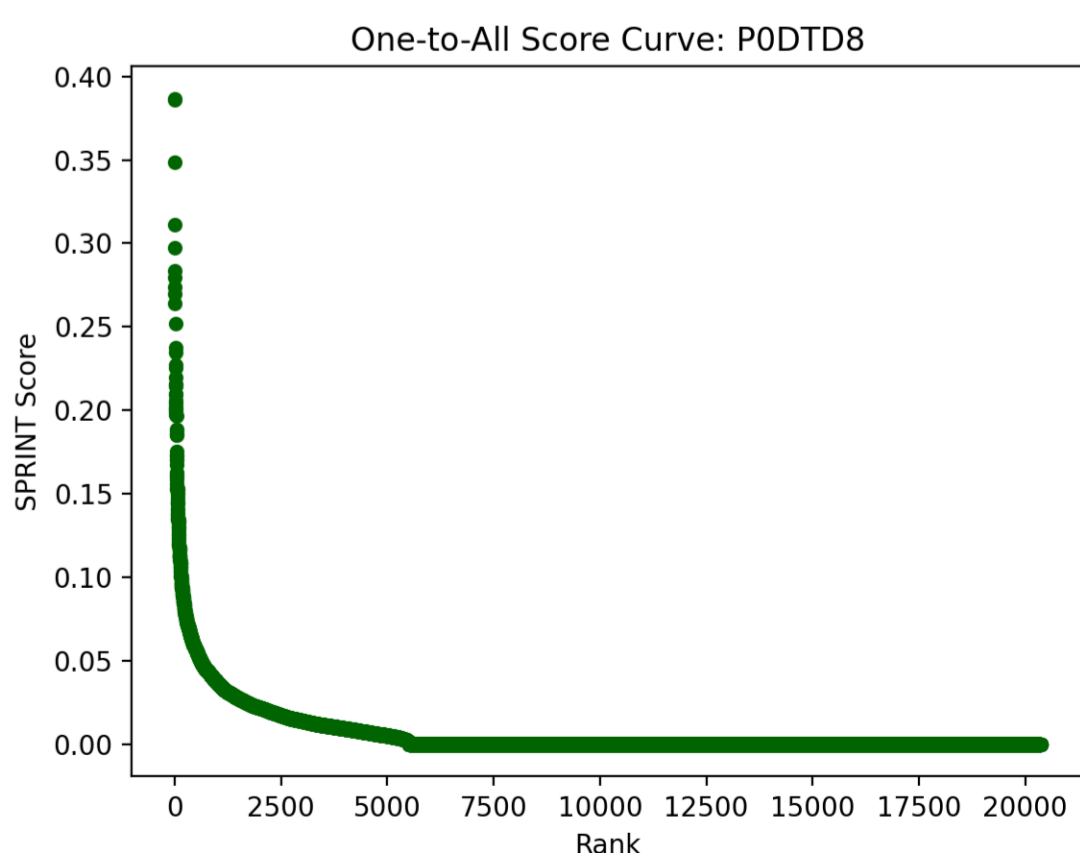

Supplement: Supplemental Information 11 — Each of the subplots depicts a characteristic “L”-shape, where there are a relatively small number of high-scoring pairs as compared to a large number of low-scoring pairs within the baseline. Note that the y-axes are not shared among subplots. [file peerj-09-11117-s011.pdf]

# SPRINT

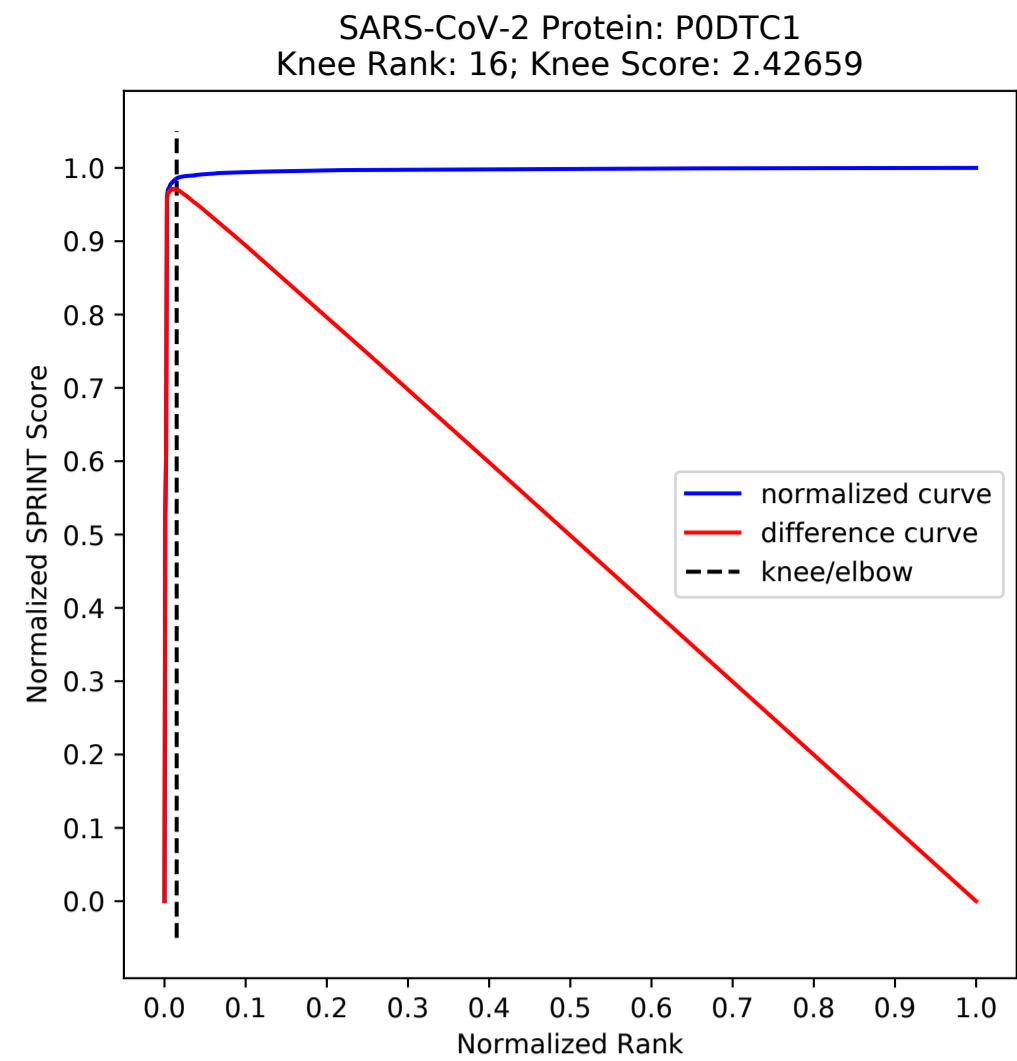

# PIPE4

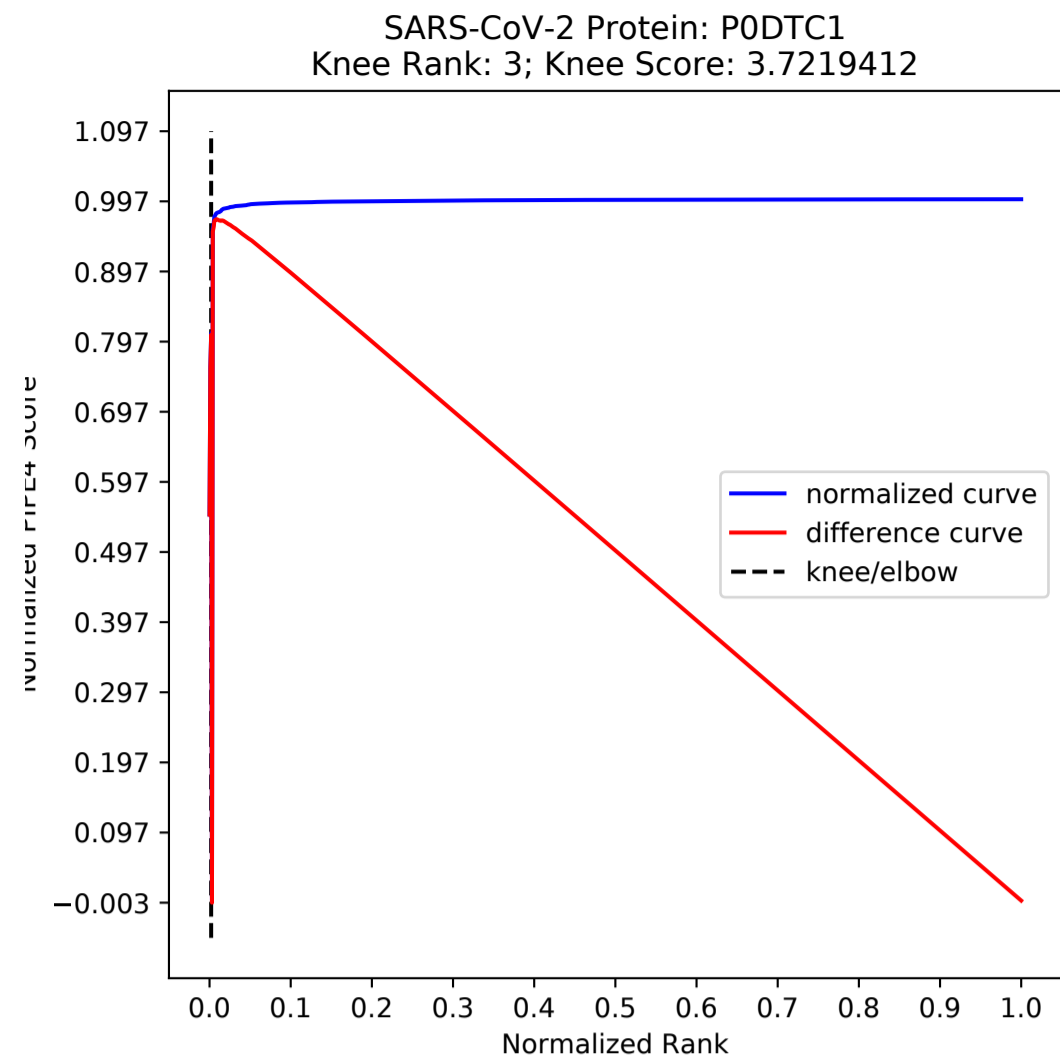

# PIPE4

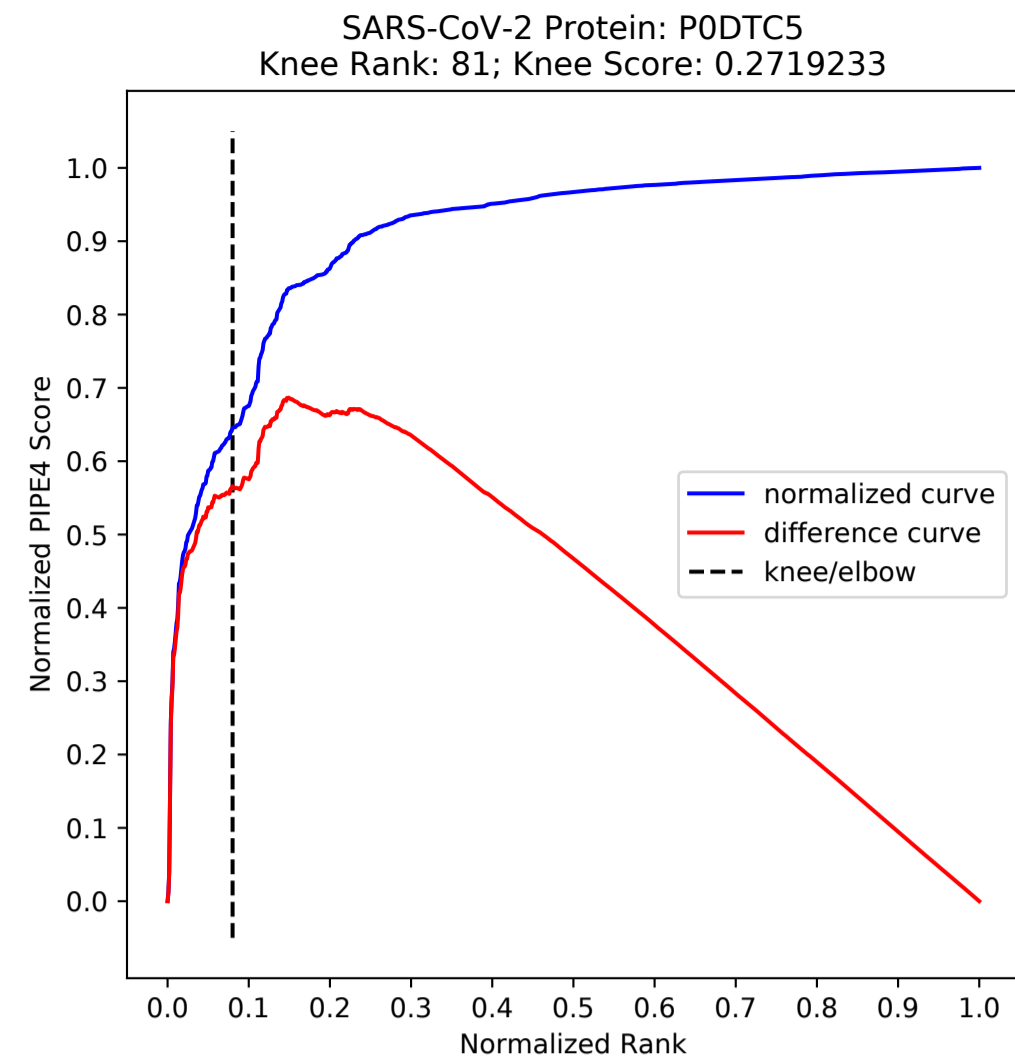

# SPRINT

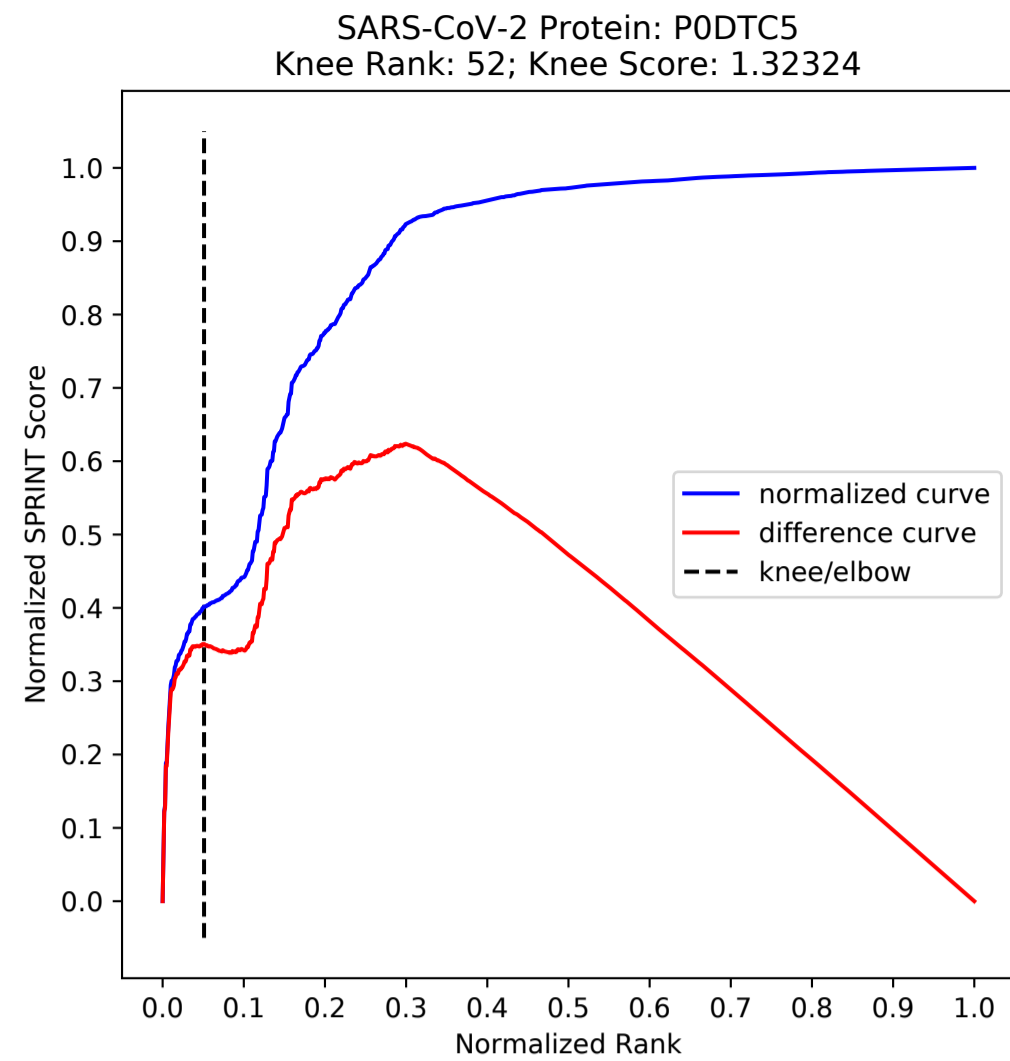

# SPRINT

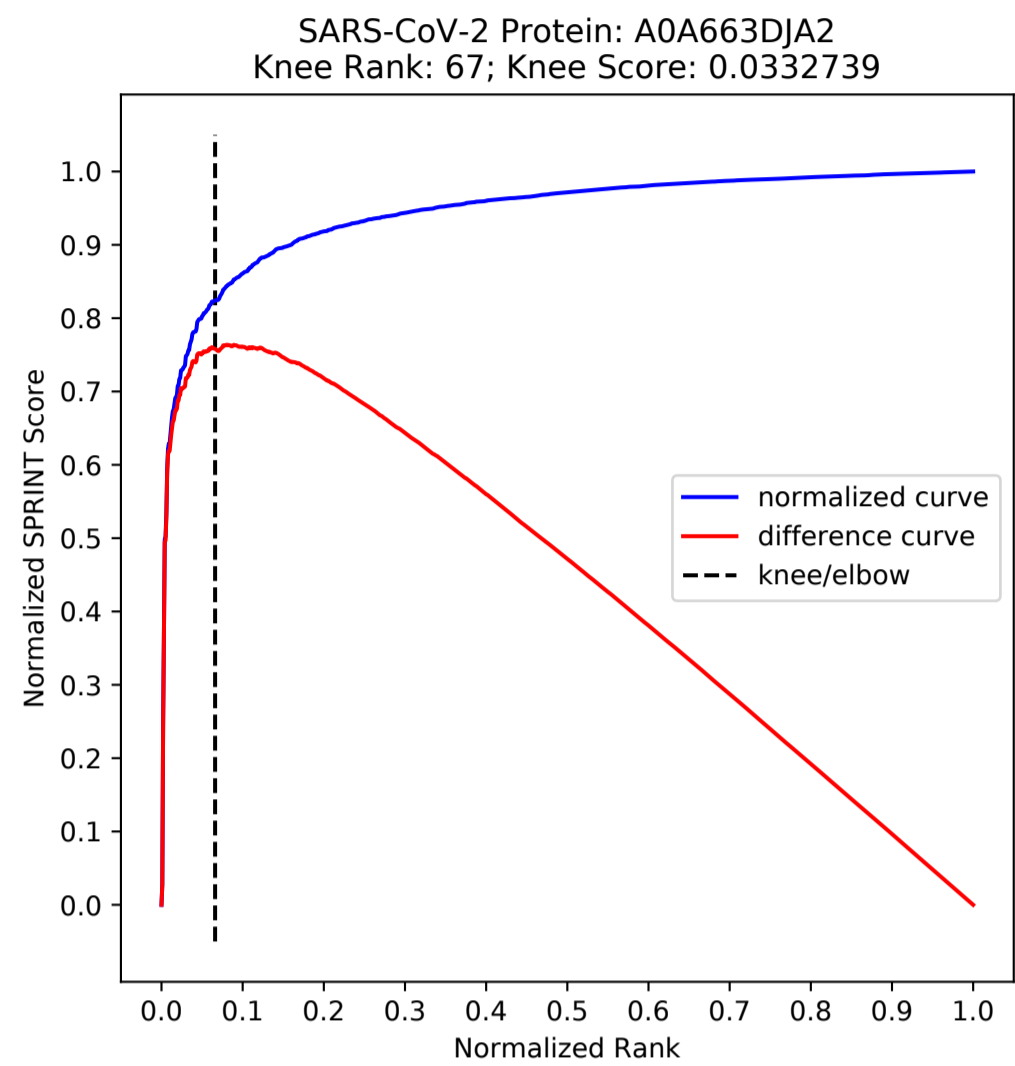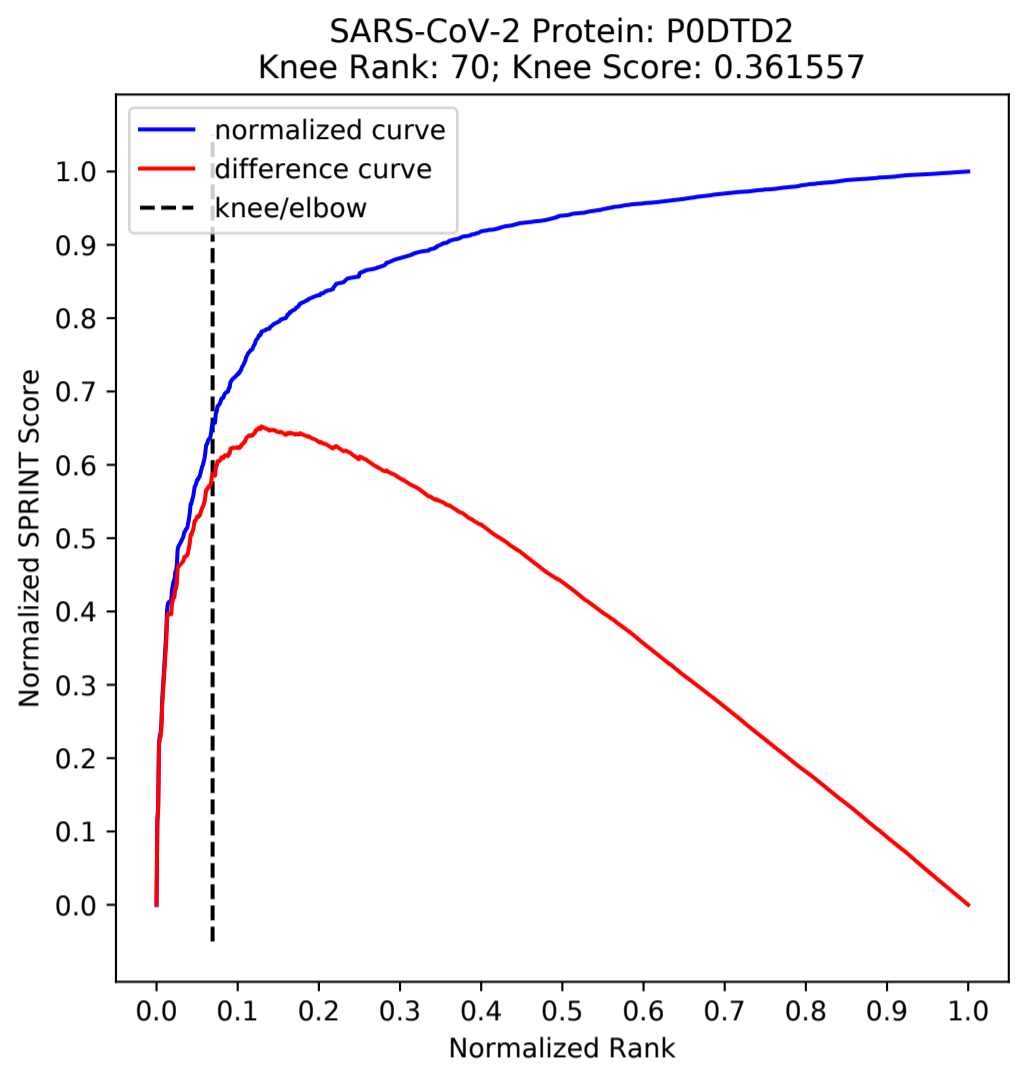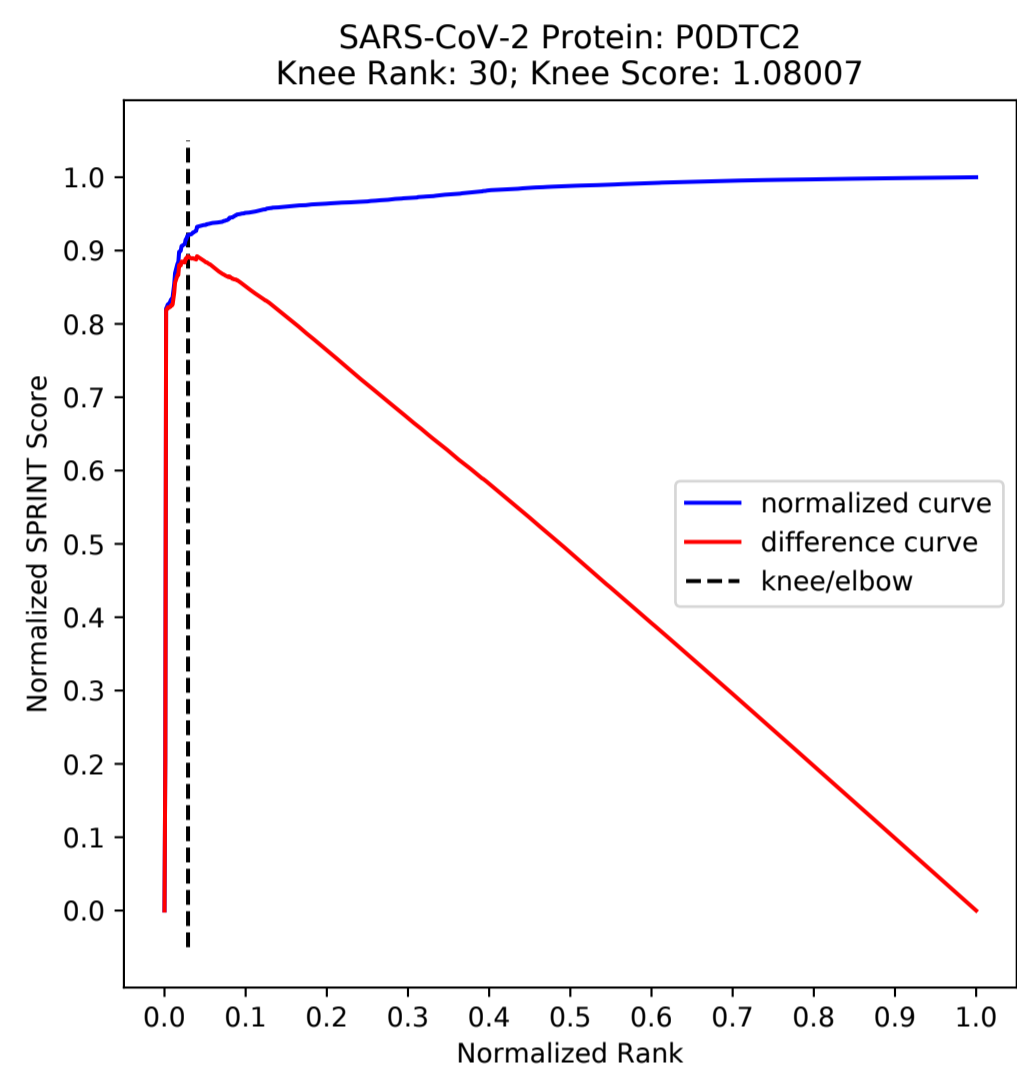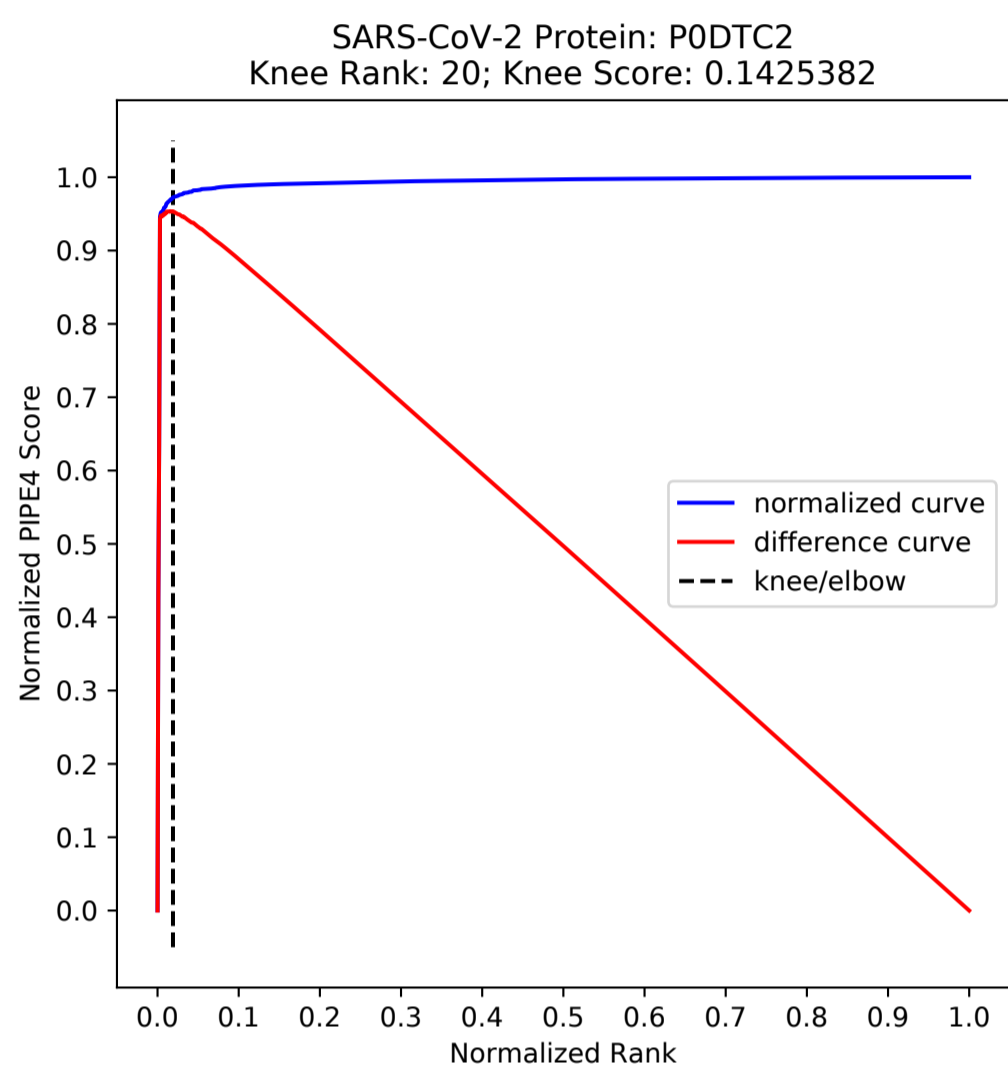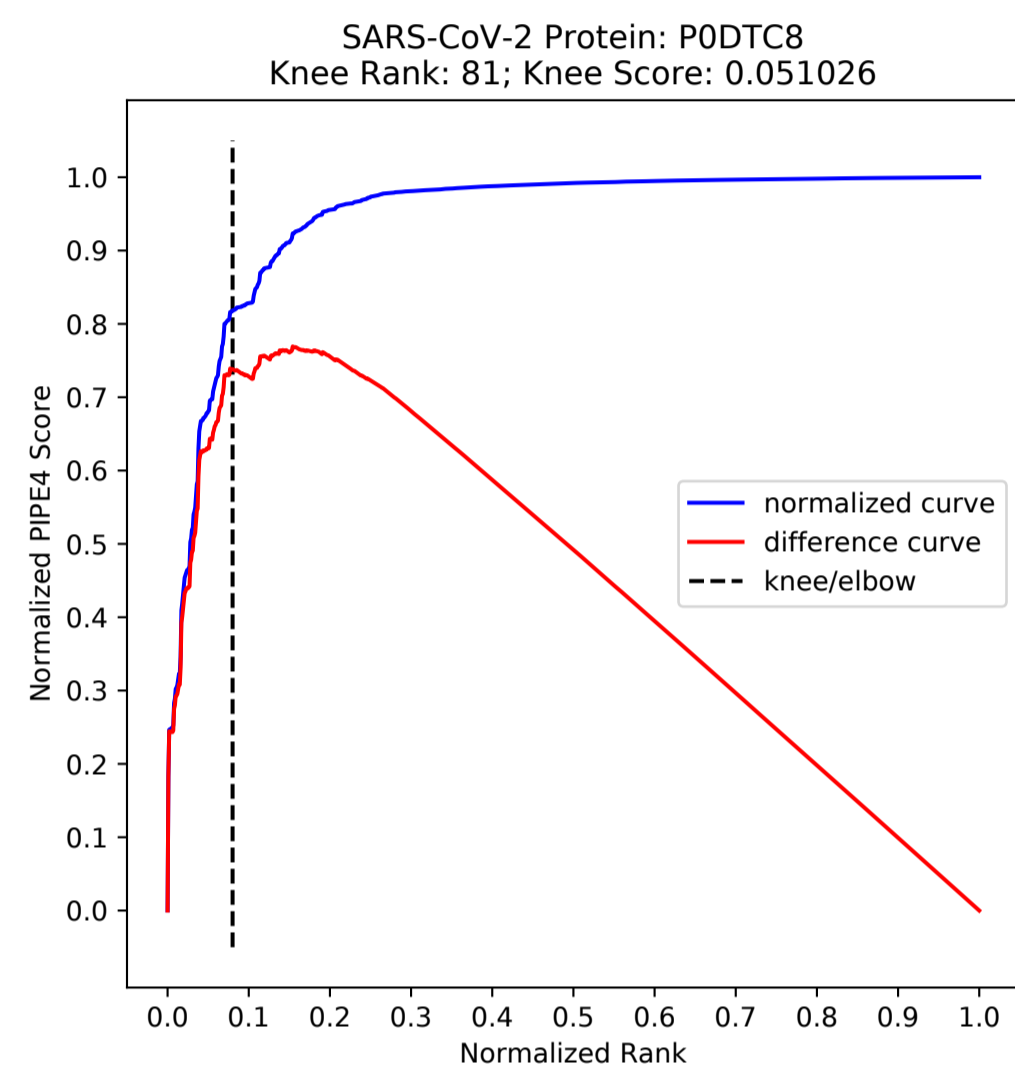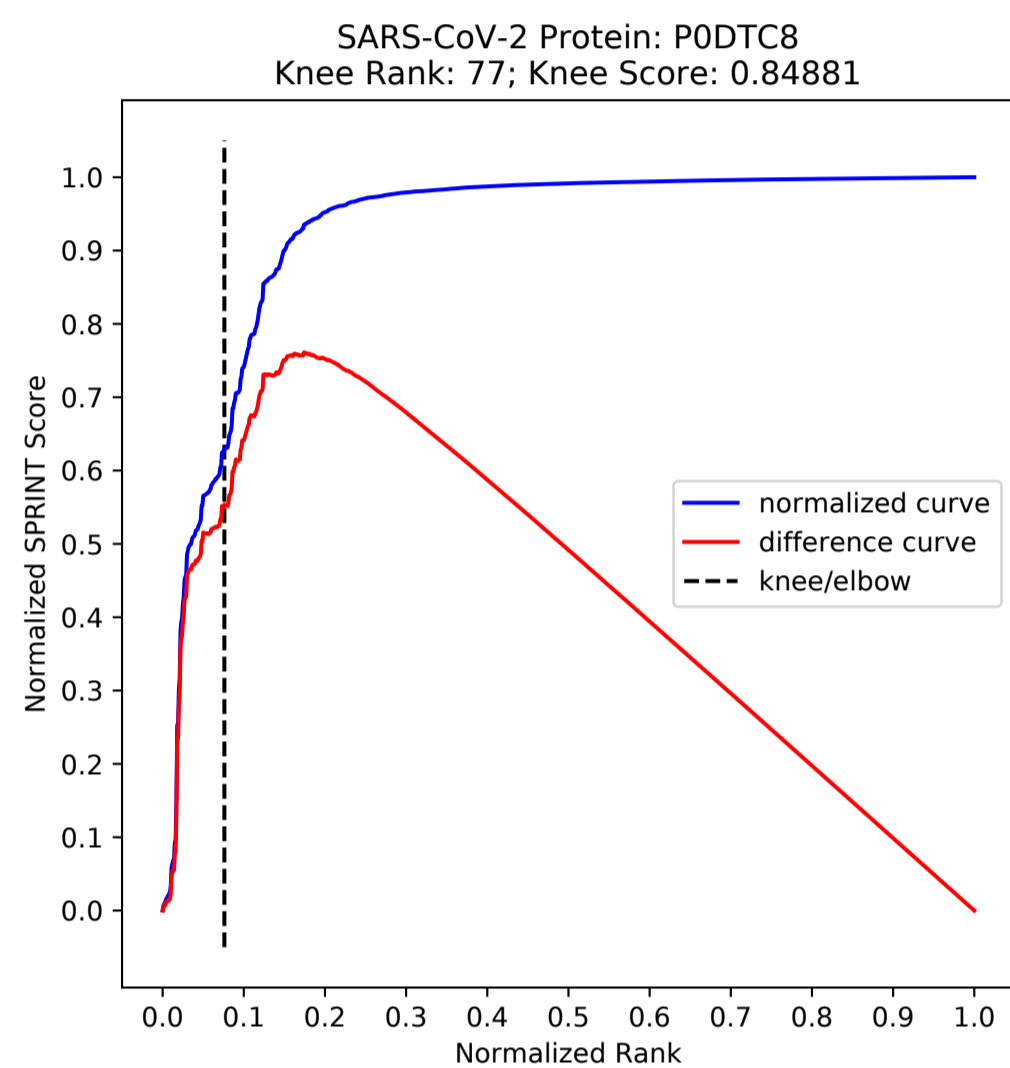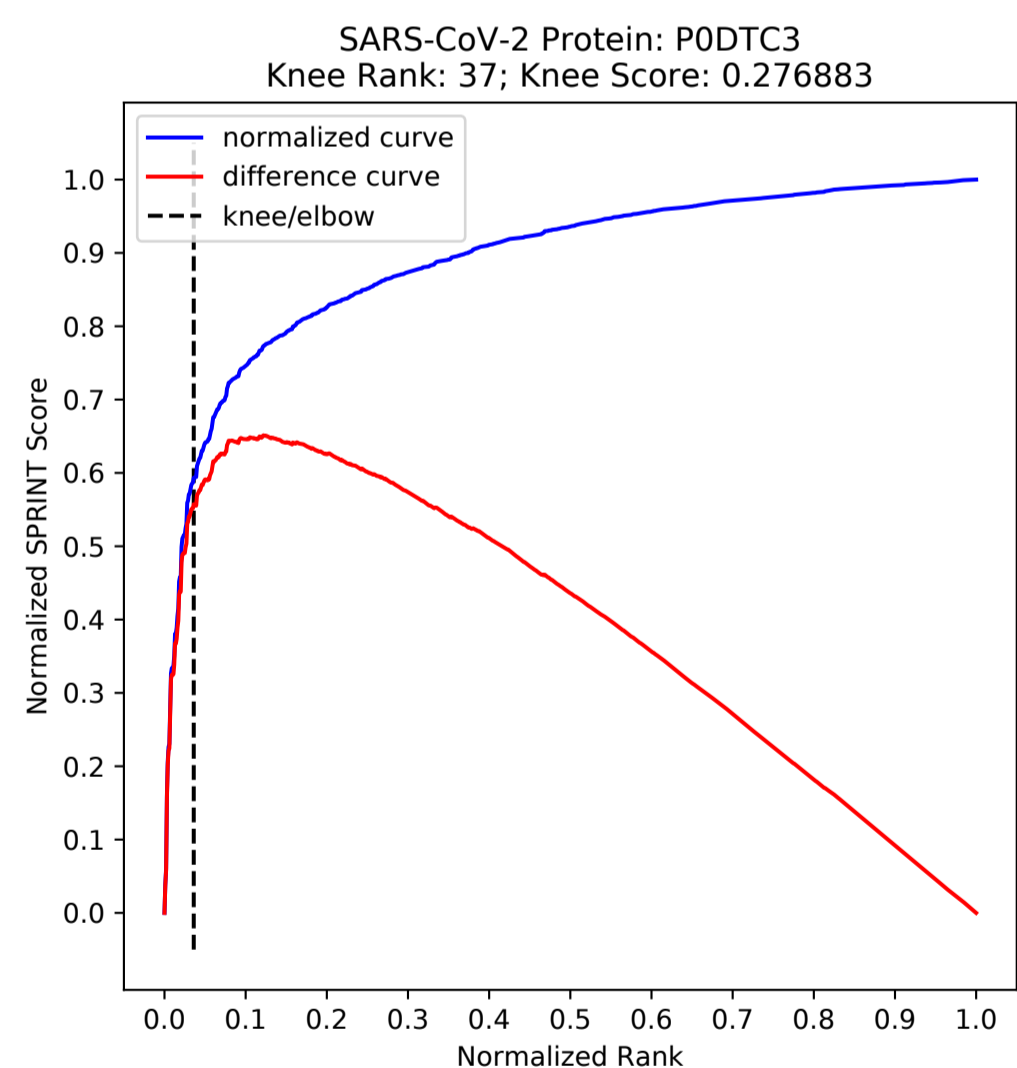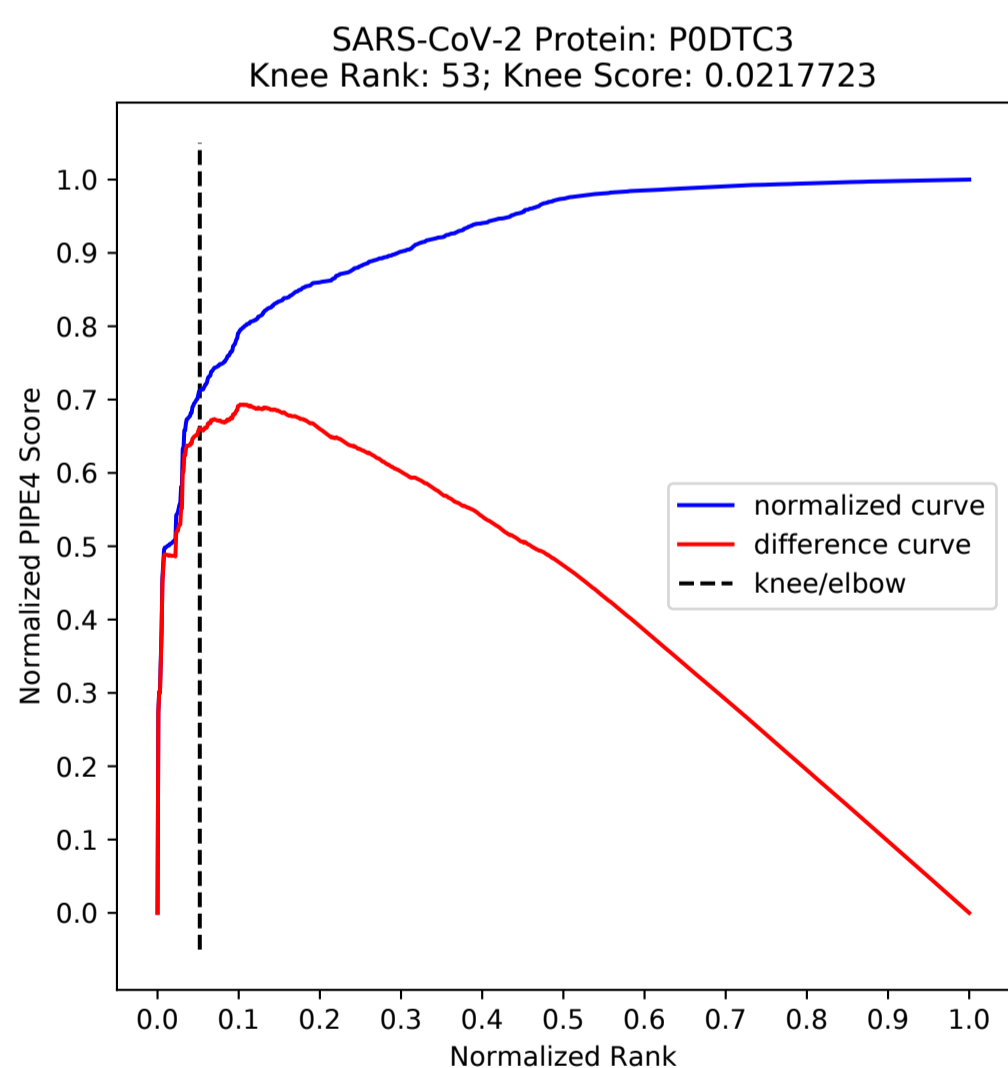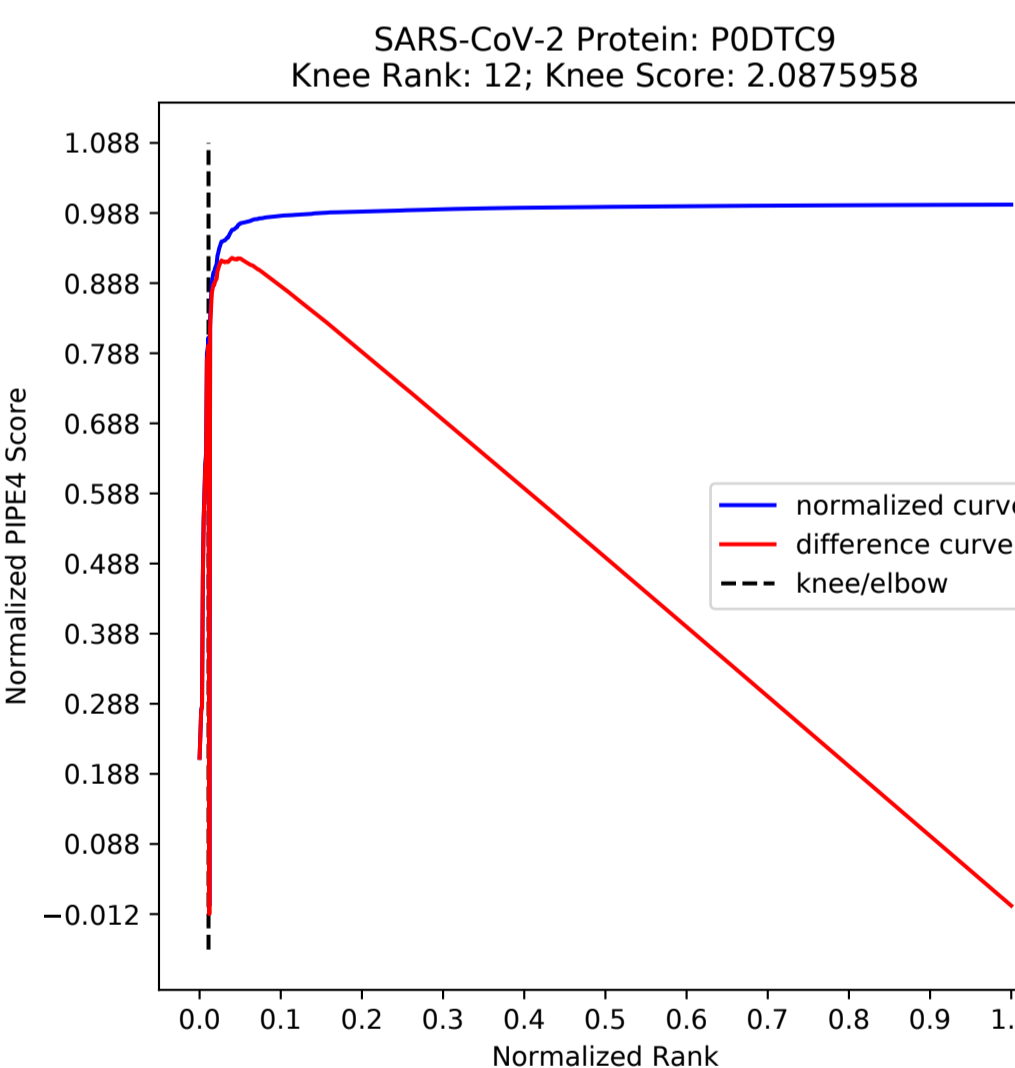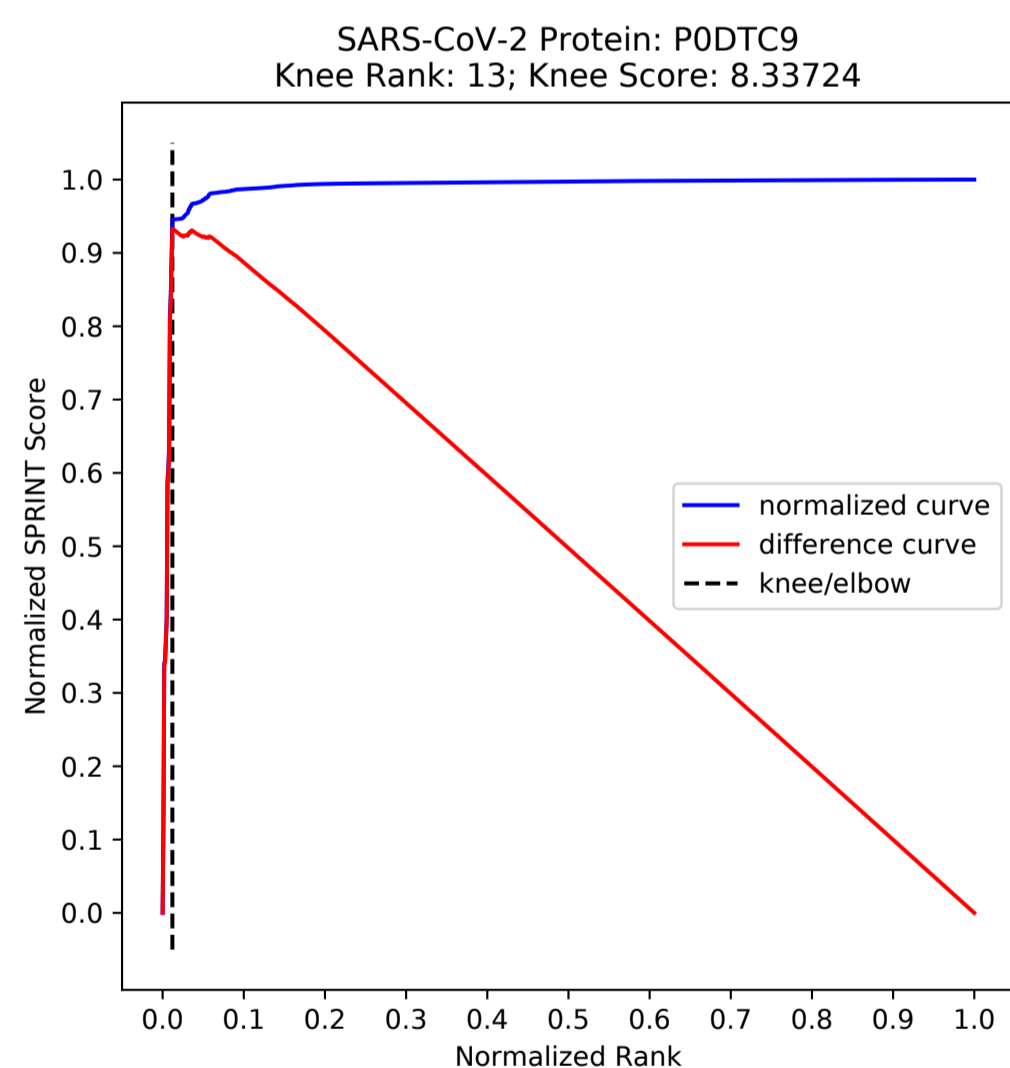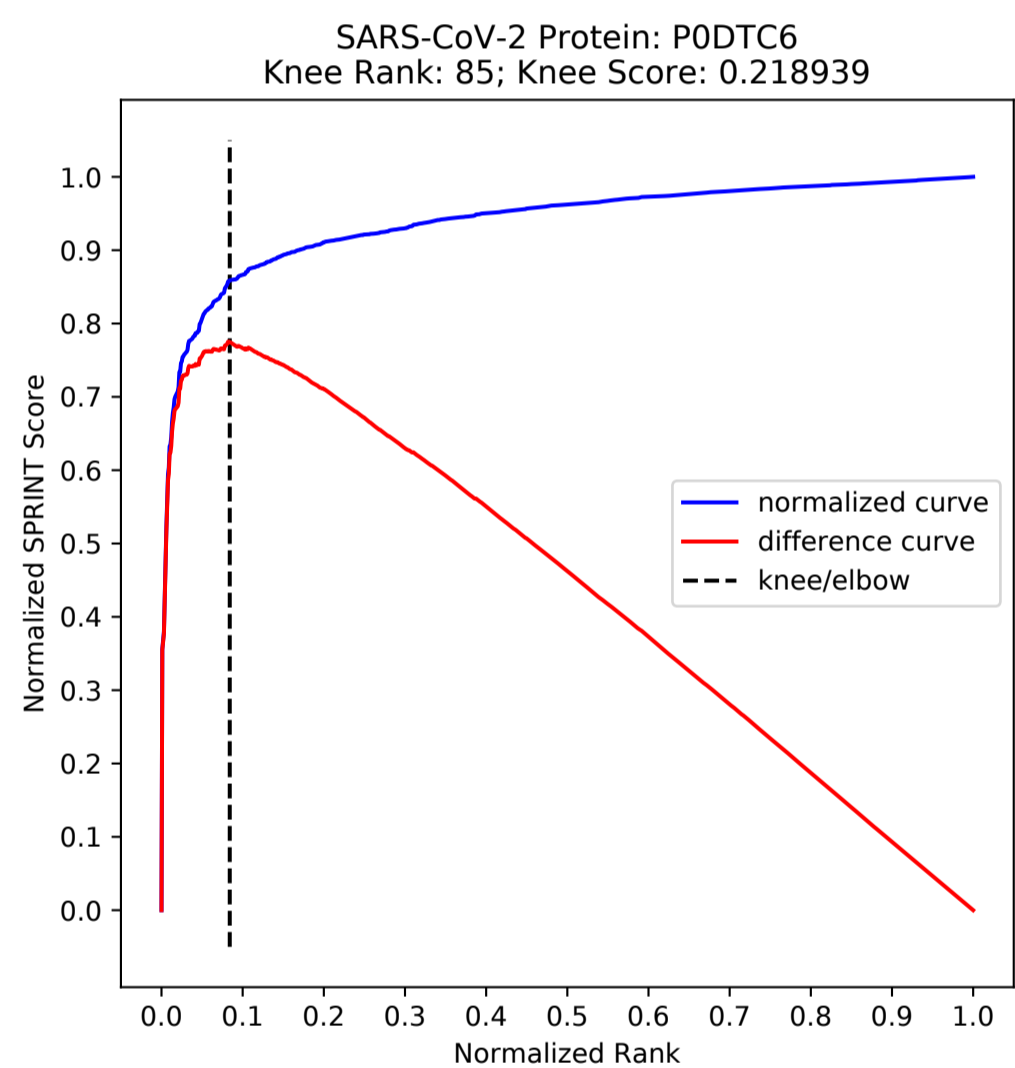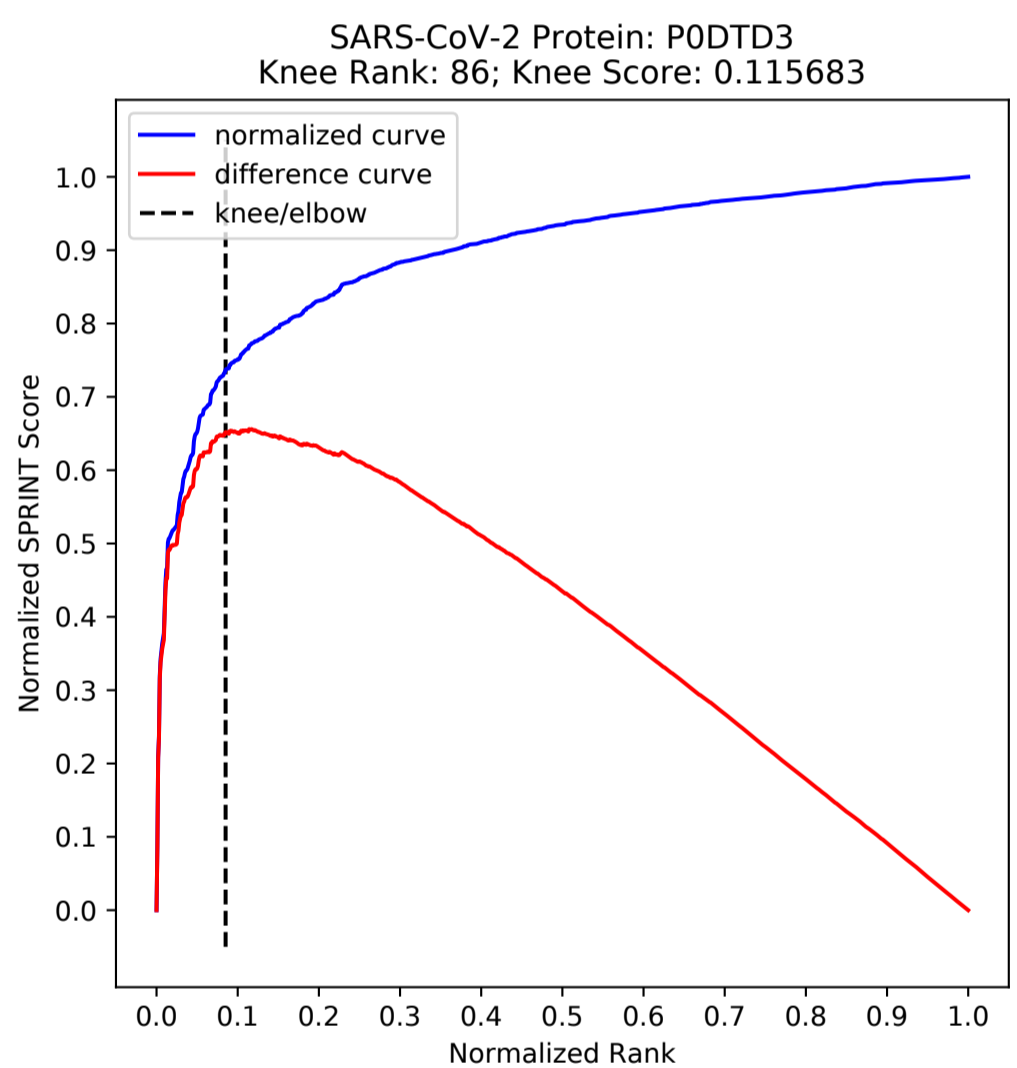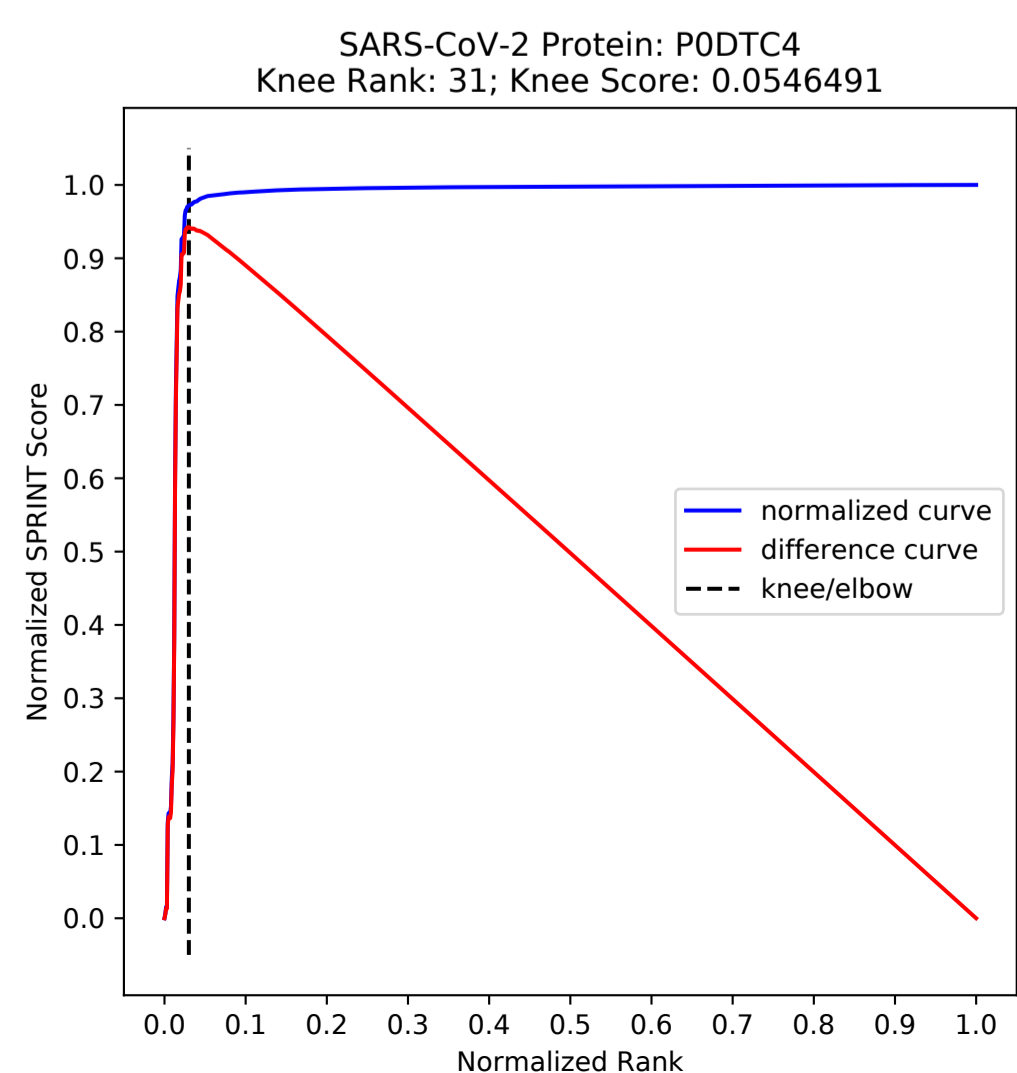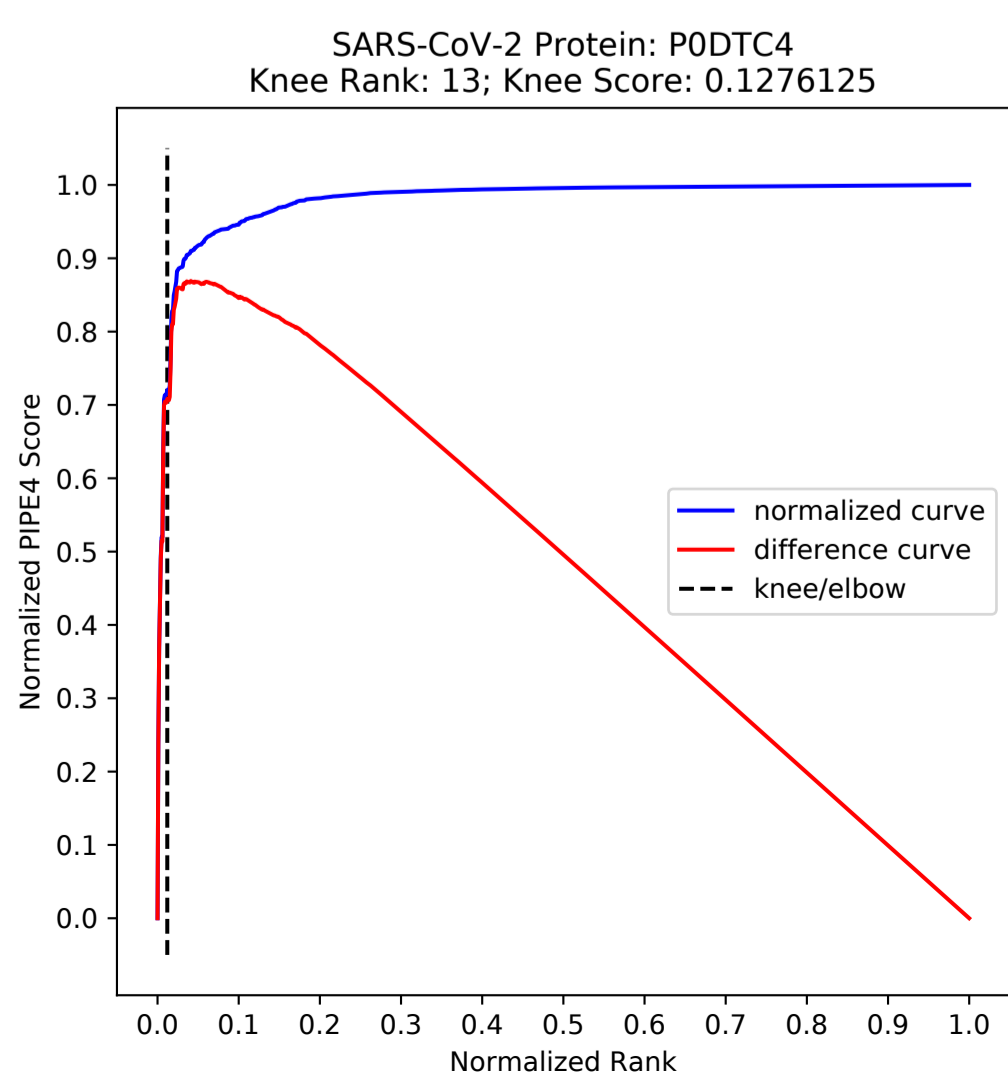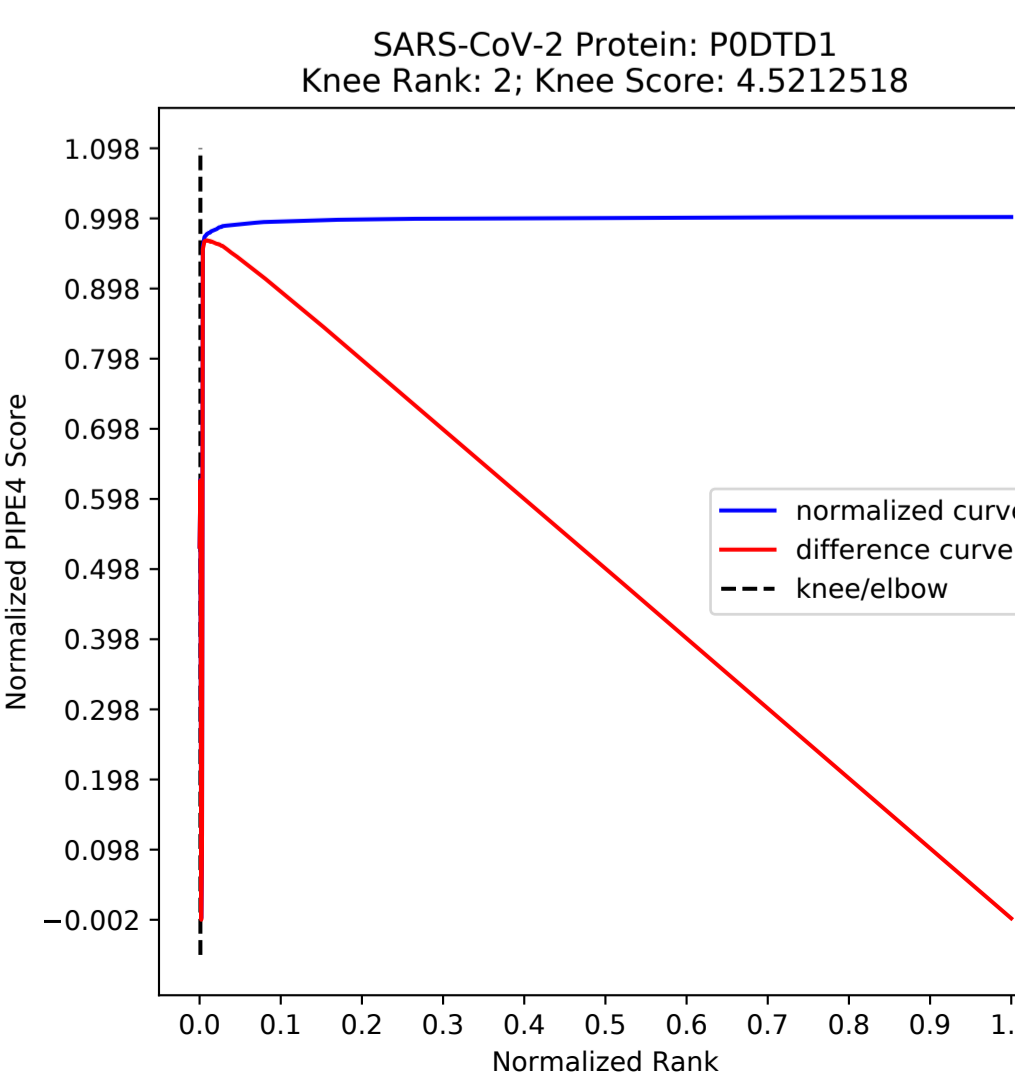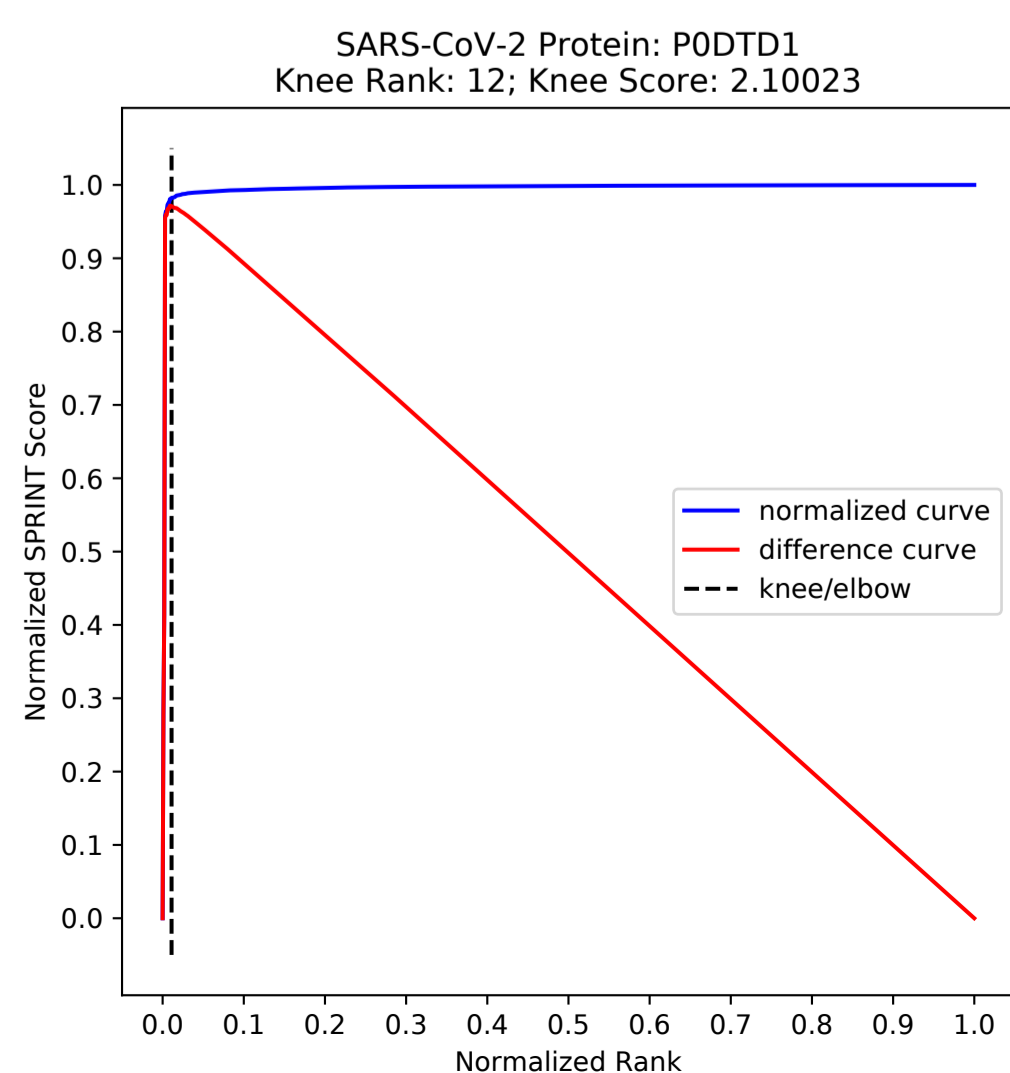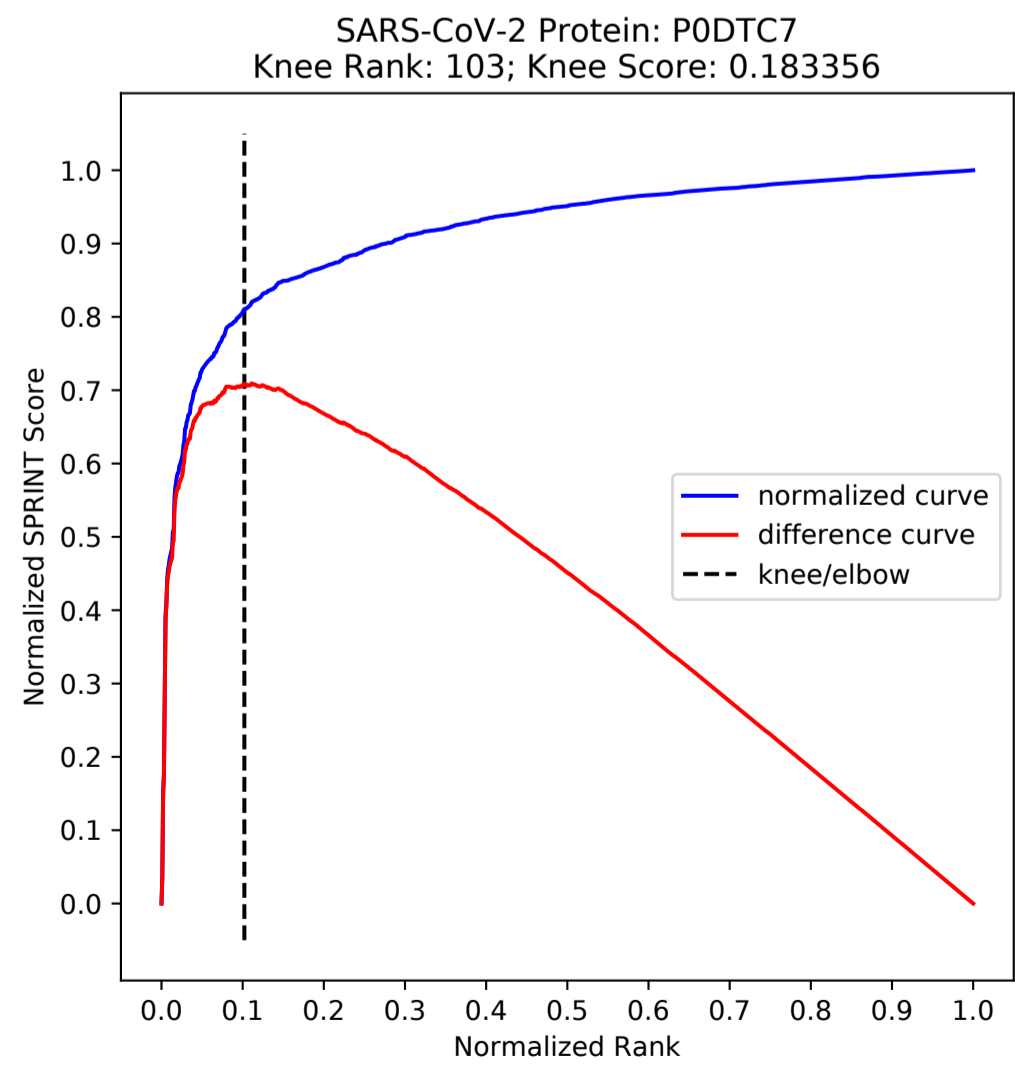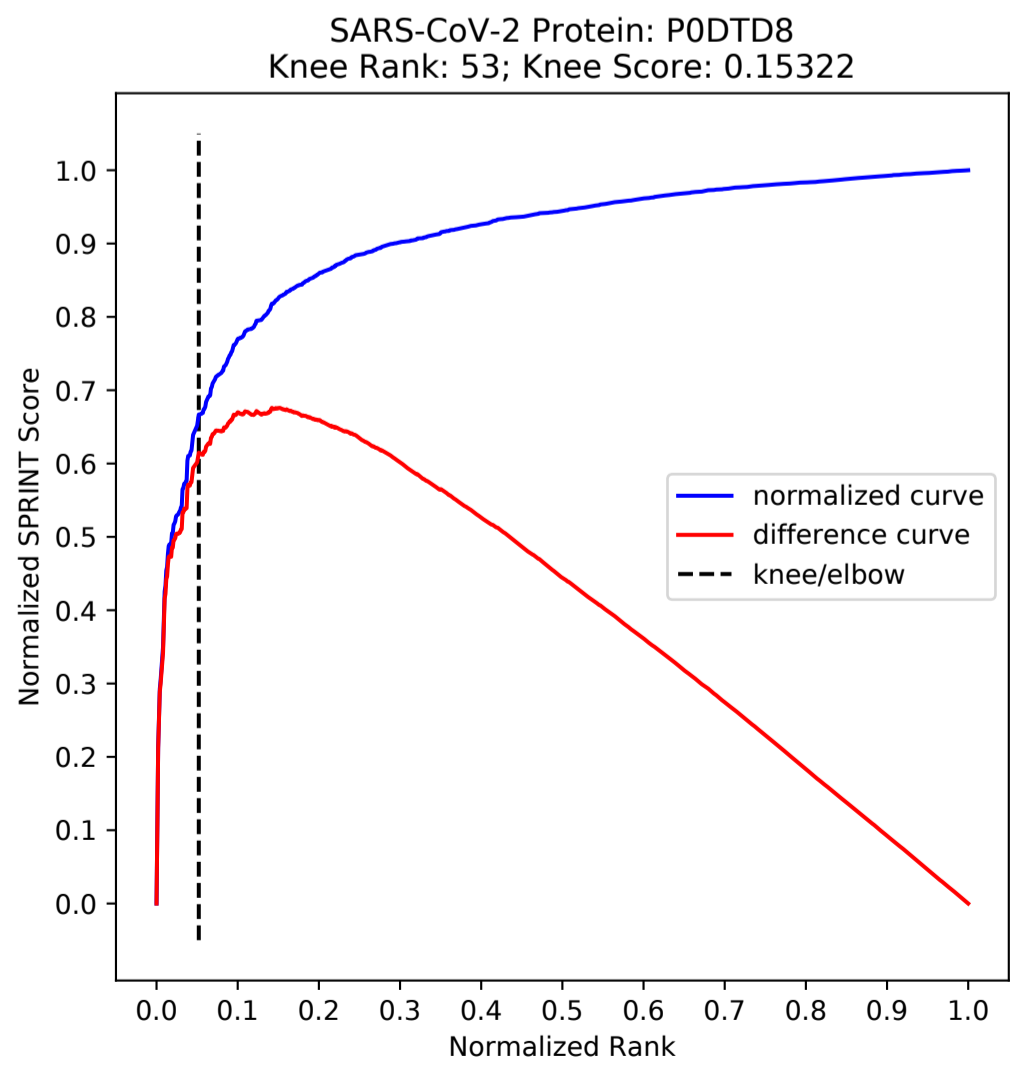

Supplement: Supplemental Information 12 — Each of the subplots highlights the detected knee of the normalized top-1000 predictions obtained using the Kneedle algorithm. The differences curve plots the value obtained from subtracting the perpendicular distance of each point to y=x from the distance of each point vertically to y=x of the normalized plot. The peak of this curve, parameterized by S, estimates the location of the knee. [file peerj-09-11117-s012.pdf]

# SPRINT

# PIPE4

# PIPE4

# SPRINT

# SPRINT

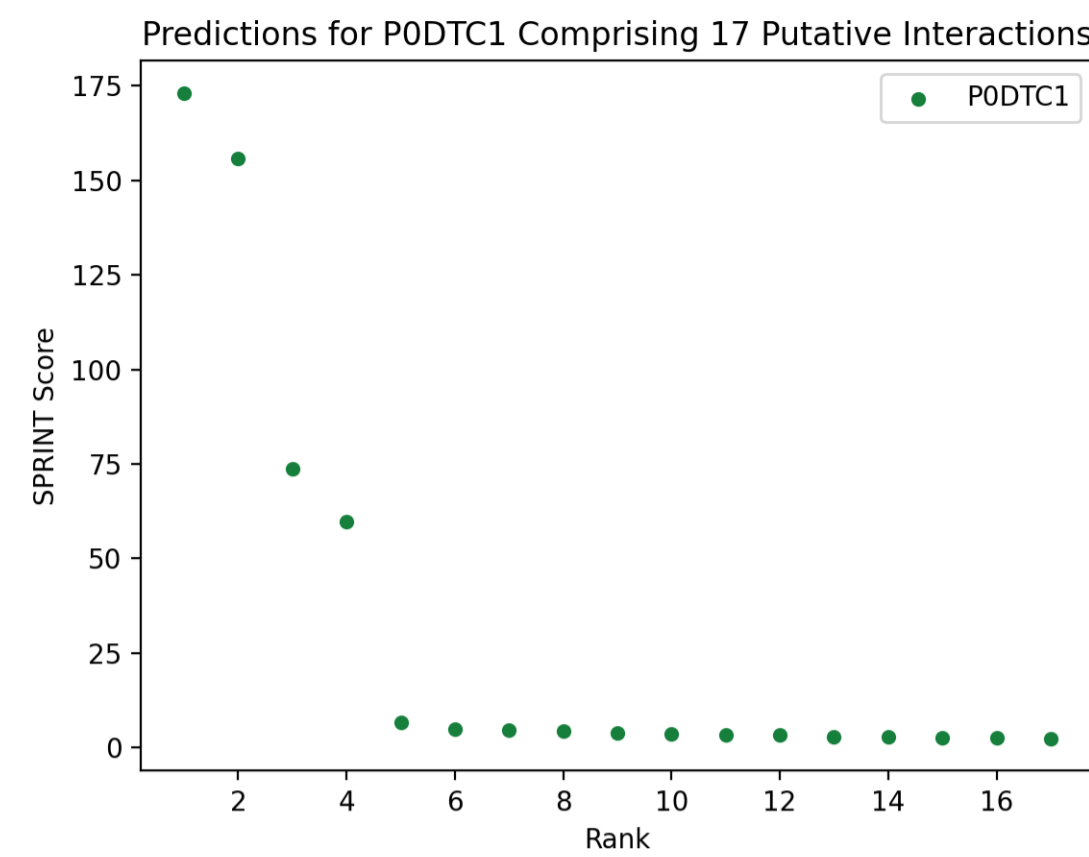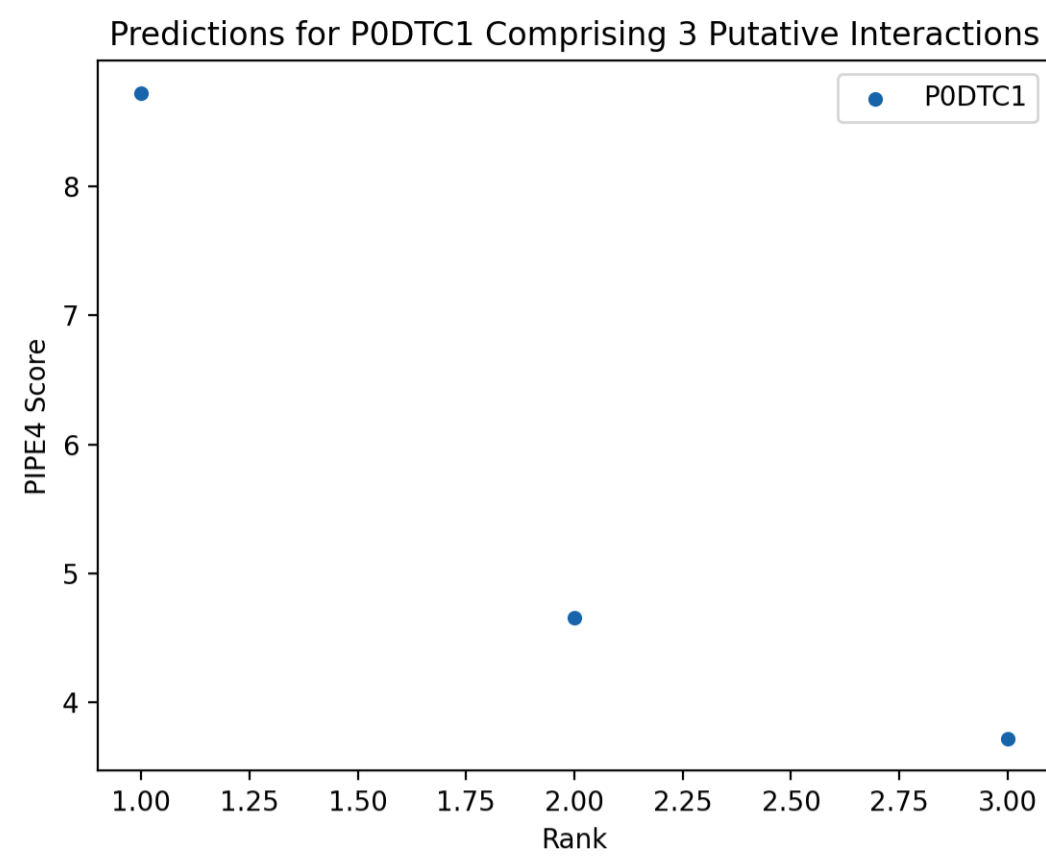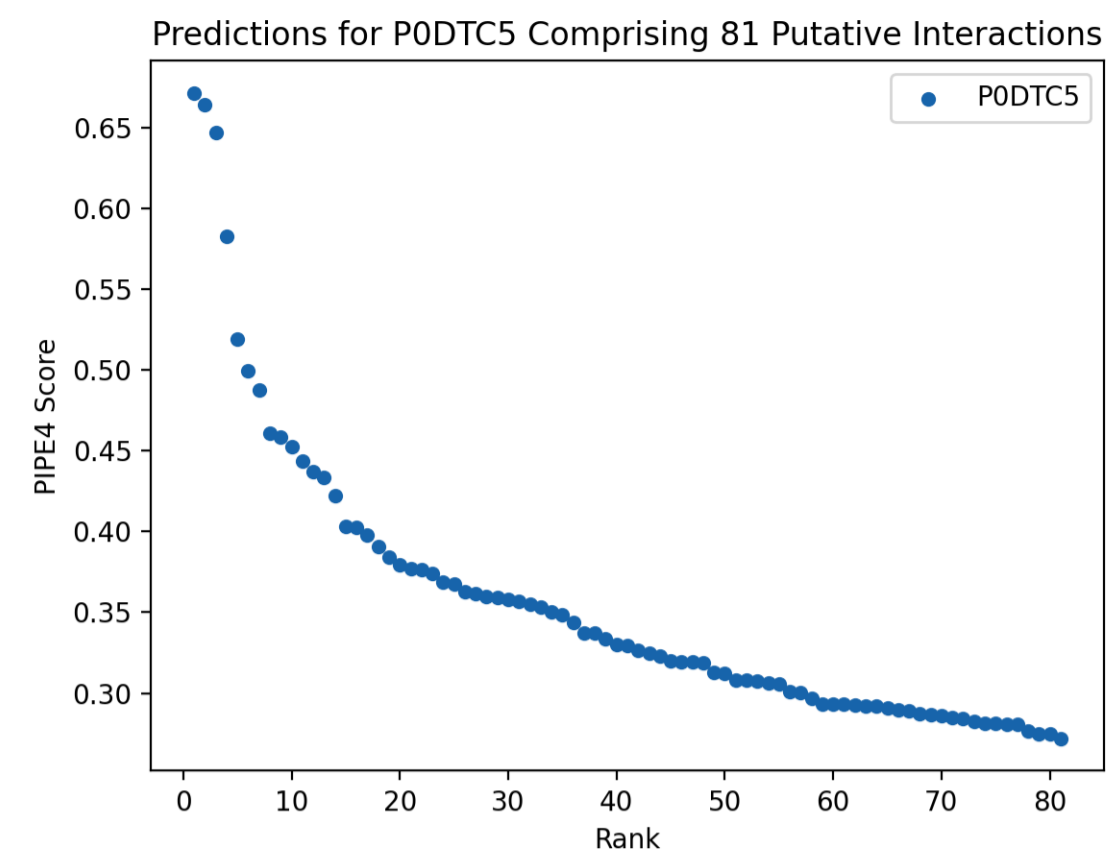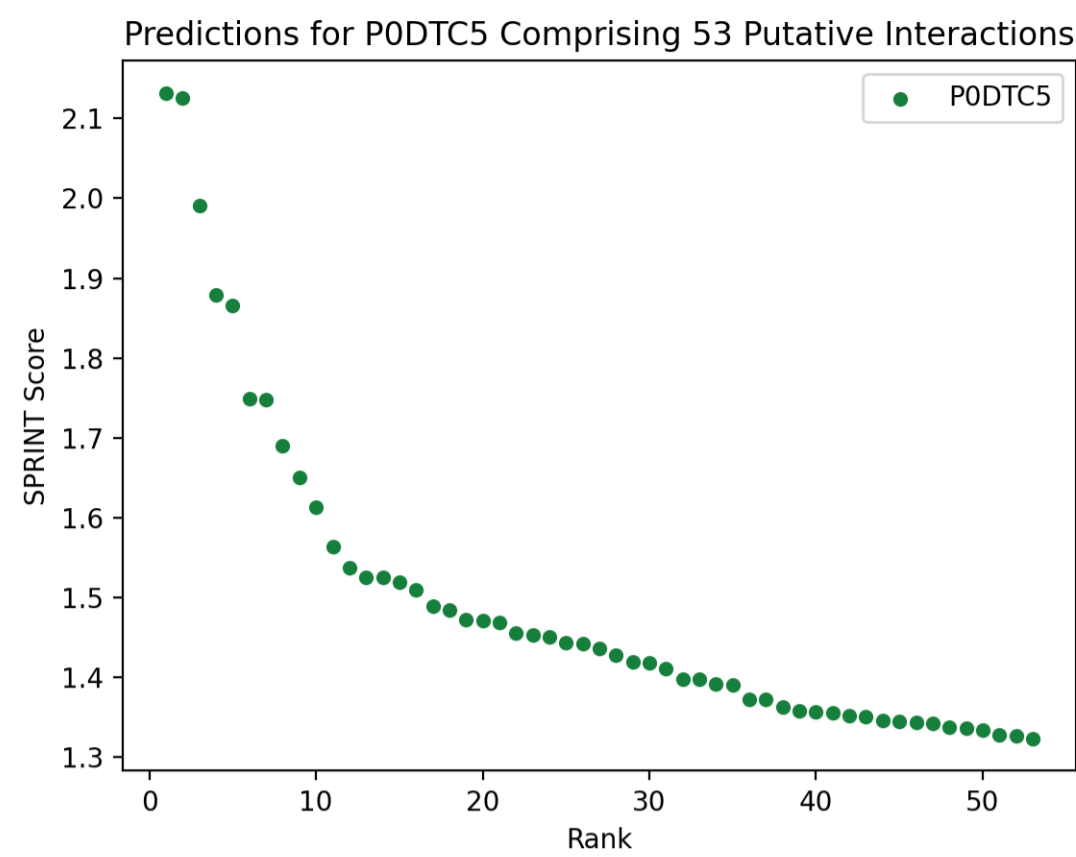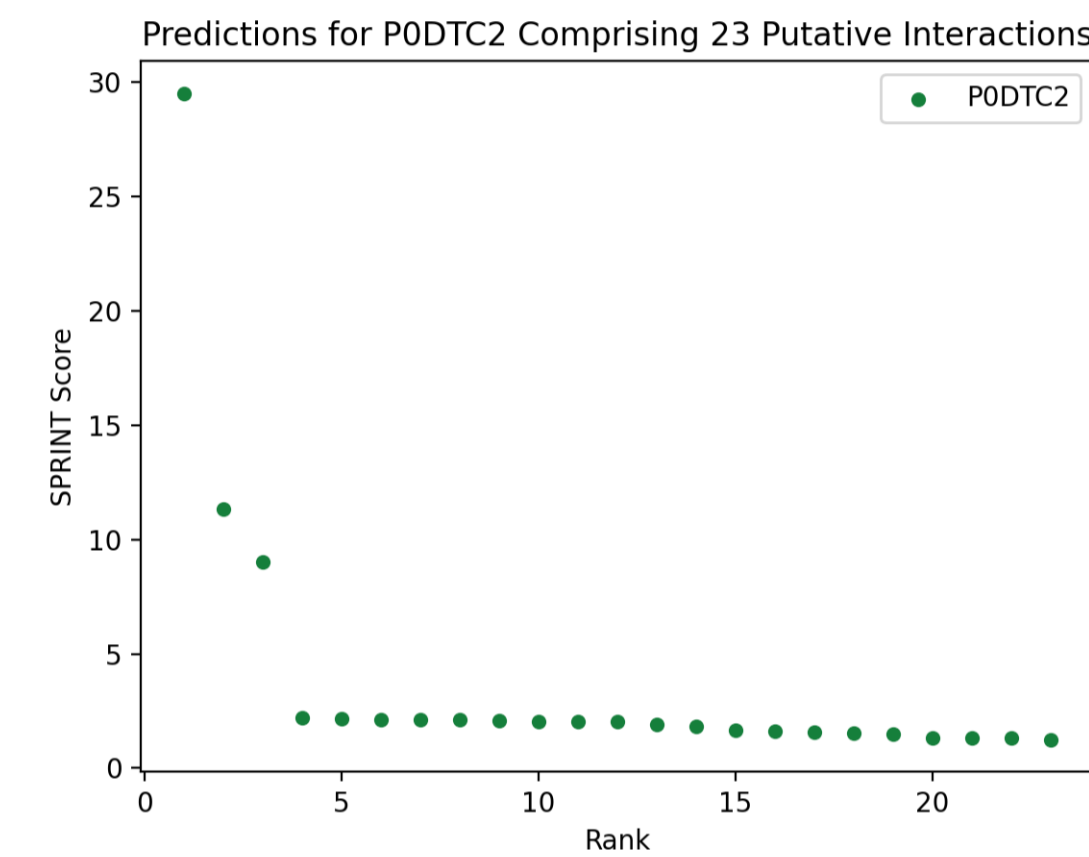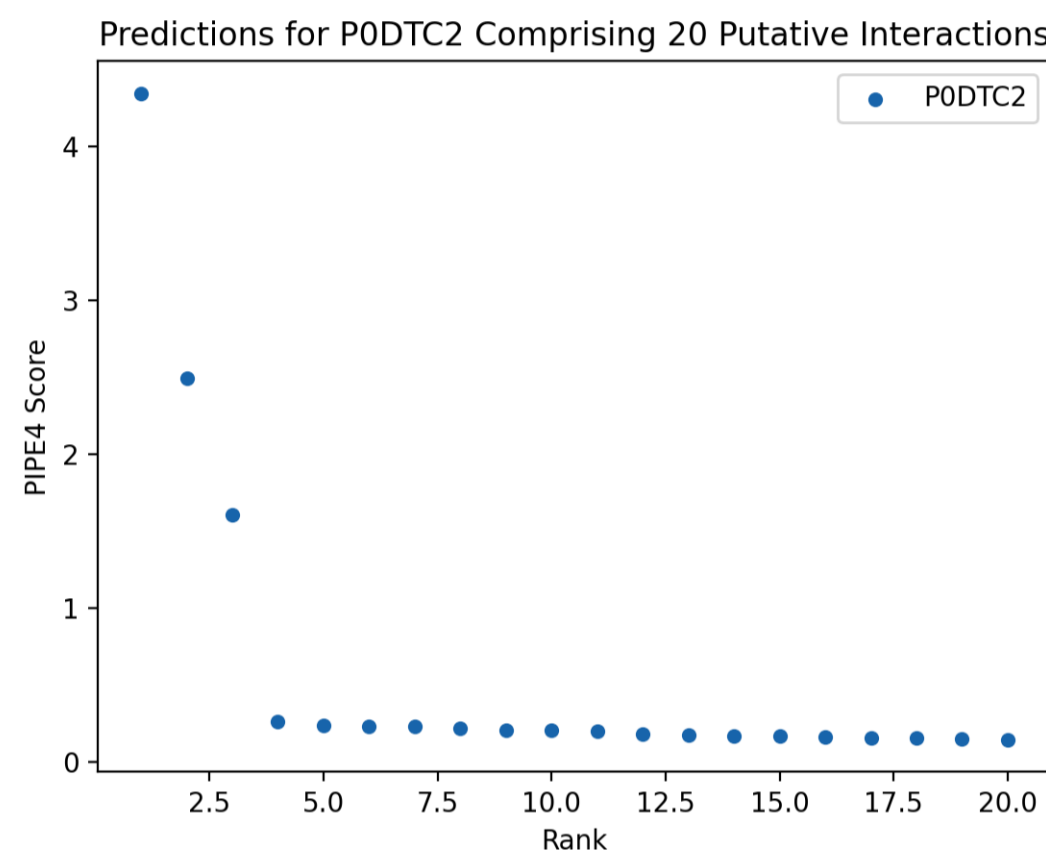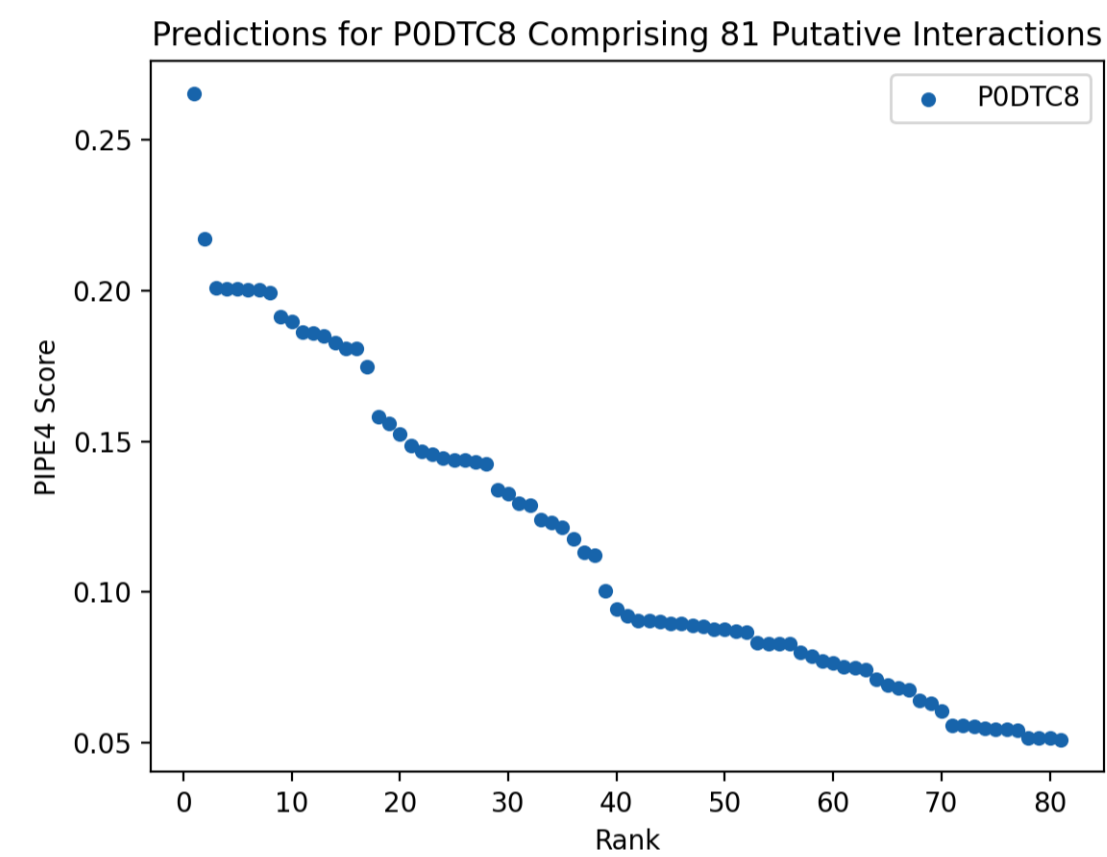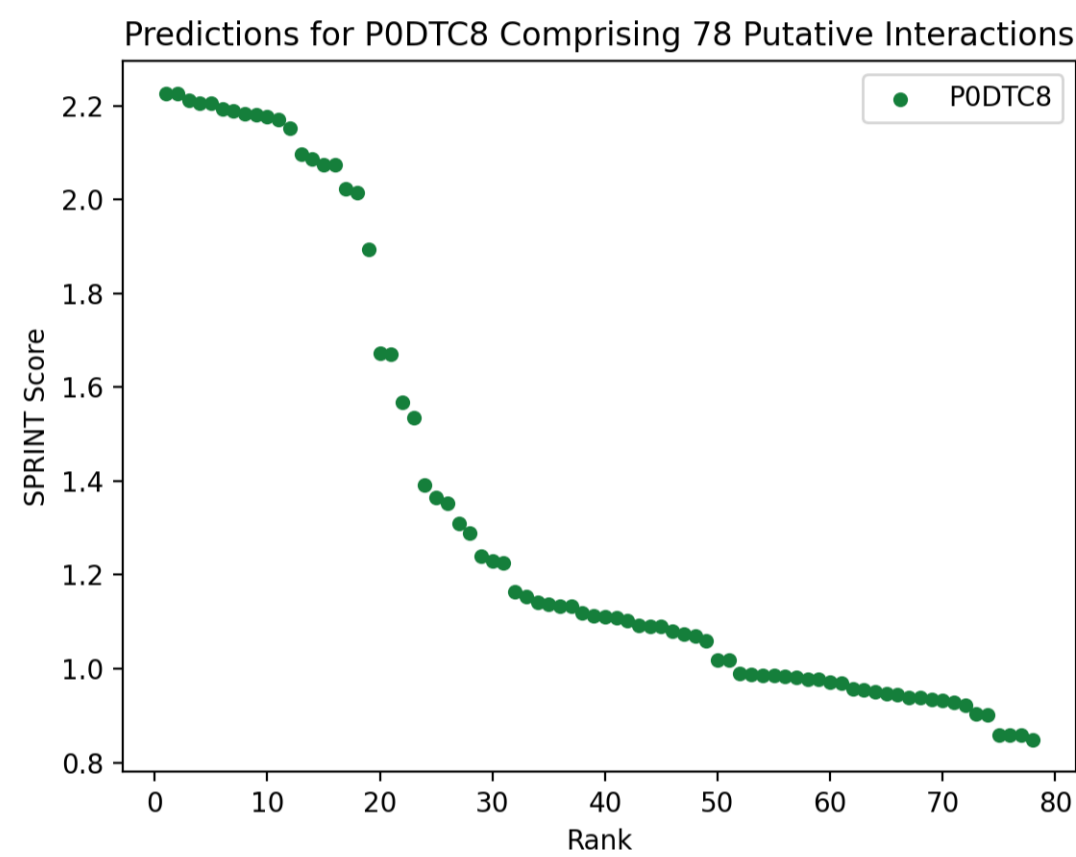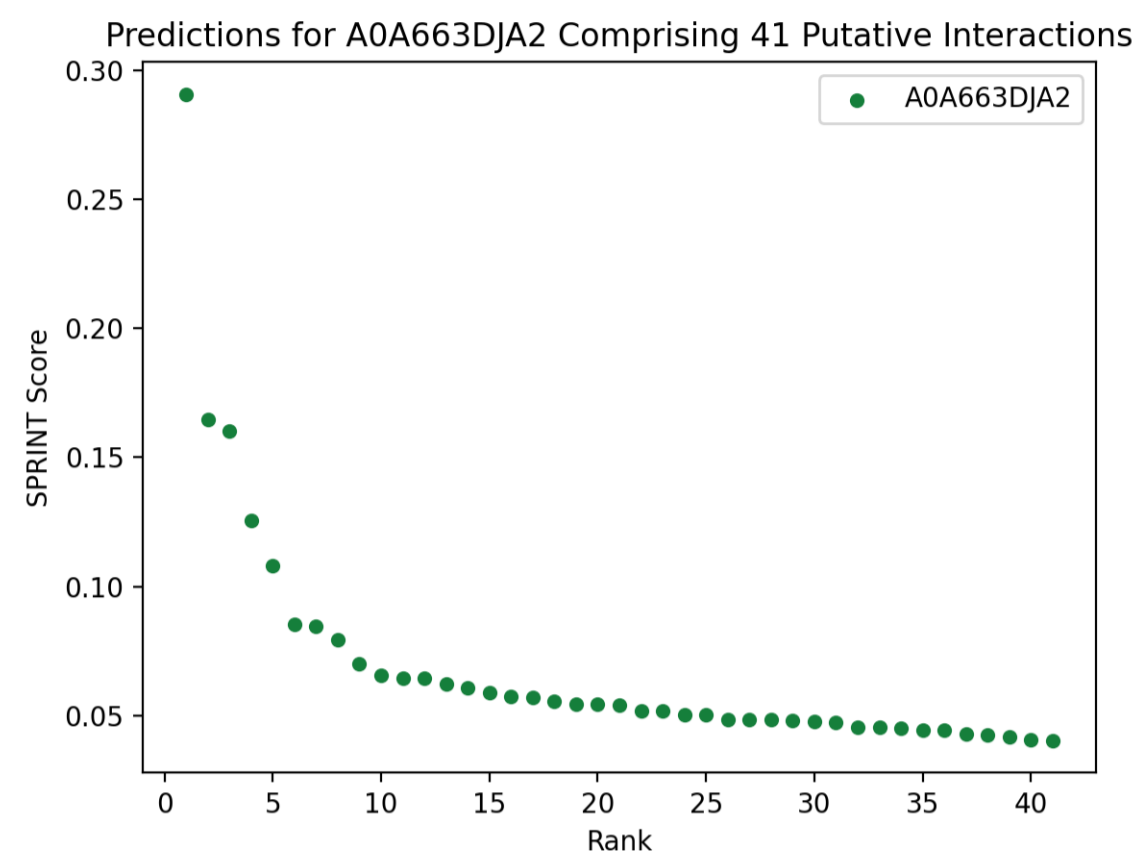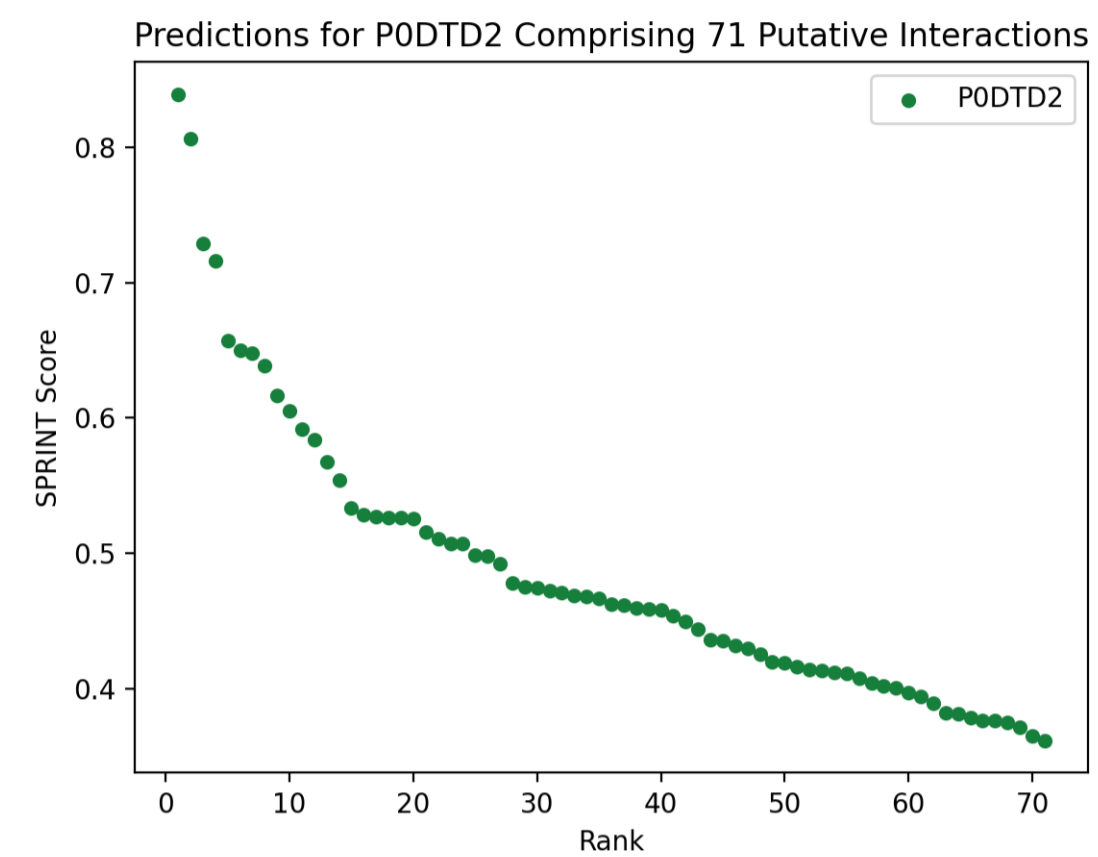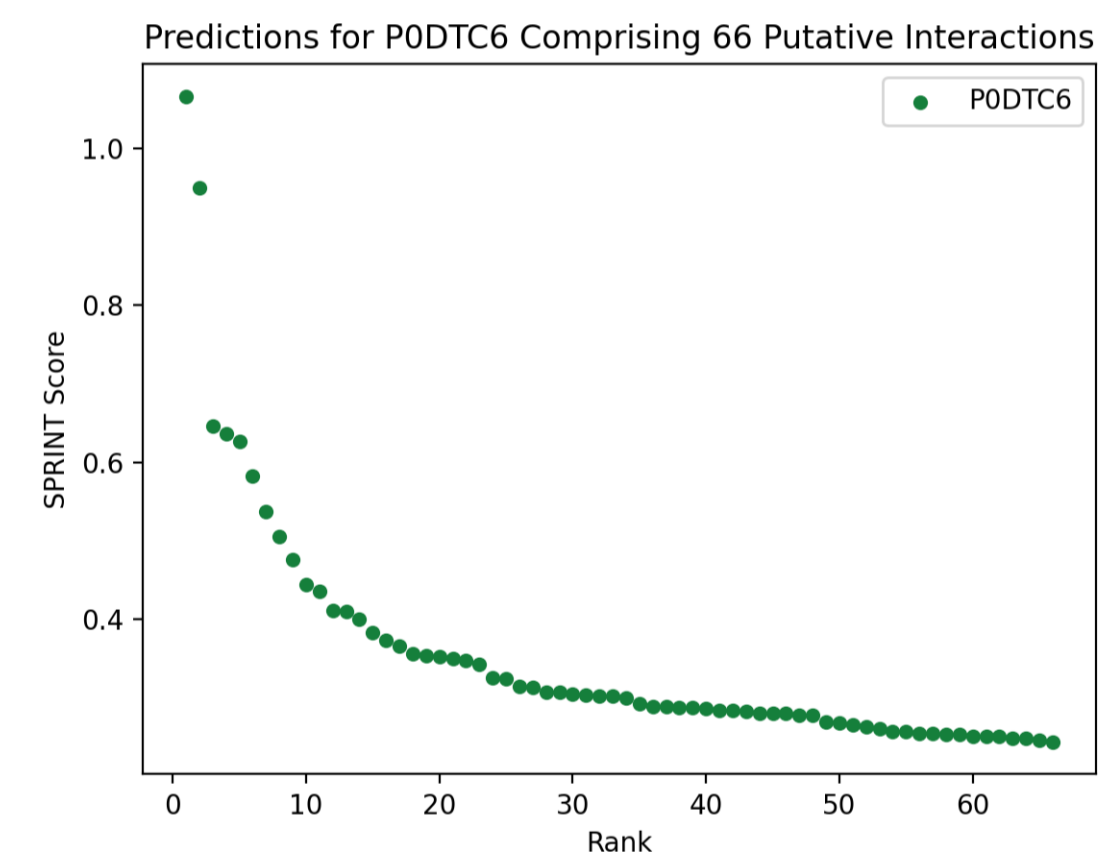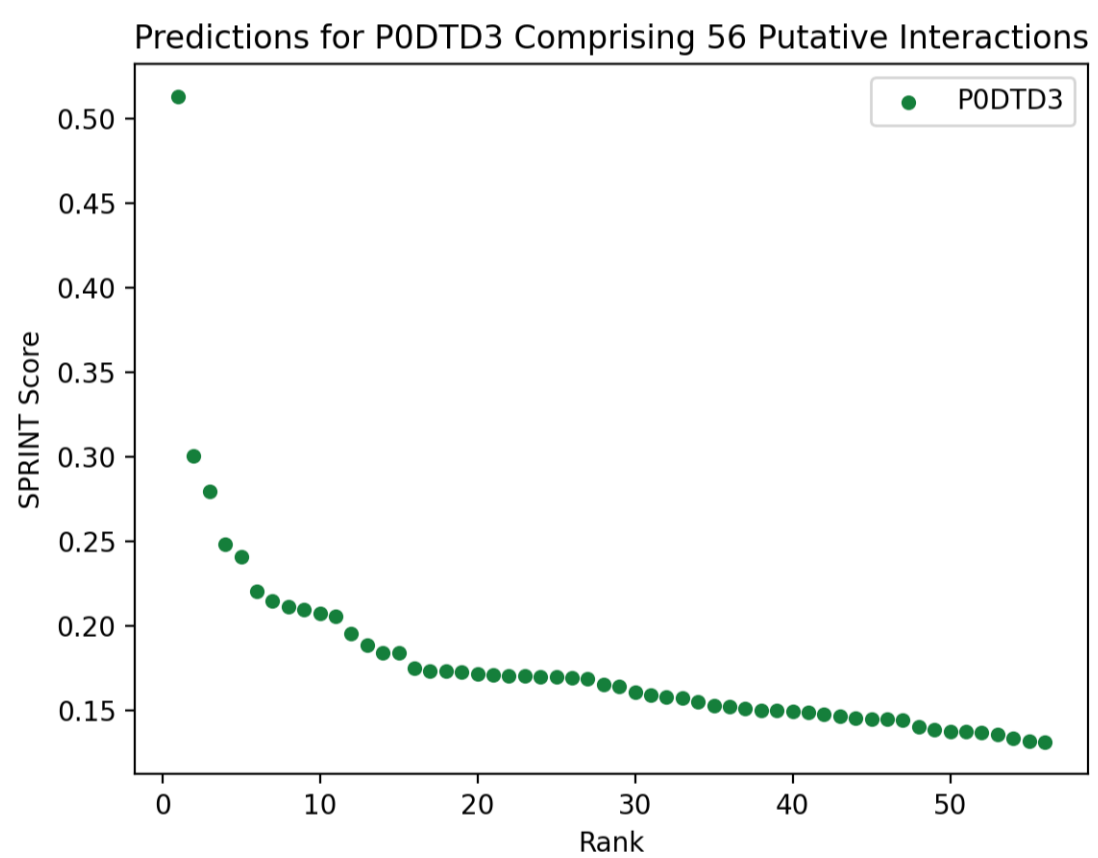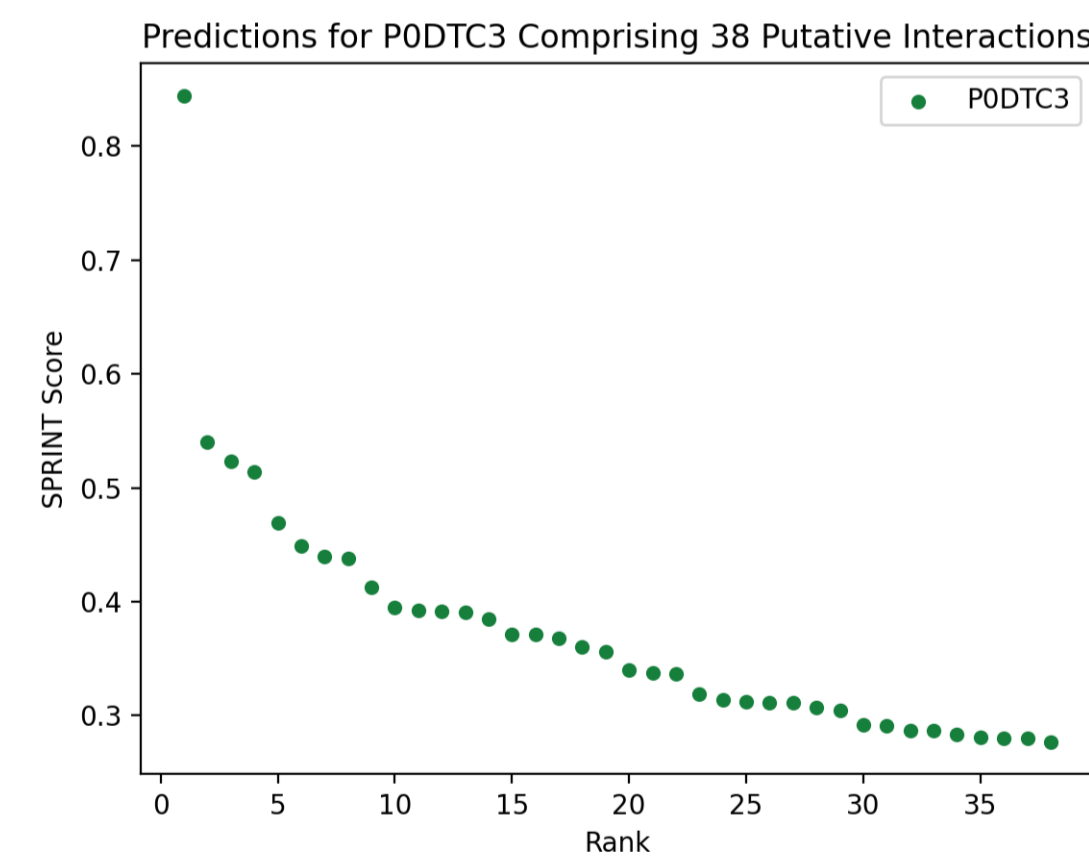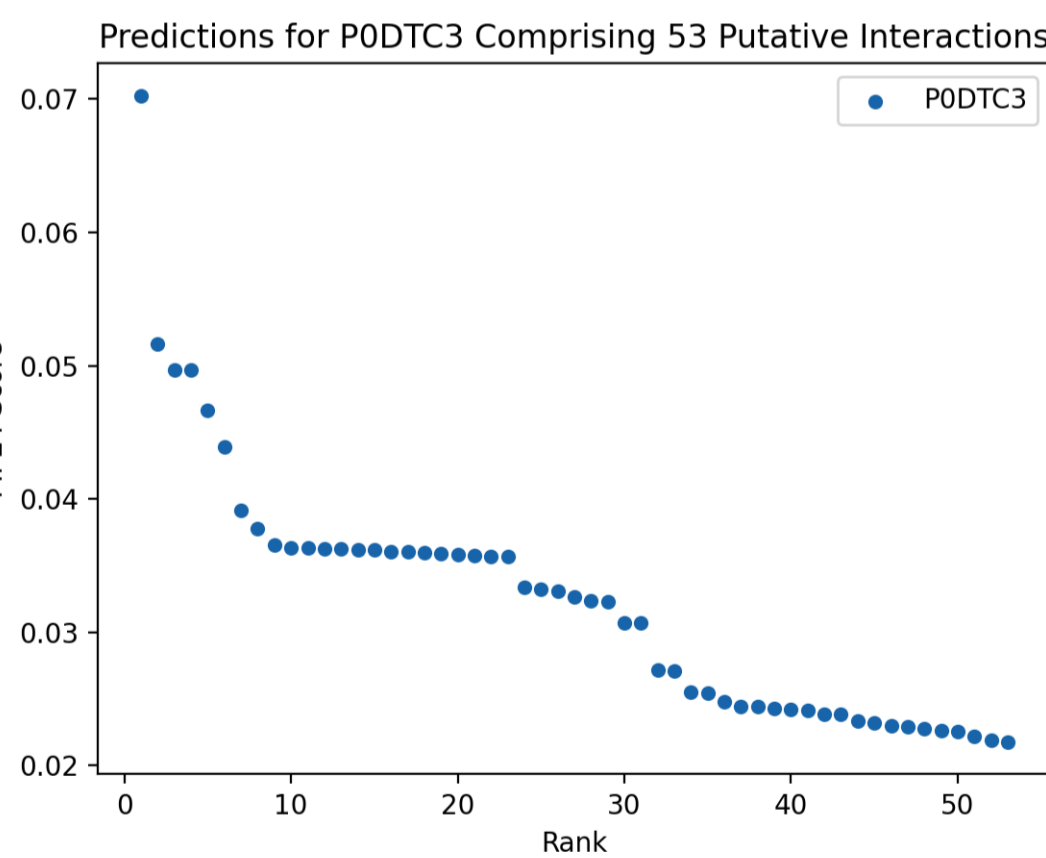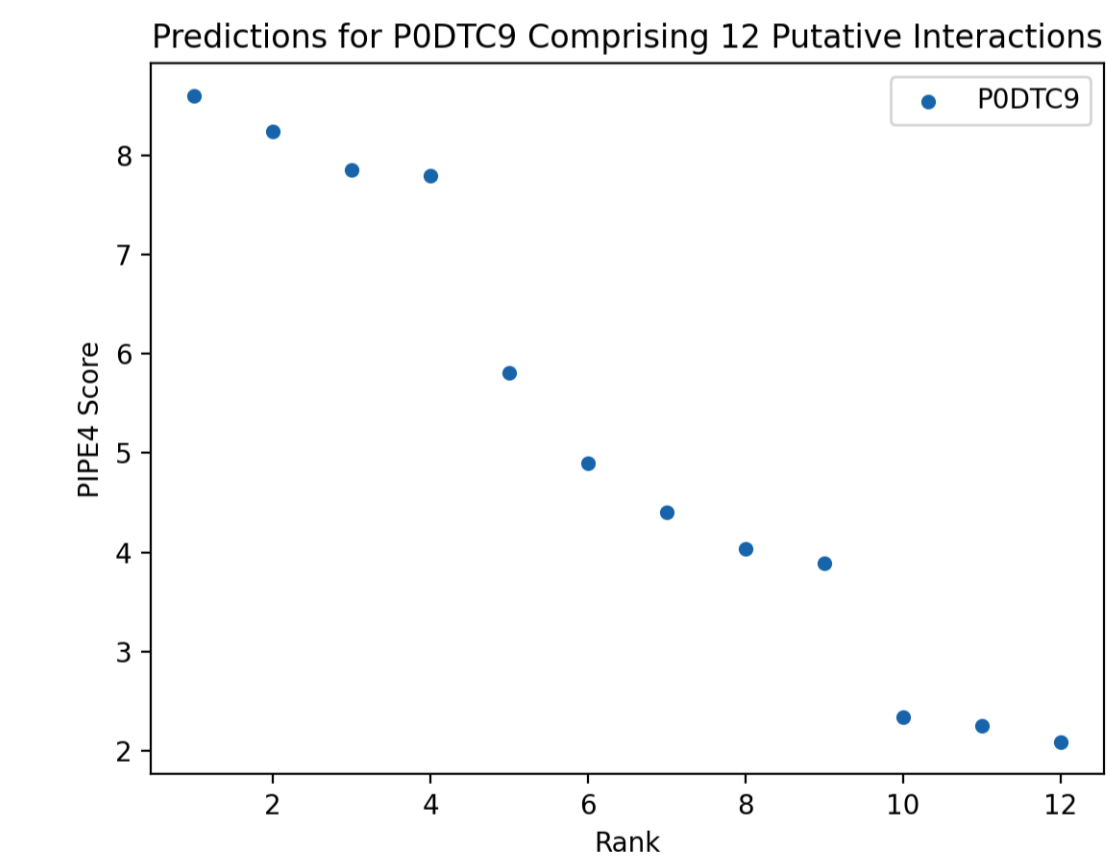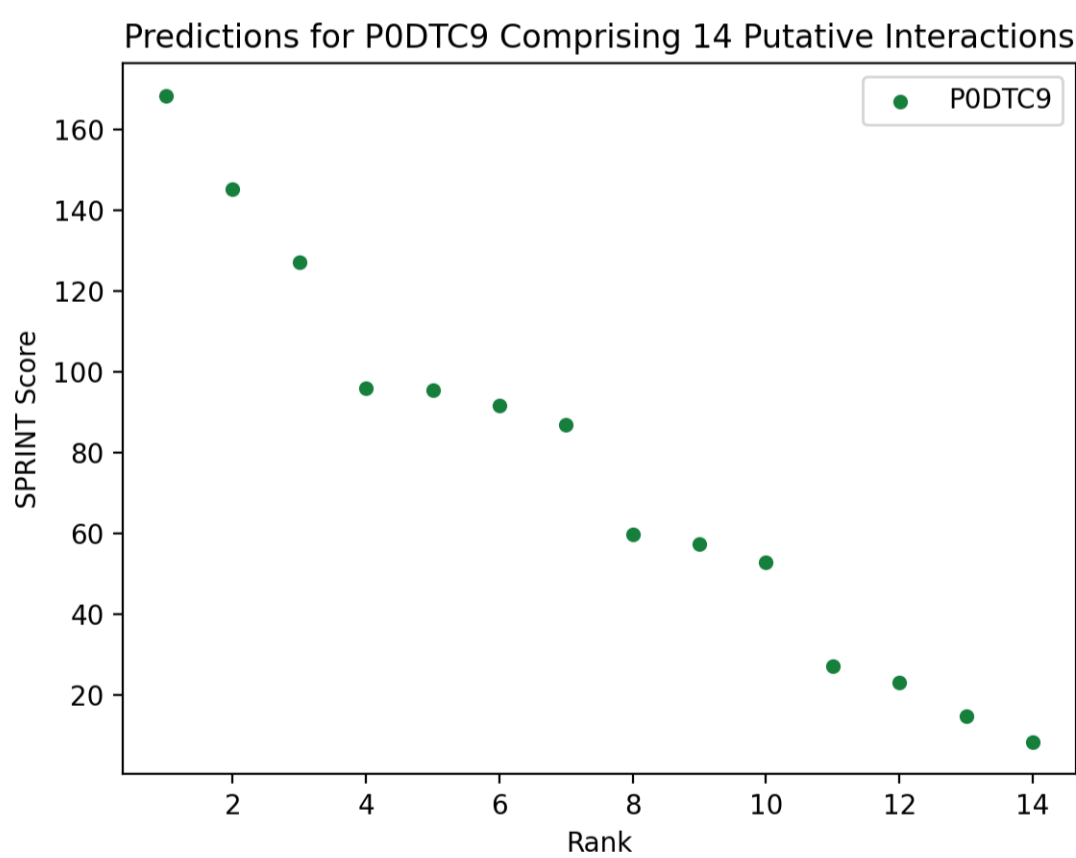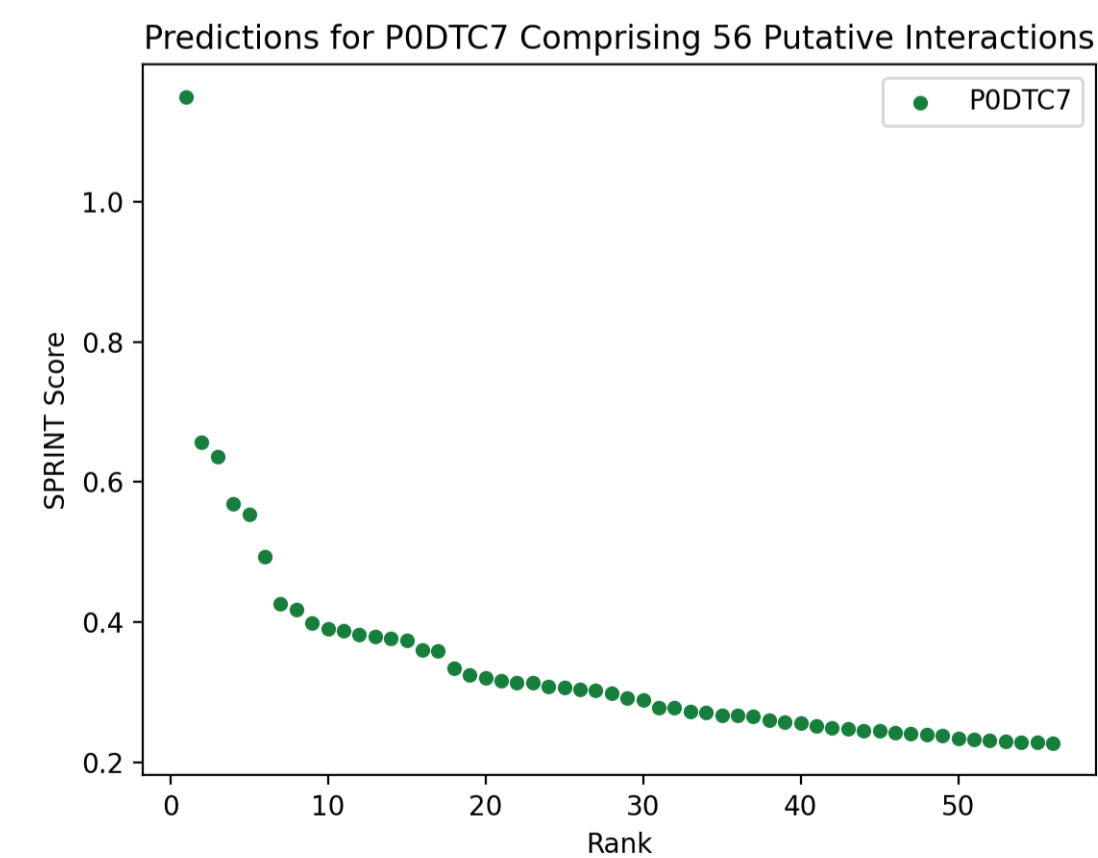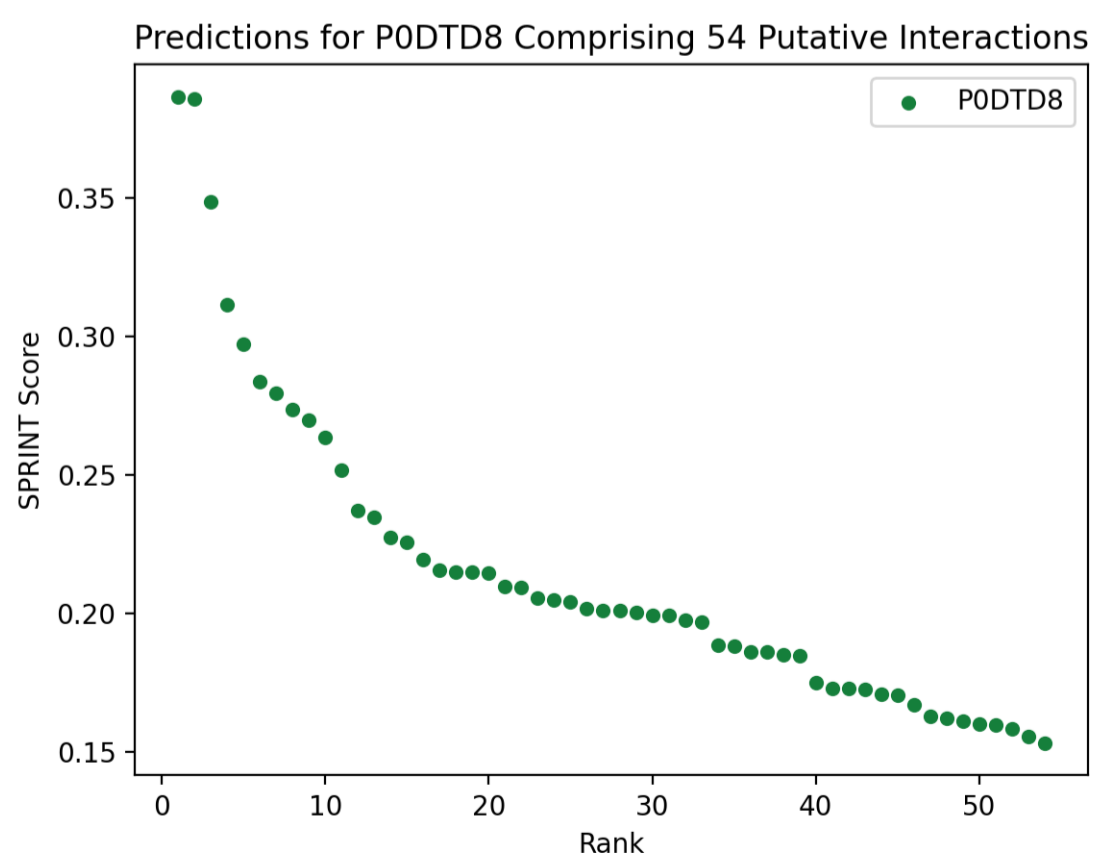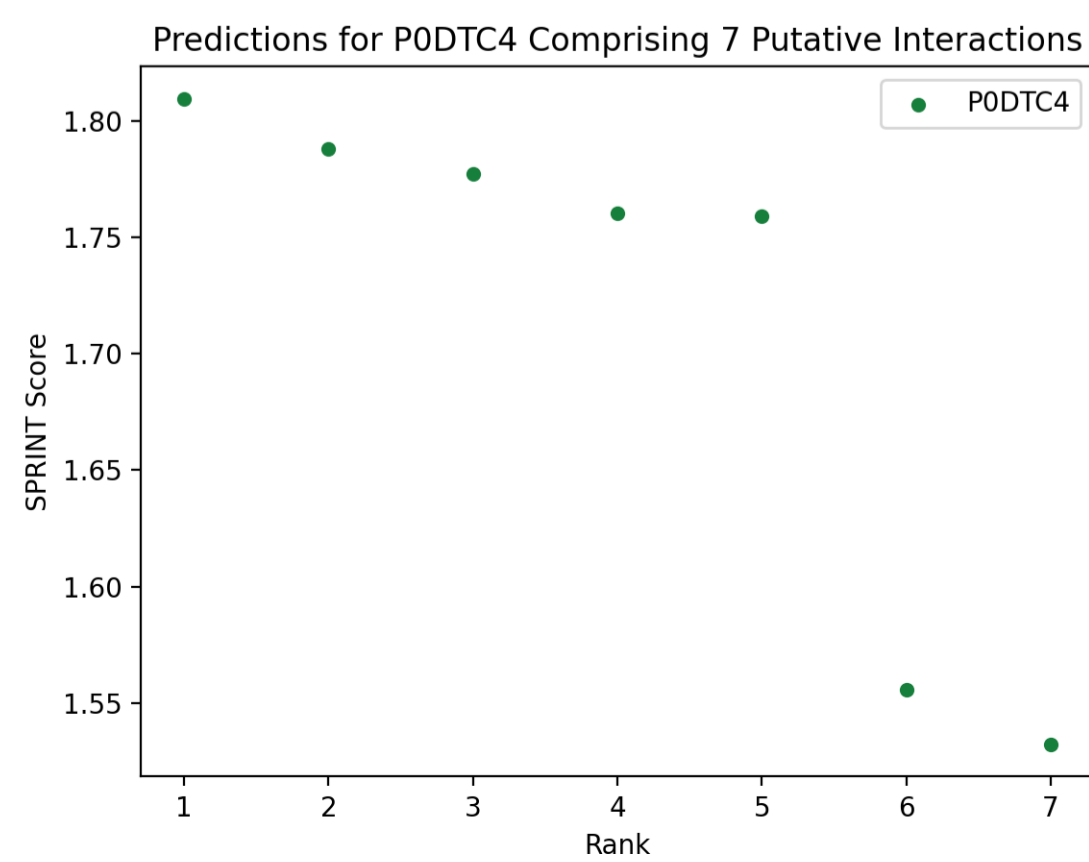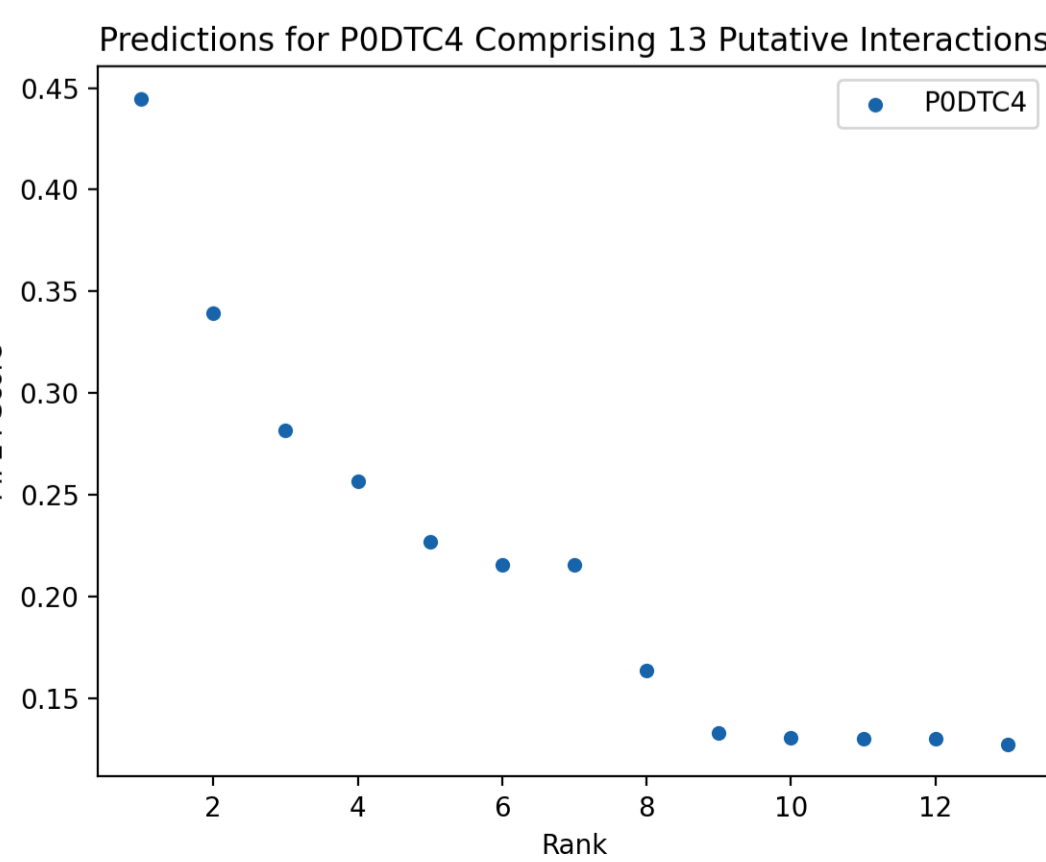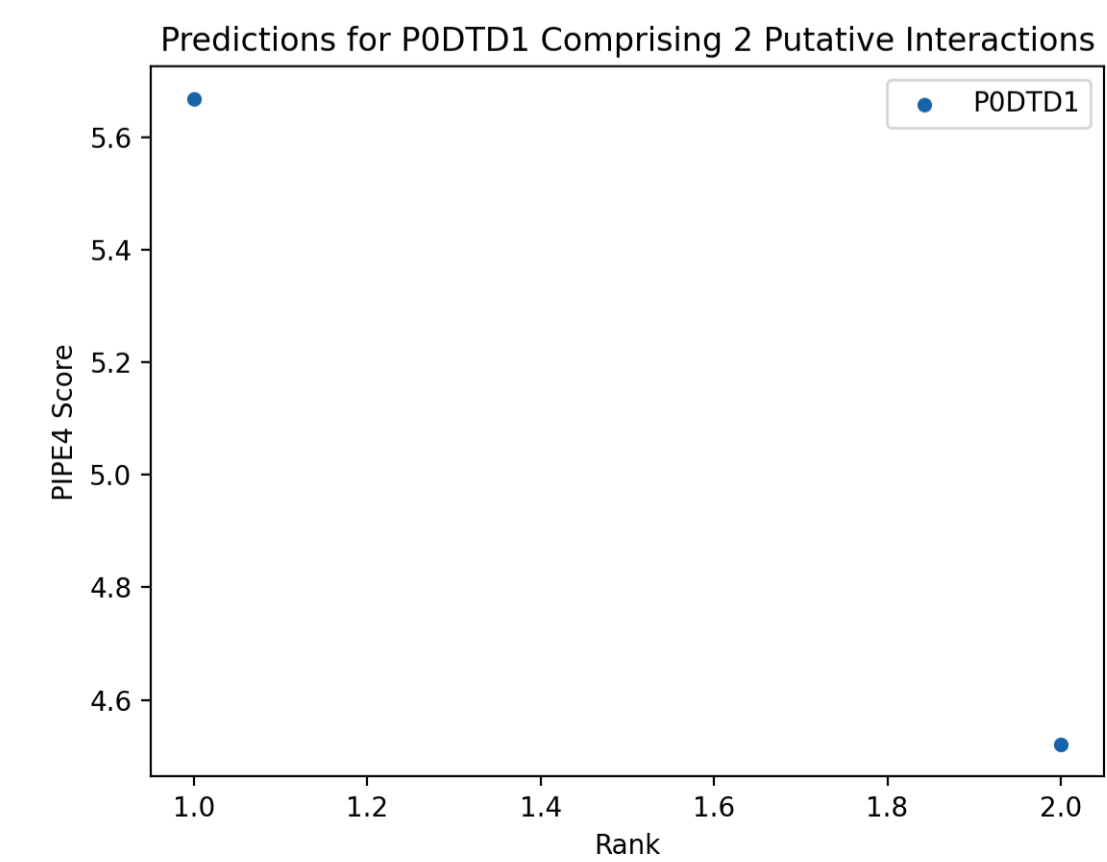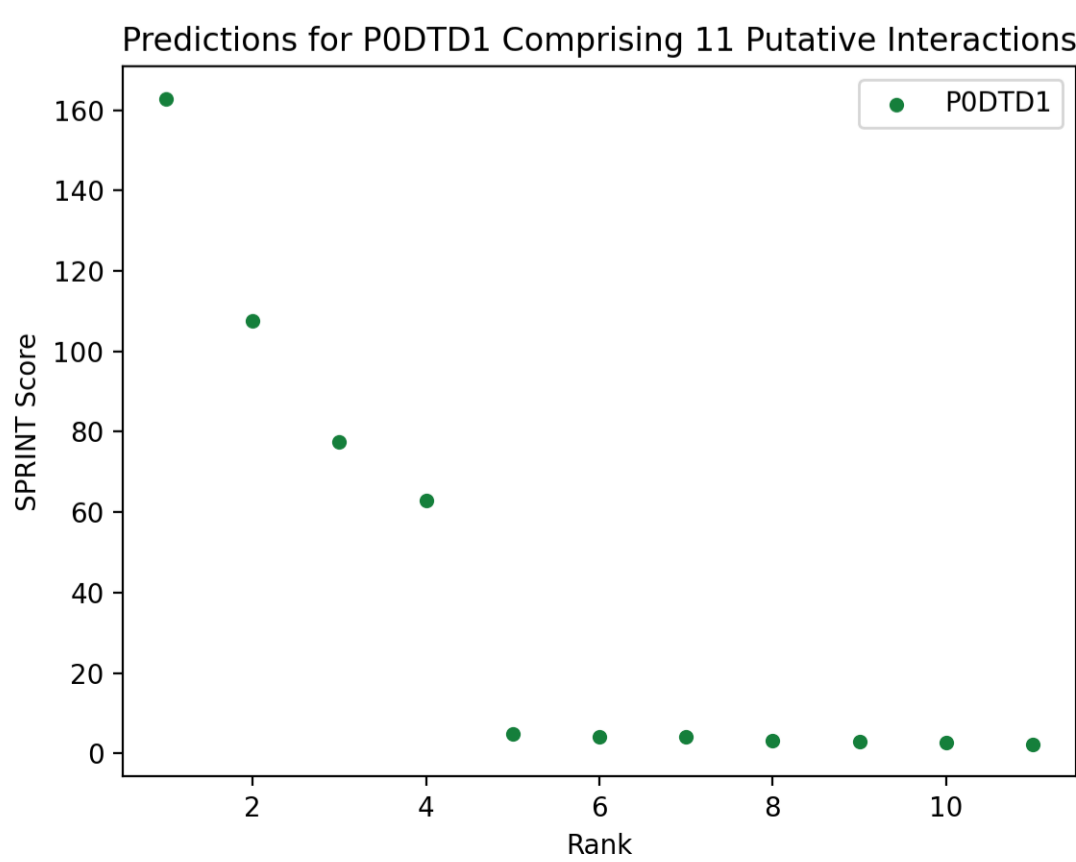

Supplement: Supplemental Information 13 [file peerj-09-11117-s013.pdf]
