# Supplementary material for: Multi-schema computational prediction of the comprehensive SARS-CoV-2 vs. human interactome
Source: PeerJ. 2021 Apr 5;9:e11117. doi: 10.7717/peerj.11117 (PMC8029698; doi:10.7717/peerj.11117)
Supplement: Supplemental Information 5 — The combined use of PIPE4 and SPRINT features within the RP-PPI Joint model depicts an overall average improvement in performance. Interestingly, the improvement does not appear to be symmetric: the improvement of performance when SPRINT features are joined with the PIPE4 features (A, blue & grey) is greater than when the PIPE4 features are joined with SPRINT features (B, blue & grey). [file peerj-09-11117-s005.pdf]

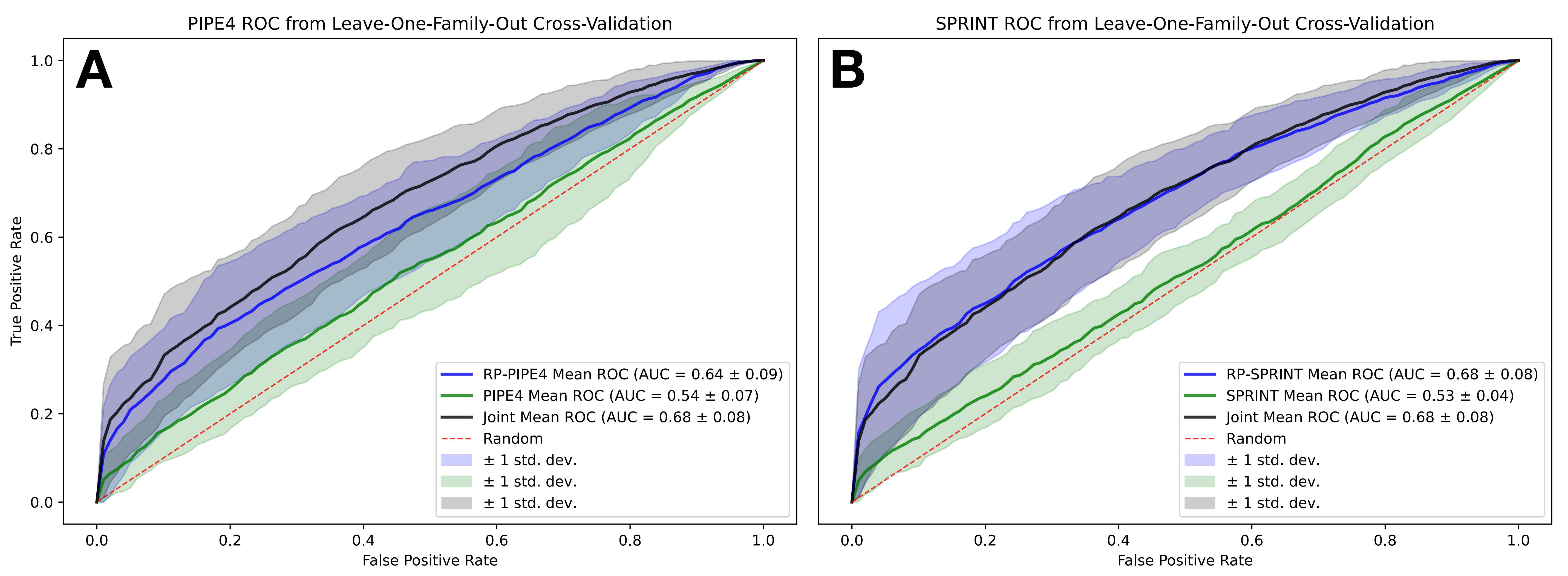

PIPE4 ROC from Leave-One-Family-Out Cross-Validation
A
True Positive Rate
False Positive Rate
RP-PIPE4 Mean ROC (AUC = 0.64 ± 0.09)
PIPE4 Mean ROC (AUC = 0.54 ± 0.07)
Joint Mean ROC (AUC = 0.68 ± 0.08)
Random
± 1 std. dev.
± 1 std. dev.
± 1 std. dev.
SPRINT ROC from Leave-One-Family-Out Cross-Validation
B
False Positive Rate
RP-SPRINT Mean ROC (AUC = 0.68 ± 0.08)
SPRINT Mean ROC (AUC = 0.53 ± 0.04)
Joint Mean ROC (AUC = 0.68 ± 0.08)
Random
± 1 std. dev.
± 1 std. dev.
± 1 std. dev.
